# On the Mechanism of the Formal [2+2] Cycloaddition – Retro‐electrocyclization (*CA‐RE*) Reaction

**DOI:** 10.1002/chem.202202833

**Published:** 2022-11-22

**Authors:** Jonathan Kirschner Solberg Hansen, Christian G. Tortzen, Preben Graae Sørensen, Mogens Brøndsted Nielsen

**Affiliations:** ^1^ Department of Chemistry University of Copenhagen Universitetsparken 5 2100 Copenhagen Denmark

**Keywords:** autocatalysis, cycloaddition, kinetics, NMR spectroscopy, retro-electrocyclization

## Abstract

The [2+2] cycloaddition ‐ retro‐electrocyclization (*CA*‐*RE*) reaction is a “click‐like” protocol for facile synthesis of donor‐acceptor chromophores from an alkyne and tetracyanoethylene. Herein we shed light on the mechanism of this reaction by detailed kinetics studies using ^1^H NMR spectroscopy. By considering several experiments simultaneously, a variety of mechanistic models was evaluated. Surprisingly, a model in which the final 1,1,4,4‐tetracyanobuta‐1,3‐diene product promoted the first step was the only one that described well the experimental data. This autocatalysis model also involved a non‐concerted, stepwise formation of the cyclobutene cycloaddition adduct. By proper choice of conditions, we were able to generate the transient cyclobutene in sufficient amount to verify it as an intermediate using ^13^C NMR spectroscopy. For its final retro‐electrocyclization step, simple first‐order kinetics was observed and only minor solvent dependence, which indicates a concerted reaction.

## Graphical abstract

**The “click‐like” [2+2] cycloaddition** ‐ retro‐electrocyclization (*CA*‐*RE*) reaction was followed under a variety of conditions using NMR spectroscopy. Several mechanistic models were evaluated by considering multiple experiments simultaneously. Surprisingly, only a model in which the final 1,1,4,4‐tetracyanobuta‐1,3‐diene product promoted the first step described well all the data. The *CA‐RE* reaction is hence an autocatalytic reaction.

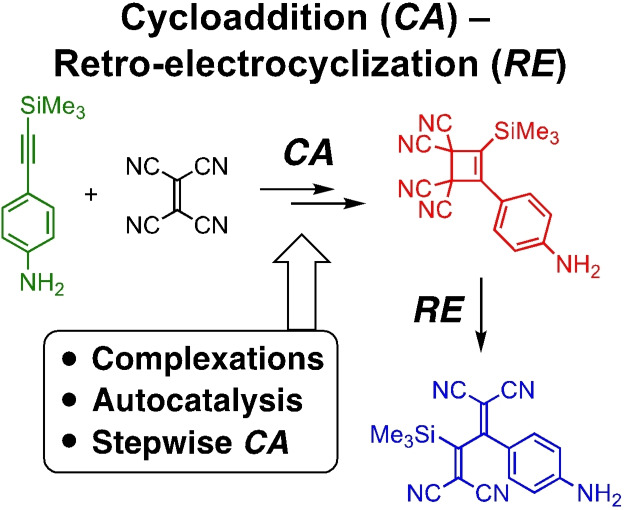

## Introduction

**Scheme 1:**
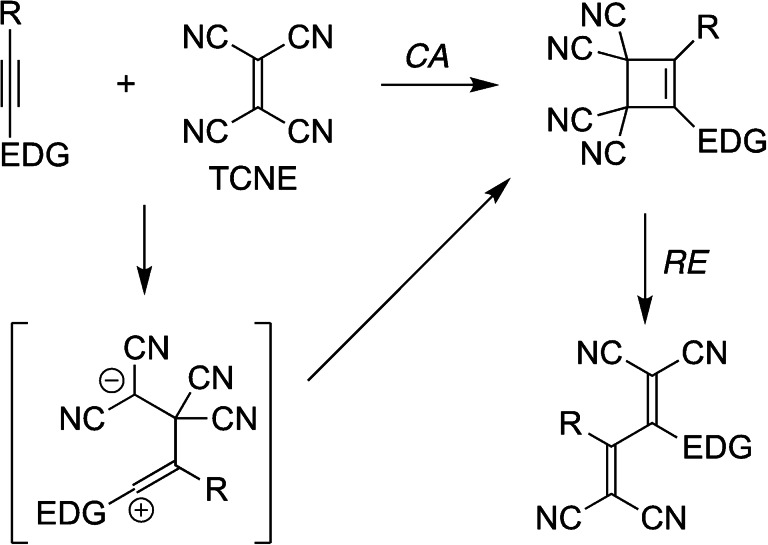
Cycloaddition (*CA*) ‐ retro‐electrocyclization (*RE*) reaction. EDG=electron‐donating group.

The [2+2] cycloaddition ‐ retro‐electrocyclization (*CA*‐*RE*) reaction between the strong electron acceptor tetracyanoethylene (TCNE) and an alkyne containing an electron‐donating substituent group (EDG) is an efficient and atom‐economic protocol for the synthesis of 1,1,4,4‐tetracyanobuta‐1,3‐diene derivatives with no side‐products being formed (Scheme 1). This “click‐like” reaction was first reported in 1981 by Bruce and co‐workers^[1]^ employing electron‐rich ruthenium‐acetylide complexes as alkyne substrates and later expanded to electron‐rich alkynylferrocene derivatives^[2]^ as well as purely organic alkynes.[^3^, ^4^] Diederich and co‐workers^[3]^ systematically explored the reaction for the synthesis of a large variety of donor‐acceptor chromophores using anilino‐substituted alkynes as substrates and also expanded the reaction to less electron‐poor di‐ and tricyanoethylenes.^[5]^ A computational study on the reaction between 1,1‐dicyanoethylene and 4‐ethynyl‐*N*,*N*‐dimethylaniline suggests a stepwise cycloaddition step via a zwitterionic intermediate.^[3b]^ In a detailed experimental and computational study, it was found that the reaction between 4‐(*N*,*N*‐dimethylamino)phenylacetylene and *para*‐substituted benzylidenemalononitriles in polar solvents followed bimolecular, second‐order kinetics.^[5]^ The rate‐determining step was also in this case suggested to be a nucleophilic attack of the terminal alkyne carbon onto the dicyanovinyl electrophile, furnishing a transient zwitterionic intermediate that subsequently underwent ring closure to form the cyclobutene intermediate. A zwitterionic intermediate was also moved forward by Trolez and co‐workers in the reaction between an anthracene‐ynamide derivative and TCNE.^[4i]^ The stepwise mechanism as an alternative to direct and concerted [2+2] cycloaddition is shown in Scheme 1 for TCNE. However, as stated by Diederich and co‐workers,^[5]^ the reaction between 4‐(*N*,*N*‐dimethylamino)phenylacetylene and TCNE was inconclusive as to whether the reaction is first or second order, and the actual mechanism for this reaction is still puzzling.

We became interested in shedding further light on this reaction that has found so widespread use for facilely accessing molecular or polymeric donor‐acceptor push‐pull chromophores, and here we present kinetics data obtained by NMR spectroscopic studies. Data from experiments performed under different conditions were altogether fed into a custom‐made program, and different mechanistic models were evaluated. Ultimately, we found that a model involving autocatalysis described the experimental data excellently.

## Results and Discussion

**Scheme 2:**
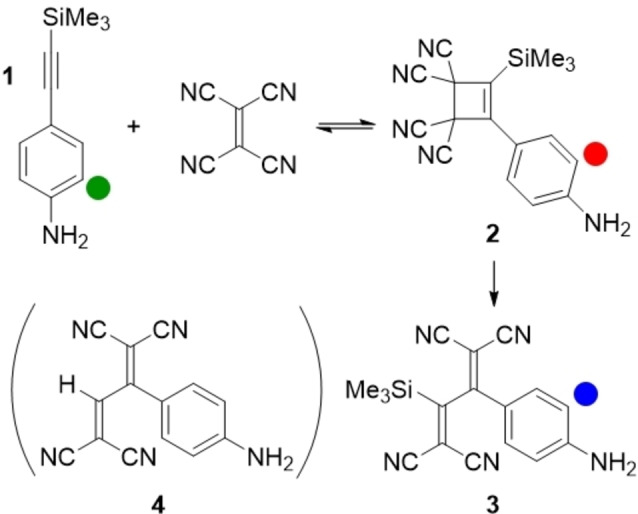
*CA‐RE* reaction subject to study with the three aryl species shown that can be detected by NMR spectroscopy (protons used as probes for kinetics studies are labelled).

**Scheme 3:**
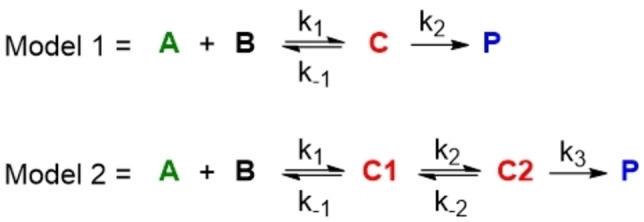
Models 1 and 2 used for fits shown in Figure 4 (**C2** corresponds to **C**).

We chose the anilino‐substituted alkyne **1** shown in Scheme 2 as the key substrate for the study of the *CA*‐*RE* reaction. This substrate was chosen as it would allow not only ^1^H‐ and ^13^C NMR spectroscopic studies, but also ^29^Si NMR spectroscopic studies on account of the SiMe_3_ group. A Si(*i*‐Pr)_3_ end‐group was discarded due to its bulkiness. Upon treatment with TCNE, **1** should form the presumed intermediate **2** in a cycloaddition that may occur either concerted (as shown) or asynchronously (via a transient zwitterion as shown in Scheme 1). A subsequent retro‐electrocyclization finally provides the product **3**. On the NMR timescale, we can only detect three aryl species (see below): the alkyne substrate **1**, the presumed cyclobutene intermediate **2**, and the product **3**; when using ^13^C NMR spectrosocopy, TCNE can also be detected. The species are simplistically also labelled with letters **A** (=**1**), **B** (TCNE), **C** (=**2**), and **P** (=**3**) in the concerted model (Model 1) shown in Scheme 3. For a stepwise mechanism (Model 2) also shown in Scheme 3, we label the two intermediates **C1** (corresponding to the zwitterion) and **C2** (corresponding to the cyclobutene **2**, labeled **C** in Model 1).

**Figure 1:**
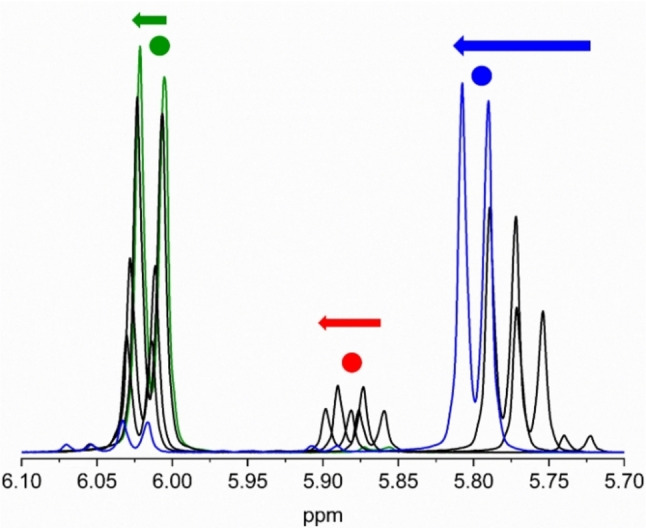
Five selected superimposed ^1^H NMR spectra (selected region 6.10–5.70 ppm; 500 MHz, C_6_D_6_) of the reaction mixture between **1** and TCNE (ratio of 1 : 1) recorded at times 0 min (green spectrum; compound **1**), 50 min, 3.3 h, 5.4 h (black spectra), and 15 h (blue spectrum; product **3**). For the assignments (aryl protons), see Scheme 2. Each of the 3 doublet signals shifts position over the duration of the experiment indicated by arrows.

**Figure 2:**
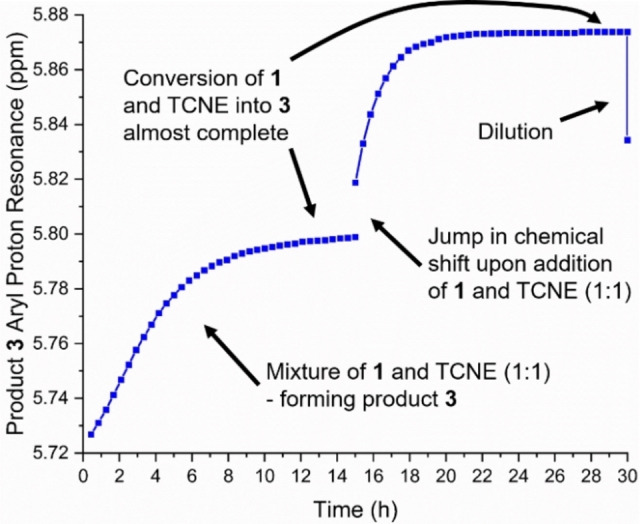
Proton chemical shift of the aryl protons *ortho* to the amino group in product **3** followed in time (300 K); start: reaction mixture of **1**/TCNE in a ratio of 1 : 1 in C_6_D_6_; after ca. 16 h: new addition of **1** and TCNE; after ca. 30 h: dilution of the reaction mixture.

**Figure 3:**
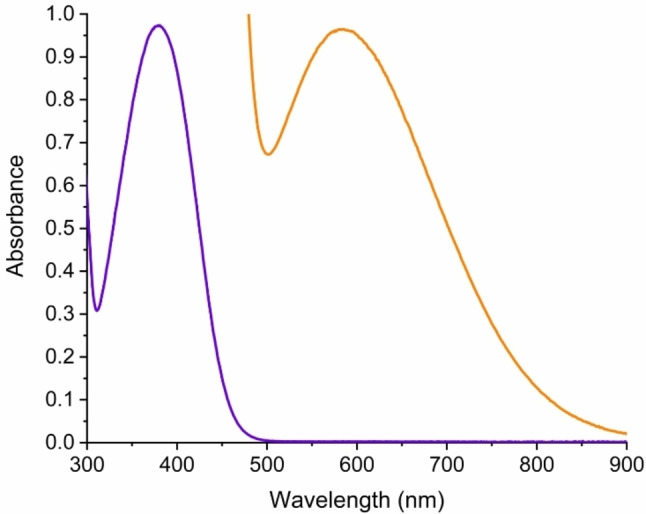
UV‐Vis absorption spectra (in C_6_H_6_) of a 1 : 1 mixture of aniline and TCNE in concentrations of 3.22 mM (purple curve) and 14.2 mM (orange curve; only region from above 500 nm is shown here, corresponding to the charge‐transfer absorption band of the TCNE•aniline complex).

First, the reaction works well in various solvents, such as CD_3_CN, CDCl_3_, C_6_D_6_, and C_6_D_5_CD_3_. Yet, in CD_3_CN and other polar solvents, protodesilylation was found to occur readily, providing the product **4** (Scheme 2), while this reaction was almost fully avoided in the other solvents. Protodesilylation also occurred upon isolation of the product as previously observed for the related *N*,*N*‐dimethylaniline derivative.^[3b]^ A complete list of all tested solvents can be found in the Supporting Information (Supporting Information, p. 6). In deuterated chloroform and toluene, inconvenient signal overlaps between substrate, products, and residual non‐deuterated solvent occur (see Supporting Information, p. S37 and S45), while only two signals were found to overlap with the solvent signal in deuterated benzene. For this reason, we focused our ^1^H NMR spectroscopic studies to C_6_D_6_. Selected ^1^H NMR spectral regions over time are shown in Figure 1; they reveal the build‐up and disappearance of an intermediate that we tentatively assign to **2** and a build‐up of a product which we assign to **3**. The same progress is observed when following the NH_2_ and trimethylsilyl resonances (see Supporting Information, p. S49). Interestingly, both the aryl, NH_2_ and to a lesser extent trimethylsilyl proton resonances of **1**, **2**, and **3** move slightly during the reaction progress, which is clearly evident when expanding the spectra (Figure 1 and Supporting Information, p. S51). This change is most significant for the amino protons of **3** that move ca. 0.2 ppm downfield as the reaction proceeds (see Supporting Information, p. S51**)**. Figure 2 shows the change in chemical shift of the aryl protons *ortho* to the amino group of product **3** during the reaction, after adding more reactants, and finally after diluting the product sample. The evident changes suggest the presence of some fast exchanging species on the NMR timescale with a weight‐averaged chemical shift (of particular importance for the analysis of our kinetics data, see below). It is well‐known that aniline and TCNE can form charge‐transfer complexes,^[6]^ and such associations are likely to occur in the reaction mixture. Figure 3 shows two UV‐Vis absorption spectra of 1 : 1 mixtures of aniline and TCNE, but at different concentrations, in benzene. For the spectrum recorded at high concentration, a charge‐transfer absorption band is indeed observed at *λ*_max_ 584 nm. From a series of measurements at different concentrations, we obtain an association constant for the aniline•TCNE complex of 2 M^−1^ (see Supporting Information, p. S114–S116). For comparison, the association constant in dichloromethane has previously been determined to 5.4 M^−1^ (ref. [6a]; *λ*_max_ 610 nm) and to 2–3 M^−1^ (ref. [6b]). Finally, we note that also alkynes are known to form very weak complexes with TCNE.^[7]^ Also the solvent itself, benzene, will form a complex with TCNE.^[7c]^

## Kinetics Studies at Various Substrate Ratios

The reaction progress was followed by ^1^H NMR spectroscopy using different ratios between the aniline‐substituted alkyne **1** and TCNE; i.e., **1**/TCNE ratios of 1 : 1, 1 : 2, 1 : 5 and 2 : 1 (solvent: C_6_D_6_; temperature 300 K). The spectra were recorded with an internal standard (cyclohexane) in a known concentration, from which the concentrations of all species were calculated using the most upfield aryl signals (where no interfering solvent signal is present); the corresponding protons used for analysis are labelled in Scheme 2.

**Figure 4:**
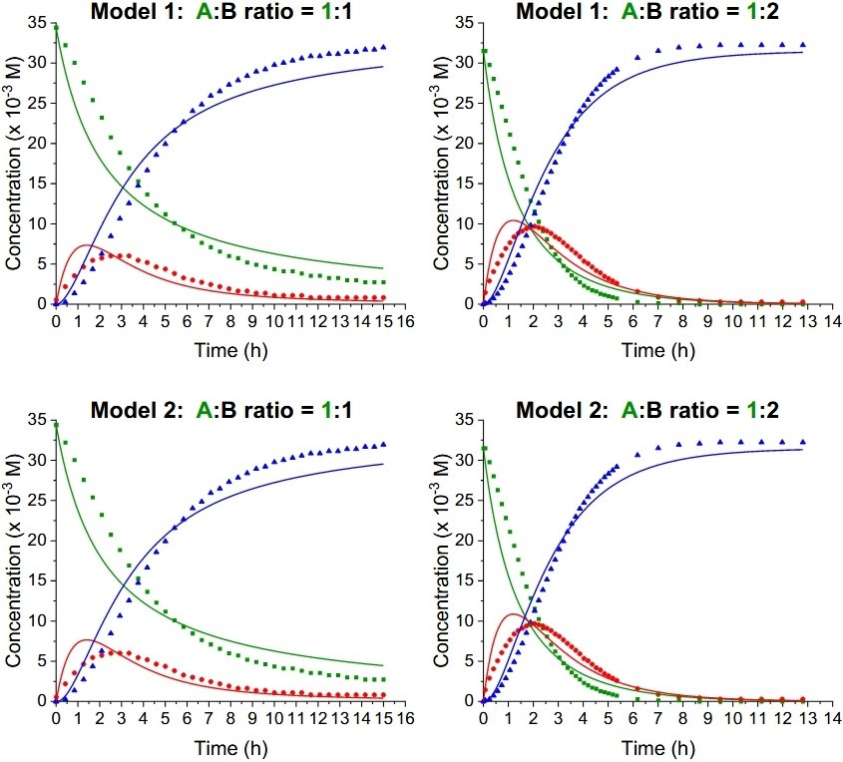
Plots showing the disappearance in time of substrate **1** (also labelled **A**; green square), appearance and disappearance of intermediate **2** (also labelled **C**; red circle), and formation of product **3** (also labelled **P**; blue triangle) over time at two different substrate ratios (**A : B**; **B**=TCNE). The fits using Model 1 (top) and Model 2 (bottom) were constructed using four different substrate ratios (and hence not only using the two shown here; see Supporting Information, p. S120–S125).

By fitting the simple Models 1 and 2 outlined in Scheme 3 to the recorded data from the four different substrate ratio experiments, the best possible rate constants were obtained for each model using a custom‐made kinetics program. The program repeatedly scans values of rate constants until the sum of the deviations between simulated and experimental concentrations of **A**, **C**, and **P** for Model 1 and **A**, **C_1_** +**C_2_**, and **P** for Model 2 is as small as possible. A detailed explanation and walk‐through of the program is described in Supporting Information, p. S2, and a separate article describing the code will be published in a suitable journal at a later stage. Importantly, the advanced fitting takes all four experiments into account simultanenously. Considering data from each experiment individually could generate better, near‐perfect individual fits, but it would give non‐identical rate constants between the experiments. A couple of selected fits are shown in Figure 4 for two of the substrate ratios using either Model 1 (top) or Model 2 (bottom) (the combined fits for all four substrate ratios are shown in Supporting Information, p. S120). It transpires that these models do not fit the data very well. This result is in line with the statement by Diederich and co‐workers^[5]^ that the reaction does not seem to be a simple second‐order reaction. We also took a weak association between **1** and TCNE into account in our fitting, but this model would not either give a good result.

Our experimental data indicate that the intermediate **C** (=**2**) builds up slower than the models 1 and 2 are accounting for. To figure out the reason for this, we performed systematic studies where conditions were slightly changed, except for keeping the initial concentrations of the two starting materials close to identical (unless otherwise specified). An overview of all the experiments are provided in Supporting Information, p. S5 and S6. Here we shall only discuss selected ones. First, we tested whether a spinning or non‐spinning sample would make a difference. A comparison between experiments with a spinning sample and non‐spinning sample can be seen in Supporting Information, p. S55; there is no noticeable difference between the two. We then tested whether the presence of water would have an effect; samples of different (**1**/TCNE) ratios of 1 : 1, 1 : 2 and 2 : 1 in anhydrous C_6_D_6_ and in C_6_D_6_ saturated with water (see Supporting Information, p. S74–S75) were studied. The accumulation of the intermediate saw no difference between dry and wet samples, but protodesilylation to compound **4** occurred in all wet samples and to a smaller degree in the anhydrous 2 : 1 ratio experiment. We note that actual isolation of product **3** failed as it is readily desilylated to furnish **4** after exposure to air for a short period of time. Based on the wet/dry experiments together with our failed attempt to isolate **3**, we conclude that **3** is highly sensitive to water and will form **4** if water is present, but this sensitivity is not critical for studying the actual *CA*‐*RE* reaction of interest here.

## Effect of Additives

A range of acid/base additive experiments was also conducted. As base additives, we used a drop of Et_3_N, 1,8‐diazabicyclo[5.4.0]undec‐7‐ene (DBU), and a spatula tip of Na_2_CO_3_ in separate experiments. Both Et_3_N and DBU resulted in a black tar of decomposed TCNE, most likely resulting from a nucleophilic attack. The addition of Na_2_CO_3_ gave no apparent change of the outcome of the reaction.

Next, we added trifluoroacetic acid (TFA) in various equivalents, but such additions would always result in immediate deprotection of the trimethylsilyl group. A comparison of the effect of addition of instead AcOH in various equivalents can be seen in Supporting Information, p. S87. These experiments showed only slightly faster intermediate formation with more equivalents of AcOH. To further investigate the effect of acetic acid, we also used AcOD‐*d_4_* as the solvent for the reaction. This actually gave a massive increase in reaction rate but we reckon that this result originates from an increased polarity of the solvent rather than the acidic properties of AcOD‐*d_4_*. The polarity influence could indicate a stepwise cycloaddition mechanism involving a transient zwitterion. In actual fact, the acid properties of acetic acid retard the *RE* step (see below), but this step is not rate‐determining. The reaction was also found to be much faster in CD_2_Cl_2_ relative to benzene, but quantitative experiments were difficult to perform reliably on account of the volatility of this solvent. The reaction progress from an experiment using CD_2_Cl_2_ as solvent can be seen in Supporting Information, p. S100. The intermediate is formed almost instantaneously. An immediate color change to blue is also observed when mixing together the reactants in this solvent, ascribed to formation of charge‐transfer complexes (see Supporting Information, video recordings). We tested a model using H^+^ as a catalyst for the first step, but once again no good fit could be obtained based on all four experiments. The motivation for investigating such a model was that both the Si and the NH_2_ group could potentially promote protonation of the carbon carrying the SiMe_3_ group; Si via stabilization of the resulting carbocation at the neigboring carbon (hyperconjugation) and NH_2_ by forming via its lone pair electrons an allene resonance form that could engage in the cycloaddition. Lastly, we used a buffer system with NH_4_OAc in C_6_D_6_ but it neither inhibited nor enhanced the reaction in any way and only resulted in formation of **4**.

As discussed earlier, TCNE can form charge‐transfer complexes with aniline, and we therefore performed an experiment with aniline as additive in a 1 : 2 : 1 ratio of **1**/TCNE/aniline. This resulted in a noticable slower formation of intermediate **2** (**C**) compared to conditions using a ratio of 1 : 2 (**1**/TCNE), but still faster than conditions using a ratio of 1 : 1 (**1**/TCNE). A smaller build‐up of **C** suggests that competing charge‐transfer complexes greatly inhibit the reaction. The effect can be seen in Supporting Information, p. S98.

## Kinetics Studies with Product Present from the Beginning

**Figure 5:**
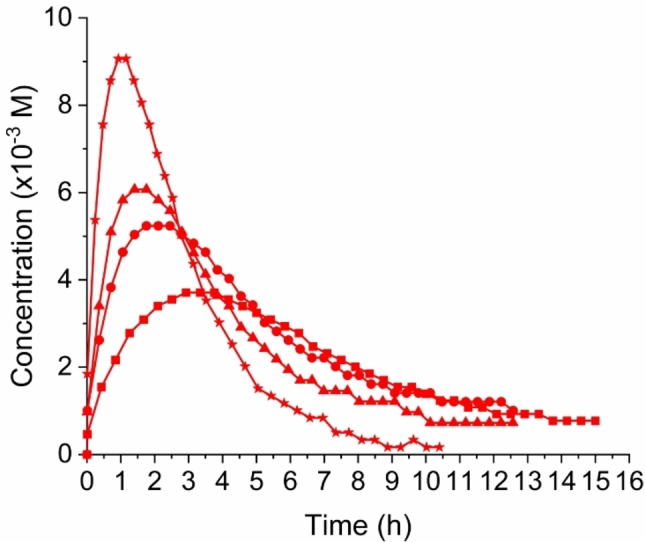
Build‐up and decay of intermediate **2** in four experiments with a 1 : 1 **A : B** mixture (**1**/TCNE), each in starting concentrations of 0.0272 M and with various concentrations of product present from the beginning: 0 M (square), 0.00573 M (circle), 0.0115 M (triangle) and 0.0272 M (star). For the two experiments of highest concentration of product present, formation of compound **4** in small concentrations interferes with the experiment due to signal overlap in the ^1^H NMR spectra used to calculate the concentrations. We have corrected data from these two experiments by subtracting the integral of the relevant signal of **4** from the integral of the relevant signal of **2**.

**Scheme 4:**
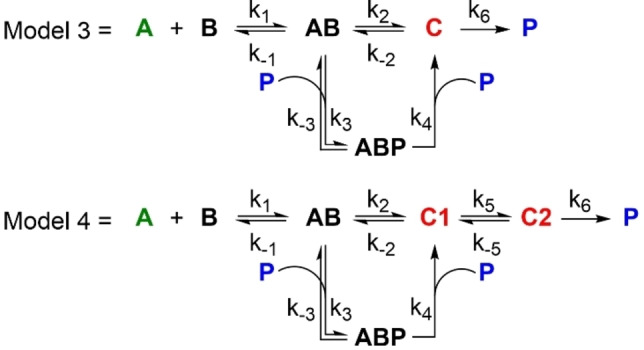
Models 3 and 4 including product autocatalysis and complexation between starting materials **A** and **B** ‐ Used for fits shown in Figure 6.

**Figure 6:**
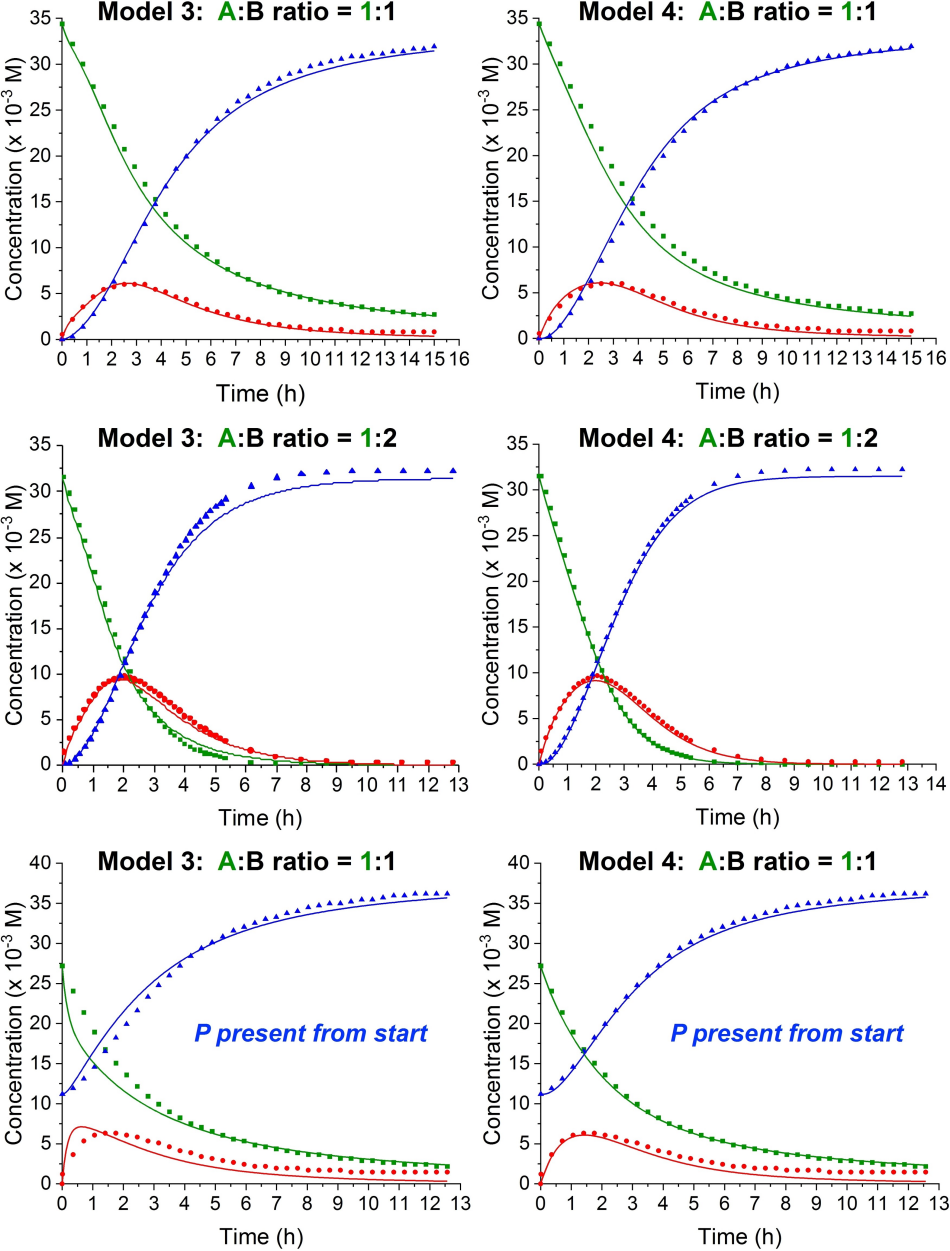
Plots showing the disappearance in time of **1** (also labelled **A**; green square), appearance and disappearance of intermediate **2** (also labelled **C**; red circle), and formation of product **3** (also labelled **P**; blue triangle) over time at two different substrate ratios (**A : B**; **B**=TCNE). The fits using Model 3 (left) and Model 4 (right) were constructed using six different substrate ratios (see Supporting Information, p. S125–132), two of which were with product present at the beginning.

The color changes occurring during reaction indicate both intramolecular (donor‐acceptor chromophore product) and intermolecular charge‐transfer absorptions. Together with the fact that NMR spectroscopy showed that chemical shifts of specific protons in the alkyne substrate, intermediate and products changed during the entire reaction progress, this seems to indicate that we have some associations between the species. This observation stimulated us to perform experiments where the product would be present from the very beginning of the reaction in various concentrations. Somewhat surprisingly, this presence strongly enhanced the build‐up of intermediate as shown in Figure 5. In other words, the reaction seems to be autocatalytic. This result makes us move forward Models 3 and 4 shown in Scheme 4, where associations between substrates (complex **AB**) as well as with product (complex **ABP**) are taken into account, using either a concerted first step or a stepwise formation of the cyclobutene product. These two models were now used to fit the experimental data, and the results are shown in Figure 6. Both models were found to fit the data excellently, but the stepwise Model 4 involving a zwitterionic intermediate (**C1**) seemed to perform slightly but noticeably better. This result is in line with previous suggestions based on studies of other substrates.[^3b^, ^4i^, ^5^] The stepwise mechanism (Model 4) includes a rather fast conversion of **C1** into the cyclobutene intermediate **C2** (with ratio of rate constants k2/k5=20.4), hence being almost concerted. The rate constants at 300 K for all the steps of the two models are listed in Table 1. As association constants (=k_1_/k_−1_) for complex **AB**, we obtain 0.04 M^−1^ (Model 3) and 0.03 M^−1^ (Model 4), and for complex **ABP** (k_3_/k_−3_), we obtain 3.4×10^6^ M^−1^ (Model 3) and 1.2×10^6^ M^−1^ (Model 4). The complex formation between **AB** and **P** thus seems remarkably strong, while the complex formation between **A** and **B** is 2 orders of magnitude smaller than we determined for the complex between aniline and TCNE (which is reasonable considering the stronger donor strength of unsubstituted aniline). The complex **ABP** transforms much faster into intermediate **C** or **C1** (and released **P**) than does complex **AB**: k_4_/k_2_=118 (Model 3); k_4_/k_2_=62 (Model 4).

**Table 1:** Rate constants (k) for steps shown in Scheme 4 (Solvent: C_6_D_6_; Temperature: 300 K).

Rate constant [s^−1^]	Model 3	Model 4
k_1_	0.137	0.250
k_‐1_	3.61	9.21
k_2_	0.0636	0.0961
k_‐2_	7.73×10^−4^	2.65×10^−3^
k_3_	43.4	16.5
k_‐3_	1.29×10^−4^	1.40×10^−4^
k_4_	7.48	5.98
k_5_	–	4.71×10^−3^
k_‐5_	–	4.25×10^−5^
k_6_	2.35×10^−4^	2.54×10^−4^

## The Retro‐electrocyclization Step

**Figure 7:**
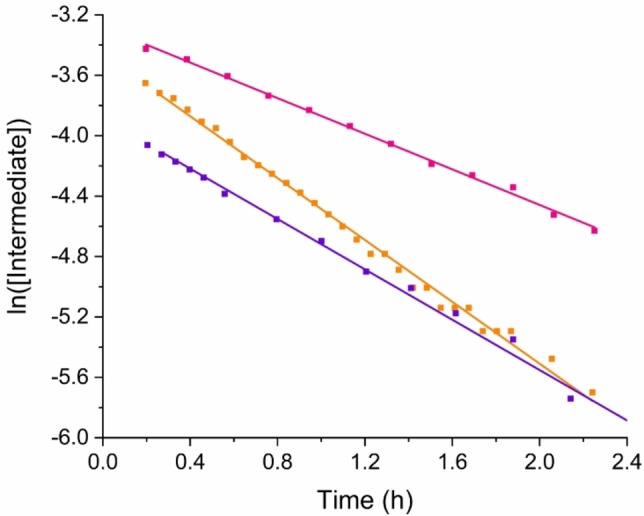
Plots of the logarithm to the concentration of intermediate **2** over time (at 300 K; three experiments, all with 1 : 1 ratio of **1**/TCNE). A straight line was in each case fitted to the data. Orange: solvent=CD_2_Cl_2_ (trend line with slope of −2.8×10^−4^ s^−1^); Purple: solvent=CD_2_Cl_2_ with C_6_D_6_ added after 8 minutes of reaction time (volume increasing from 0.5 mL to 0.75 mL; trend line with slope of −2.3×10^−4^ s^−1^); Pink: solvent=CD_3_COOD (trend line with slope of −1.6×10^−4^ s^−1^).

Using CD_2_Cl_2_ as solvent resulted in intermediate **2** being by far the highest in concentration after 15 minutes of reaction time (see Supporting Information, p. 100). This conveniently enables us to look at the last step of the proposed mechanism, the retroelectrocyclization (*RE*) step. Thus, we decided to study any solvent polarity influence. We initiated two identical experiments in CD_2_Cl_2_ but added C_6_D_6_ to one of the samples after 8 minutes of reaction time (increasing the volume by a factor of 1.5) to reduce the polarity. Figure 7 shows logarithmic plots of the intermediate concentration versus time in neat CD_2_Cl_2_ and in a mixture of CD_2_Cl_2_ and C_6_D_6_. The experimental data were well described by straight lines in both cases, indicating clear first‐order kinetics of the last step. Moreover, the slopes of the trend lines are quite similar in the two cases. These results offer strong evidence for a concerted last step of the reaction, the retro‐electrocyclization, with no polar intermediates involved. From the experiment in CD_2_Cl_2_/C_6_D_6_, we obtain a rate constant of 2.3×10^−4^ s^−1^, and in neat CD_2_Cl_2_ a rate constant of 2.8×10^−4^ s^−1^. These rate constants are close to those obtained from the fits of Models 3 and 4, namely 2.35×10^−4^ s^−1^ (Model 3) and 2.54×10^−4^ s^−1^ (Model 4). Yet, our previously mentioned experiment in pure AcOD‐*d_4_* gave a rate constant of 1.6×10^−4^ s^−1^. It appears that the acid slightly retards the reaction, presumably by protonating the aniline. Thus, the presence of an electron‐donating aryl group, that is a neutral aniline, does seem of some importance for promoting the *RE* step.

## ^13^C NMR Spectrum of the Cyclobutene Intermediate

**Figure 8:**
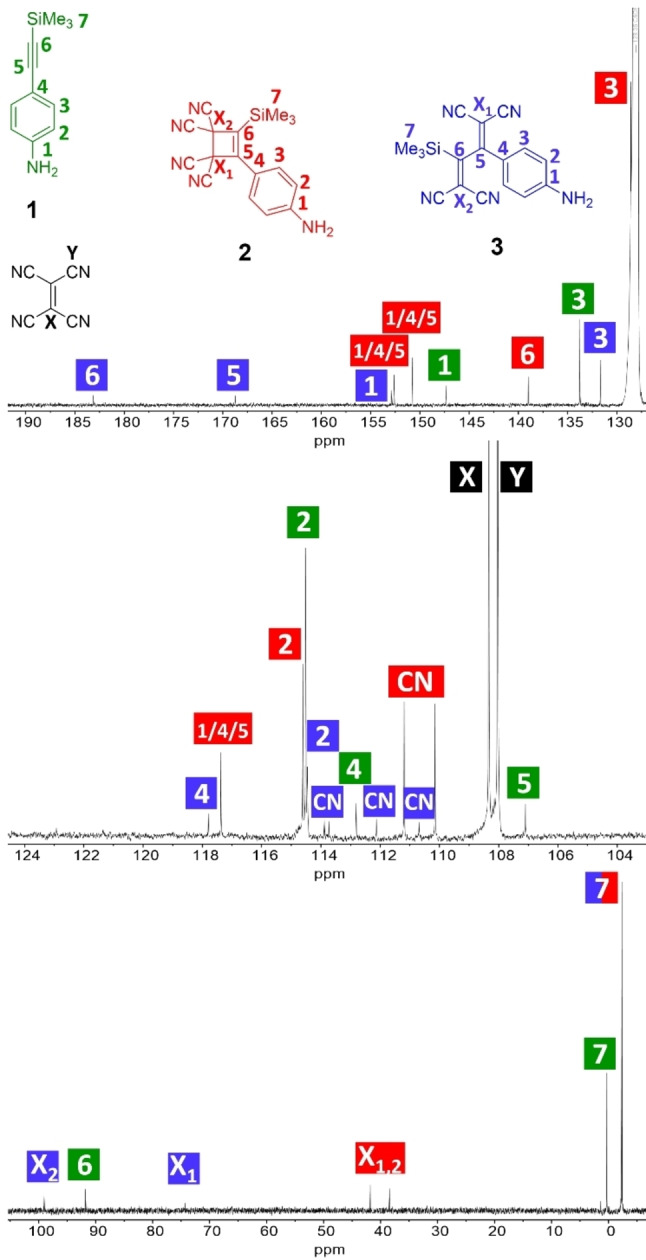
^13^C NMR spectrum (three regions shown; 125 MHz, C_6_D_6_) of a reaction mixture of **1**/TCNE in a ratio of 1 : 5 taken after 1 h of reaction time and assigned signals. The labeling for the intermediate was done with a combination of COSY, HSQC and HMBC and from knowing which signals belonged to starting materials and product.

Our systematic studies allow us to use conditions that can provide additional characterization of the transient cyclobutene intermediate **2** (=**C**=**C2**). Thus, by using a (**1**/TCNE) ratio of 1 : 5, we are able to get the highest possible intermediate concentration and by timing a measurement for ^13^C NMR spectroscopy when the intermediate is at its highest concentration, we managed to obtain a detailed ^13^C NMR spectrum of **2** with the least interfering signals of **1** and **3**. Figure 8 shows regions of the ^13^C NMR spectrum with partial assignments. The chemical shifts are listed in Table 2, and a full spectrum can be seen in Supporting Information, p. S14. We assigned the signals from intermediate **2** with HSQC (Supporting Information, p. S16) and HMBC (Supporting Information, p. S17). Due to the short lifetime of intermediate **2**, the signal to noise ratio was less than desireable but we were still able to assign carbon 6 resonating at 139.00 ppm from the HMBC spectrum and carbon atoms 2 and 3 resonating at 114.61 and 128.60 ppm, respectively, from the HSQC spectrum. For comparison, the sp^2^‐carbon atoms of cyclobutene resonate at 162.3 ppm,^[8]^ while they resonate at 130.57 ppm for a tetracyano‐cyclobutene derivative in which the C=C bond is part of a dibenzo‐fused cyclooctatrienyne ring.^[9]^ Upon ring‐opening of **2**, the signals for these carbon atoms move significantly downfield, 183.13 and 168.74 ppm (C5 and C6 of **3**). The signal at −2.35 ppm of compound **2** we have confidently assigned to the aliphatic carbon 7. The signals at 38.34 and 41.82 ppm are assigned to carbon atoms X_1_ and X_2_ but we cannot say which signal belongs to which carbon. Similarly, the two cyano signals cannot be distinguished. The remaining signals corresponding to carbon atoms 1, 4 and 5 cannot be individually assigned to these three atoms by any of our experiments.

**Table 2:** ^13^C NMR chemical shifts (125 MHz, C_6_D_6_); For assignments, see Figure 8 (X=C(CN), Y=CN.).

Compound	δ(^13^C)/ppm (assignment)
TCNE	108.33 (C−X), 108.03 (C−Y)
**1**	147.61 (C‐1), 133.75 (C‐3), 114.52 (C‐2), 112.81 (C‐4), 107.10 (C‐5), 91.78 (C‐6), 0.30 (C‐7)
**2**	152.61 (C‐1 or C‐4 or C‐5), 150.77 (C‐1 or C‐4 or C‐5), 139.00 (C‐6), 128.60 (C‐3), 117.38 (C‐1 or C‐4 or C‐5), 114.61 (C‐2), 111.19 (C‐Y_1_ or C‐Y_2_), 110.15 (C‐Y_1_ or C‐Y_2_), 41.82 (C‐X_1_ or C‐X_2_), 38.34 (C‐X_1_ or C‐X_2_), −2.35 (C‐7)
**3**	183.13 (C‐6), 168.74 (C‐5), 152.85 (C‐1), 114.45 (C‐2), 131.70 (C‐3), 117.80 (C‐4), 113.88/113.73/112.13/110.69 (C‐Y_a_, C‐Y_b_, C‐Y_c_, or C‐Y_d_), 99.05 (C‐X_2_), 74.32 (C‐X_1_), −2.29 (C‐7)

## ^29^Si NMR spectroscopic Studies

**Figure 9:**
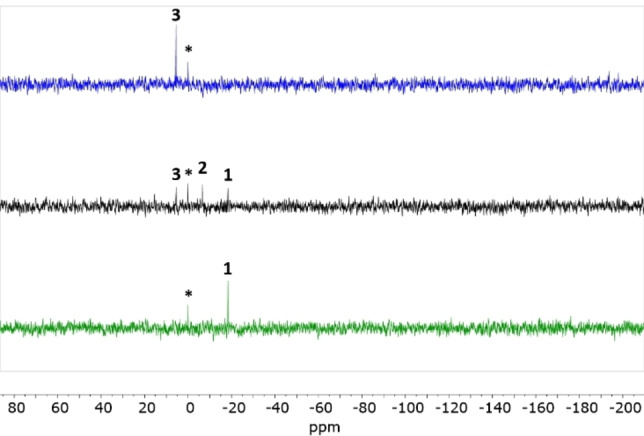
Three selected ^29^Si NMR spectra (99.32 MHz, Standard DEPT sequence observed on ^29^Si with a 7‐Hz ^2^ *J* silicium carbon hydrogen coupling selection) of a reaction mixture of compounds **1**/TCNE in a ratio of 1 : 5 recorded after 0 h (bottom), 80 min (middle) and 12 h (top). The reference compound is tetramethylsilane resonating at 0 ppm, labelled by a *. The signals from the middle spectrum originate from compounds **3**, tetramethylsilane, **2**, and **1**.

As the compounds contain a trimethylsilyl group, we also turned to ^29^Si NMR spectroscopy to follow the conversion. With a (**1**/TCNE) ratio of 1 : 5, a reaction path including three species was observable on the NMR time scale (see Figure 9 and Supporting Information, p. S60 for all ^29^Si NMR spectra). The starting material **1** showed the ^29^Si resonance at −18.37 ppm (green spectrum of Figure 9, referenced relative to tetramethylsilane), the intermediate **2** at −6.59 ppm (black spectrum; reaction mixture), and the product **3** at 5.53 ppm (blue spectrum; complete reaction). The ^29^Si NMR spectroscopic study thus revealed the same patterns as the ^1^H NMR spectroscopic study, with three observable species each behaving as expected, namely one species decreasing in intensity in time, another one appearing and later disappearing, and the last one appearing and persisting.

## IR Spectroscopic Studies

**Figure 10:**
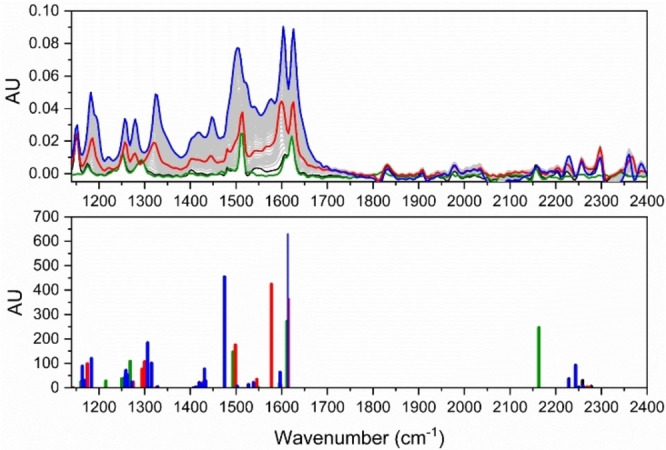
Top: Experimental IR spectra (selected region 1250–2400 cm^−1^) during the reaction progress after mixing substrates **1** and TCNE in a ratio of 1 : 5 (**1**/TCNE) in benzene at concentrations (0.04 M:0.20 M); (start of experiment: green; intermediate at highest concentration: red; end of experiment: blue). Bottom: Calculated vibrational frequencies for **1** (green), TCNE (black), **2** (red), and **3** (blue). AU=arbitrary units.

Finally, the reaction progress was followed using in situ IR spectroscopy (Figure 10). We used a reaction mixture of **1**/TCNE (1 : 5) with concentrations of 0.04 M/0.2 M in benzene. Over the course of 8 h, multiple IR spectra (in the range 3000 cm^−1^ to 650 cm^−1^) of the reaction mixture were recorded.

A computational study was performed for comparison. Thus, the two substrates, the cyclobutene intermediate and the final product were optimized at the B3LYP/6‐31+G(d,p) level using the Gaussian 03 program package.^[10]^ All vibrations were calculated and scaled by 0.9648. The results are shown in Figure 10. Relative good qualitative agreements between experimental and calculated data are observed. The characteristic C≡N stretching vibrations in the region 2200–2300 cm^−1^ are rather weak in both the experimental and calculated spectra. Calculations provided peaks for the C=C of the new cyclobutene ring formed in **2** at 1577, 1598 and 1615 cm^−1^ (with vibrational modes coupled to phenylene and amino units).

## Other Alkyne Substrates

We also attempted the *CA*‐*RE* reaction on alkynes where the *p*‐NH_2_ group of **1** is replaced by either *p*‐NH(CO)CH_3_, *p*‐OMe, *p*‐CN, or *m*‐NH_2_ (details are provided in Supporting Information, p. S5). At room temperature, none of these alkynes reacted with TCNE. This is in line with previous findings^[3b]^ that 1‐ethynyl‐4‐methoxybenzene and 3‐ethynyl‐*N*,*N*‐dimethylaniline require forcing conditions to react, that is, elevated temperatures (70 °C), and that alkynes with electron‐withdrawing substituents are unreactive.

## Conclusion

**Figure 11:**
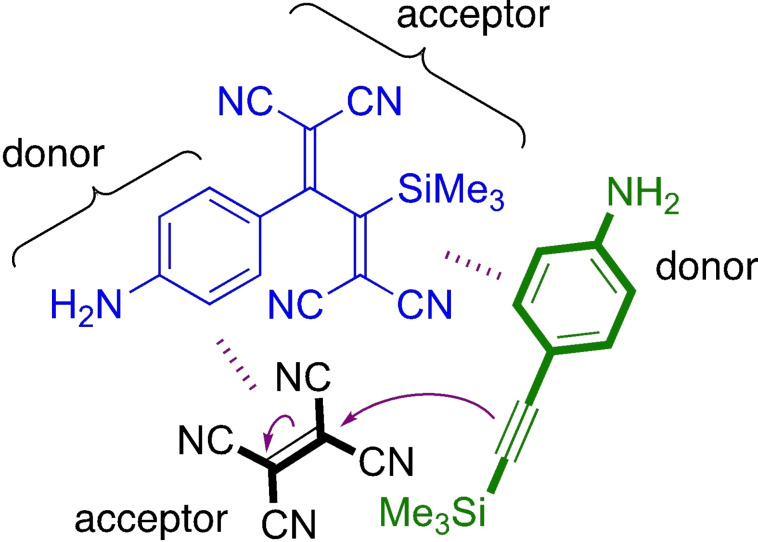
Tentative model for the autocatalysis: via donor‐acceptor complex formations, the final butadiene product is here proposed to assist as a template in organizing the reactants in the right manner for the first step of the cycloaddition (by which zwitterionic intermediate **C1** is formed).

Detailed kinetics studies using ^1^H NMR spectroscopy have revealed that the first and rate‐determining step of the *CA‐RE* reaction cannot be described as a simple second‐order reaction. By fitting various models to a large set of experimental data, we found that the mechanism involves autocatalysis. Thus, the final product of the reaction was found to promote generation of the cyclobutene intermediate, most likely on account of formation of charge‐transfer complexes. It is speculative at the moment how such complex formations actually promote the cycloaddition, but the product may act as a template organizing the reacting species in the right manner as schematically suggested in Figure 11. We hope that our experimental work will stimulate future computational work to shed light on this. It seems most probable that the cycloaddition itself occurs stepwise via a zwitterionic intermediate. Moreover, it was found that the conversion of the cyclobutene intermediate to the final product proceeded as a first‐order reaction in line with a retro‐electrocyclization mechanism. A slight decrease in this first‐order reaction was observed in acetic acid as solvent, which indicates that the aniline‐cyano donor‐acceptor push‐pull effect plays a role for this step. Finally, our systematic studies allowed us to find conditions for maximizing the contents of the cyclobutene intermediate relative to starting materials and product. Thereby, we managed to record a high‐quality ^13^C NMR spectrum of the intermediate and were able to assign several of its signals unequivocally, verifying it as an intermediate in the *CA*‐*RE* reaction. The three species (in fast exchange with complexes) observed by ^1^H NMR spectroscopy were also observed by ^29^Si NMR spectroscopy.

We hope that the autocatalysis moved forward in this work for the *CA*‐*RE* reaction may inspire and lead to future development of simple catalysts for this “click‐like” reaction (or other reactions involving TCNE^[11]^), promoting even further its use for synthesizing elaborate donor‐acceptor chromophores of interest for electronic and optoelectronic devices.

## Experimental Section

**NMR Spectroscopic Studies**: All NMR spectra were recorded on either a 300 MHz or a 500‐MHz instrument. All experiments run in C_6_D_6_ or toluene‐*d*_8_ were conducted by making two stock solutions of the alkyne to be investigated and TCNE, respectively, with a fitting amount of an internal standard (cyclohexane) added to the alkyne solution. Aliquots from each of these solutions, corresponding to the desired substrate ratio, were mixed in an NMR tube right before the sample was subjected to NMR spectroscopy. The experiments were set up with one measured sample followed by a “ghost measurement” that corresponds to the waiting time until the next utilized measurement. This procedure was repeated for as many hours as needed or until no change in concentration of product was observed. From the NMR spectroscopic data, the concentrations of species could then be calculated by using the integral from the internal standard (cyclohexane) as a reference with a known constant concentration by comparing all other integrals towards this reference. A custom‐made kinetics program was used to fit models to the recorded data as described in the Supporting Information.

All NMR experiments run in other solvents than C_6_D_6_ or toluene‐*d*_8_ were performed by adding pure alkyne **1** and TCNE to the NMR tube followed by adding solvent with a reference (cyclohexane). The sample was then measured in the same way as described above and concentrations of species calculated in the same manner.

**IR Spectroscopic Studies**: The IR spectroscopic measurements were performed with a 6.3‐mm AgX fiber conduit with a wetted Au, diamond, C22 IR probe equipped to an in situ IR‐measuring device; IR spectra were recorded at meaningful intervals of 5–10 minutes. The reaction mixture itself was prepared by dissolving **1** (32.8 mg, 0.173 mmol) in benzene (5 mL) in a 10 mL, 2‐necked, pear‐shaped flask. The IR probe was then lowered into the mixture and a single spectrum of compound **1** was measured. The probe was then removed shortly before the next measurement, and TCNE (126.2 mg, 0.985 mmol) was added and the probe inserted again. The reaction mixture was stirred for 30 s, whereafter the first measurement of the reaction was recorded. The reaction mixture would not be stirred for the rest of the experiment.

**UV‐Vis Absorption Spectroscopic Studies**: The UV‐Vis spectroscopic measurements were performed in a 1‐cm path length cuvette. Absorption spectra of 1 : 1 mixtures of aniline and TCNE at different concentrations were measured. The absorbance of the charge‐transfer absorption band at each concentration was used to calculate the association constant.

**Computations – IR Frequencies**: Compounds were optimized at the B3LYP/6‐31+G(d,p) level using the Gaussian 03 program package.^[10]^ All vibrations were calculated and scaled by 0.9648.

## Conflict of interest

The authors declare no conflict of interest.

1

## Supporting information

As a service to our authors and readers, this journal provides supporting information supplied by the authors. Such materials are peer reviewed and may be re‐organized for online delivery, but are not copy‐edited or typeset. Technical support issues arising from supporting information (other than missing files) should be addressed to the authors.

Supporting Information

Supporting Information

Supporting Information

Supporting Information

Supporting Information

Supporting Information

Supporting Information

Supporting Information

Supporting Information

Supporting Information

Supporting Information

## Data Availability

The data that support the findings of this study are available in the supplementary material of this article.
