# Supplementary material for: On the Mechanism of the Formal [2+2] Cycloaddition – Retro‐electrocyclization (CA‐RE) Reaction
Source: Chemistry. 2022 Nov 22;29(3):e202202833. doi: 10.1002/chem.202202833 (PMC10099493; doi:10.1002/chem.202202833)
Supplement: Supplementary file 1 — Supporting Information [file CHEM-29-0-s006.pdf]

Chemistry–A European Journal

Supporting Information

On the Mechanism of the Formal [2+2] Cycloaddition – Retro-electrocyclization (*CA-RE*) Reaction

Jonathan Kirschner Solberg Hansen, Christian G. Tortzen, Preben Graae Sørensen, and Mogens Brøndsted Nielsen*

Supporting Information

Table of Contents

Section 1 – Experimental....S1
Section 2 – Overview of entries where intermediate **C** and product **P** were **not** formed....S5
Section 3 – Overview of entries where intermediate **C** and product **P** were formed....S6
Section 4 – Overview of UV-Vis absorption experiments....S8
Section 5 – Overview of IR spectroscopic measurements....S8
Section 6 – Overview of Video recordings of the *CA-RE* reaction....S8
Section 7 – Spectroscopic data of individual compounds....S9
Section 8 – Spectroscopic data for experiments where intermediate **C** or product **P** were **not** formed....S20
Section 9 – Spectroscopic data for experiments where intermediate **C** or product **P** were formed....S37
Section 10 – Spectroscopic data for UV-Vis experiments....S114
Section 11 – Spectroscopic data for the IR experiment....S116
Section 12 – Description of video recordings of the *CA-RE* reaction....S118
Section 13 – Summary of simulations of experimental data....S119
Section 14 – Example of Kinetic Studies on an unnumbered model....S133

Section 1 – Experimental

Section 1.1 – NMR Spectroscopic Studies

All NMR experiments run in C_6D_6 or toluene-d_8 were conducted by making two stock solutions of the alkyne to be investigated and TCNE, respectively, with a fitting amount of an internal standard (cyclohexane) added to the alkyne solution. Aliquots from each of these solutions, corresponding to the desired substrate ratio, were mixed in an NMR tube right before the sample was subjected to NMR spectroscopy (over the duration of the experiment). The experiments were set up with one measured sample followed by a “ghost measurement” that corresponds to the waiting time until the next utilized measurement. This procedure was repeated for as many hours as needed or until no change in concentration of product was observed.

From the NMR spectroscopic data, the concentrations of species could then be calculated by using the integral from the internal standard (cyclohexane) as a reference with a known constant concentration by comparing all other integrals towards this reference.

All NMR experiments run in other solvents than C_6D_6 or toluene-d_8 were performed by adding pure alkyne **1** and TCNE to the NMR tube followed by adding solvent with a reference (cyclohexane). The sample was then measured in the same way as described above and concentrations of species calculated in the same manner.

All NMR spectra were recorded on either a 300 MHz or a 500-MHz instrument. The 300 Mhz instrument is a Varian 300 Mhz system with a switchable quadro-probe measuring at 298 K. The 500 MHz is a Bruker Avance IIIHD System with a broad band probe measuring at 300 K.

Section 1.2 – IR Spectroscopic Studies

The IR spectroscopic measurements were performed with a 6.3-mm AgX fiber conduit with a wetted Au, diamond, C22 IR probe equipped to an *in-situ* IR-measuring device; IR spectra were recorded at meaningful intervals of 5-10 minutes. The reaction mixture itself was prepared by dissolving 4-[(trimethylsilyl)ethynyl]aniline (**1**) (32.8 mg, 0.173 mmol) in benzene (5 mL) in a 10 mL, 2-necked, pear-shaped flask. The IR probe was then lowered into the mixture and a single spectrum of compound **1** was measured. The probe was then removed shortly before the next measurement and TCNE (126.2 mg, 0.985 mmol) was added and the probe inserted again. The reaction mixture was stirred for 30 s, whereafter the first measurement of the reaction was recorded. The reaction mixture would not be stirred for the rest of the experiment

Section 1.3 – UV-Vis Absorption Spectroscopic Studies

The UV-Vis spectroscopic measurements were performed in a 1-cm path length cuvette, and UV−Vis absorption spectra were obtained by scanning the wavelength from 1000 to 250 nm. The samples measured were prepared by either making a large stock solution and then taking a desired amount of the solution and diluting it and doing the same for every measurement in that experiment or by making a dilution series from a smaller stock solution and measuring after every new dilution.

Section 1.4 – Computational method for estimation of rate constants for a chemical reaction network from multiple experimental measurements

The best methods for estimation of rate constants for chemical reactions are based on the use of analytical solutions for the rate equations of the proposed mechanism by using regression methods to fit the parameters of the analytical solutions to the experimental data. Unfortunately, analytical solutions can only be found for a limited number of very simple mechanisms. Numerical solutions for the rate equations for any homogeneous kinetic model can however be found by numeric integration of the corresponding differential equations, together with a chosen set of rate constants and of the initial concentrations used in the experiments. An estimate of the validity of a chosen model and a set of rate constants can then be calculated as the sum d(sim,exp) = $\sum_i |sim(t_i)-exp(t_i)|$ of the numerical distances between the simulated values $sim(t_i)$ and the experimental values $exp(t_i)$ of the concentations. If several concentrations or sum of concentrations are known from the experiments, d(sim,exp) is calculated as the sum dT over numerical distances between all measured concentrations and/or sum of simulated concentration dT(sim,exp) = $\sum_{i,j} |sim_j(t_i)-exp_j(t_i)|$. In a perfect fit without noise this number is zero. In practice the aim is to search for a mechanism and a set of rate constants for which dT(sim,exp) is as small as possible. To achieve this we have designed a program using the Python language which is computationally effective and easily manageable for the user. The program works as a code generator which from a text describing the chemical model and a list of sets of rate constants, together with a table of experimental data automatically generate the code which when executed simulate the model and find the set of rate constants which minimize dT(sim,exp). An example of the input information supplied by the user is shown below. Our experience with this approach has shown that in order to get reliable results, it is best to fit a model simultaneously to as many experiments as possible performed with different initial conditions and searching for the set of rate constants which are optimal by producing the mininum value of the sum dU(sim,exp) = $\sum_{k,i,j} |sim_{k,j}(t_i)-exp_{k,j}(t_i)|$ over all the different experimental and computed values of the concentrations. As numeric integrator of the kinetic equations, the program uses Cvode from Sundials in BDF mode with a numeric Jacobian. The Cvode program is described in http://robotics.stanford.edu/~scohen/cvode.paper.pdf

The fitting program requires that the model is described by a text displaying the chemical mechanism and proposed values of the rate constants. For a mechanism like A+B <-> C <-> D -> P the input text used in the program may look like

```
1: A + B    <=>    C    ; k> = 0.1; k< = 0.002;
2: C        <=>    D    ; k> = 0.001; k< = 0.0001;
3: D         ->    P    ; k> = 0.01;
```

The parameters k> and k< give the values of forward and reverse rate constants for each reaction. For a multiple search using a grid of rate constants the input text may look like

k1 = 0.04, 0.07, 0.1, 0.13, 0.16;
km1 = 0.008, 0.014, 0.002, 0.0026, 0.0032; k2 = 0.0004, 0.0007, 0.001, 0.0014, 0.0018;
km2 = 0.00004, 0.00007, 0.0001, 0.00014, 0.00018;
k3 = 0.004, 0.007, 0.01, 0.013, 0.016;

1: A + B <=> C ; k> = k1; k< = km1;
2: C <=> D ; k> = k2; k< = km2;
3: D -> P ; k> = k3;

When rate constants are assigned through lists, an integration and comparison with experimental data is done for all combinations of parameter values in the lists. If lists of m different values are used for each of the n parameters, the total number of combinations of rate constants are m^n. In the example above the total number of integrations of the kinetic equations is 5^5 = 3125. On our own office computers these integrations take ~10s. The experimental data are taken from a list which may look like this

time A C+D P
0.0 0.029999 0.000084 0.000113
161.0 0.021644 0.008118 0.000082
1087.0 0.013648 0.007984 0.008278
1431.0 0.012332 0.007173 0.010692
1791.0 0.011365 0.005476 0.012427
1866.0 0.011435 0.005163 0.013506
1960.0 0.011113 0.005078 0.013521
2309.0 0.010095 0.004070 0.015067
2626.0 0.009336 0.003894 0.016355
3179.0 0.008735 0.002954 0.018218
3378.0 0.007852 0.002934 0.018351
3479.0 0.008038 0.002765 0.019277
3933.0 0.007243 0.002316 0.020014
4775.0 0.006412 0.001625 0.021862
4777.0 0.006082 0.001880 0.021820
4813.0 0.006631 0.001649 0.021823
4851.0 0.006955 0.001487 0.022461
5144.0 0.006375 0.001327 0.022600
5218.0 0.006024 0.001564 0.022634
5590.0 0.005884 0.001314 0.022951
5703.0 0.005421 0.000741 0.023122
5927.0 0.005178 0.001027 0.023525
6034.0 0.005306 0.001173 0.023549
6479.0 0.005166 0.001221 0.023746
6540.0 0.005020 0.000577 0.023667
6556.0 0.005259 0.001011 0.024223
6606.0 0.004693 0.001070 0.023877
7063.0 0.004782 0.000563 0.024255

7354.0 0.004501 0.000995 0.024929
7542.0 0.004514 0.000755 0.024835
8534.0 0.003853 0.000645 0.025413
8727.0 0.003703 0.000430 0.025221
9067.0 0.003863 0.000544 0.025949
9441.0 0.003541 0.000513 0.026013
9661.0 0.003615 0.000299 0.025948

Where the first column is time, the second column [A], the third column [C]+[D], and the fourth column [P]. The values in this example of the parameters giving the minimal value of dT(num,exp) are

k1 = 0.1, km1 = 0.002, k2 = 0.001, km2 = 0.001 and k3 = 0.01.

This example is made with computer generated experimental data with added noise so emulate realistic experimental data and the fitting towards the data is given as a continuous line.

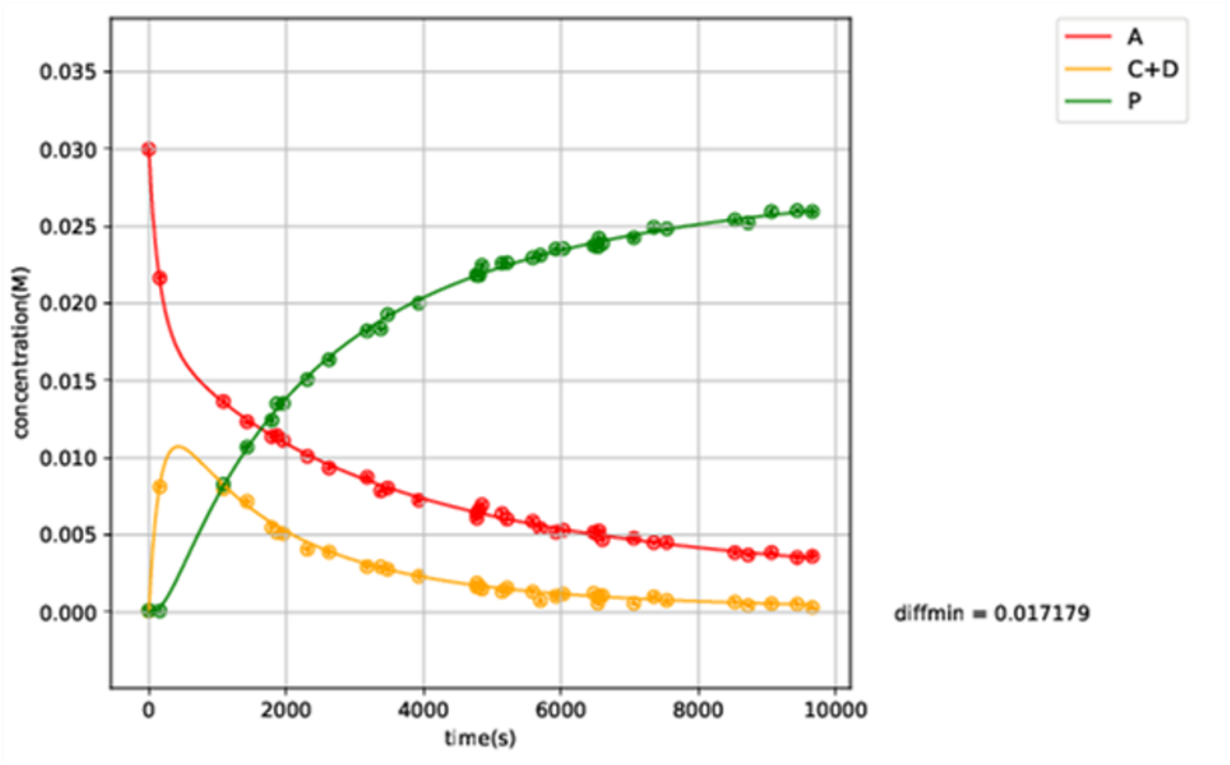

Figure 1.4.1. Computer generated experimental data with added noise to emulate realism as data points and the resulting fit as a continuous line. The value of dT is given by diffmin.

Section 2 – Overview of entries where intermediate **C** and product **P** were **not** formed

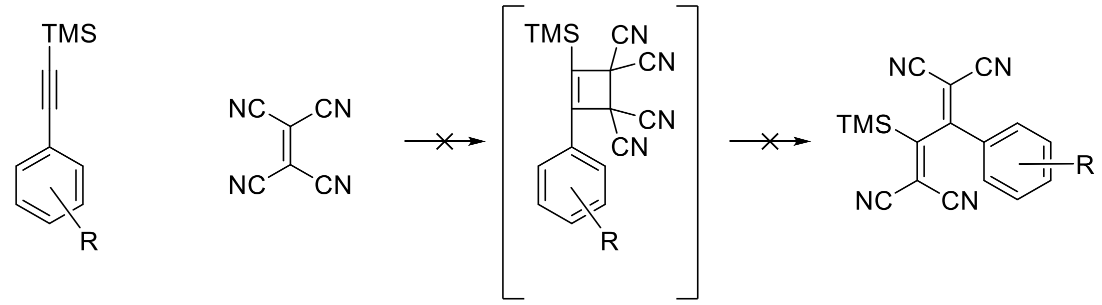

A **B** **C** **P**

In most cases, unreacted starting materials were observed; in a few cases, fast degradation was observed, as indicated below with *.

Entry	R group	Solvent	Additive	Additive amount	Ratio A:B
SI-1	*p*-OMe	C_6D_6	-	-	1:1
SI-2	*p*-OMe	C_6D_6	NH_4OAc	Spatula tip	1:1
SI-3	*p*-OMe	C_6D_6	CuI	Spatula tip	1:1
SI-4	*p*-OMe	C_6D_6	$ZnCl_2$	Spatula tip	1:1
SI-5	*p*-OMe	C_6D_6	TFA (excess)	-	1:1
SI-6	*p*-CN	C_6D_6	-	-	1:1
SI-7	*p*-CN	C_6D_6	CuI	Spatula tip	1:1
SI-8	*p*-CN	C_6D_6	$AlCl_3$	Spatula tip	1:1
SI-9	*p*-CN	C_6D_6	$ZnCl_2$	Spatula tip	1:1
SI-10	*p*-CN	C_6D_6	NH_4OAc	Spatula tip	1:1
SI-11	*p*-NH-Ac	C_6D_6	-	-	1:1
SI-12	*m*-NH_2	C_6D_6	-	-	1:1
SI-13	*p*-NH_2	C_6D_6	DBU*	-	1:1
SI-14	*p*-NH_2	C_6D_6	Et_3N*	-	1:1
SI-15	*p*-CN	CD_3COOD	-	-	1:1
SI-16	*p*-OMe	CD_3COOD	-	-	1:1

*Reaction mixture turned black and sticky.

Section 3 – Overview of entries where intermediate **C** and product **P** were formed *(in some cases, **P** was protodesilylated)*

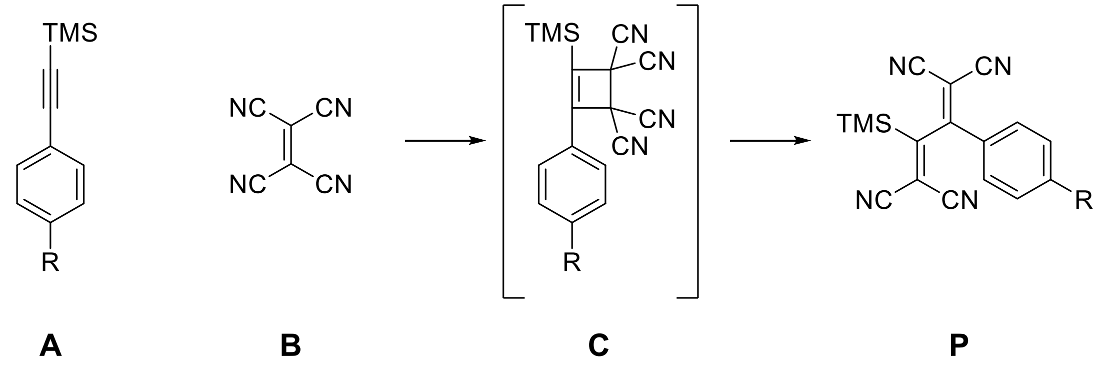

For the kinetics corresponding to the entries, we refer the reader to figures in the manuscript or in SI below.

Entry	R group	Solvent	Additive	Proto-desilylation	Ratio A:B	Measured Nucleus
SI-17	*p*-NH_2	$CDCl_3$	-	No	1:1	^1H
SI-18	*p*-NH_2	Acetone-d_6	-	Yes	1:1	^1H
SI-19	*p*-NH_2	CD_3CN	-	Yes	1:1	^1H
SI-20	*p*-NH_2	THF-d_8	-	Yes	1:1	^1H
SI-21	*p*-NH_2	Toluene	-	No	1:1	^1H
SI-22	*p*-NH_2	Toluene	-	No	1:5	^1H
SI-23	*p*-NH_2	C_6D_6	-	No	1:1	^1H
SI-23X	*p*-NH_2	C_6D_6	-	No	1:1	^1H
SI-23Y	*p*-NH_2	C_6D_6	-	No	1:1	^1H
SI-24	*p*-NH_2	C_6D_6	-	No	1:2	^1H
SI-25	*p*-NH_2	C_6D_6	-	No	1:5	^1H
SI-26	*p*-NH_2	C_6D_6	-	No	1:5	^{29}Si
SI-27	*p*-NH_2	C_6D_6	H_2O	Yes	1:1	^1H
SI-28	*p*-NH_2	C_6D_6	H_2O	Yes	1:2	^1H
SI-29	*p*-NH_2	C_6D_6	H_2O	Yes	2:1	^1H
SI-30	*p*-NH_2	C_6D_6 (dry)	-	No	1:1	^1H
SI-31	*p*-NH_2	C_6D_6 (dry)	-	No	1:2	^1H
SI-32	*p*-NH_2	C_6D_6 (dry)	-	Yes	2:1	^1H
SI-33	*p*-NH_2	C_6D_6	TFA (1 equiv)	Yes	1:1	^1H
SI-34	*p*-NH_2	C_6D_6	TFA (0.5 equiv)	Yes	1:1	^1H
SI-35	*p*-NH_2	C_6D_6	TFA (excess)	Yes	1:1	^1H

SI-36	*p*-NH_2	C_6D_6	AcOH (0.25 equiv)	No	1:1	^1H
SI-37	*p*-NH_2	C_6D_6	AcOH (0.5 equiv)	Yes	1:1	^1H
SI-38	*p*-NH_2	C_6D_6	AcOH (1 equiv)	Yes	1:1	^1H
SI-39	*p*-NH_2	CD_2Cl_2	AcOH (1 equiv)	No	1:1	^1H
SI-40	*p*-NH_2	CD_3COOD	-	No	1:1	^1H
SI-41	*p*-NH_2	C_6D_6	NH_4OAc	Yes	1:1	^1H
SI-42	*p*-NH_2	C_6D_6	Na_2CO_3	No	1:1	^1H
SI-43	*p*-NH_2, *p*-CN	C_6D_6	-	Yes	1:1:1	^1H
SI-44	*p*-NH_2	C_6D_6	Aniline (1 equiv)	No	1:2	^1H
SI-45	*p*-NH_2	CD_2Cl_2	-	No	1:1	^1H
SI-46	*p*-NH_2	CD_2Cl_2	C_6D_6 (after 5 min)	No	1:1	^1H
SI-47	*p*-NH_2	C_6D_6	C_6D_6 (after 24 h)	No	1:1	^1H
SI-48	*p*-NH_2	C_6D_6	-	No	1:5	^1H, COSY, ^{13}C, HSQC
SI-49	*p*-NH_2	C_6D_6	-	No	1:5	^1H, ^{13}C, HMBC
SI-50	*p*-NH_2	C_6D_6	TCNE	No	1:1	^1H
SI-51	*p*-NH_2	C_6D_6	TCNE + **A**	Yes	1:1	^1H
SI-52	*p*-NH_2	C_6D_6	**P** (0.2 equiv)	No	1:1	^1H
SI-53	*p*-NH_2	C_6D_6	**P** (0.4 equiv)	No	1:1	^1H
SI-54	*p*-NH_2	C_6D_6	**P** (1 equiv)	Yes	1:1	^1H

Section 4 – Overview of UV-Vis absorption experiments

Entry	Components measured	Solvent	Ratio A:B	Comment
SI-UV	Aniline/TCNE	C_6D_6	1:1	Measured at different concentrations

The charge-transfer absorption between aniline itself (and not an ethynylaniline) and TCNE was here studied; to avoid parallel changes of the UV-Vis absorption spectrum on account of the *CA-RE* reaction that would take place for an ethynylaniline.

Section 5 – Overview of IR spectroscopic measurements

Entry	Components measured	Solvent	Ratio A:B	Comment
SI-IR	**A** (R = *p*-NH_2):**B**	C_6D_6	1:5	Qualitative measurement to compare to calculated IR frequencies. Measurement was repeated multiple times to test reproducibility

Section 6 – Overview of Video recordings of the *CA-RE* reaction

Entry	Components measured	Solvent	Ratio A:B	Comment
SI-VR1	**A** (R = *p*-NH_2):**B**	C_6D_6	1:1	Concentration of each species is $3.7*10^{-2}$ M
SI-VR2	**A** (R = *p*-NH_2):**B**	CD_2Cl_2	1:1	Concentration of each species is $3.6*10^{-2}$ M

Section 7 – Spectroscopic data of individual compounds

This section covers measured spectroscopy data for each compound below.

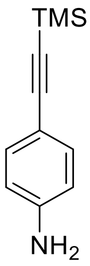

A

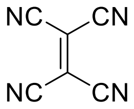

B

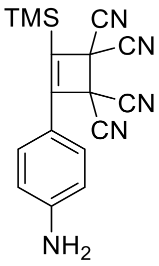

C

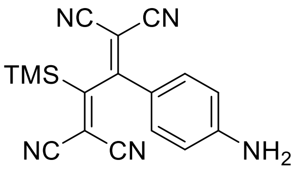

P

Section 7.1 – Spectroscopic data of A (4-[(trimethylsilyl)ethynyl]aniline)

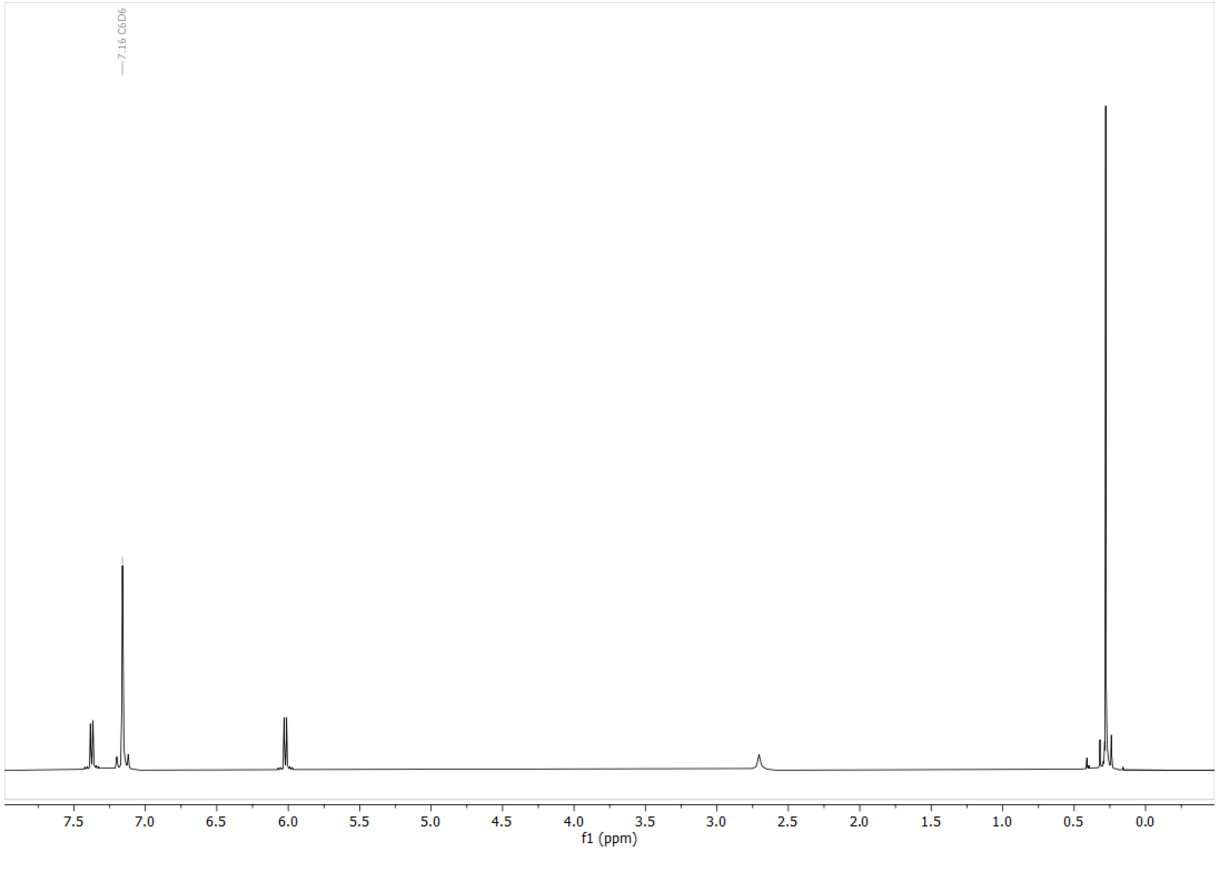

Figure S7.1.1. ^{1}H-NMR spectrum (500 MHz, C_6D_6) of compound **A**.

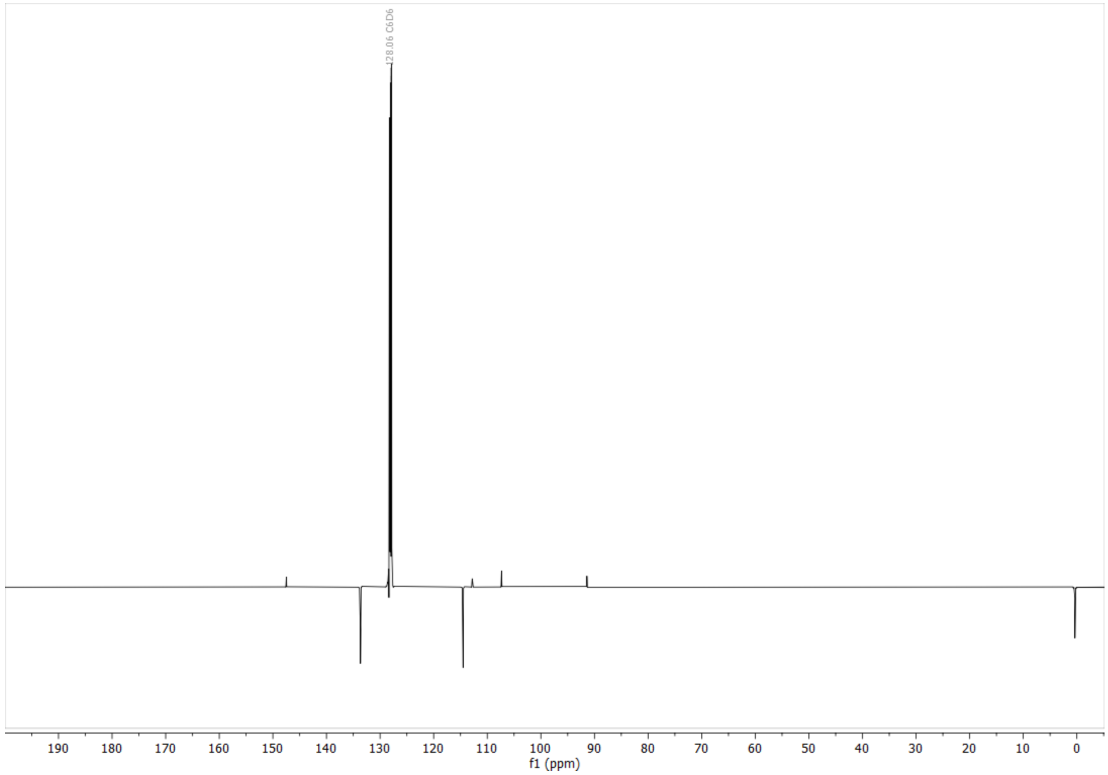

Figure S7.1.2. APT ^{13}C-NMR spectrum (126 MHz, C_6D_6) of compound **A**.

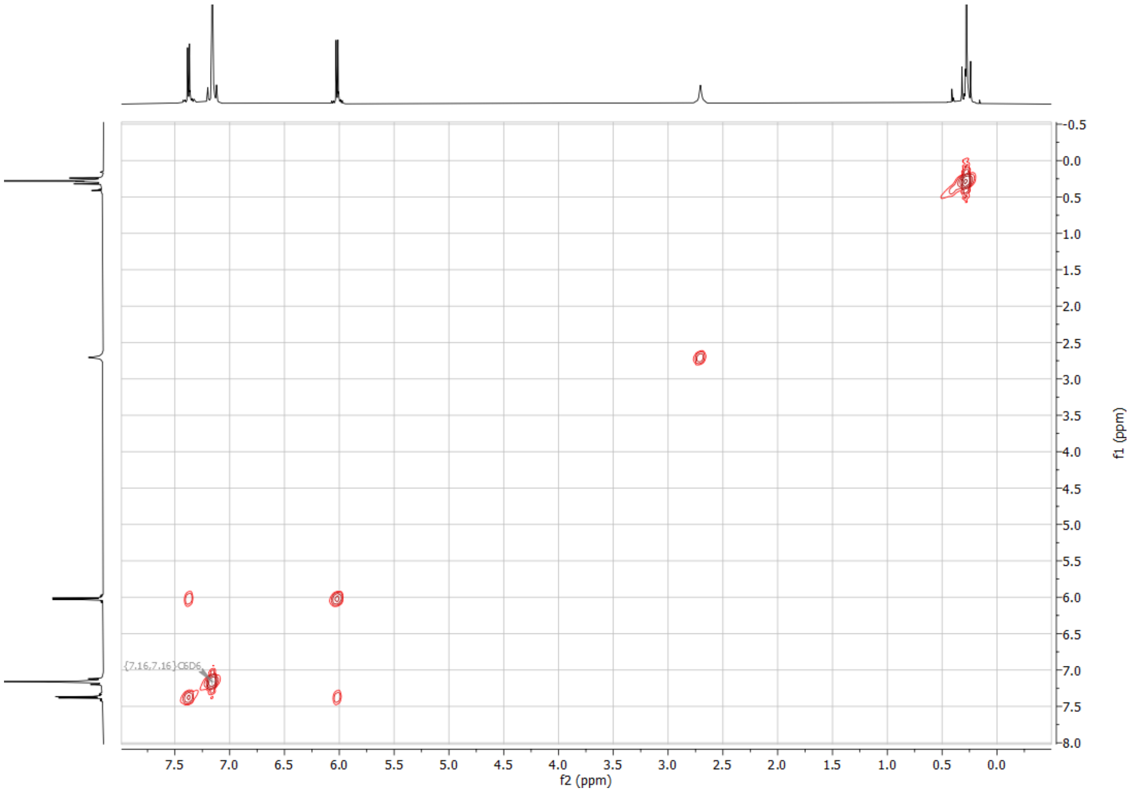

Figure S7.1.3. COSY spectrum (126 MHz, C_6D_6) of compound **A**.

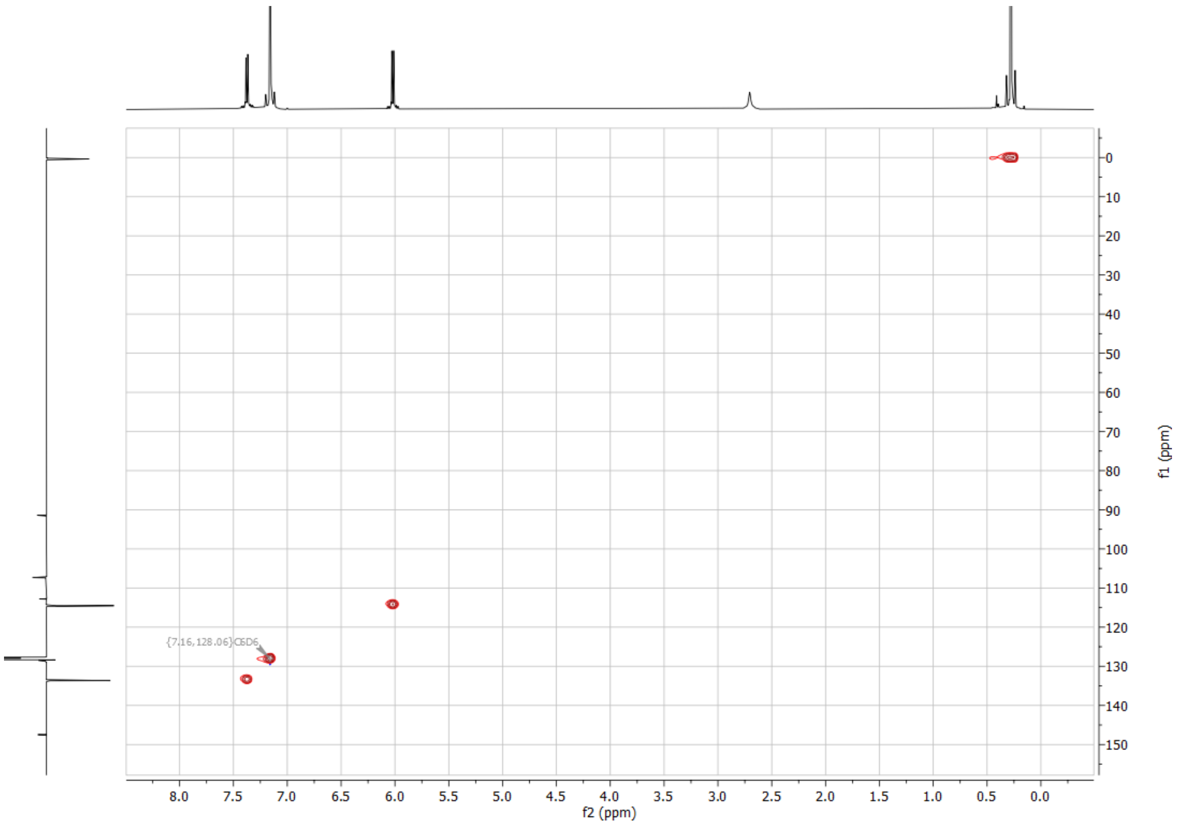

Figure S7.1.4. HSQC spectrum (C_6D_6) of compound **A**.

Section 7.2 – Spectroscopic data of **B** (TCNE)

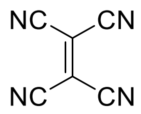

B

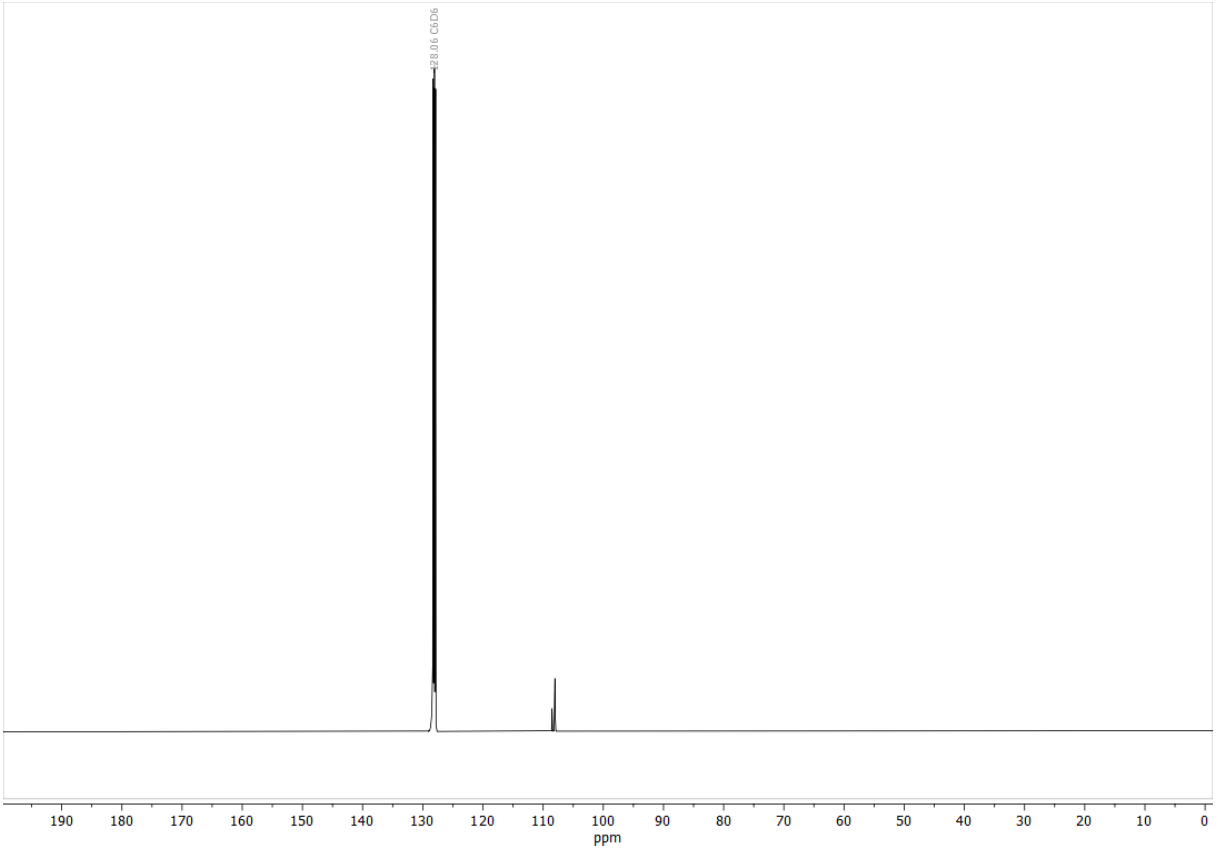

Figure S7.2.1. ^{13}C-NMR spectrum (126 MHz, C_6D_6) of compound **B**.

Section 7.3 – Spectroscopic data of **C**

This compound is not isolated, and the recorded spectra therefore contain a mixture of **A**, **B**, and **C** with **C** being the major component for the chosen spectra below. For assignment of signals, see inside article.

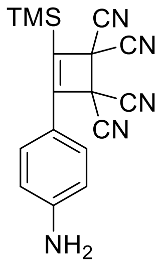

C

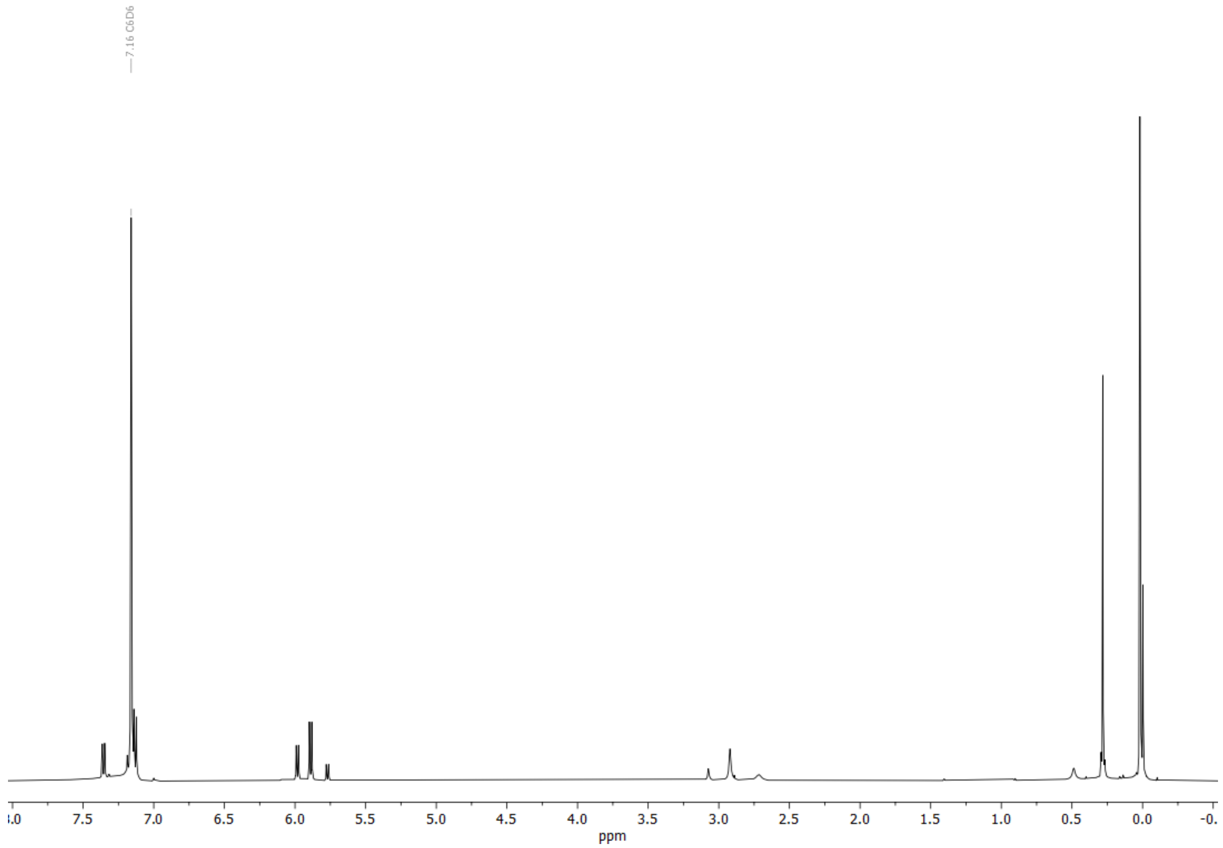

Figure S7.3.1. ^{1}H-NMR spectrum (500 MHz, C_6D_6) of **A**, **B** and **C** with compound **C** being the major component.

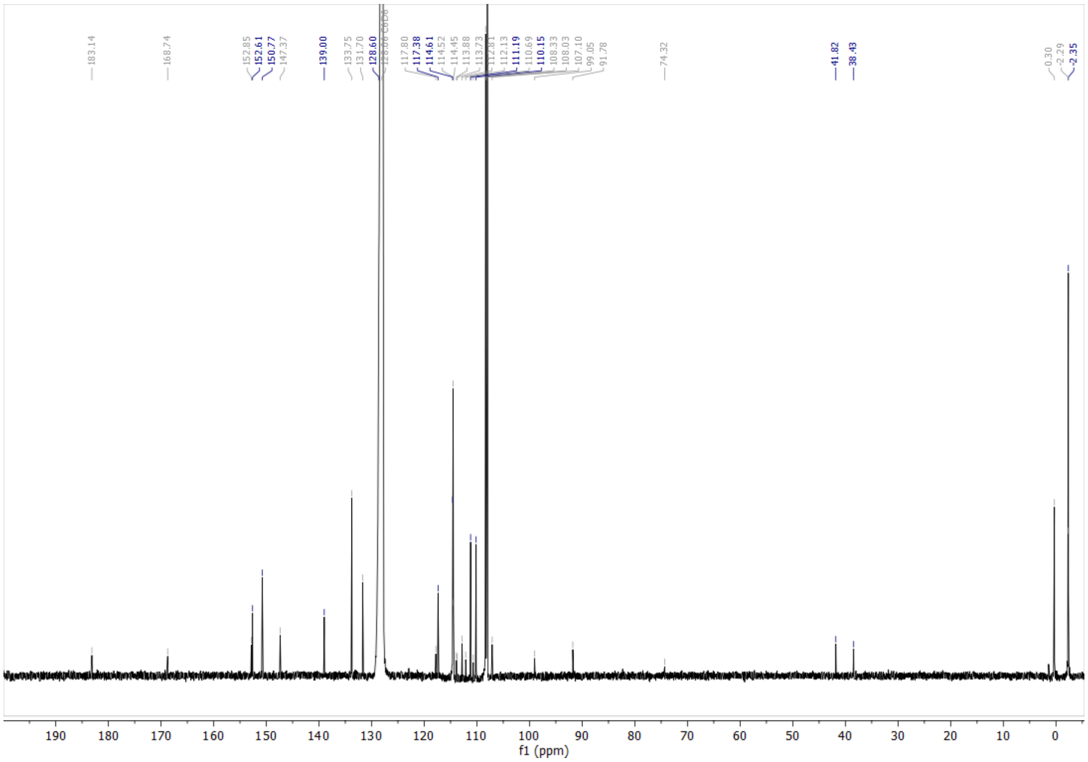

Figure S7.3.2. ^{13}C-NMR spectrum (126 MHz, C_6D_6) of **A**, **B** and **C** with compound **C** being the major component. Peaks from compound **C** are shown in blue.

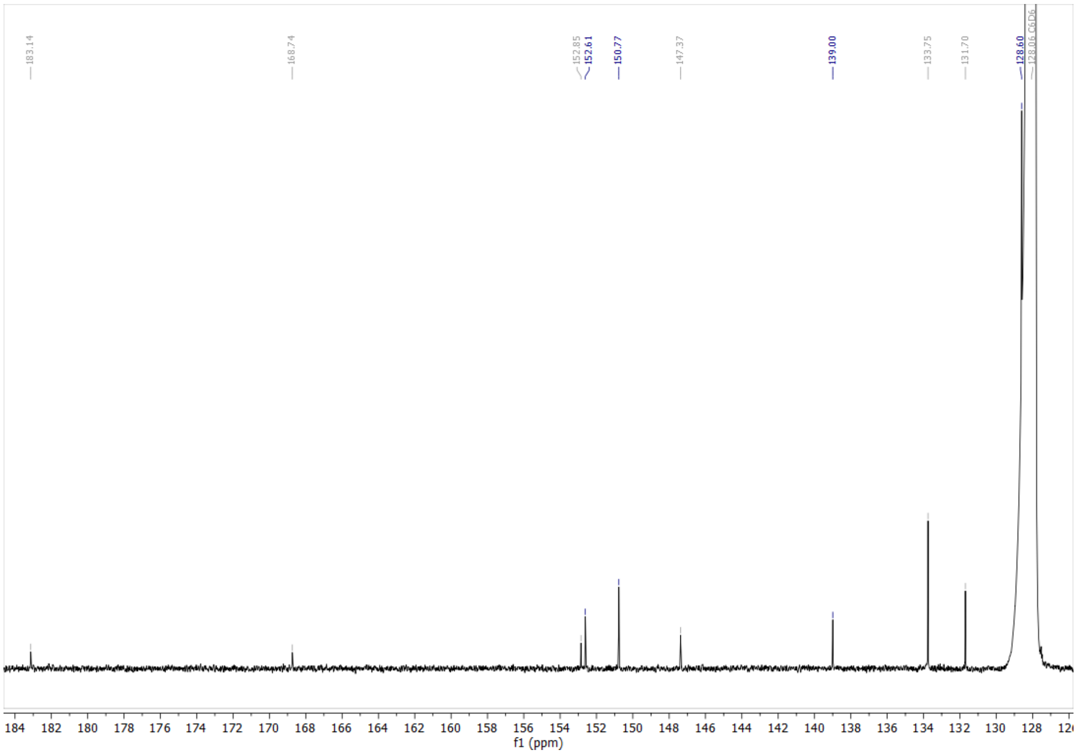

Figure S7.3.3. Selected region of a ^{13}C-NMR spectrum of **A**, **B** and **C** with compound **C** being the major component. Peaks from compound **C** are shown in blue.

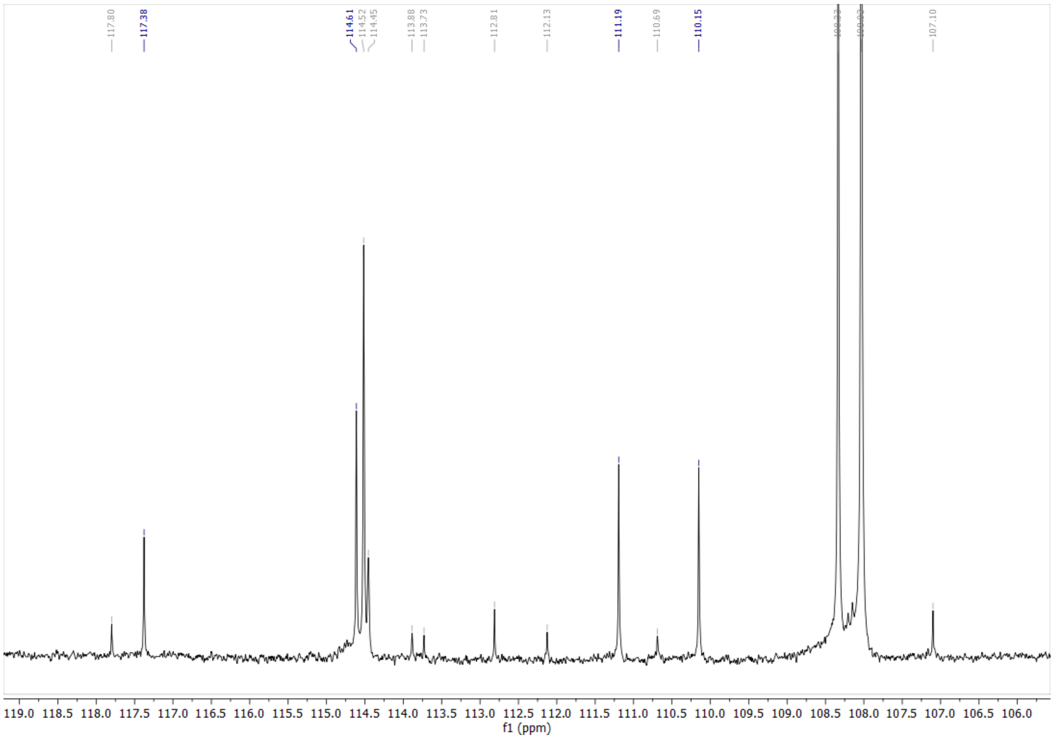

Figure S7.3.4. Selected region of a ^{13}C-NMR spectrum of **A**, **B** and **C** with compound **C** being the major component. Peaks from compound **C** are shown in blue.

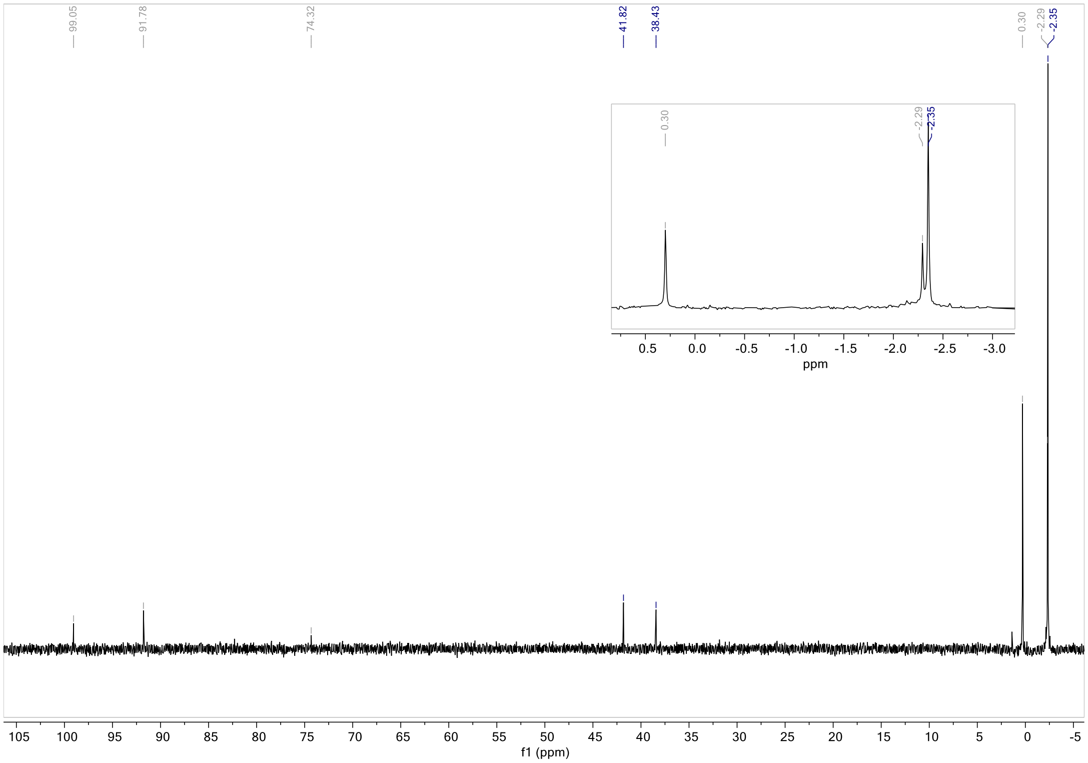

Figure S7.3.5. Selected region of a ^{13}C-NMR spectrum of **A**, **B** and **C** with compound **C** being the major component. Peaks from compound **C** are shown in blue.

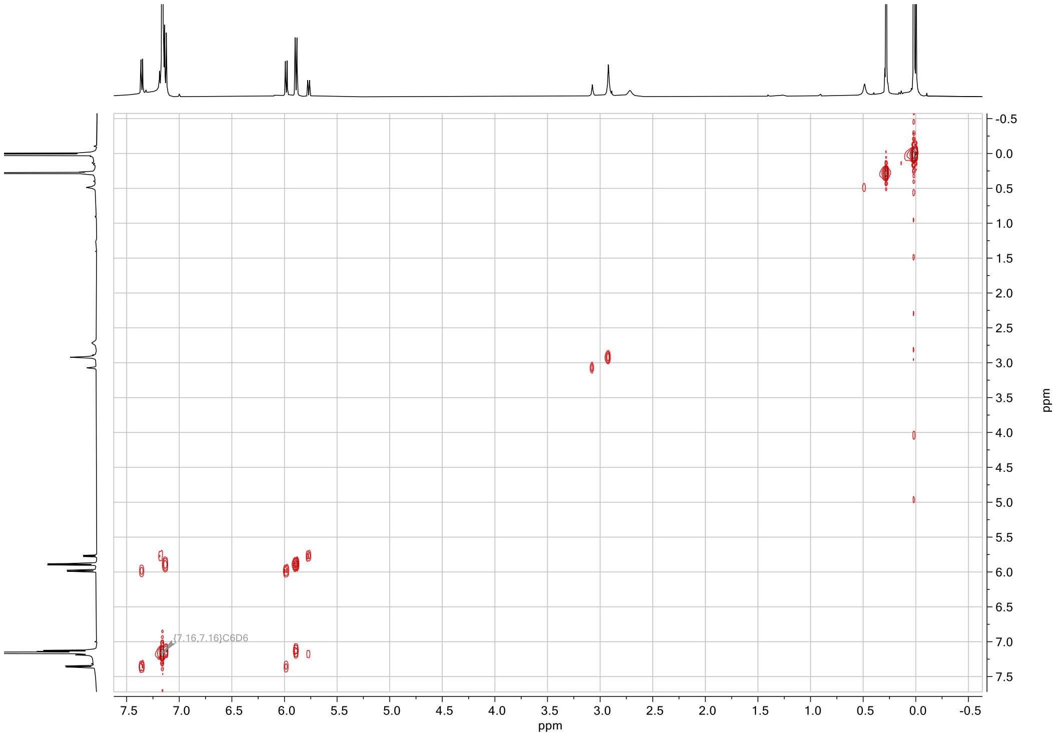

Figure S7.3.6. COSY spectrum (500 MHz, C_6D_6) of a mixture of species **A**, **B** and **C** with compound **C** being the major component.

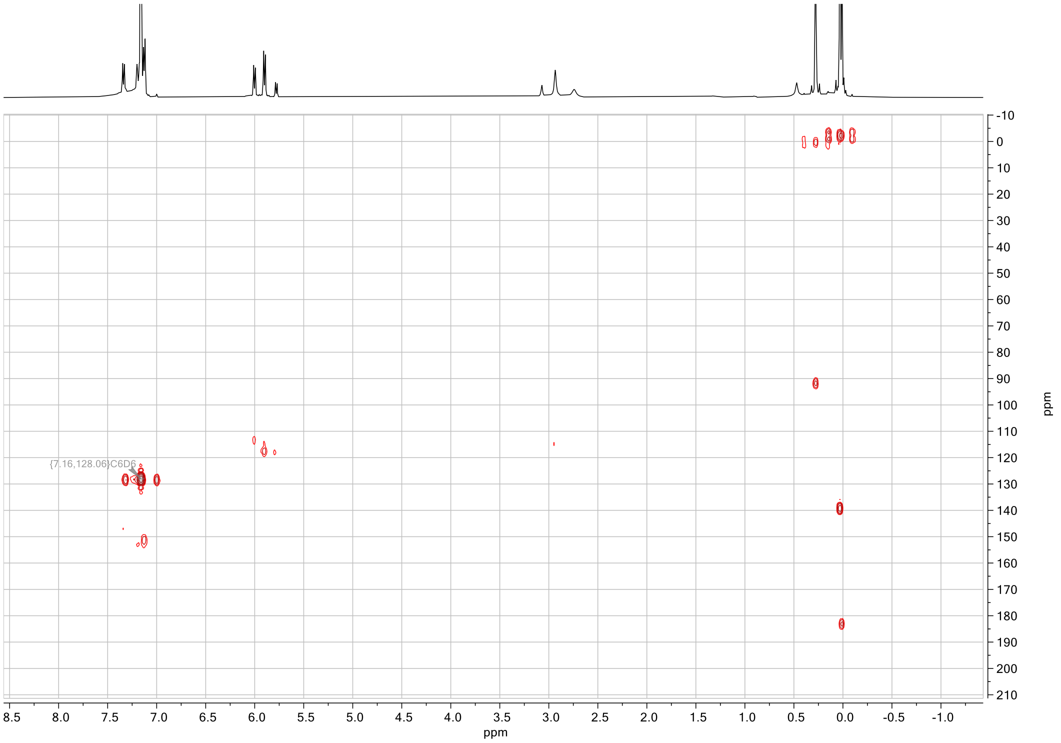

Figure S7.3.6. HMBC spectrum (500 MHz, C_6D_6) of a mixture of species **A**, **B** and **C** with compound **C** being the major component. This was the best achievable signal to noise ratio we could record due to the lifetime of the intermediate.

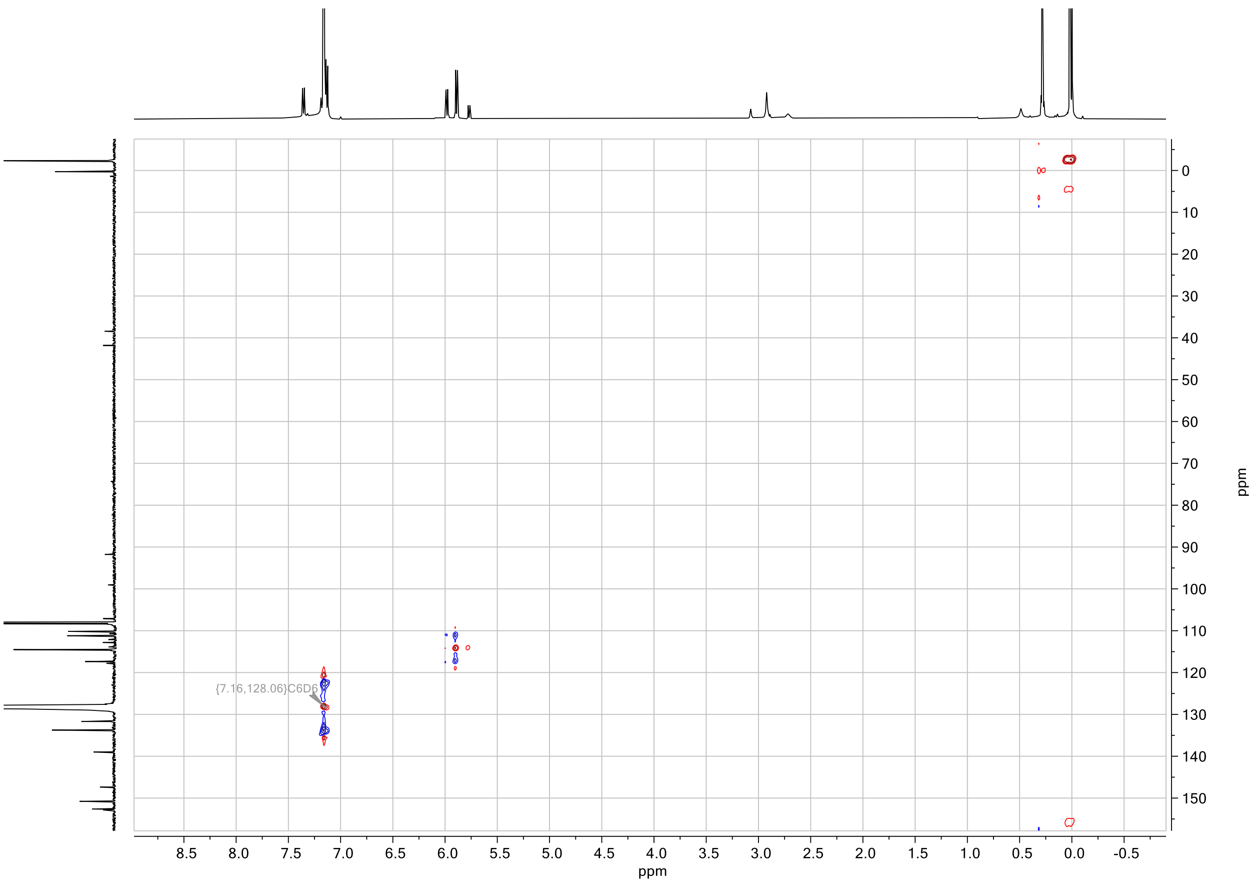

Figure S7.3.6. HSQC spectrum (500 MHz, C_6D_6) of a mixture of species **A**, **B** and **C** with compound **C** being the major component. Due to the reaction running while the spectrum was recorded the environment changes significantly enough to give rise to the unconventional phasing of the signals but this was the best achievable signal to noise ratio we could record due to the lifetime of the intermediate.

Section 7.4 – Spectroscopic data of P

Spectroscopic data for compound **P** were measured after full conversion of **A** and **B** in the NMR tube originally containing **A** and **B**. It was difficult to isolate **P** without protodesilylation occurring.

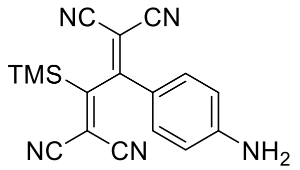

P

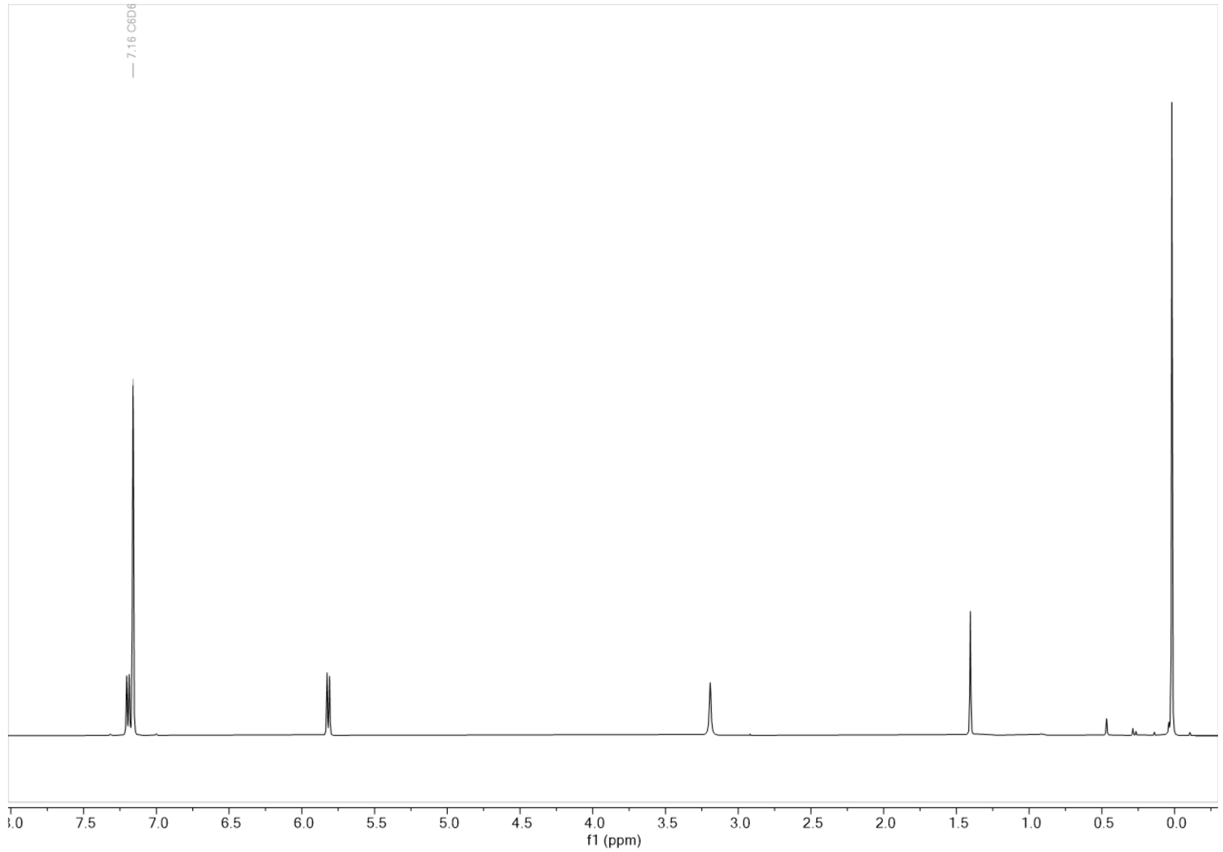

Figure S7.4.1. ^{1}H-NMR spectrum (500 MHz, C_6D_6) of compound **P**.

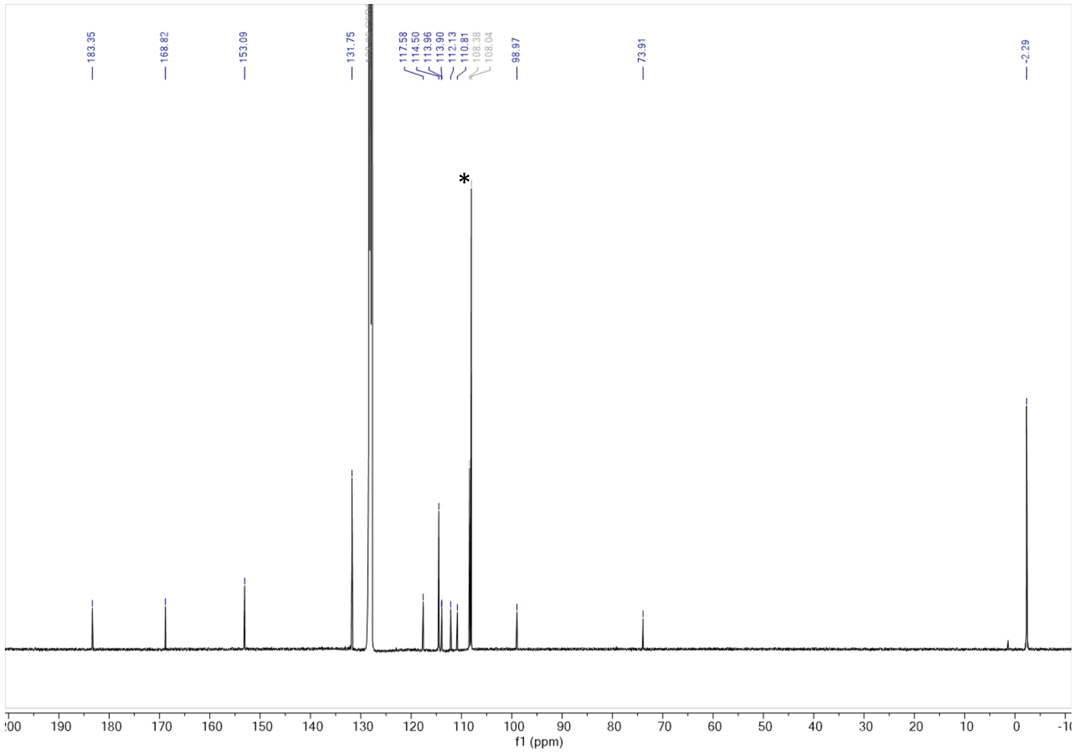

Figure S7.4.2. ^{13}C-NMR spectrum (126 MHz, C_6D_6) of compound **P**. Excess of TCNE from the reaction mixture has been marked with a star (*) and the peak values for TCNE are written in grey.

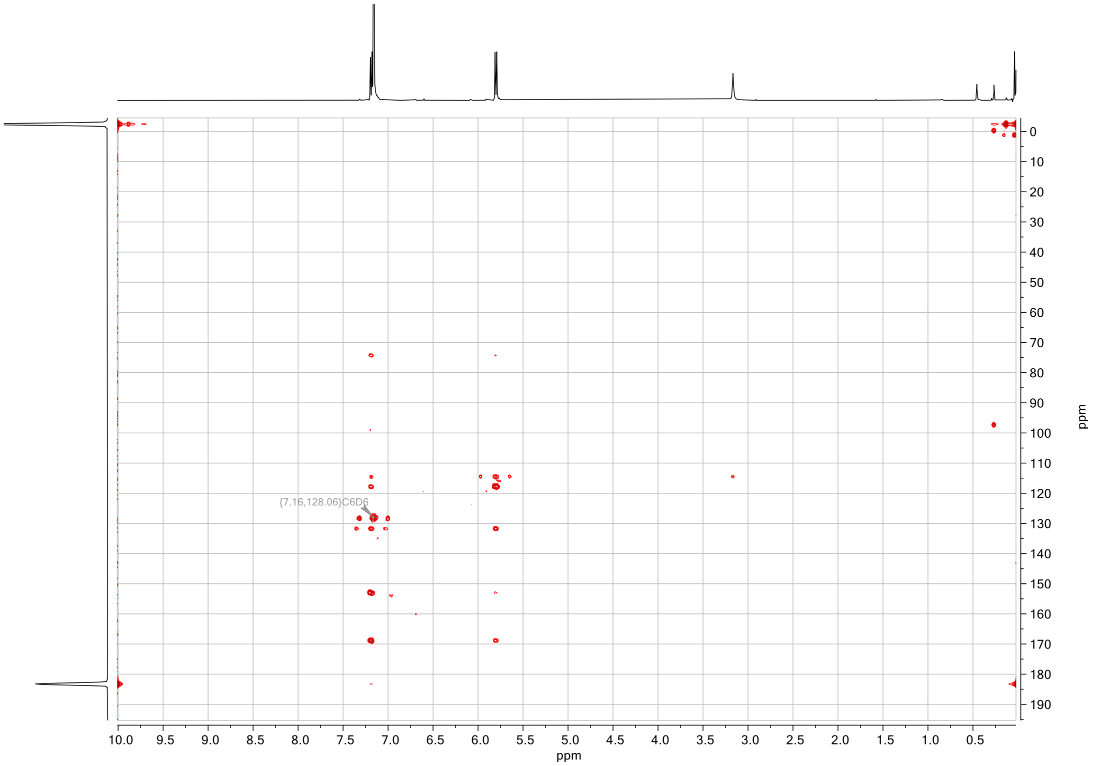

Figure S7.4.3. Detailed HMBC spectrum (500 MHz, C_6D_6) compound **P** with a little leftover of compound **C**.

Section 8 – Spectroscopic data for experiments where intermediate **C** or product **P** were **not** formed

Section 8.1 – Entry SI-1

Reaction between **A** (R = *p*-OMe) and **B** in C_6D_6. The ratio of **A:B** is 1:1.

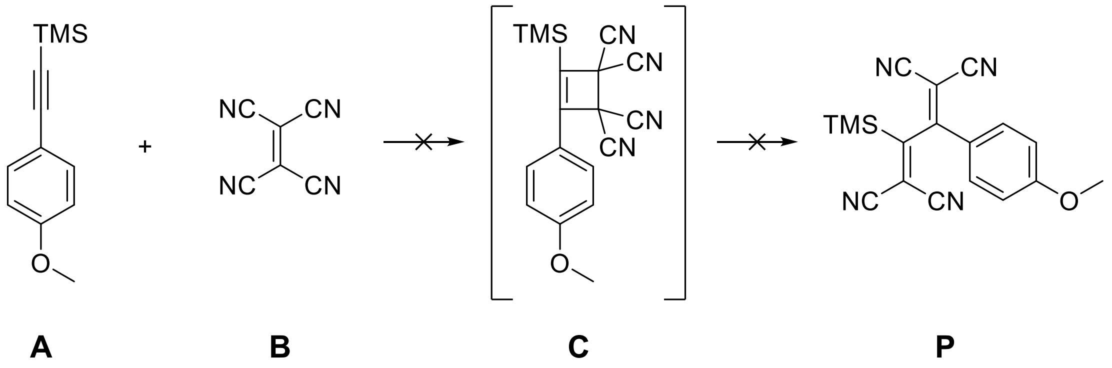

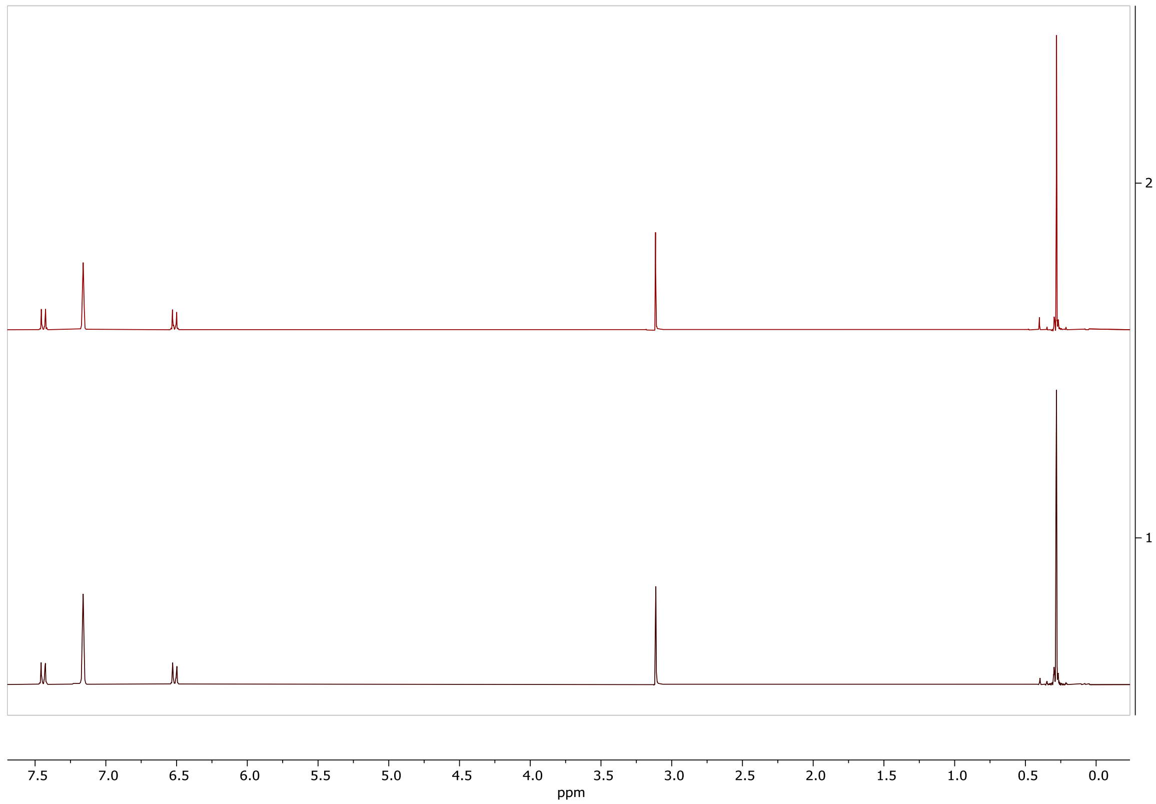

Figure S8.1.1. ^{1}H-NMR spectra (300 MHz, C_6D_6) corresponding to entry SI-1. Bottom spectrum was recorded after 3 minutes of reaction time and top spectrum was recorded after 3 days of reaction time.

Section 8.2 – Entry SI-2

Reaction between **A** (R = *p*-OMe) and **B** in C_6D_6. The ratio of **A:B** is 1:1. A spatula tip of NH_4OAc was added at the start of the reaction.

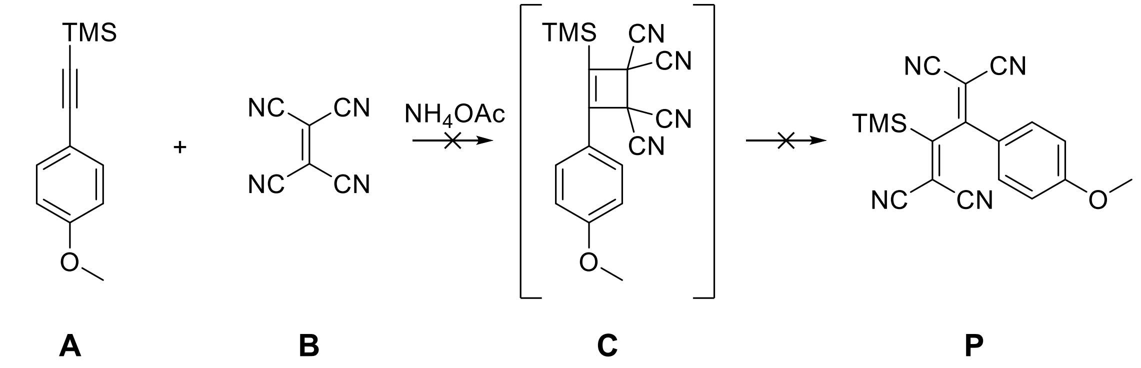

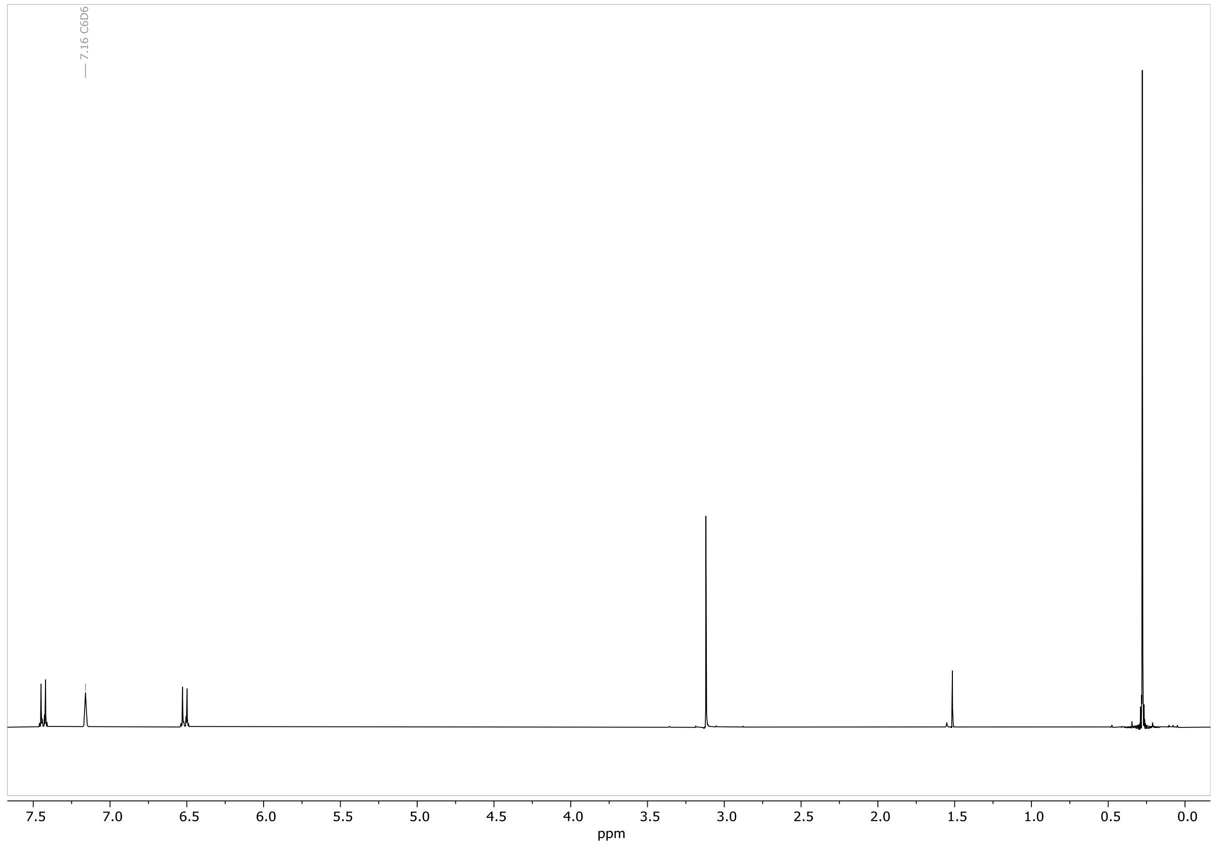

Figure S8.2.1. ^{1}H-NMR spectrum (300 MHz, $CDCl_3$) corresponding to entry SI-2. Spectrum was recorded after 2 hours of reaction time.

Section 8.3 – Entry SI-3

Reaction between **A** (R = *p*-OMe) and **B** in C_6D_6. The ratio of **A:B** is 1:1. A spatula tip of CuI was added at the start of the reaction.

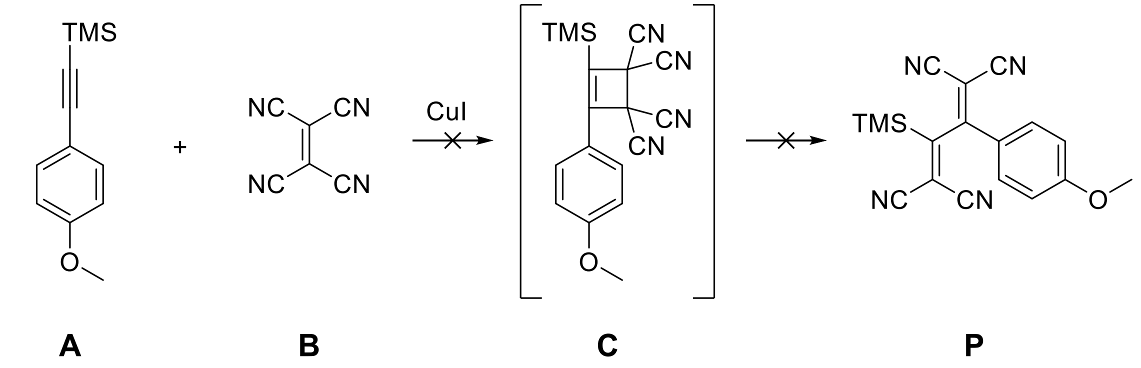

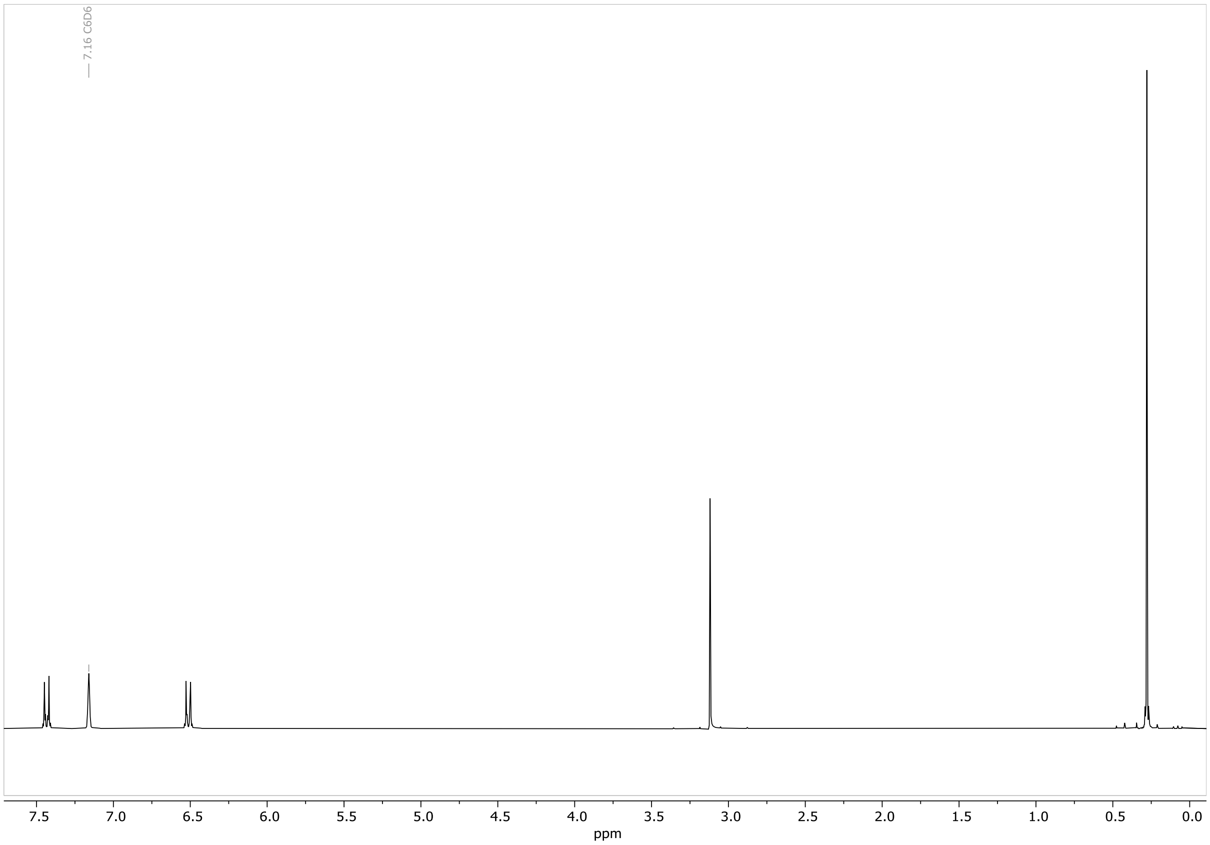

Figure S8.3.1. ^{1}H-NMR spectrum (300 MHz, C_6D_6) corresponding to entry SI-3. Spectrum was recorded after 2 hours of reaction time.

Section 8.4 – Entry SI-4

Reaction between **A** (R = *p*-OMe) and **B** in C_6D_6. The ratio of **A:B** is 1:1. A spatula tip of $ZnCl_2$ was added at the start of the reaction.

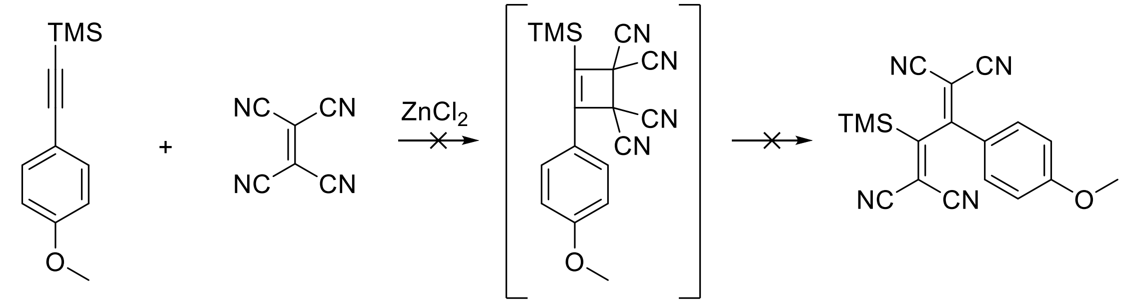

A **B** **C** **P**

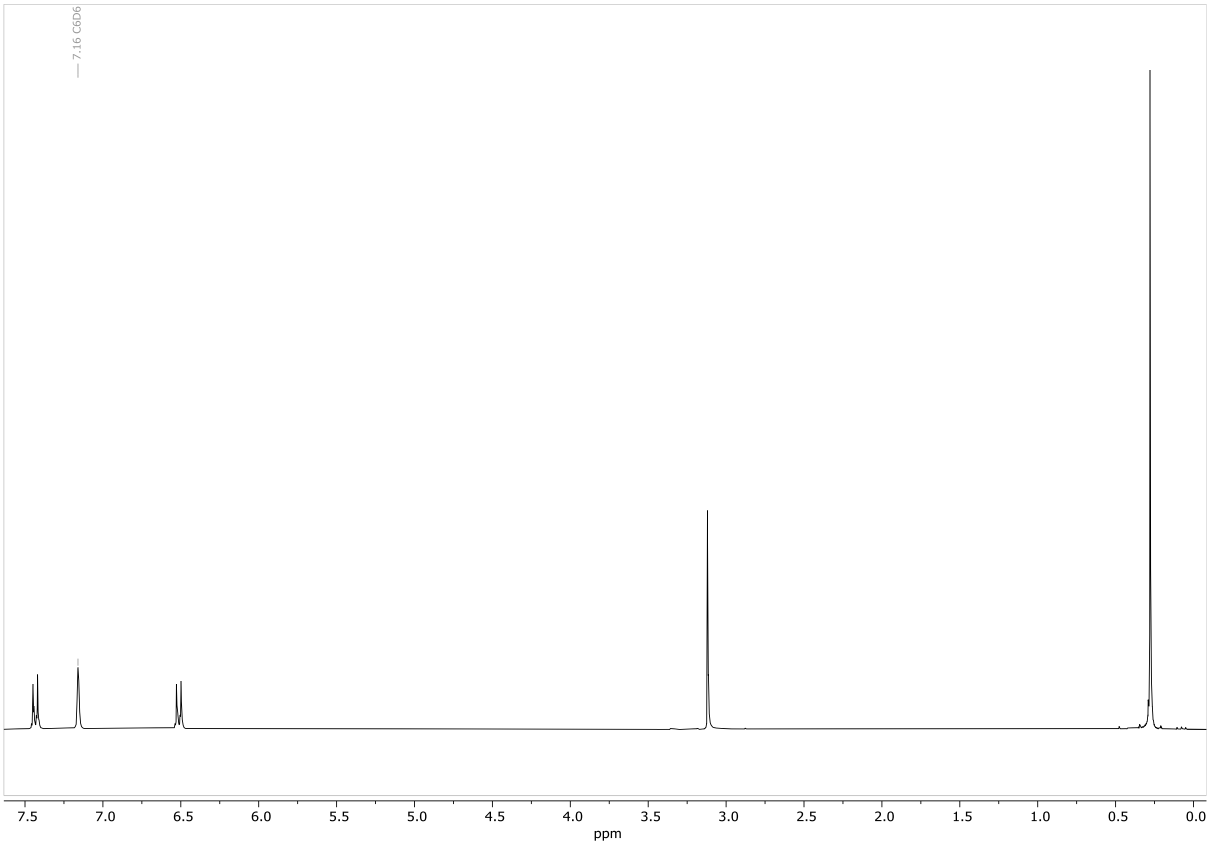

Figure S8.4.1. ^{1}H-NMR spectrum (300 MHz, C_6D_6) corresponding to entry SI-4. Spectrum was recorded after 2 hours of reaction time.

Section 8.5 – Entry SI-5

Reaction between **A** (R = *p*-OMe) and **B** in C_6D_6. The ratio of **A:B** is 1:1. CF_3COOH (TFA) was added in excess at the start of the reaction.

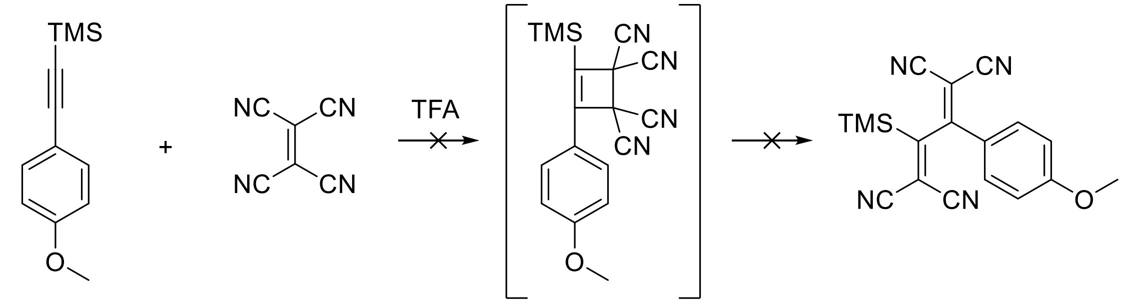

A **B** **C** **P**

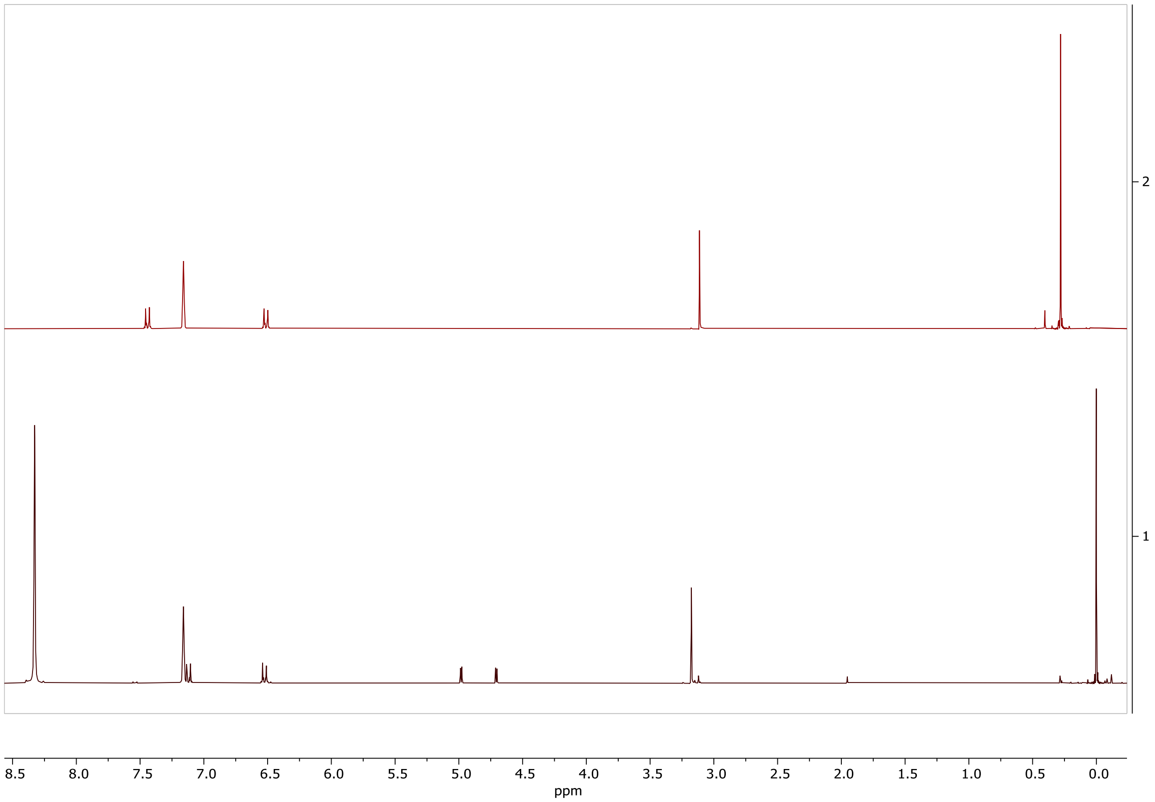

Figure S8.5.1. ^{1}H-NMR spectra (300 MHz, C_6D_6) corresponding to entry SI-5. The spectrum in the bottom was recorded after 10 minutes of reaction time, and the top spectrum was recorded after 3 days of reaction time. The signal at 8.3 ppm correlates to the acidic proton on CF_3COO**H**. The two aromatic signals at 7.2 and 6.5 ppm are shifted relative to the spectrum in absence of TFA as a result of the methoxy group being protonated. The two signals at 5.0 and 4.6 ppm are undetermined. The spectrum after 3 days of reaction time is identical to that of the same experiment without TFA.

Section 8.6 – Entry SI-6

Reaction between **A** (R = *p*-CN) and **B** in C_6D_6. The ratio of **A:B** is 1:1.

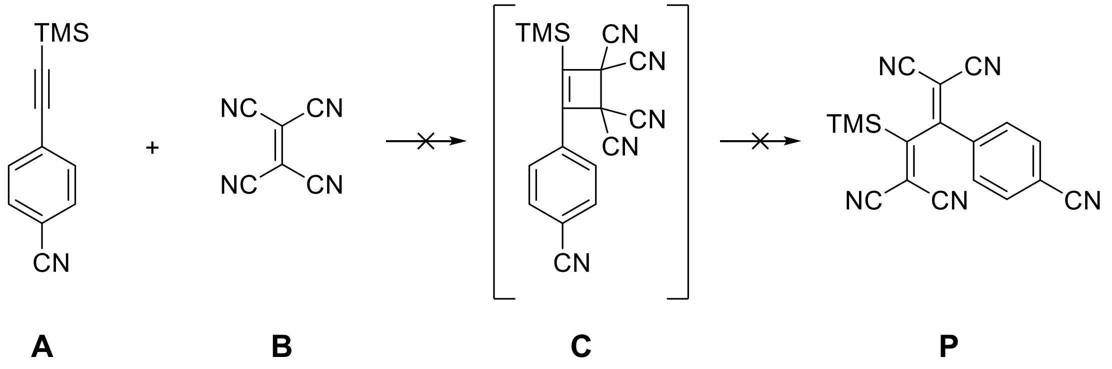

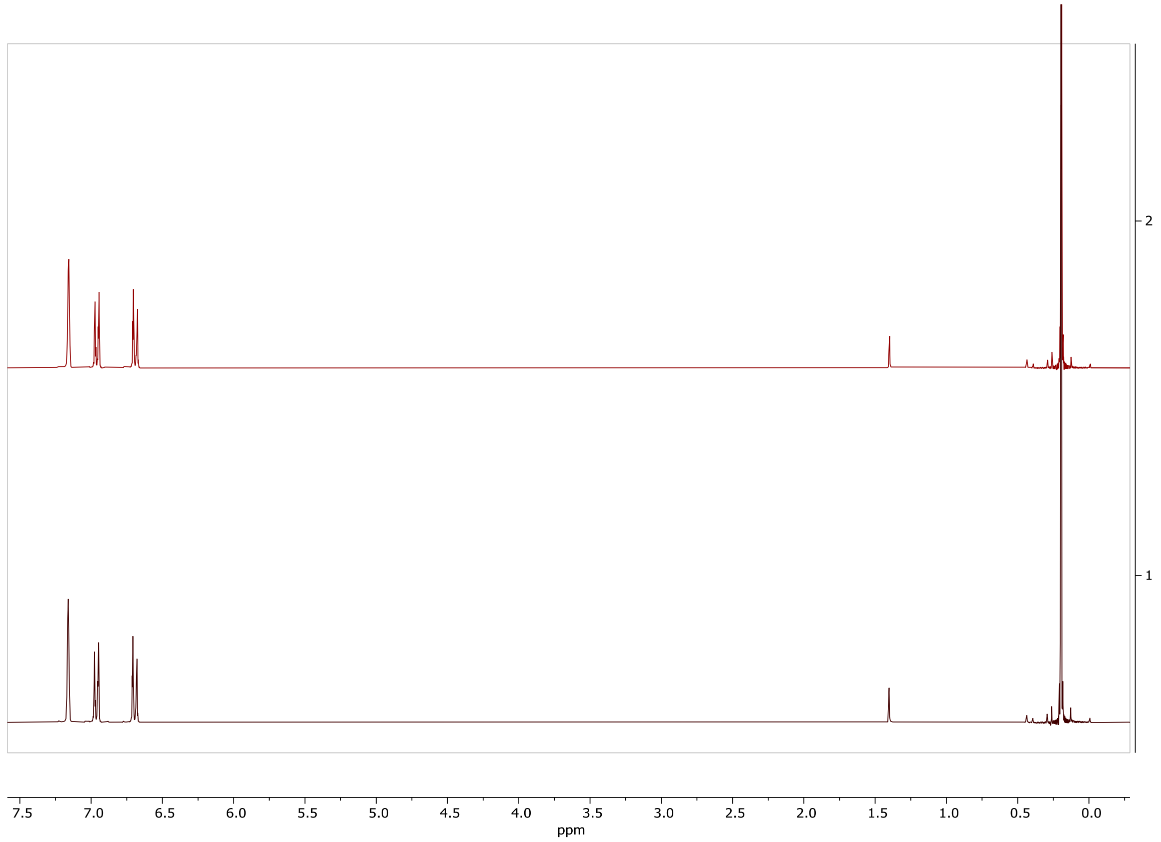

Figure S8.6.1. ^{1}H-NMR spectra (300 MHz, C_6D_6) corresponding to entry SI-6. The spectrum in the bottom was recorded immediately after reaction start, and the top spectrum was recorded after 1 hour of reaction time.

Section 8.7 – Entry SI-7

Reaction between **A** (R = *p*-CN) and **B** in C_6D_6. The ratio of **A:B** is 1:1. A spatula tip of CuI was added at the start of the reaction.

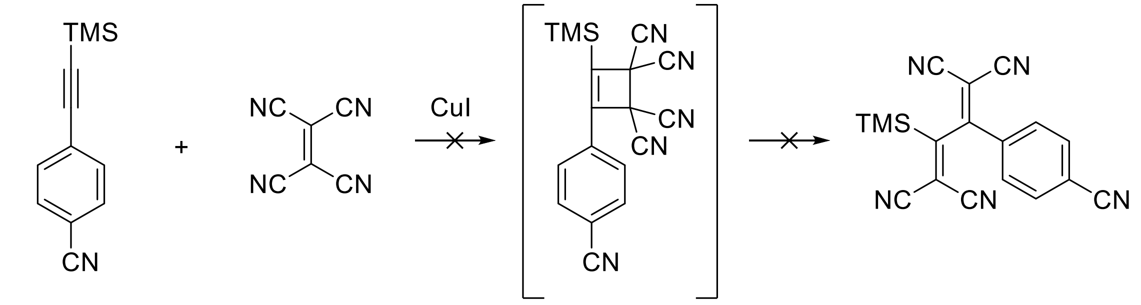

A **B** **C** **P**

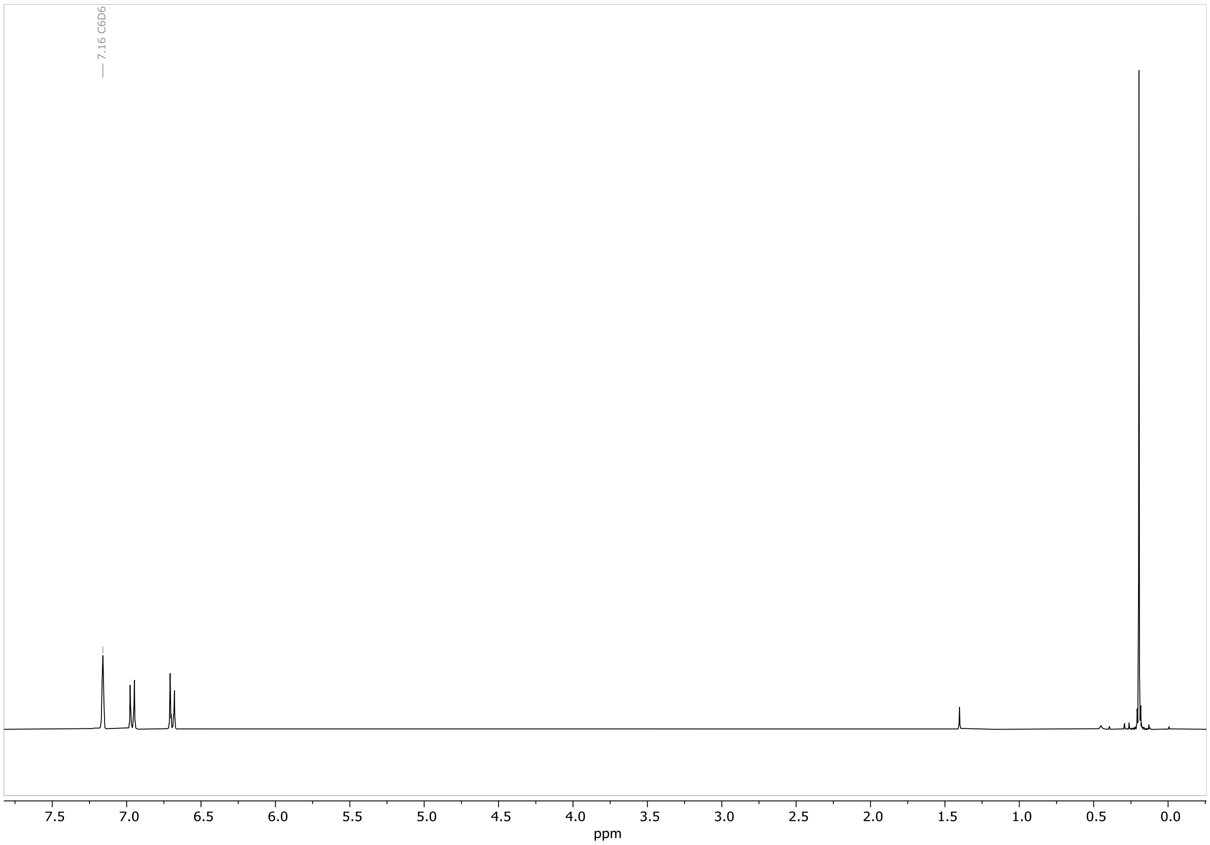

Figure S8.7.1. ^{1}H-NMR spectrum (300 MHz, C_6D_6) corresponding to entry SI-7. Spectrum was recorded after 30 minutes of reaction time.

Section 8.8 – Entry SI-8

Reaction between **A** (R = *p*-CN) and **B** in C_6D_6. The ratio of **A:B** is 1:1. A spatula tip of $AlCl_3$ was added at the start of the reaction.

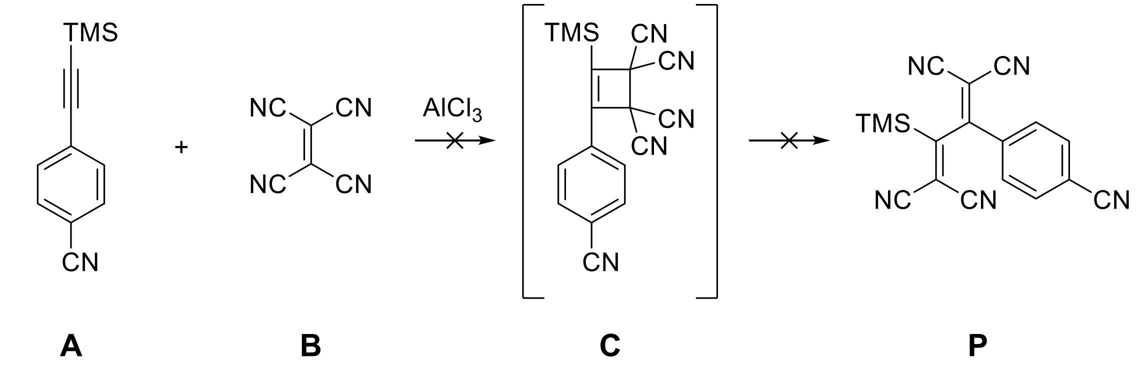

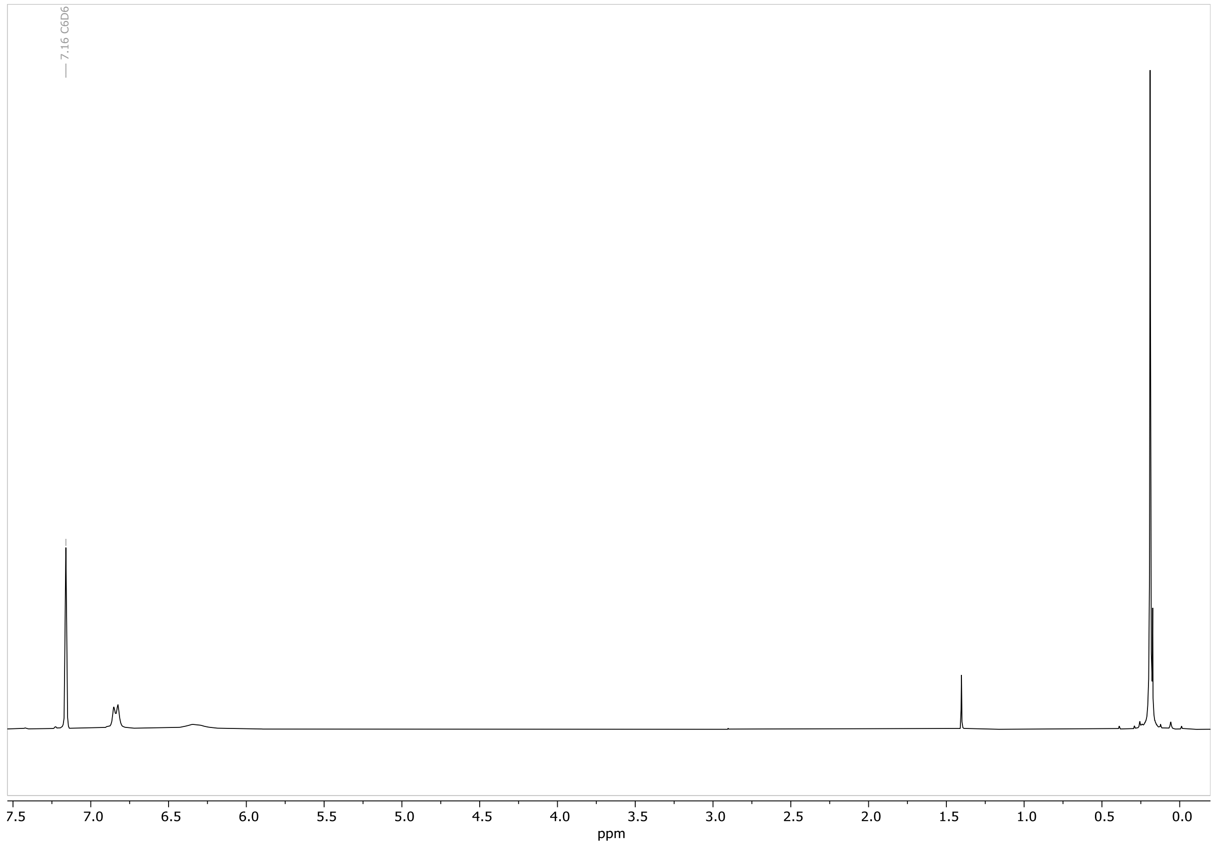

Figure S8.8.1. ^{1}H-NMR spectrum (300 MHz, C_6D_6) corresponding to entry SI-8. Spectrum was recorded after 30 minutes of reaction time. The starting material decomposed.

Section 8.9 – Entry SI-9

Reaction between **A** (R = *p*-CN) and **B** in C_6D_6. The ratio of **A:B** is 1:1. A spatula tip of $ZnCl_2$ was added at the start of the reaction.

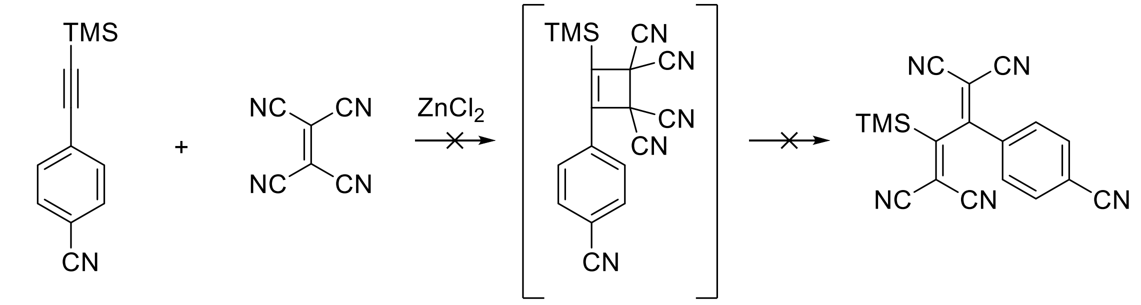

A **B** **C** **P**

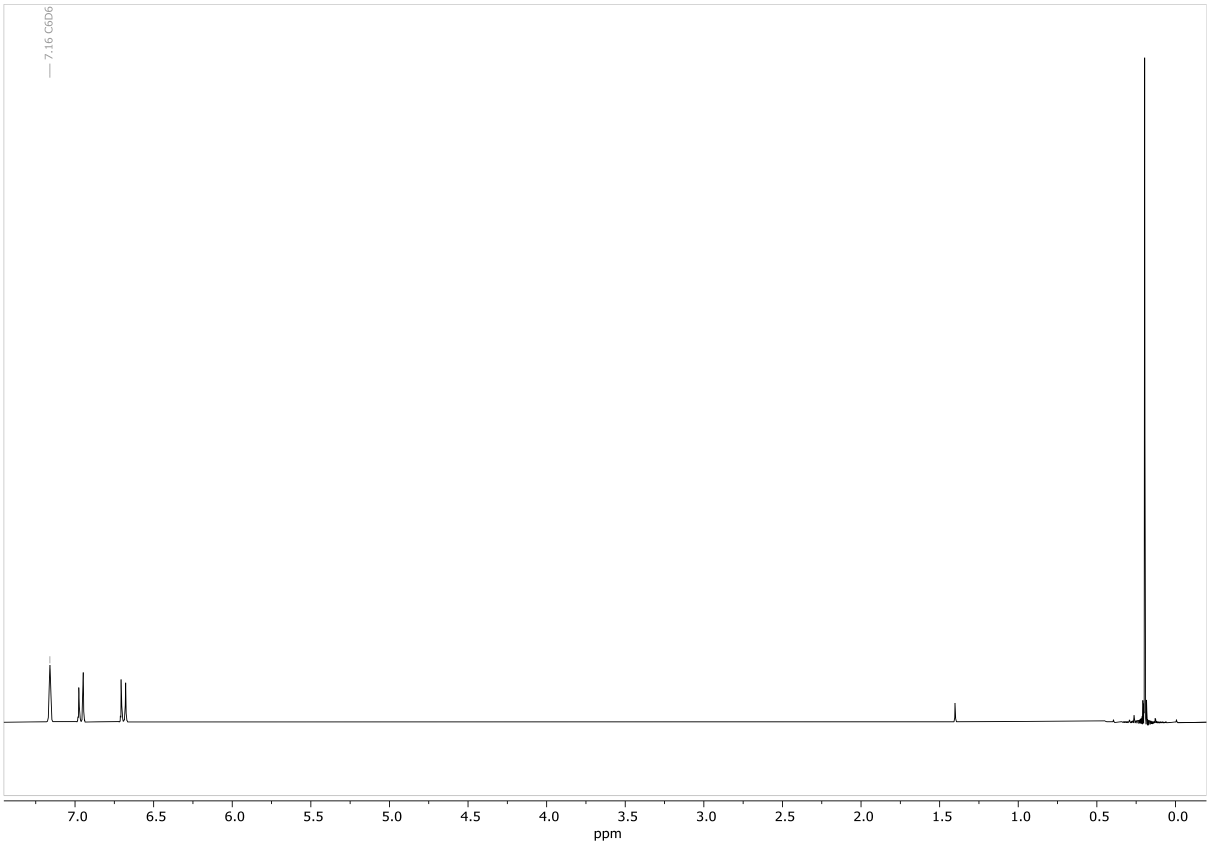

Figure S8.9.1. ^{1}H-NMR spectrum (300 MHz, C_6D_6) corresponding to entry SI-9. Spectrum was recorded after 30 minutes of reaction time.

Section 8.10 – Entry SI-10

Reaction between **A** (R = *p*-CN) and **B** in C_6D_6. The ratio of **A:B** is 1:1. A spatula tip of NH_4OAc was added at the start of the reaction.

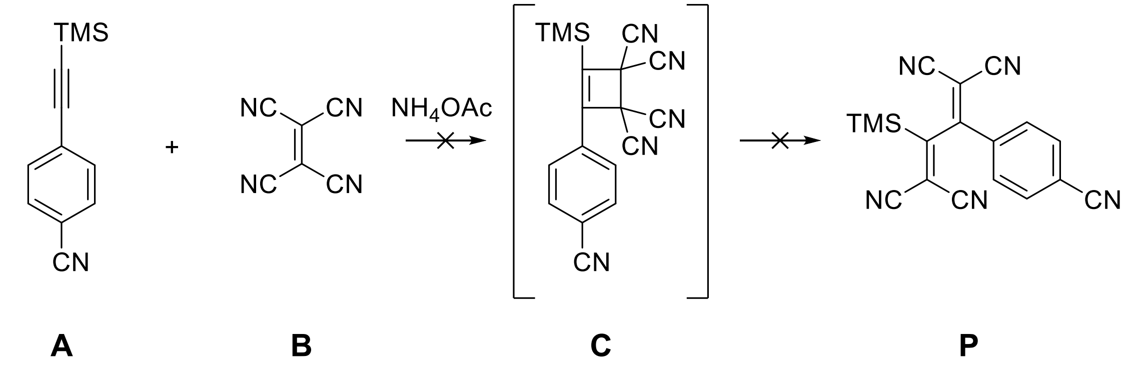

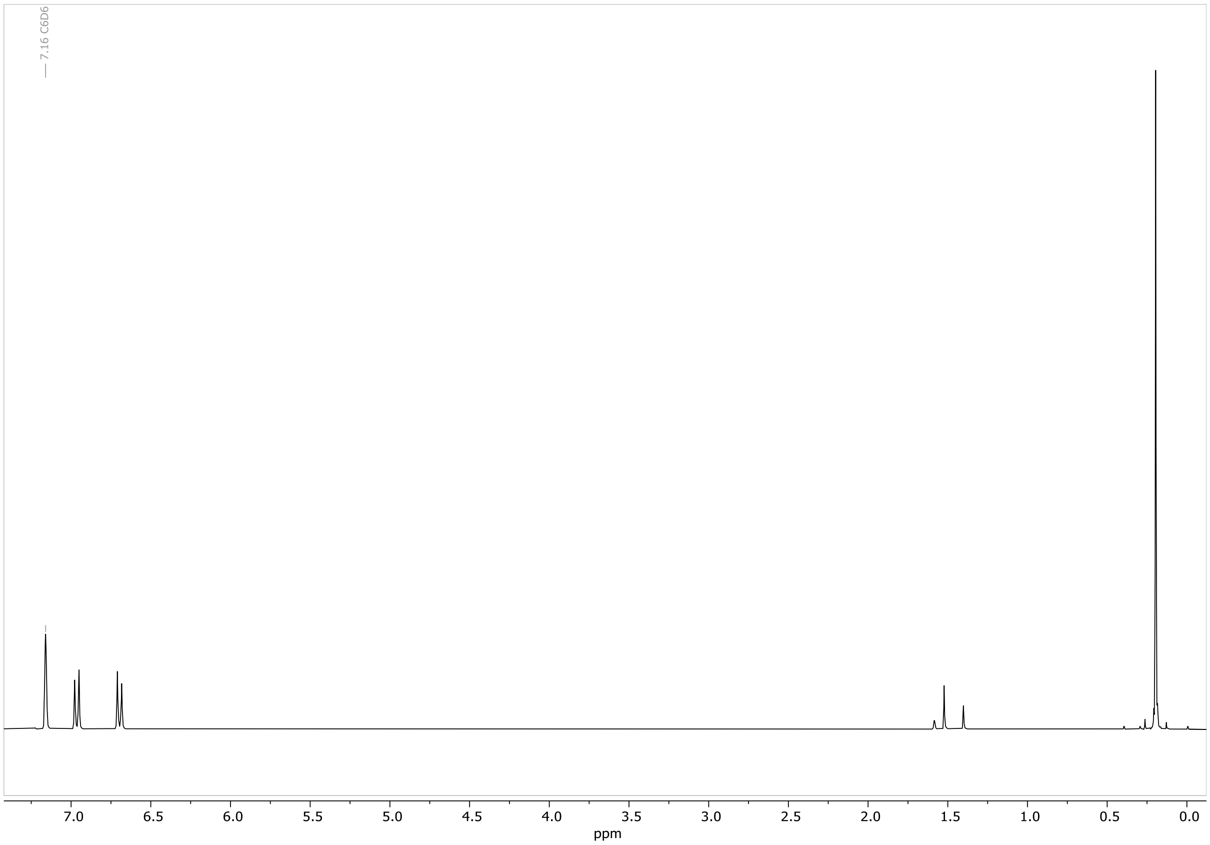

Figure S8.10.1. ^{1}H-NMR spectrum (300 MHz, C_6D_6) corresponding to entry SI-10. Spectrum was recorded after 30 minutes of reaction time.

Section 8.11 – Entry SI-11

Reaction between **A** (R = *p*-NHAc) and **B** in C_6D_6. The ratio of **A:B** is 1:1.

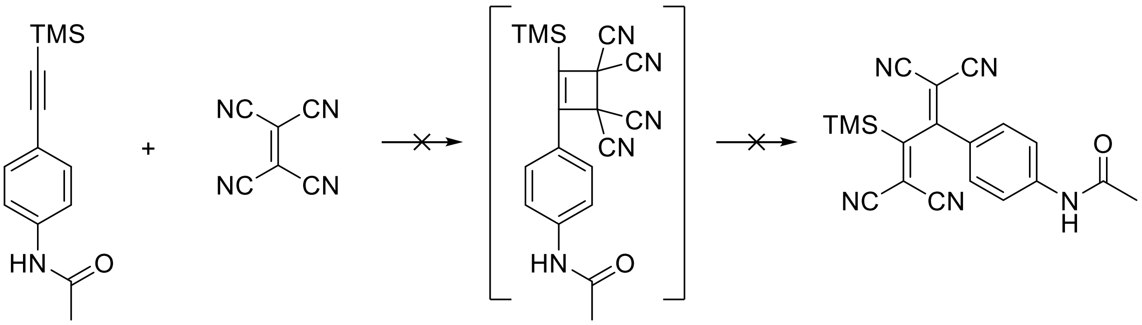

A **B** **C** **P**

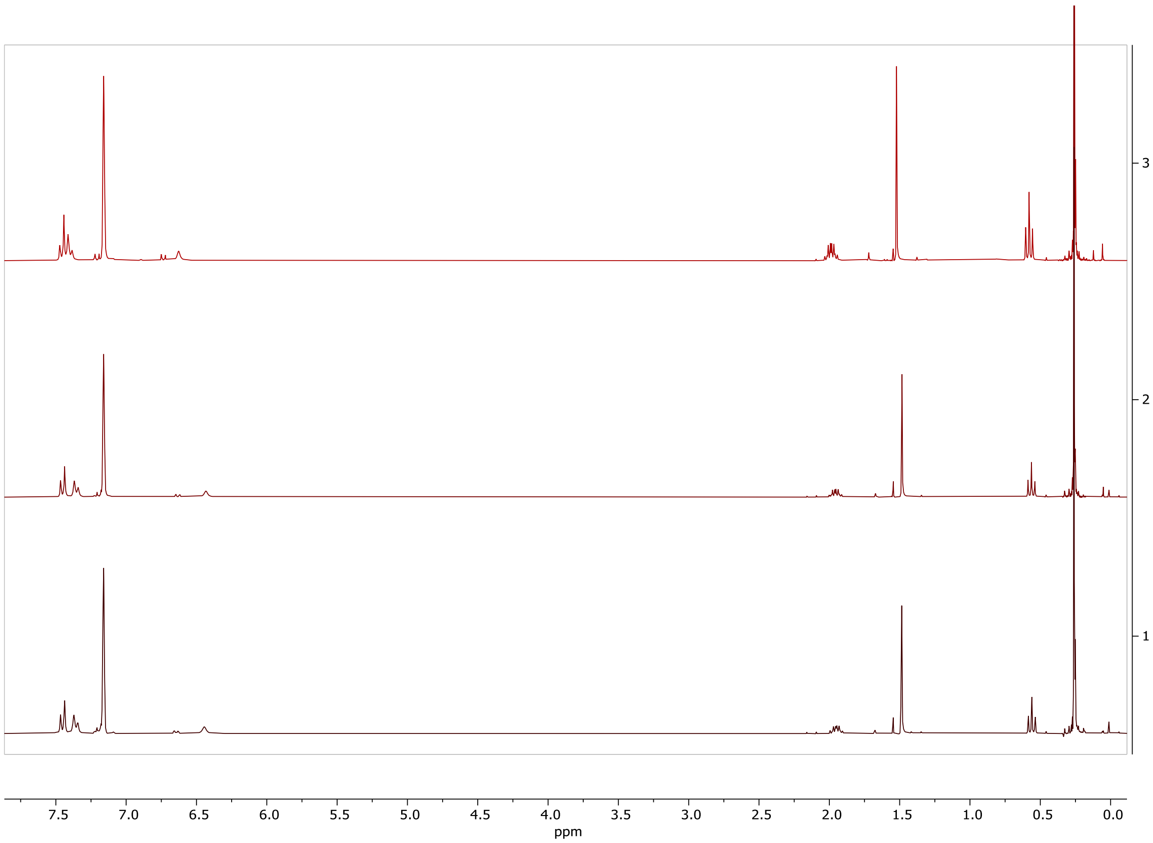

Figure S8.11.1. ^{1}H-NMR spectra (300 MHz, C_6D_6) corresponding to entry SI-11. Spectra were recorded after 10 minutes (bottom), 1 hour (middle) and 1 day (top) of reaction time.

Section 8.12 – Entry SI-12

Reaction between **A** (R = *m*-NH_2) and **B** in C_6D_6. The ratio of **A:B** is 1:1.

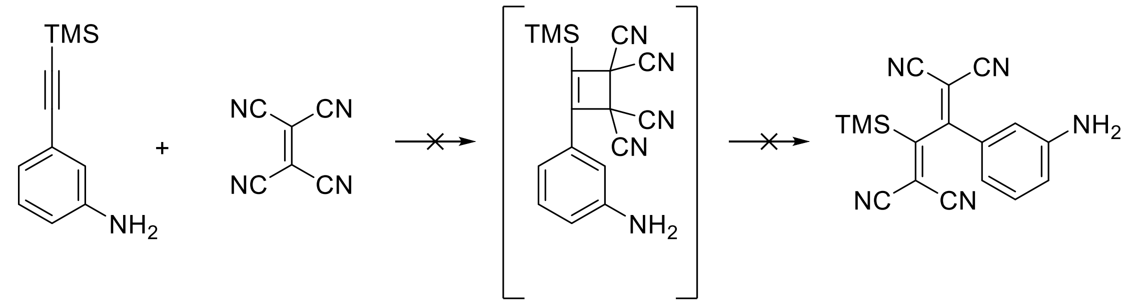

A **B** **C** **P**

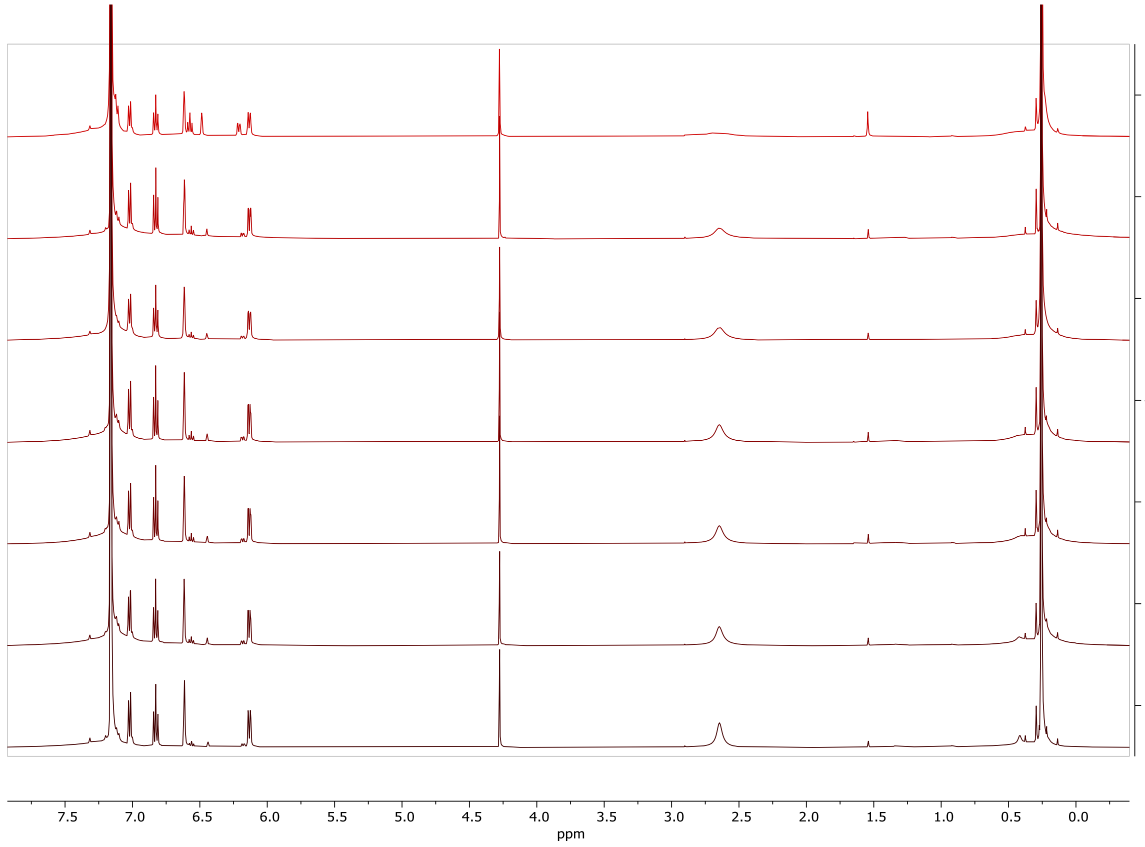

Figure S8.12.1. ^1H-NMR spectra (300 MHz, C_6D_6) corresponding to entry SI-12. Starting from the bottom, the first 6 spectra were evenly recorded over 100 minutes and the last spectrum after 48 hours. The signal at 4.27 ppm is assigned to CH_2Cl_2.

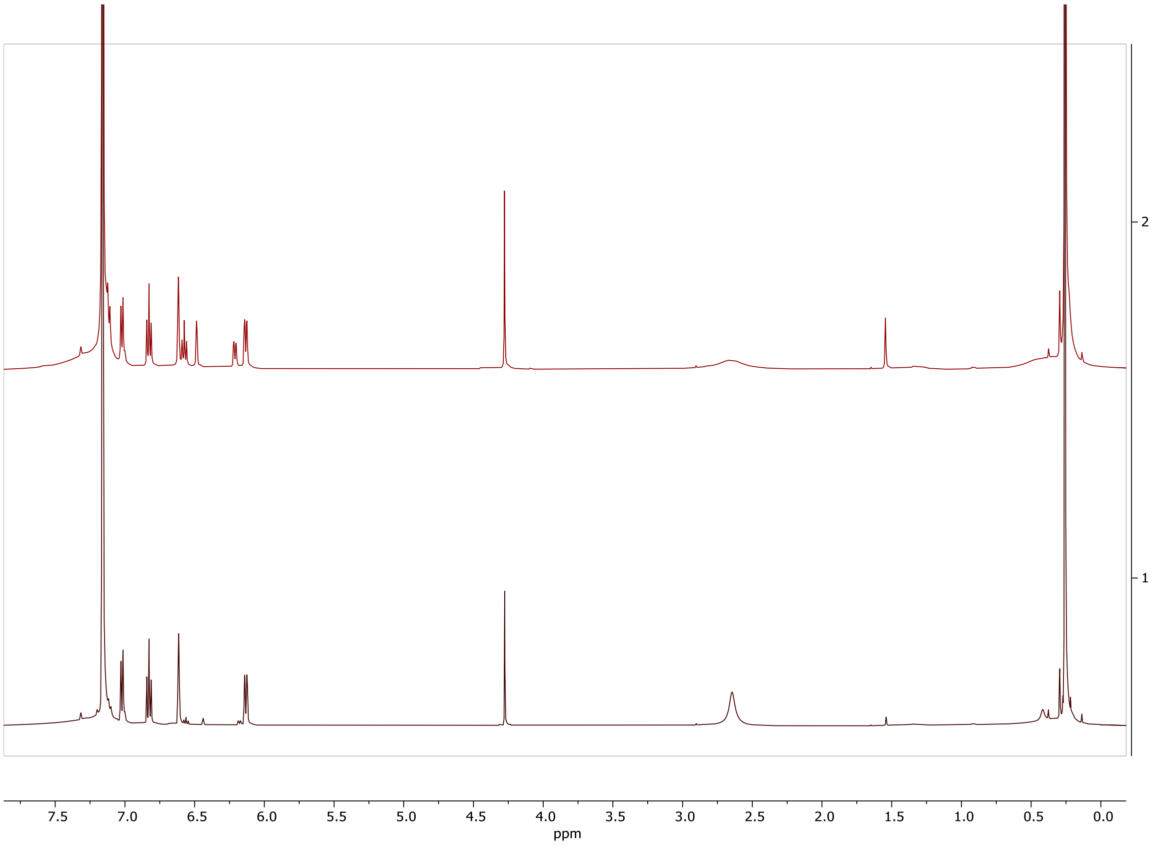

Figure S8.12.2. ^{1}H-NMR spectra (300 MHz, C_6D_6) corresponding to entry SI-12. The bottom spectrum is the first recorded spectrum recorded immediately after reactants were mixed, and the top spectrum is the last recorded spectrum which was recorded after 48 hours.

Section 8.13 – Entry SI-13

Reaction between **A** (R = *p*-NH_2) and **B** in C_6D_6. The ratio of **A:B** is 1:1. A drop of 1,8-diazabicyclo(5.4.0)undec-7-ene (DBU) was added at the start of the reaction; the reaction mixture turned black and sticky as soon as the DBU hit the solvent surface. The aromatic peaks in the recorded spectrum are identical to those of the isolated starting material, suggesting that DBU reacted with **B**, rendering **B** unable to react with **A**.

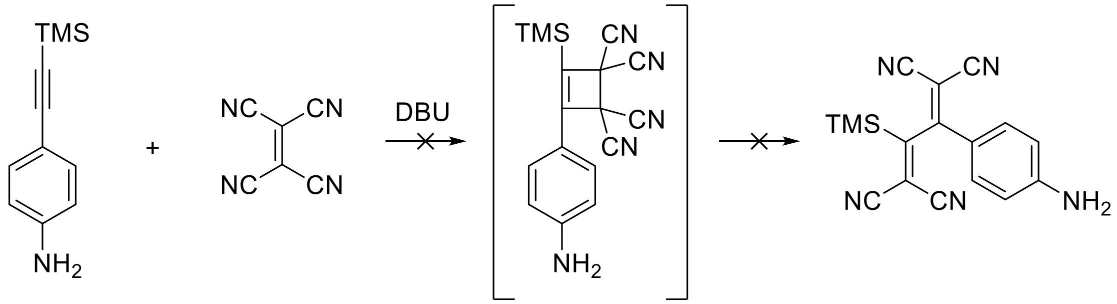

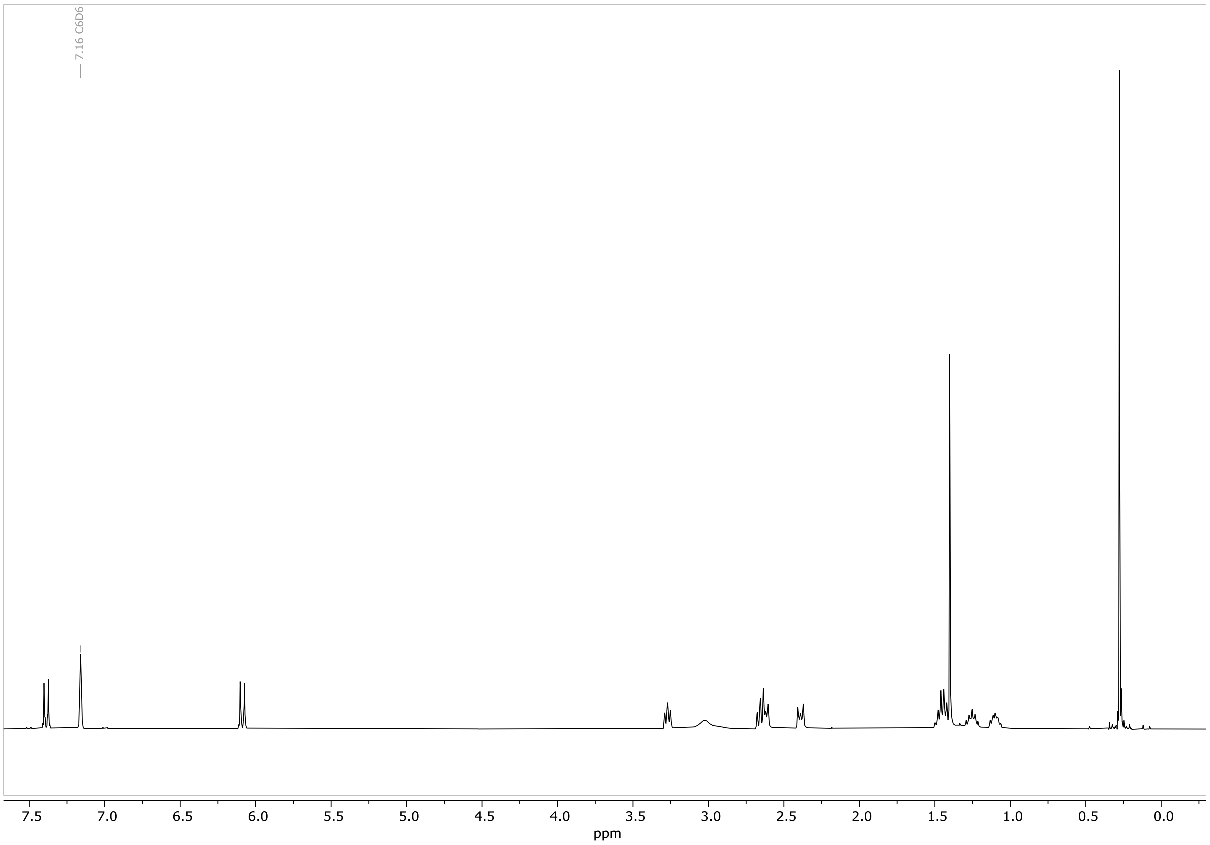

Figure S8.13.1. ^{1}H-NMR spectrum (300 MHz, C_6D_6) corresponding to entry SI-13. The spectrum was recorded after 2 hours of reaction time.

Section 8.14 – Entry SI-14

Reaction between **A** (R = *p*-NH_2) and **B** in C_6D_6. The ratio of **A:B** is 1:1. A drop of Et_3N was added at the start of the reaction; the reaction mixture turned black and sticky as soon as the Et_3N hit the solvent surface. The aromatic peaks in the recorded spectra are identical to those of the isolated starting material, suggesting Et_3N reacted with **B**, rendering **B** unable to react with **A**.

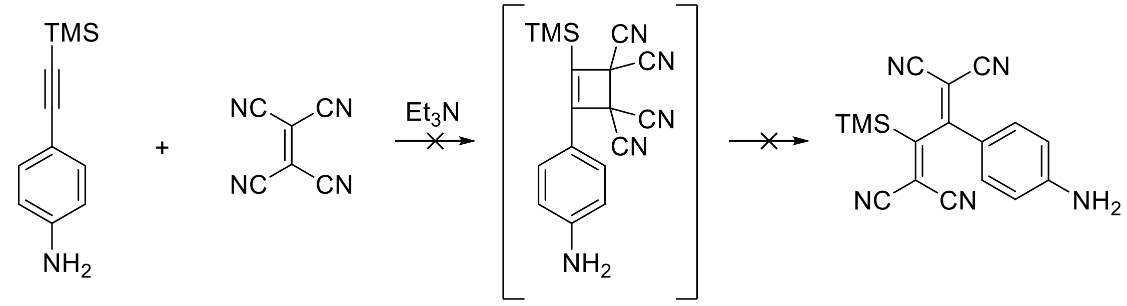

A **B** **C** **P**

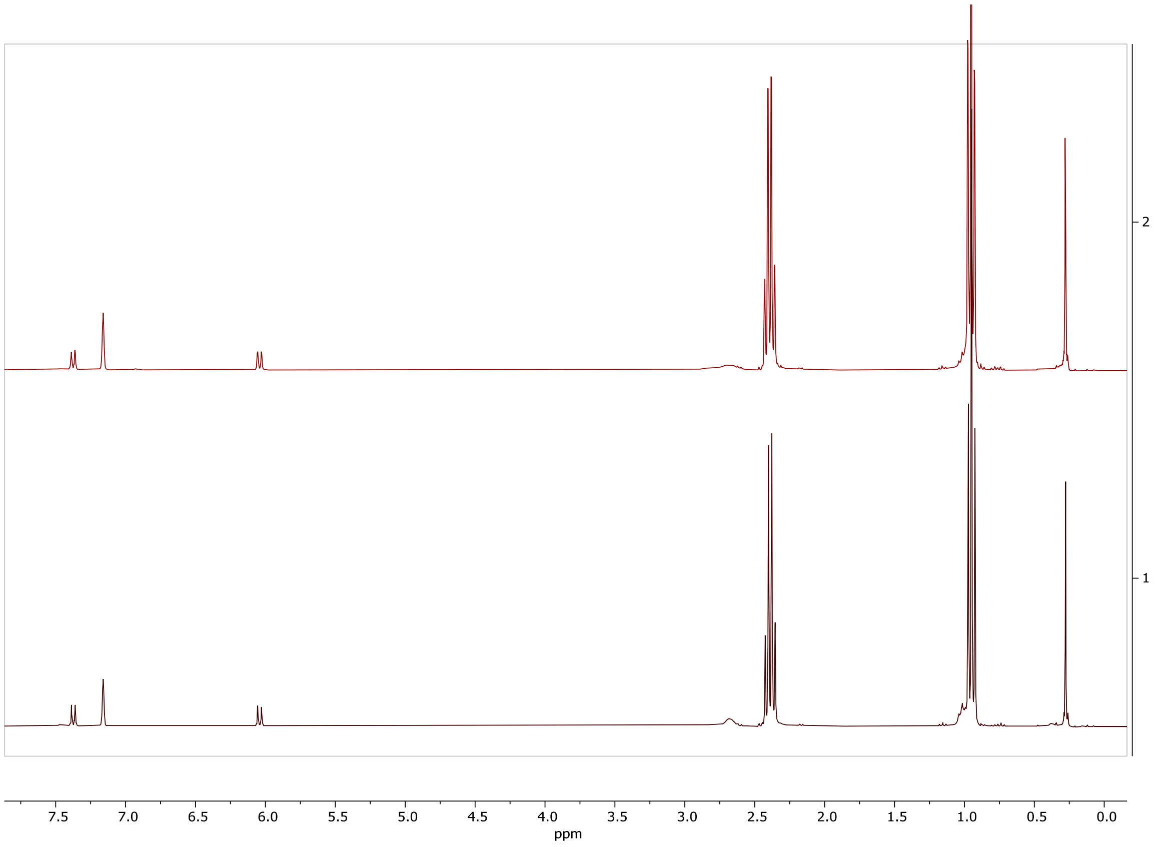

Figure S8.14.1. ^{1}H-NMR spectra (300 MHz, C_6D_6) corresponding to entry SI-14. Bottom spectrum was recorded after 23 minutes of reaction time, and the top spectrum was recorded after 24 hours.

Section 8.15 – Entry SI-15

Reaction between **A** (R = *p*-CN) and **B** in CD_3COOD. The ratio of **A:B** is 1:1.

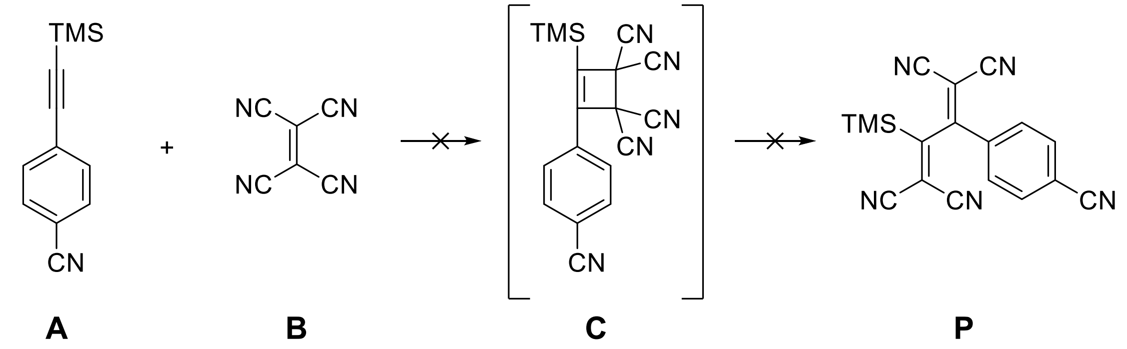

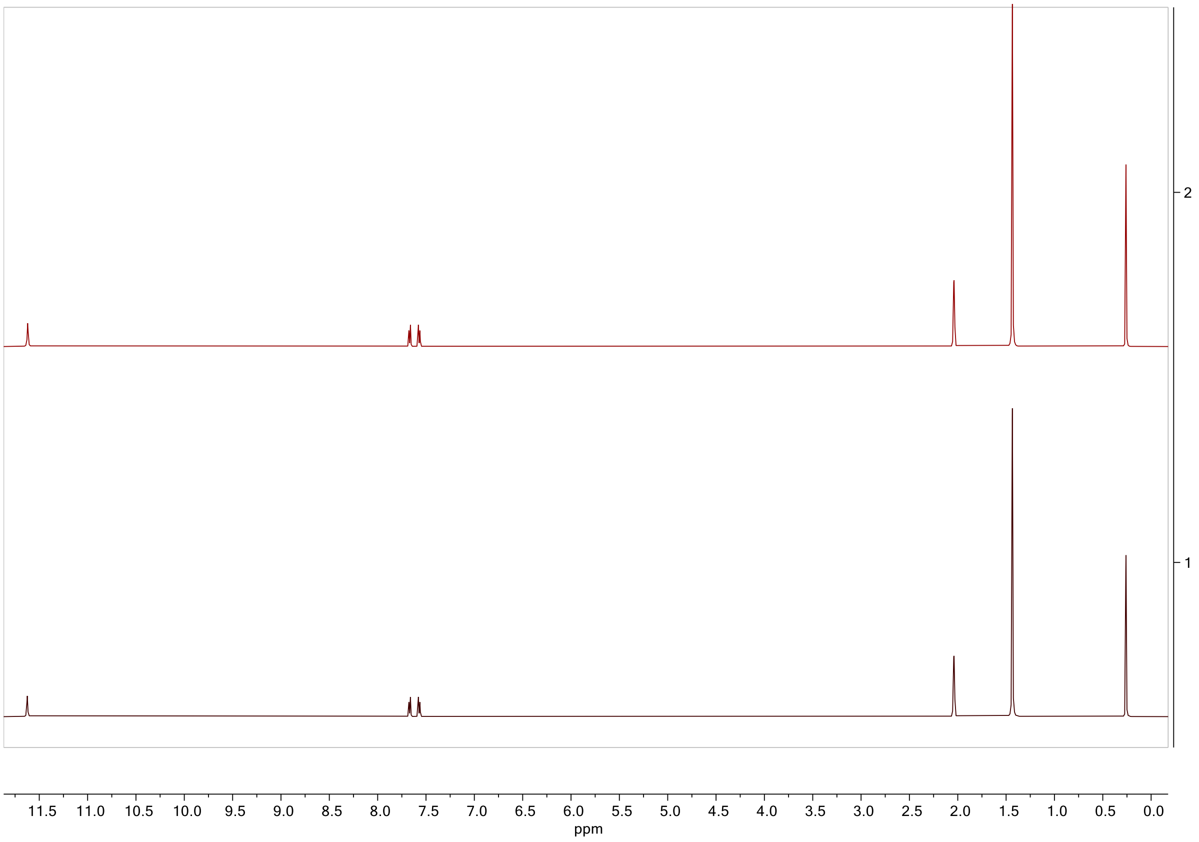

Figure S8.15.1. ^{1}H-NMR spectra (500 MHz, CD_3COOD) corresponding to entry SI-15. Bottom spectrum was recorded immediately after reaction start, and the top spectrum was recorded after 11 hours.

Section 8.16 – Entry SI-16

Reaction between **A** (R = *p*-OMe) and **B** in CD_3COOD. The ratio of **A:B** is 1:1.

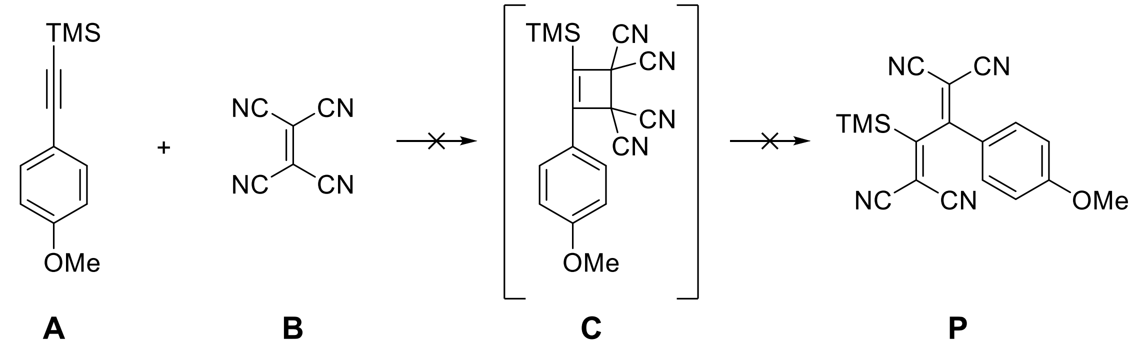

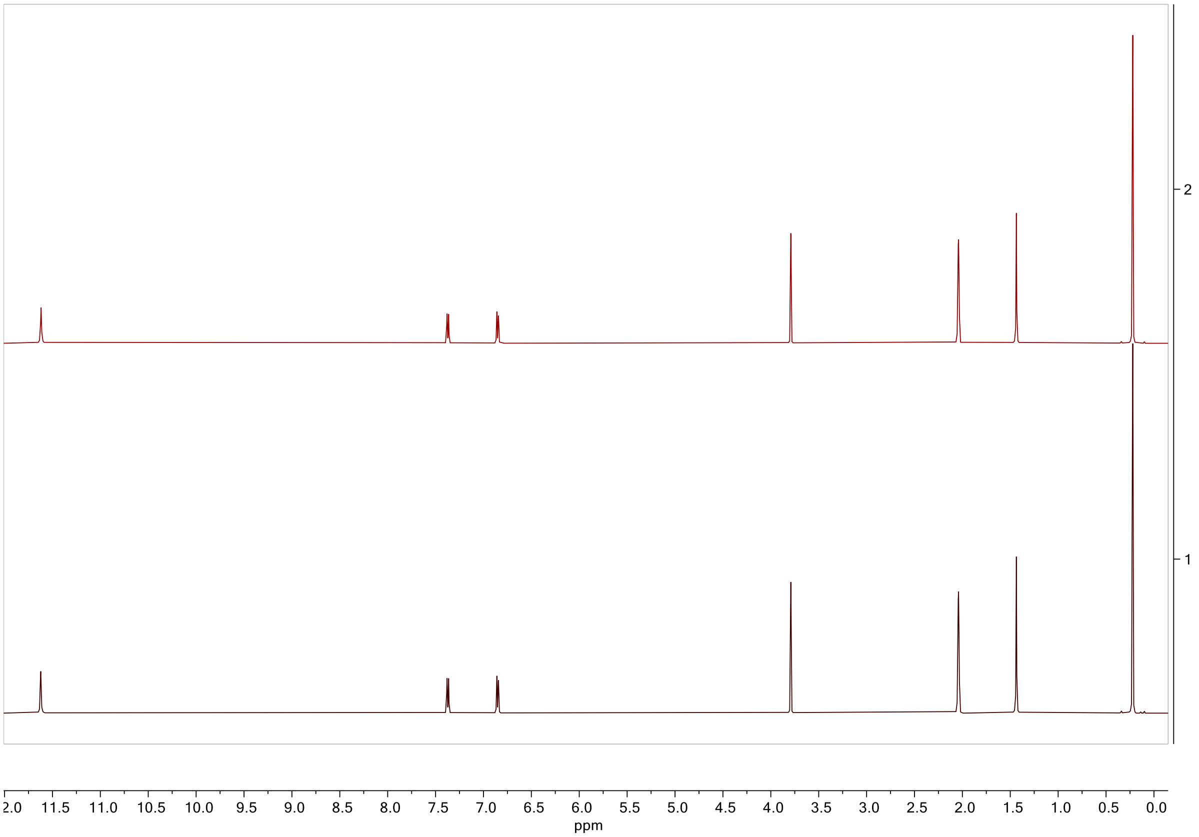

Figure S8.16.1. ^1H-NMR spectra (500 MHz, CD_3COOD) corresponding to entry SI-16. Bottom spectrum was recorded immediately after reaction start, and the top spectrum was recorded after 9 hours.

Section 9 – Spectroscopic data for experiments where intermediate **C** or product **P** were formed

Section 9.1 – Entry SI-17

Reaction between **A** (R = *p*-NH_2) and **B** in $CDCl_3$. The ratio of **A:B** is 1:1. Concentration of **A** and **B** = 0.037 M.

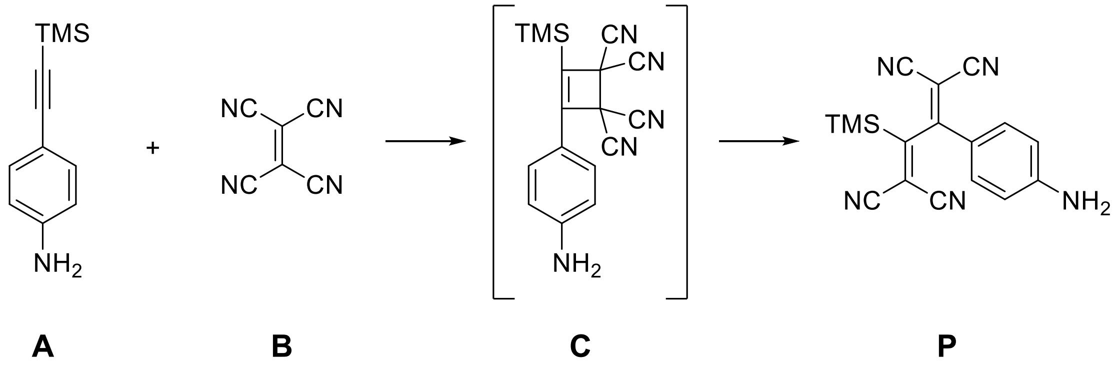

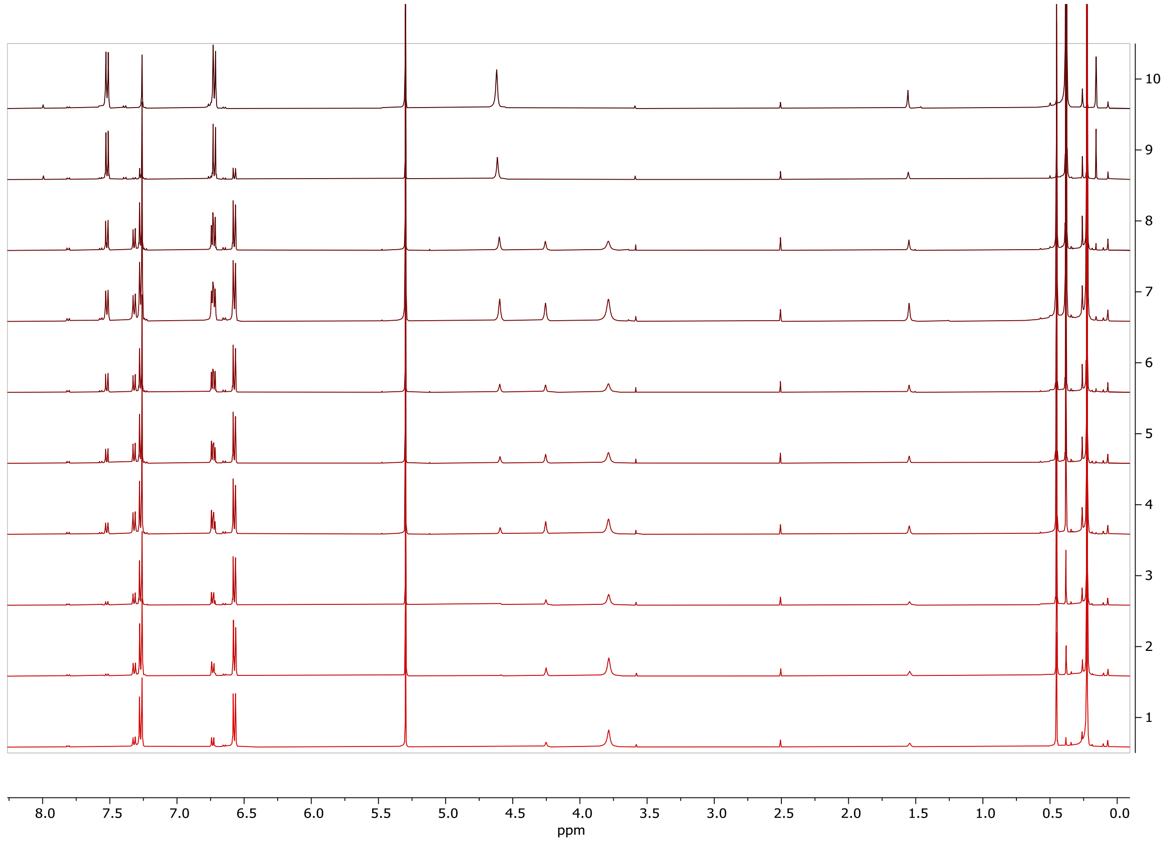

Figure S9.1.1. ^{1}H-NMR spectra (300 MHz, $CDCl_3$) corresponding to entry SI-17. Starting from the bottom, each spectrum was recorded approximately at 10 minute intervals. The signal at 2.50 ppm is an impurity from compound **A**.

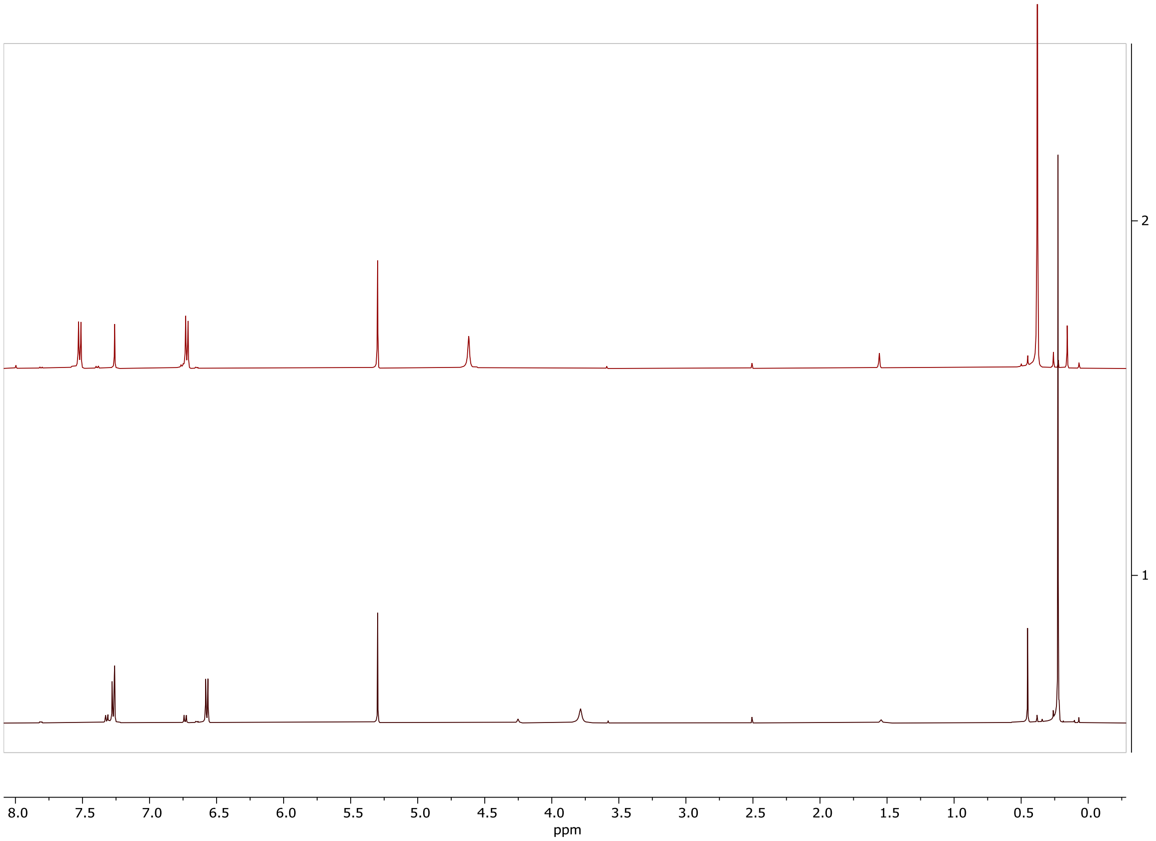

Figure S9.1.2. ^{1}H-NMR spectra (300 MHz, $CDCl_3$) corresponding to entry SI-17. Bottom spectrum was recorded at start of the reaction and top spectrum was recorded after a suitable time period had passed.

Section 9.2 – Entry SI-18

Reaction between **A** (R = *p*-NH_2) and **B** in acetone-d_6. The ratio of **A**:**B** is 1:1. Concentration of **A** and **B** = 0.035 M.

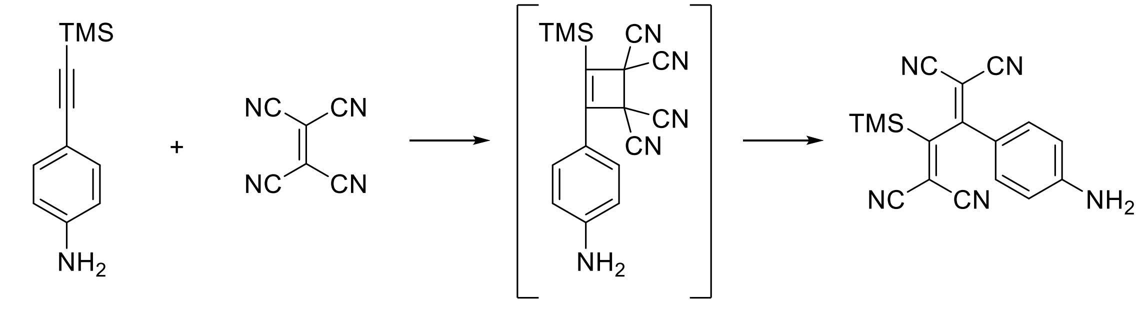

A **B** **C** **P**

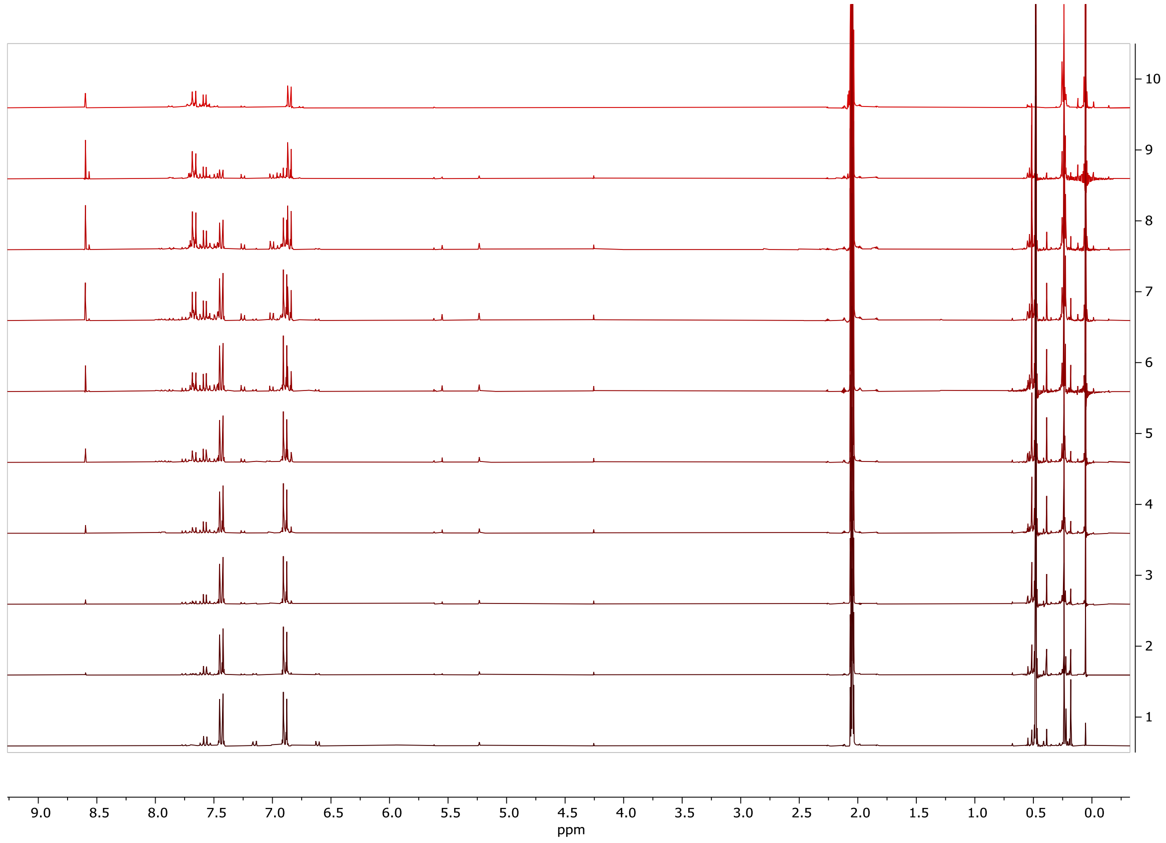

Figure S9.2.1. ^{1}H-NMR spectra (300 MHz, acetone-d_6) corresponding to entry SI-18. Starting from the bottom, each spectrum was recorded approximately at 10 minute intervals except the last 3 spectra which were recorded after 2 hours, 3 hours and 24 hours of reaction time, respectively. The signal at 8.55 ppm corresponds to the product of protodesilylation of **P**.

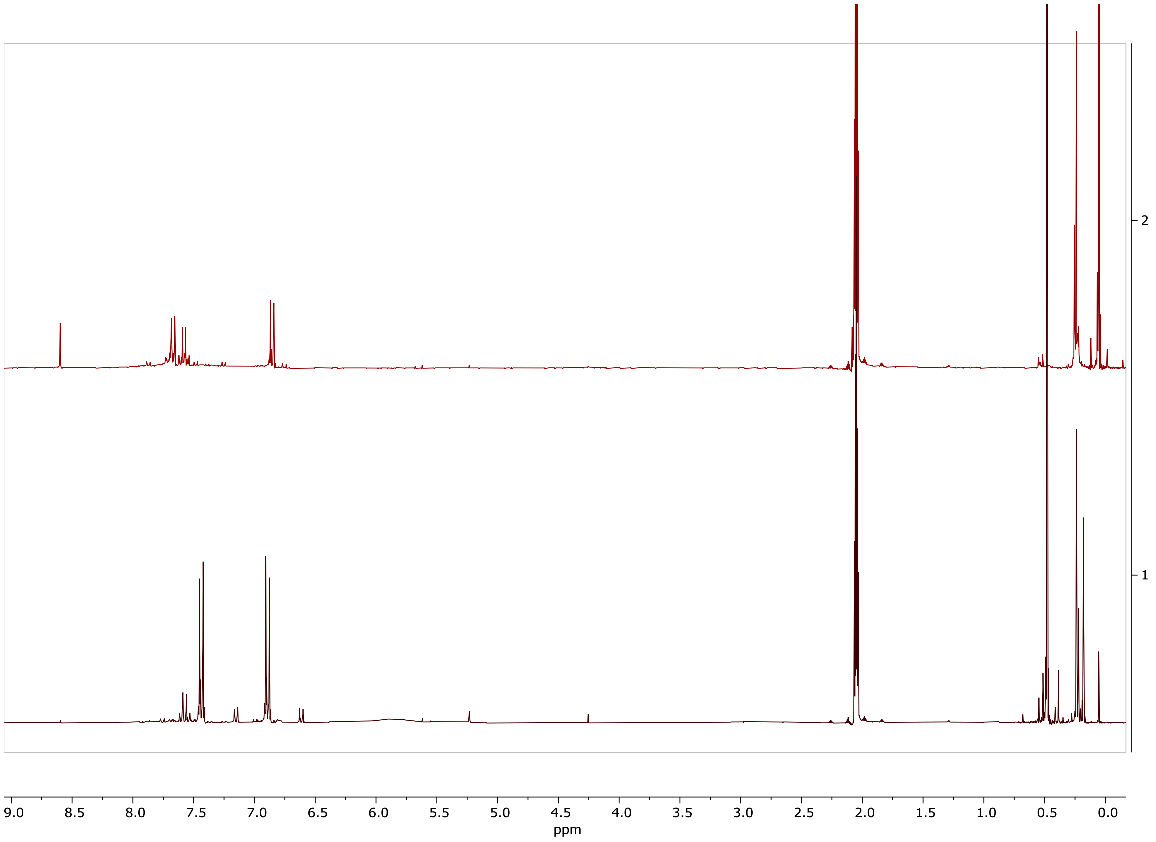

Figure S9.2.2. ^{1}H-NMR spectra (300 MHz, acetone-d_6) corresponding to entry SI-18. Bottom spectrum was recorded at start of the reaction, and top spectrum was recorded after a suitable time period had passed.

Section 9.3 – Entry SI-19

Reaction between **A** (R = *p*-NH_2) and **B** in CD_3CN. The ratio of **A:B** is 1:1. Concentration of **A** and **B** = 0.037 M.

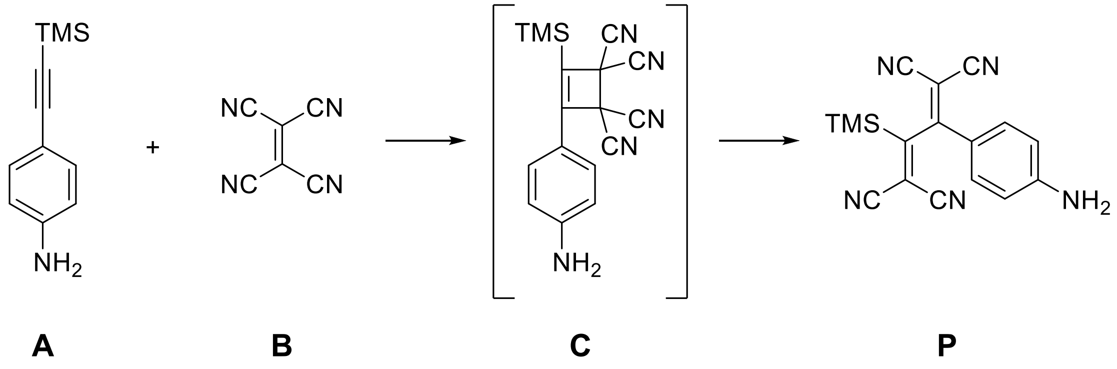

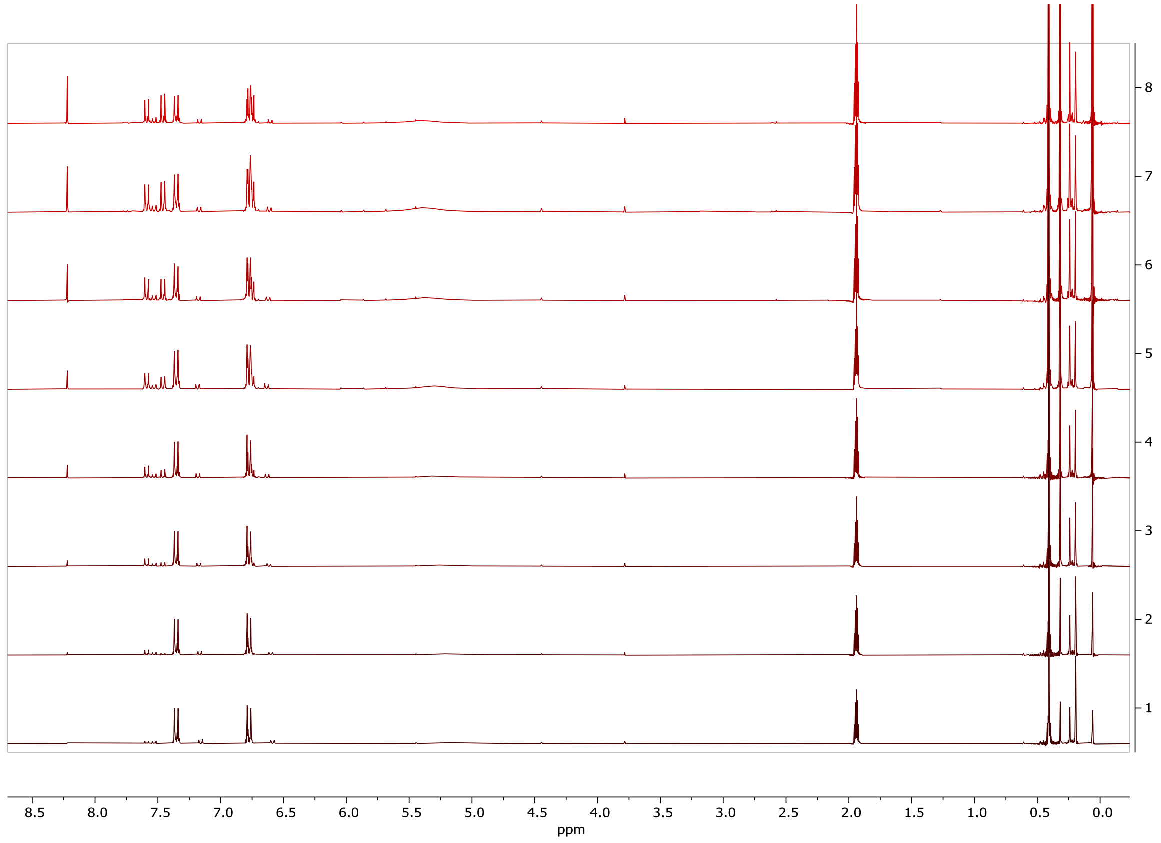

Figure S9.3.1. ^{1}H-NMR spectra (300 MHz, CD_3CN) corresponding to entry SI-19. Starting from the bottom, each spectrum was recorded approximately at 10 minute intervals. The signal at 8.55 ppm corresponds to the product of protodesilylation of **P**.

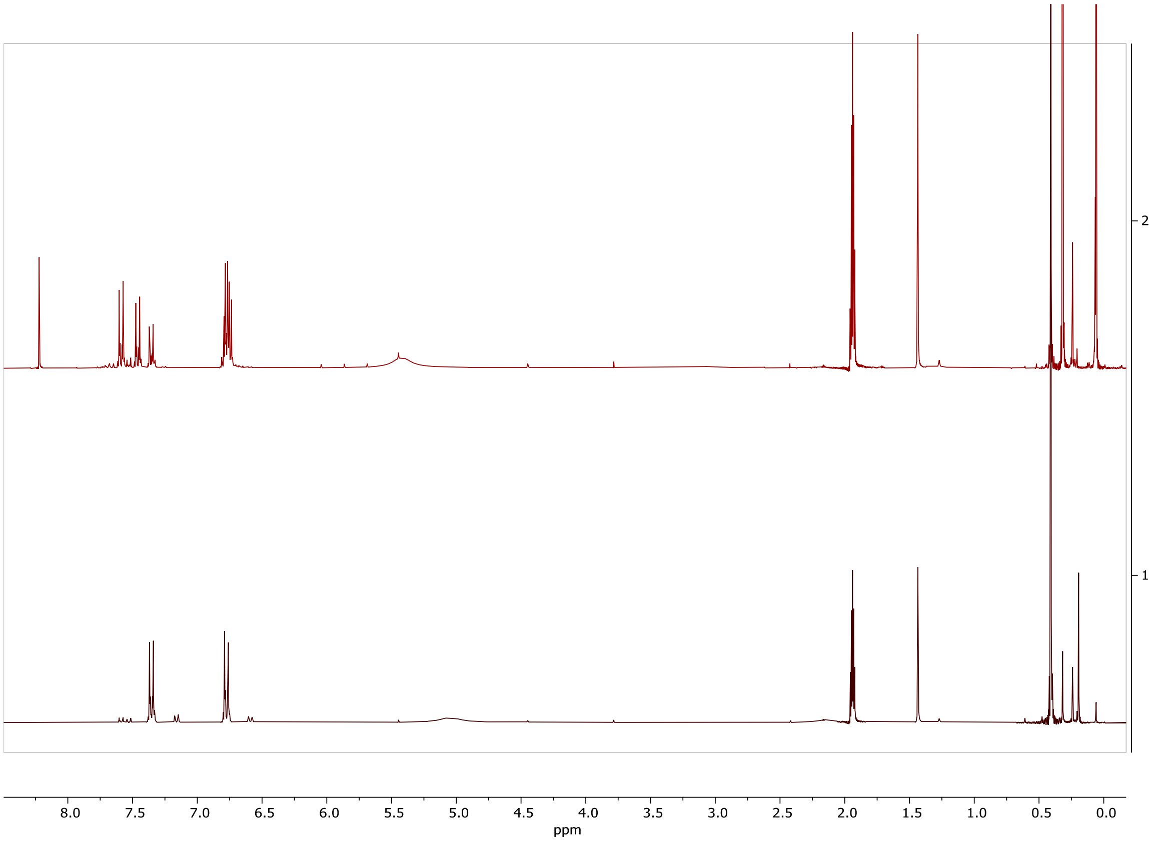

Figure S9.3.2. ^{1}H-NMR spectra (300 MHz, CD_3CN) corresponding to entry SI-19. Bottom spectrum was recorded at start of the reaction, and top spectrum was recorded after a suitable time period had passed.

Section 9.4 – Entry SI-20

Reaction between **A** (R = *p*-NH_2) and **B** in tetrahydrofurane-d_8. The ratio of **A:B** is 1:1.

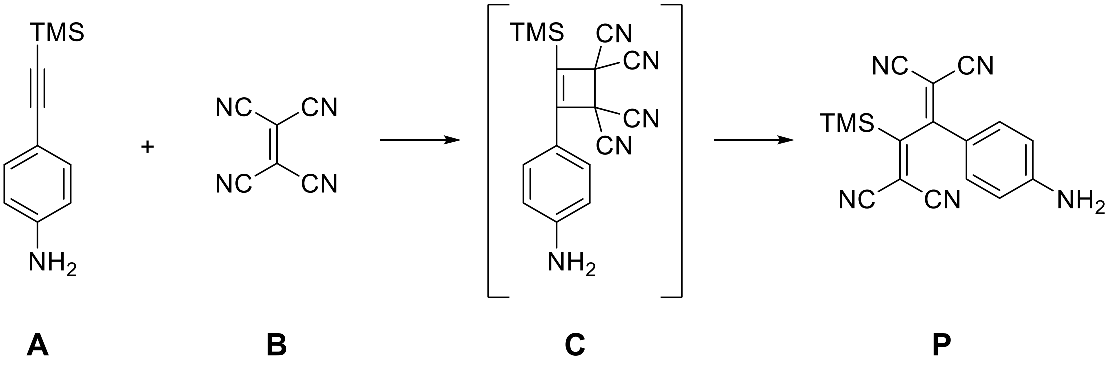

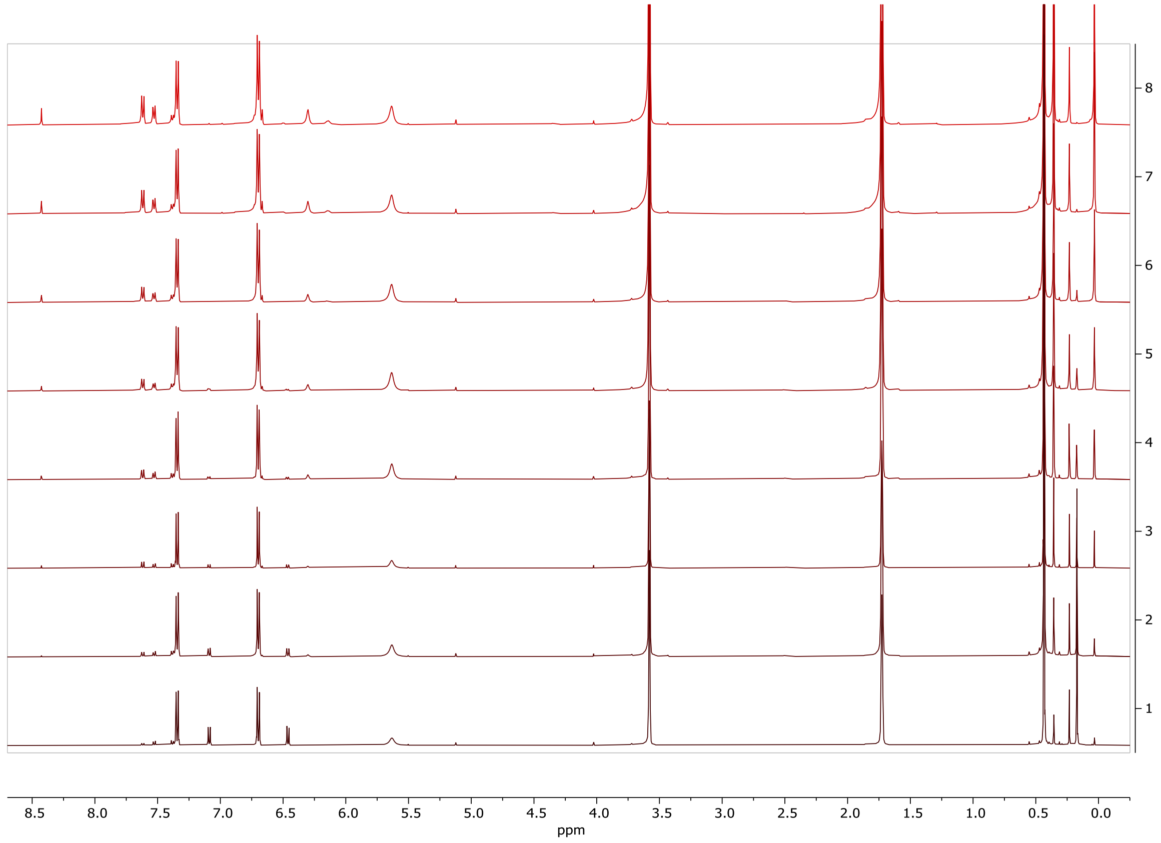

Figure S9.4.1. ^1H-NMR spectra (300 MHz, tetrahydrofurane-d_8) corresponding to entry SI-20. Starting from the bottom, each spectrum was recorded approximately at 10 minute intervals. The signal at 8.55 ppm corresponds to the product of protodesilylation of **P**.

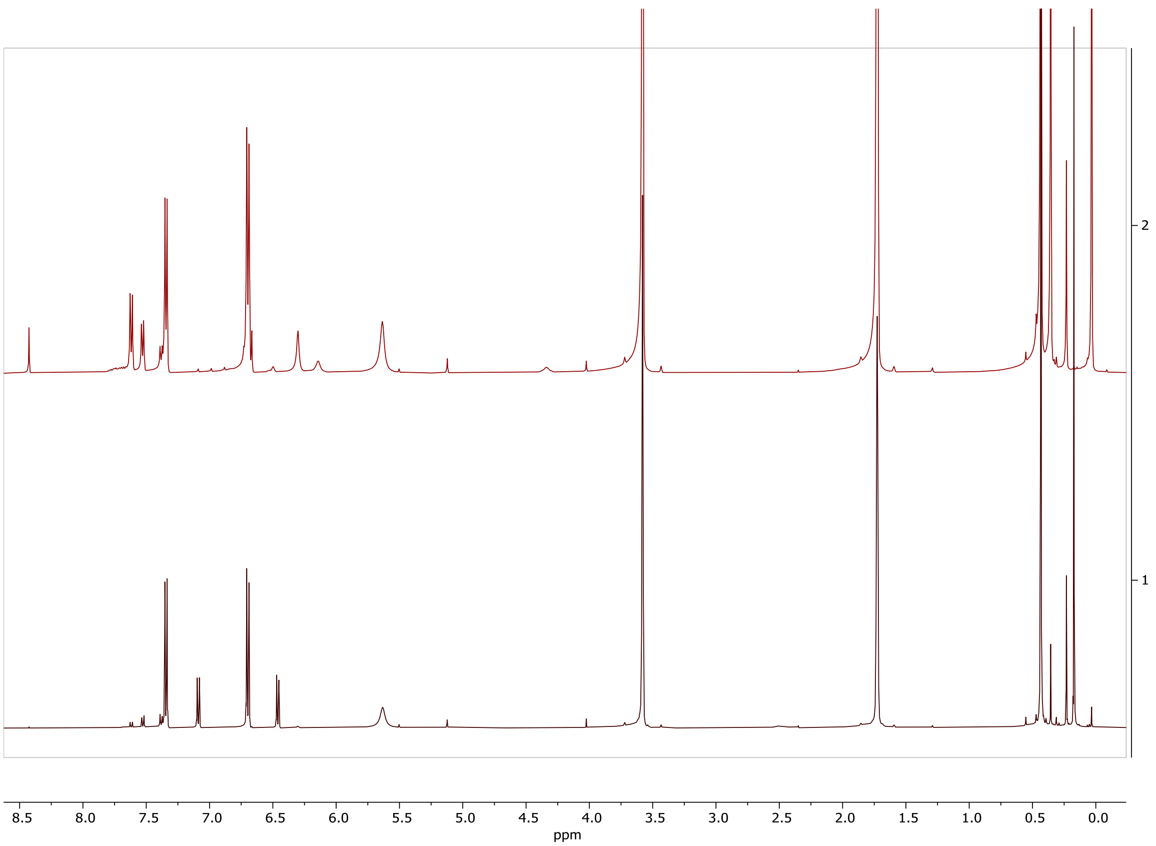

Figure S9.4.2. ^{1}H-NMR spectra (300 MHz, tetrahydrofurane-d_8) corresponding to entry SI-20. Bottom spectrum was recorded at start of the reaction, and top spectrum was recorded after a suitable time period had passed.

Section 9.5 – Entry SI-21

Reaction between **A** (R = *p*-NH_2) and **B** in toluene-d_8. The ratio of **A**:**B** is 1:1. Concentration of **A** and **B** = 0.037 M.

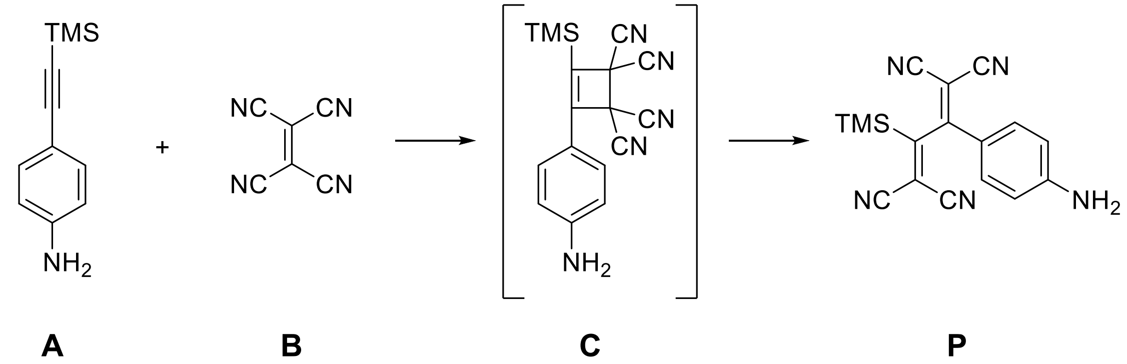

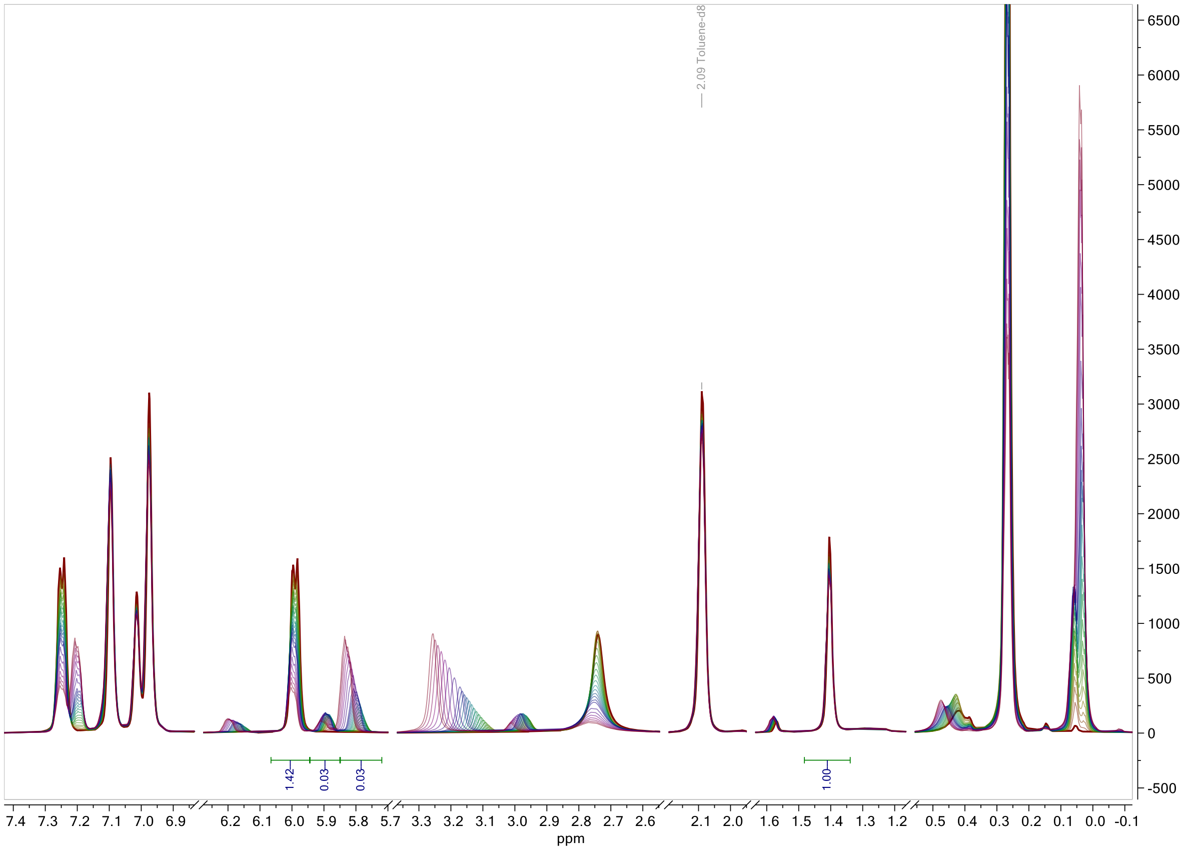

Figure S9.5.1. ^{1}H-NMR spectra (500 MHz, toluene-d_8; selected regions shown) corresponding to entry SI-21. Superimposed spectra of all ^{1}H NMR spectra recorded during the experiment. The first spectrum recorded is colored red and the last spectrum recorded is colored purple. The spectra are recorded precisely every 15 minutes. Only the regions with signals are shown; intermediate regions were removed as indicated by the slashes. The signal at 1.4 ppm corresponds to cyclohexane which was used as an internal standard.

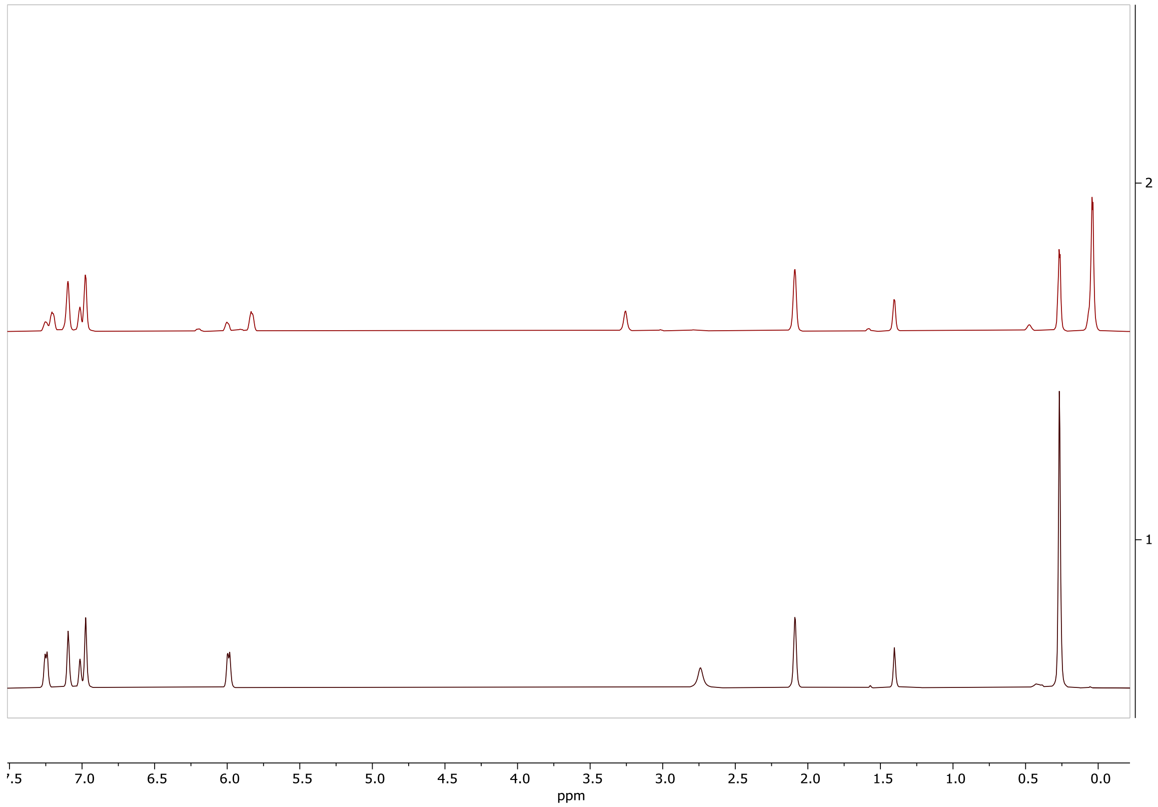

Figure S9.5.2. ^{1}H-NMR spectra (500 MHz, toluene-d_8) corresponding to entry SI-21. Bottom spectrum was recorded at start of the reaction, and top spectrum was recorded after 9.5 hours of reaction time. The signal at 1.4 ppm corresponds to cyclohexane which was used as an internal standard.

Section 9.6 – Entry SI-22

Reaction between **A** (R = *p*-NH_2) and **B** in toluene-d_8. The ratio of **A:B** is 1:5. Concentration of **A** = 0.037 M.

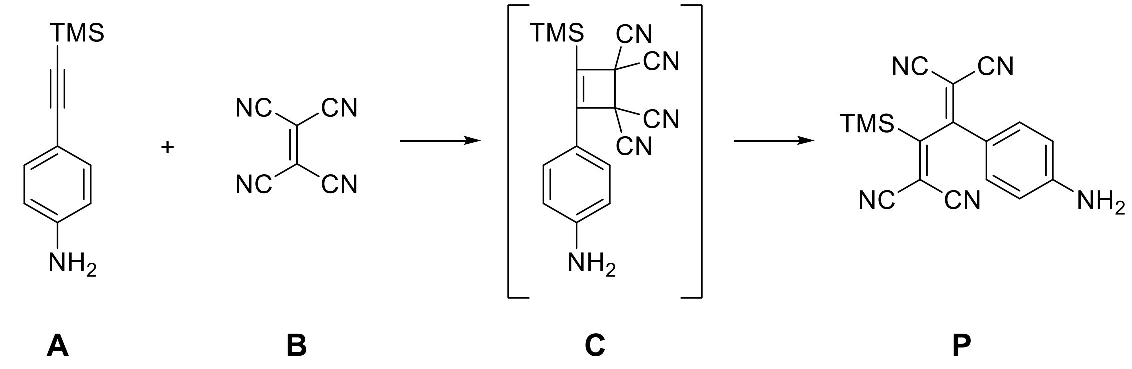

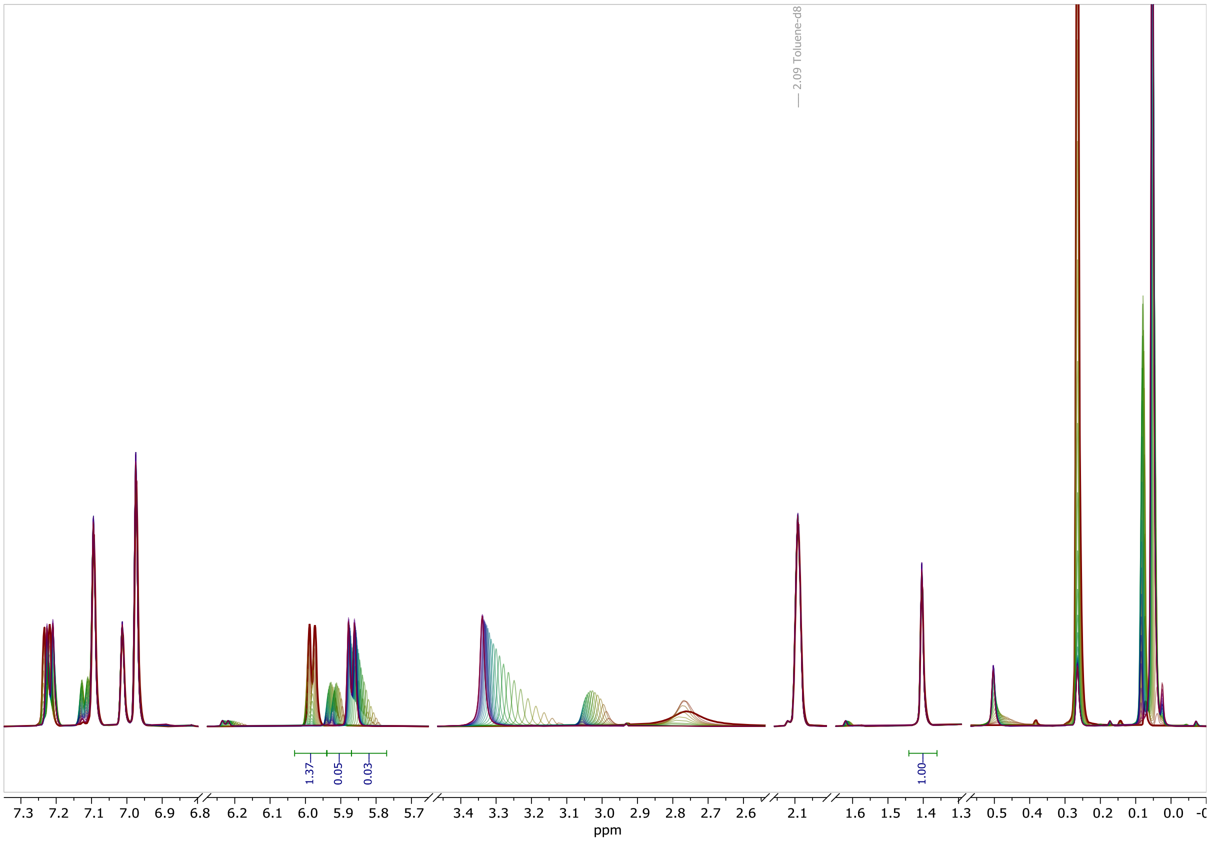

Figure S9.6.1. ^{1}H-NMR spectra (500 MHz, toluene-d_8; selected regions shown) corresponding to entry SI-22. Superimposed spectra of all ^{1}H NMR spectra recorded during the experiment. The first spectrum recorded is colored red and the last spectrum recorded is colored purple. The spectra are recorded precisely every 15 minutes. Only the regions with signals are shown; intermediate regions were removed as indicated by the slashes. The signal at 1.4 ppm corresponds to cyclohexane which was used as an internal standard.

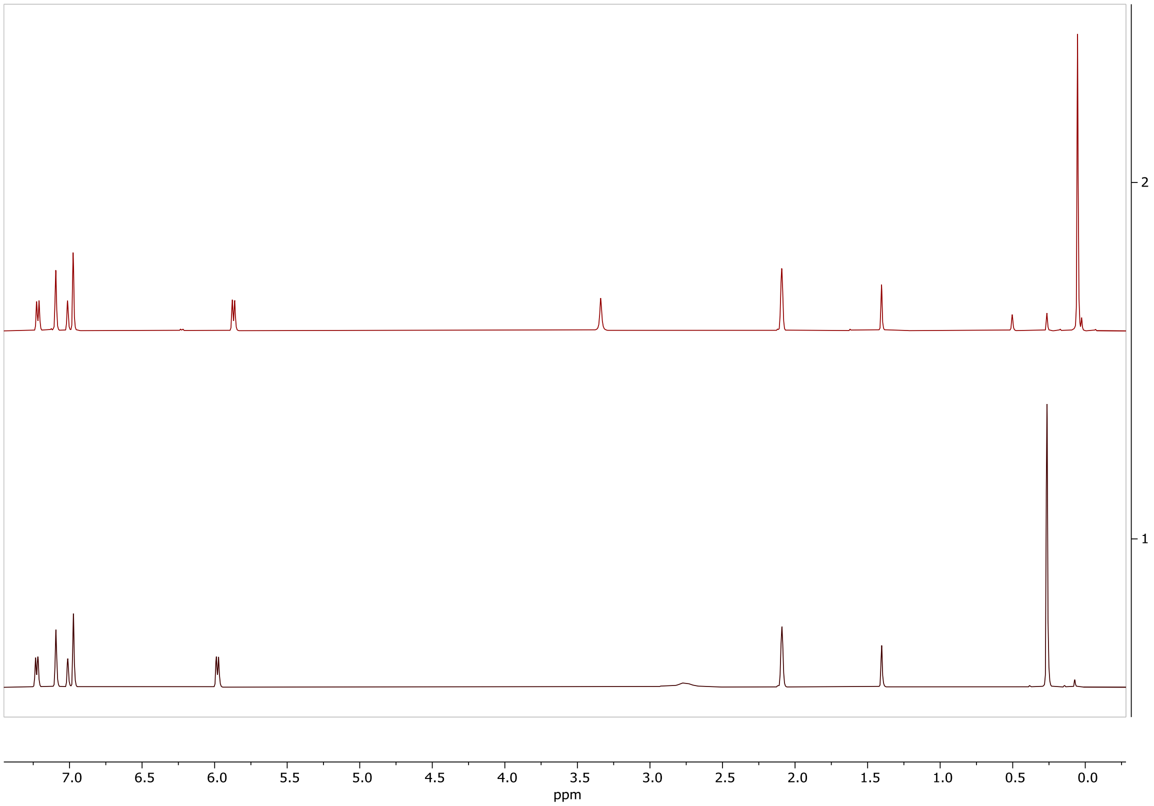

Figure S9.6.2. ^{1}H-NMR spectra (500 MHz, toluene-d_8) corresponding to entry SI-22. Bottom spectrum was recorded at start of the reaction, and top spectrum was recorded after 10.25 hours of reaction time. The signal at 1.4 ppm corresponds to cyclohexane which was used as an internal standard.

Section 9.7 – Entry SI-23

Reaction between **A** (R = *p*-NH_2) and **B** in C_6D_6. The ratio of **A:B** is 1:1. Concentration of **A** and **B** = 0.034 M.

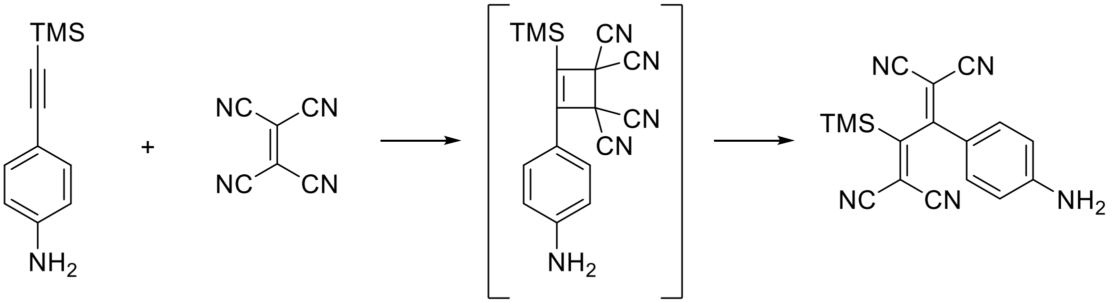

A **B** **C** **P**

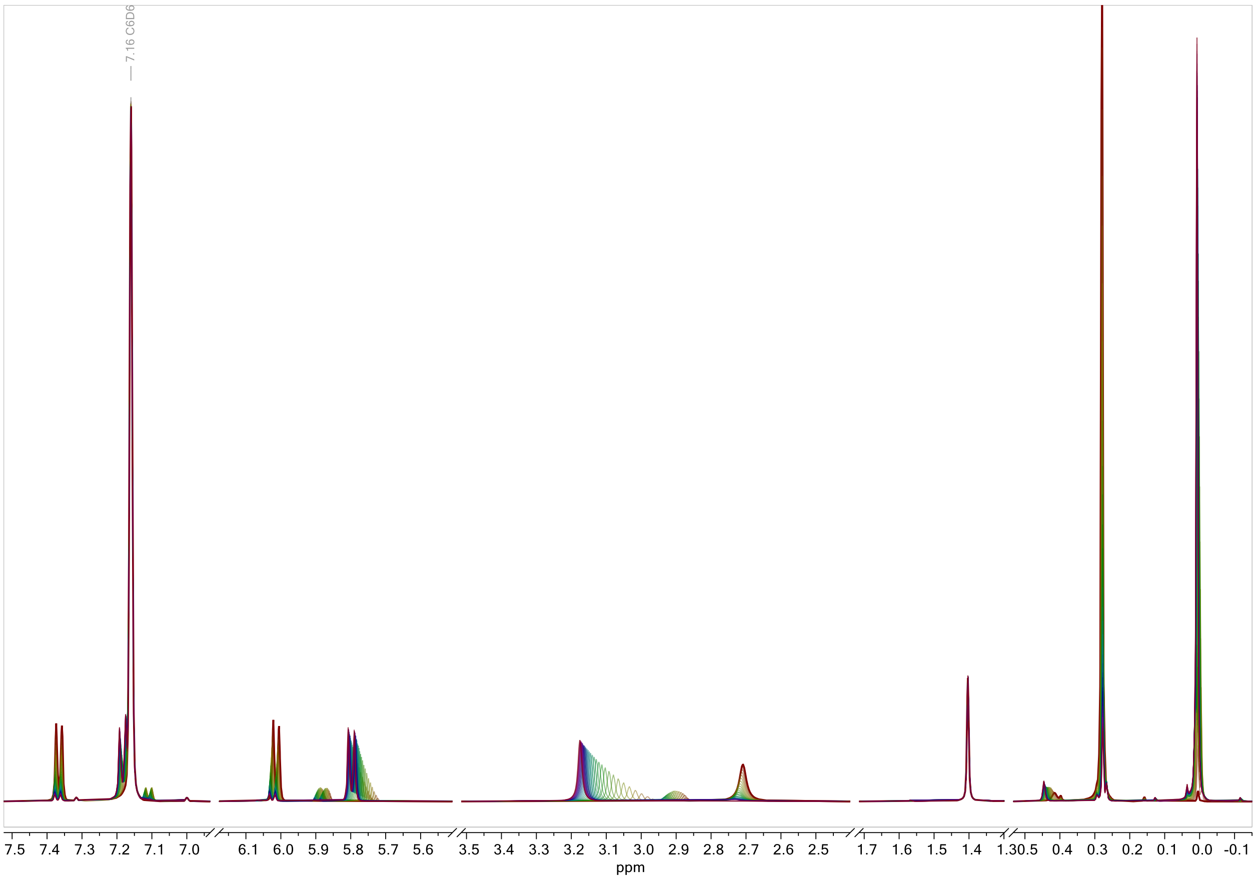

Figure S9.7.1. ^{1}H-NMR spectra (500 MHz, C_6D_6; selected regions shown) corresponding to entry SI-23. Superimposed spectrum of all ^{1}H NMR spectra recorded during the experiment. The first spectrum recorded is colored red and the last spectrum recorded is colored purple. The spectra are recorded precisely every 20 minutes for 15 hours. Only the regions with signals are shown; intermediate regions were removed as indicated by the slashes. The signal at 1.4 ppm corresponds to cyclohexane which was used as an internal standard.

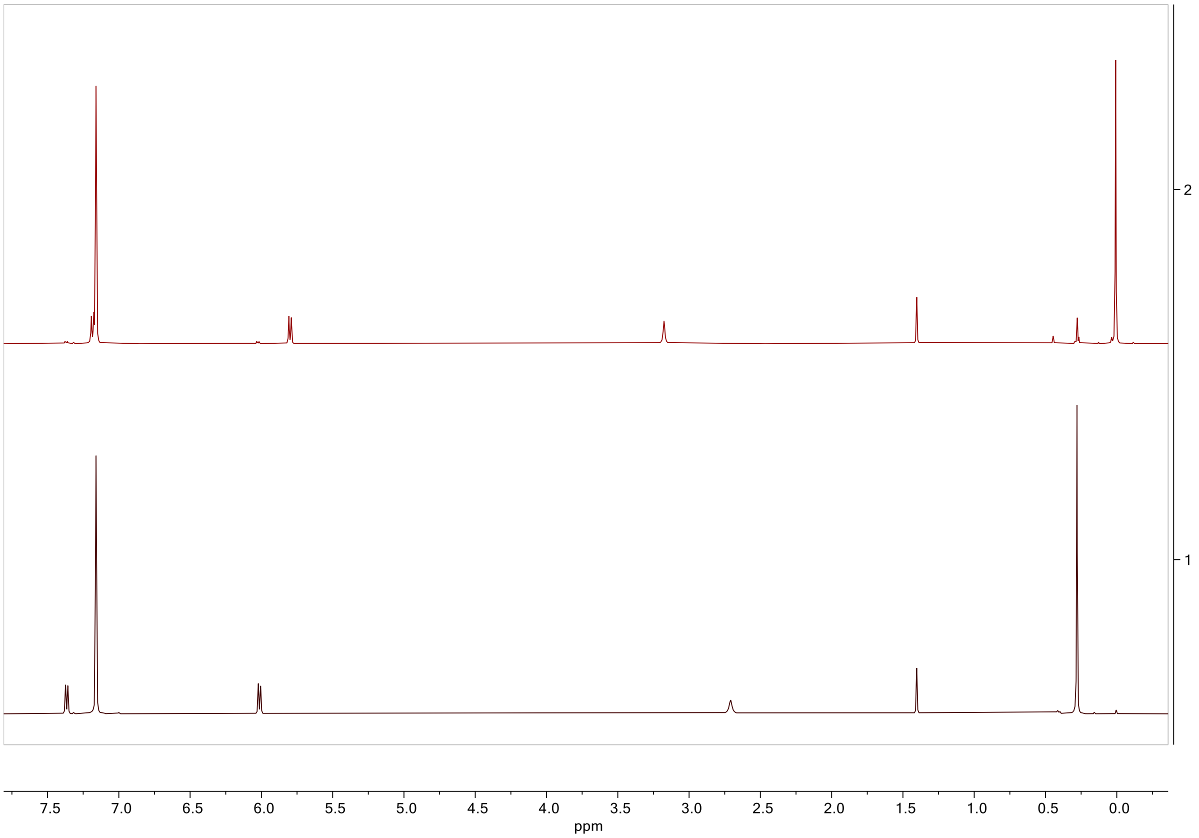

Figure S9.7.2. ^{1}H-NMR spectra (500 MHz, C_6D_6) corresponding to entry SI-23. Bottom spectrum was recorded at start of the reaction, and top spectrum was recorded after 15 hours of reaction time. The signal at 1.4 ppm corresponds to cyclohexane which was used as an internal standard.

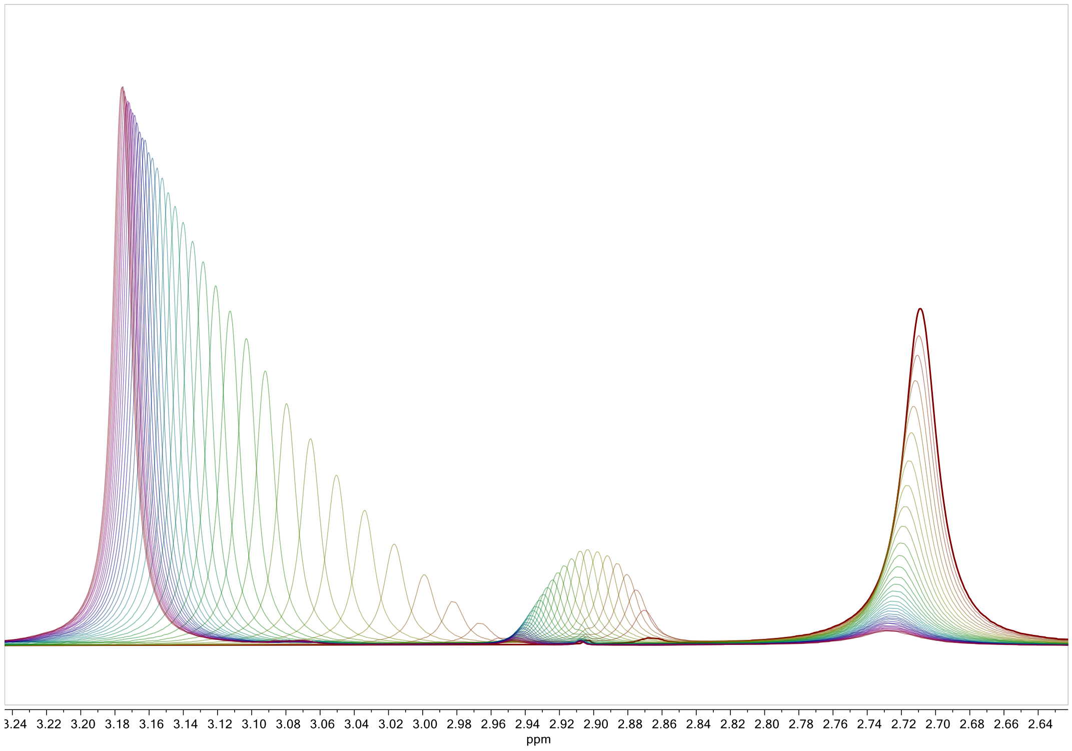

Figure S9.7.3. ^{1}H-NMR spectra (500 MHz, C_6D_6) Selected region of Figure S9.7.1 corresponding to the NH_2 protons showing the change in chemical shift over the duration of the experiment.

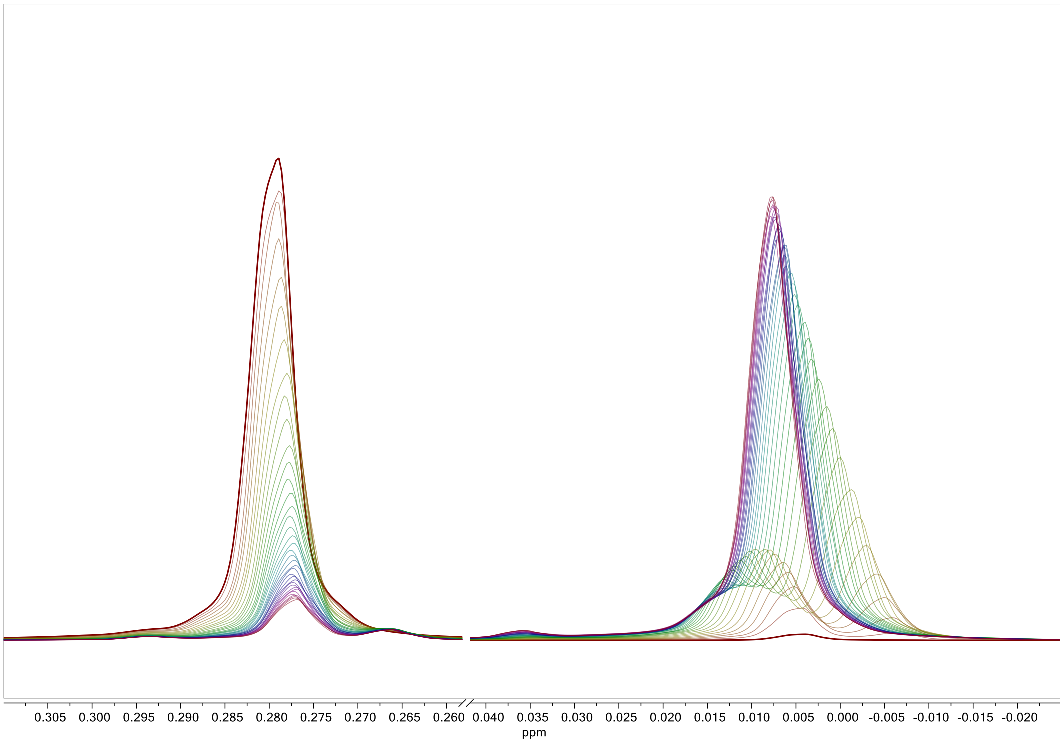

Figure S9.7.4. ^{1}H-NMR spectra (500 MHz, C_6D_6) Selected region of Figure S9.7.1 corresponding to the trimethylsilyl protons showing the change in chemical shift over the duration of the experiment.

Section 9.8 – Entry SI-23X

Reaction between **A** (R = *p*-NH_2) and **B** in C_6D_6. The ratio of **A:B** is 1:1. Concentration of **A** and **B** = 0.030 M. This experiment is identical to that of Entry SI-23 but was conducted to investigate the influence of a spinning sample vs a non-spinning sample. This experiment was run **without** spinning and was conducted in parallel to Entry SI-23Y.

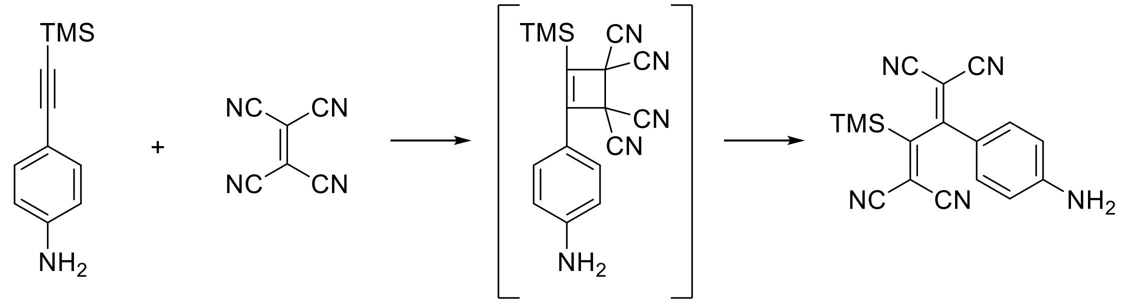

A **B** **C** **P**

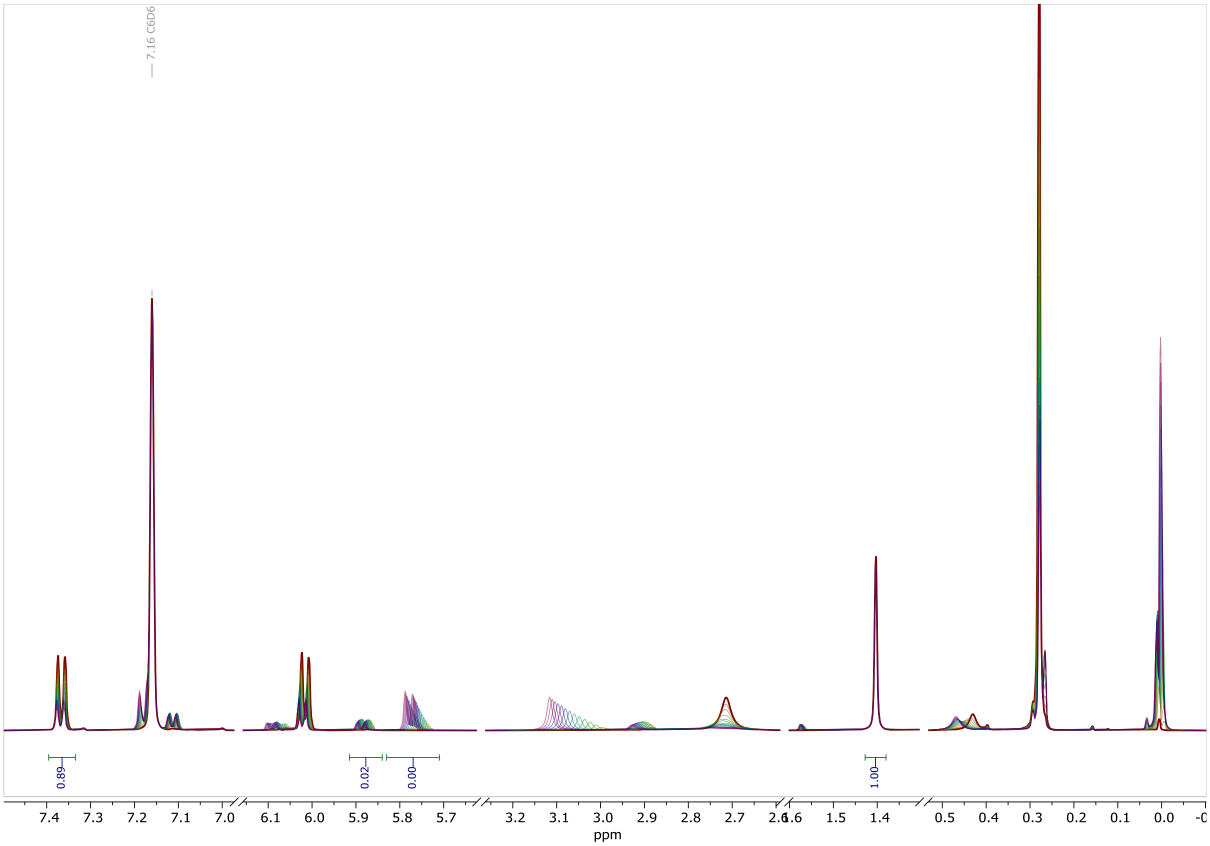

Figure S9.8.1. ^{1}H-NMR spectra (500 MHz, C_6D_6; selected regions shown) corresponding to entry SI-23X. Superimposed spectra of all ^{1}H NMR spectra recorded during the experiment. The first spectrum recorded is colored red and the last spectrum recorded is colored purple. The spectra are recorded precisely every 20 minutes for 5 hours and 20 minutes. Only the regions with signals are shown; intermediate regions were removed as indicated by the slashes. The signal at 1.4 ppm corresponds to cyclohexane which was used as an internal standard.

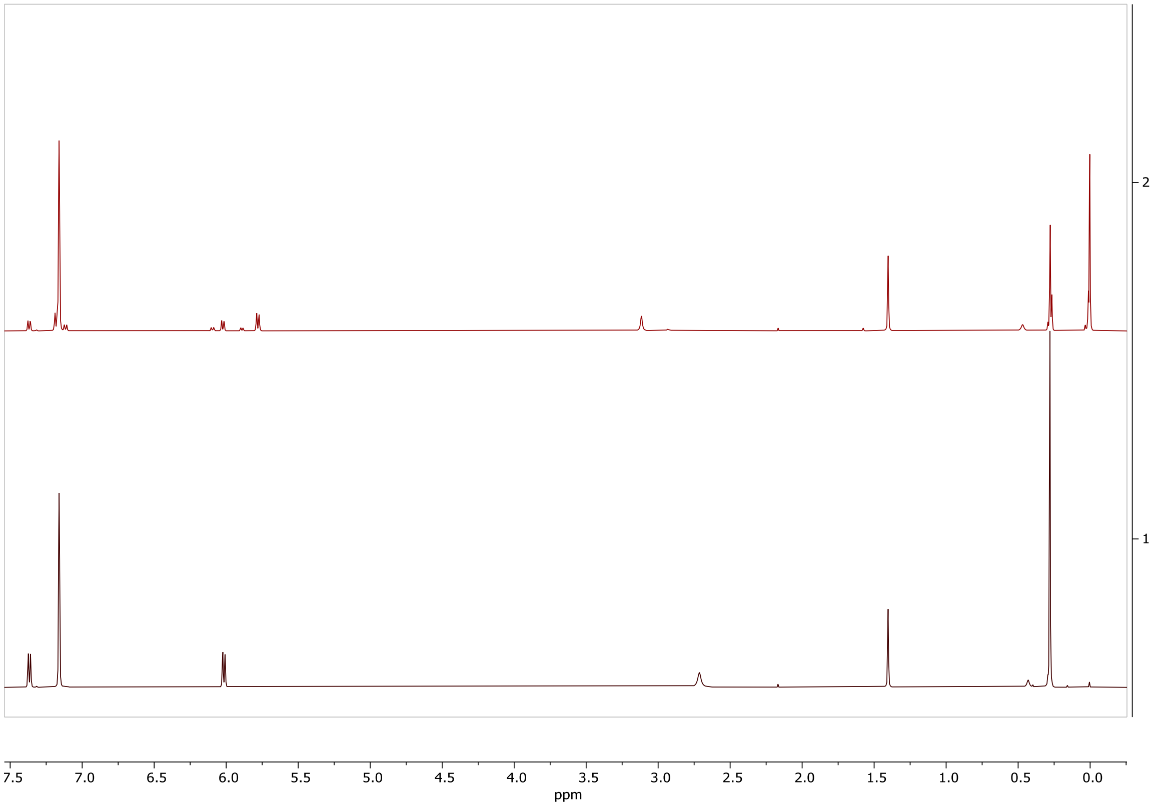

Figure S9.8.2. ^{1}H-NMR spectra (500 MHz, C_6D_6) corresponding to entry SI-23X. Bottom spectrum was recorded at start of the reaction, and top spectrum was recorded after 5 hours and 20 minutes of reaction time. The signal at 1.4 ppm corresponds to cyclohexane which was used as an internal standard.

Section 9.9 – Entry SI-23Y

Reaction between **A** (R = *p*-NH_2) and **B** in C_6D_6. The ratio of **A:B** is 1:1. Concentration of **A** and **B** = 0.030 M. This experiment is identical to that of Entry SI-23 and SI-23X but was conducted to investigate the influence of a spinning sample vs a non-spinning sample. This experiment was run **with** spinning and was conducted in parallel to Entry SI-23X.

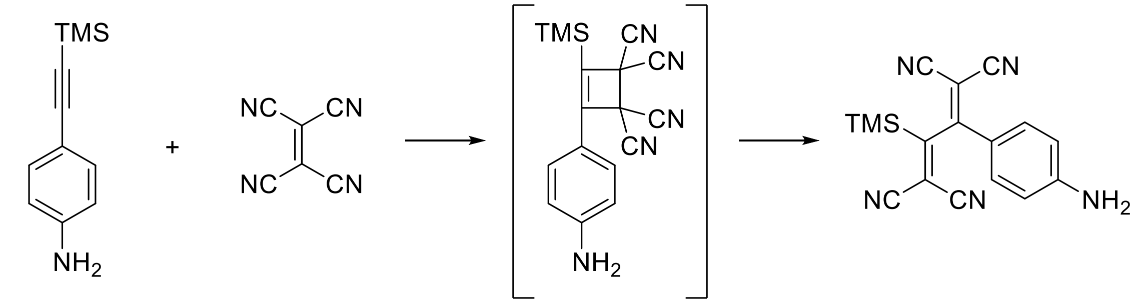

A **B** **C** **P**

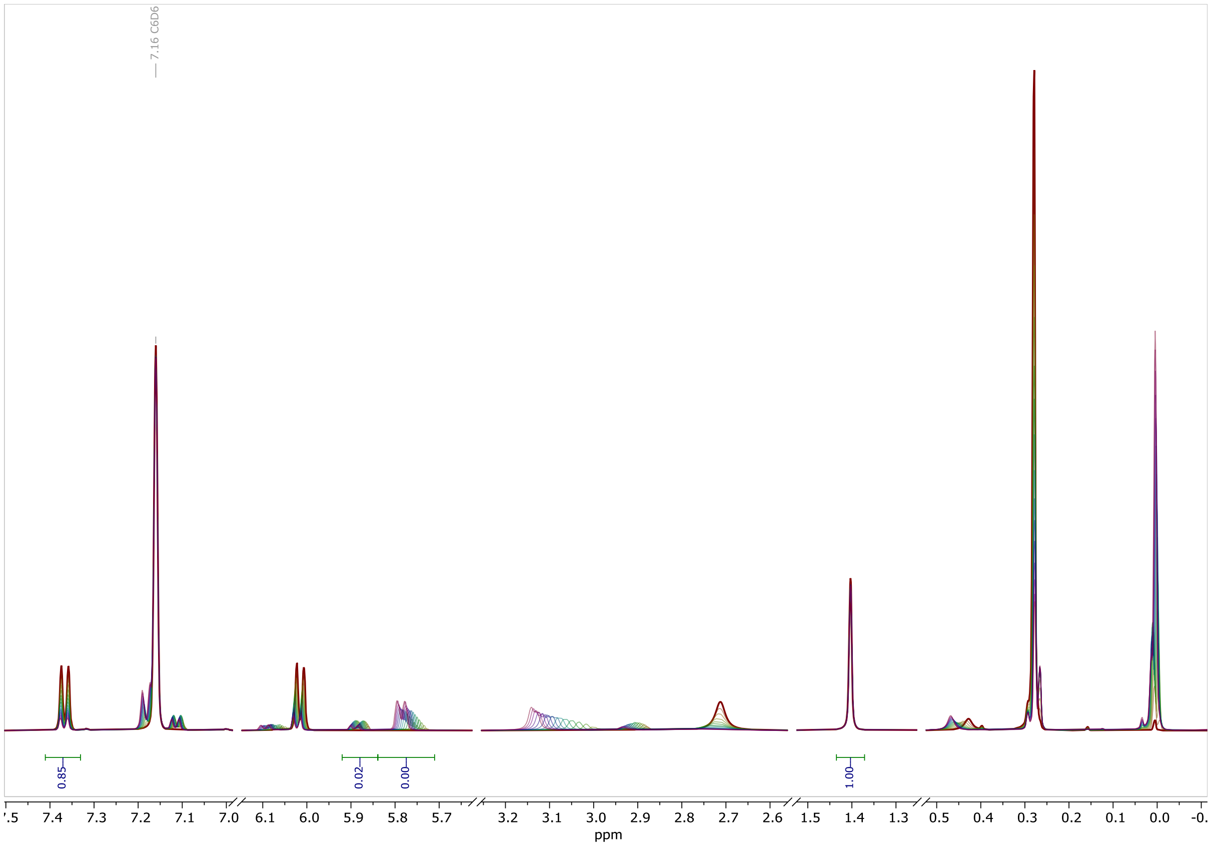

Figure S9.9.1. ^1H-NMR spectra (500 MHz, C_6D_6; selected regions shown) corresponding to entry SI-23Y. Superimposed spectra of all ^1H NMR spectra recorded during the experiment. The first spectrum recorded is colored red and the last spectrum recorded is colored purple. The spectra are recorded precisely every 20 minutes for 6 hours and 20 minutes. Only the regions with signals are shown; intermediate regions were removed as indicated by the slashes. The signal at 1.4 ppm corresponds to cyclohexane which was used as an internal standard.

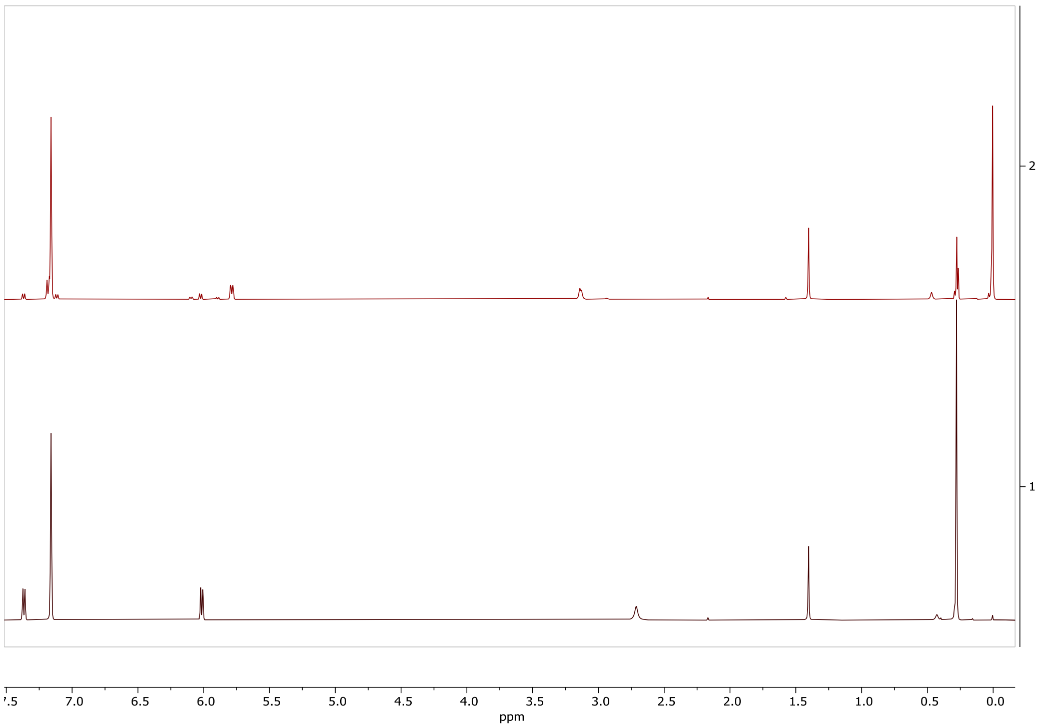

Figure S9.9.2. ^{1}H-NMR spectra (500 MHz, C_6D_6) corresponding to entry SI-23Y. Bottom spectrum was recorded at start of the reaction, and top spectrum was recorded after 6 hours and 20 minutes of reaction time. The signal at 1.4 ppm corresponds to cyclohexane which was used as an internal standard.

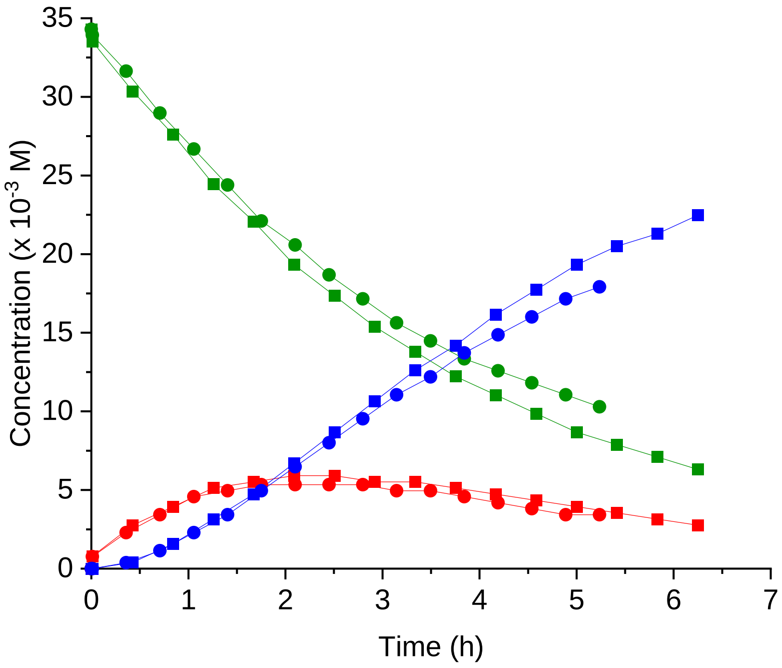

Figure S9.9.3. Concentration of species against time. Experiment SI-23X corresponds to circle data points and SI-23Y corresponds to square data point. There is no noticeable difference between the two experiments.

Section 9.10 – Entry SI-24

Reaction between **A** (R = *p*-NH_2) and **B** in C_6D_6. The ratio of **A:B** is 1:2. Concentration of **A** = 0.0315 M. This experiment is identical to that of entries SI-23 and SI-24 but with more equivalents of **B**.

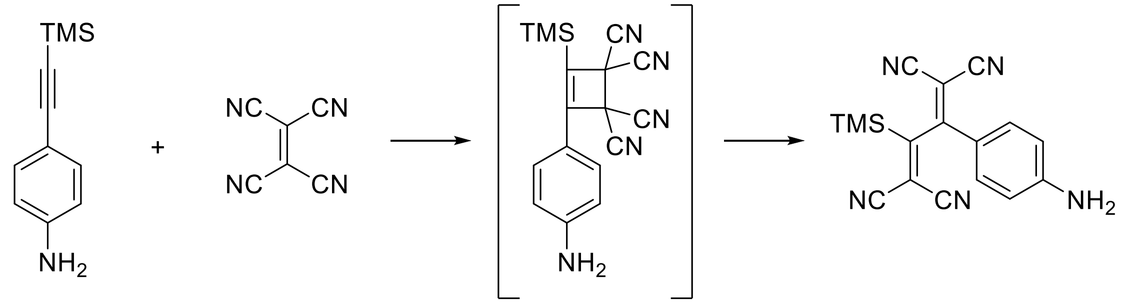

A **B** **C** **P**

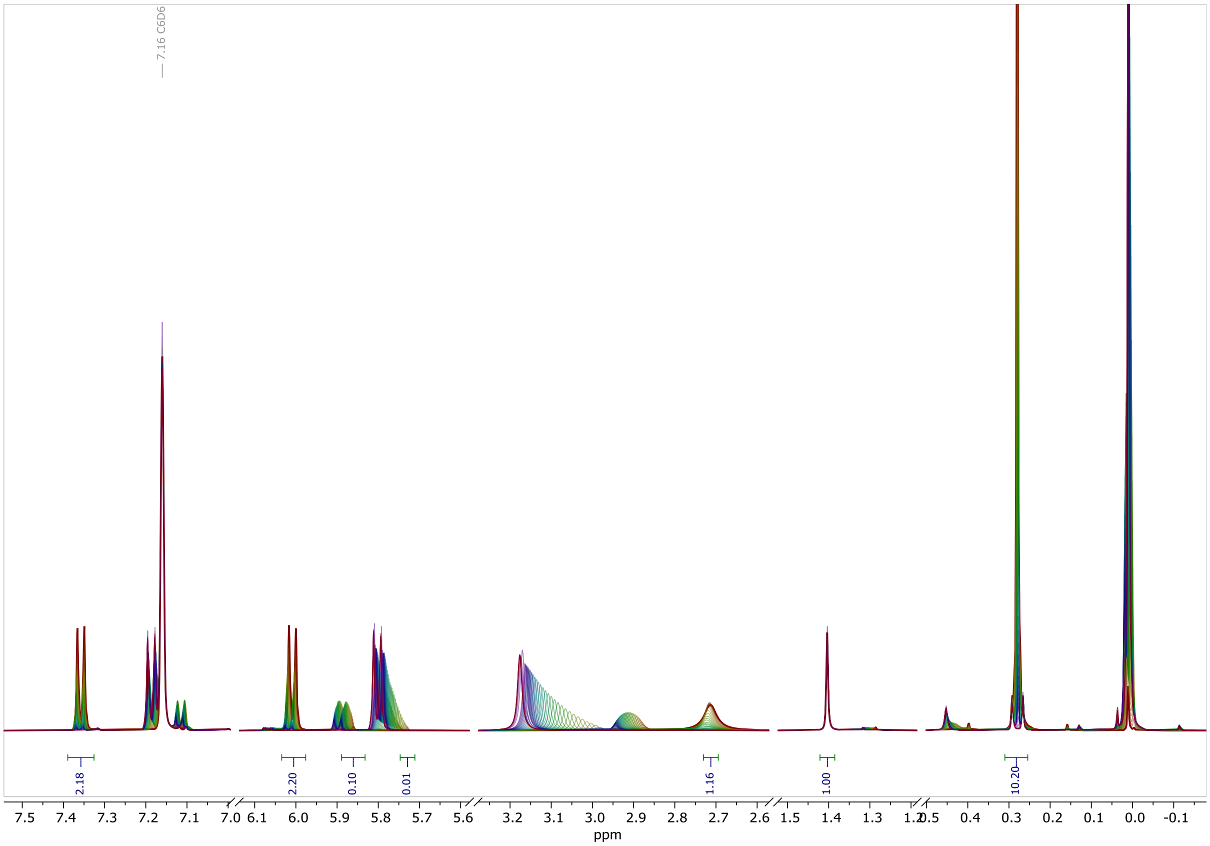

Figure S9.10.1. ^{1}H-NMR spectra (500 MHz, C_6D_6; selected regions shown) corresponding to entry SI-24. Superimposed spectra of all ^{1}H NMR spectra recorded during the experiment. The first spectrum recorded is colored red and the last spectrum recorded is colored purple. The spectra are recorded precisely every 10 minutes for the first 5 hours and 20 minutes and every 50 min for the remainder of the experiment. Only the regions with signals are shown; intermediate regions were removed as indicated by the slashes. The signal at 1.4 ppm corresponds to cyclohexane which was used as an internal standard.

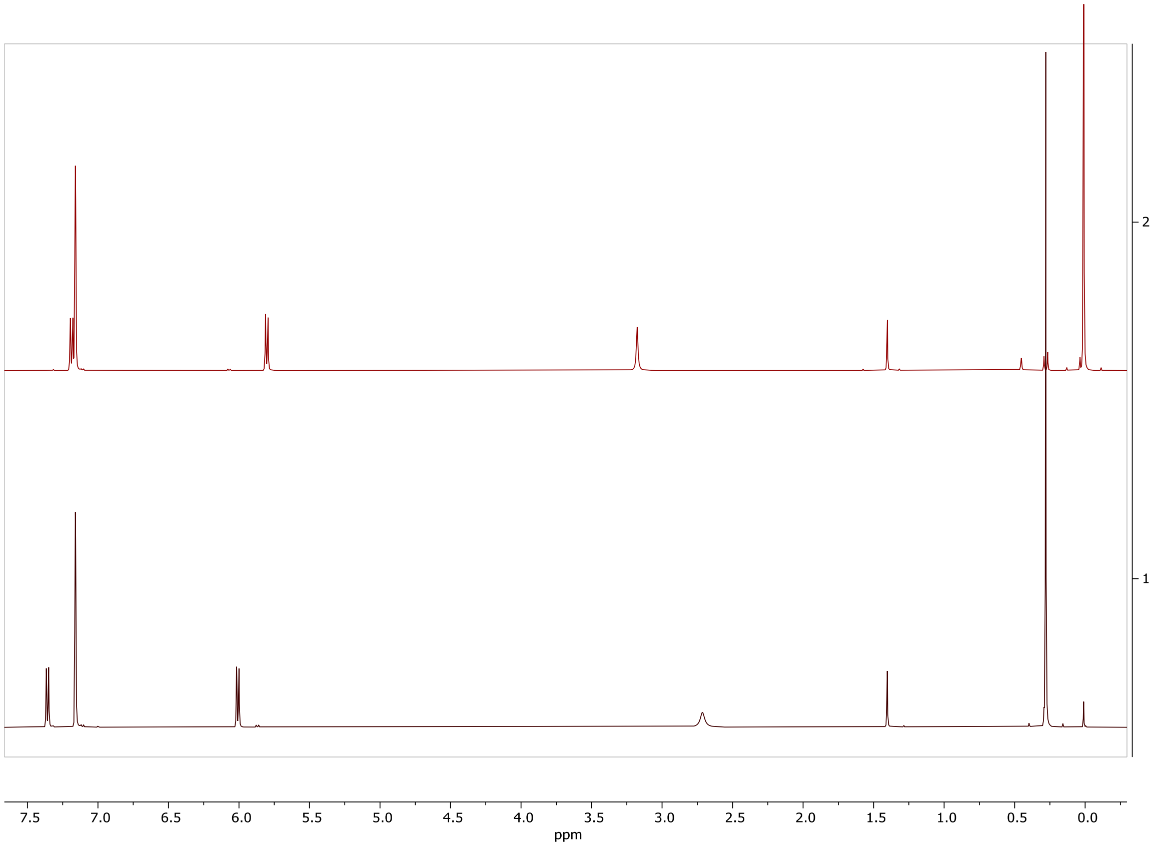

Figure S9.10.2. ^{1}H-NMR spectra (500 MHz, C_6D_6) corresponding to entry SI-24. Bottom spectrum was recorded at start of the reaction, and top spectrum was recorded after 12 hours and 50 minutes of reaction time. The signal at 1.4 ppm corresponds to cyclohexane which was used as an internal standard.

Section 9.11 – Entry SI-25

Reaction between **A** (R = *p*-NH_2) and **B** in C_6D_6. The ratio of **A:B** is 1:5. Concentration of **A** = 0.0295 M. This experiment is identical to that of entries SI-23 but with even more equivalents of **B**.

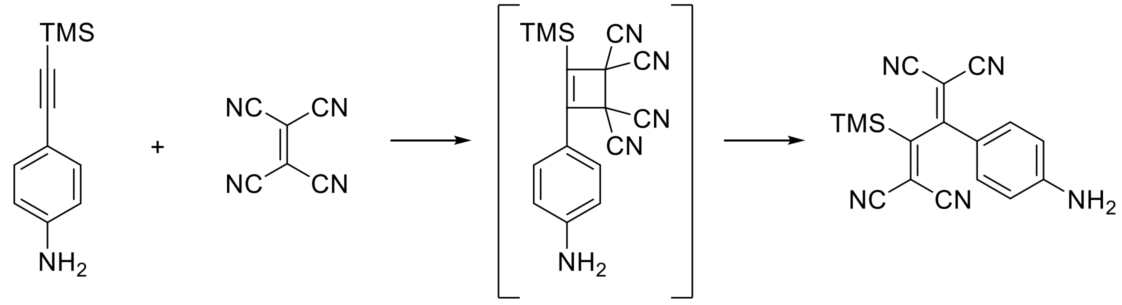

A **B** **C** **P**

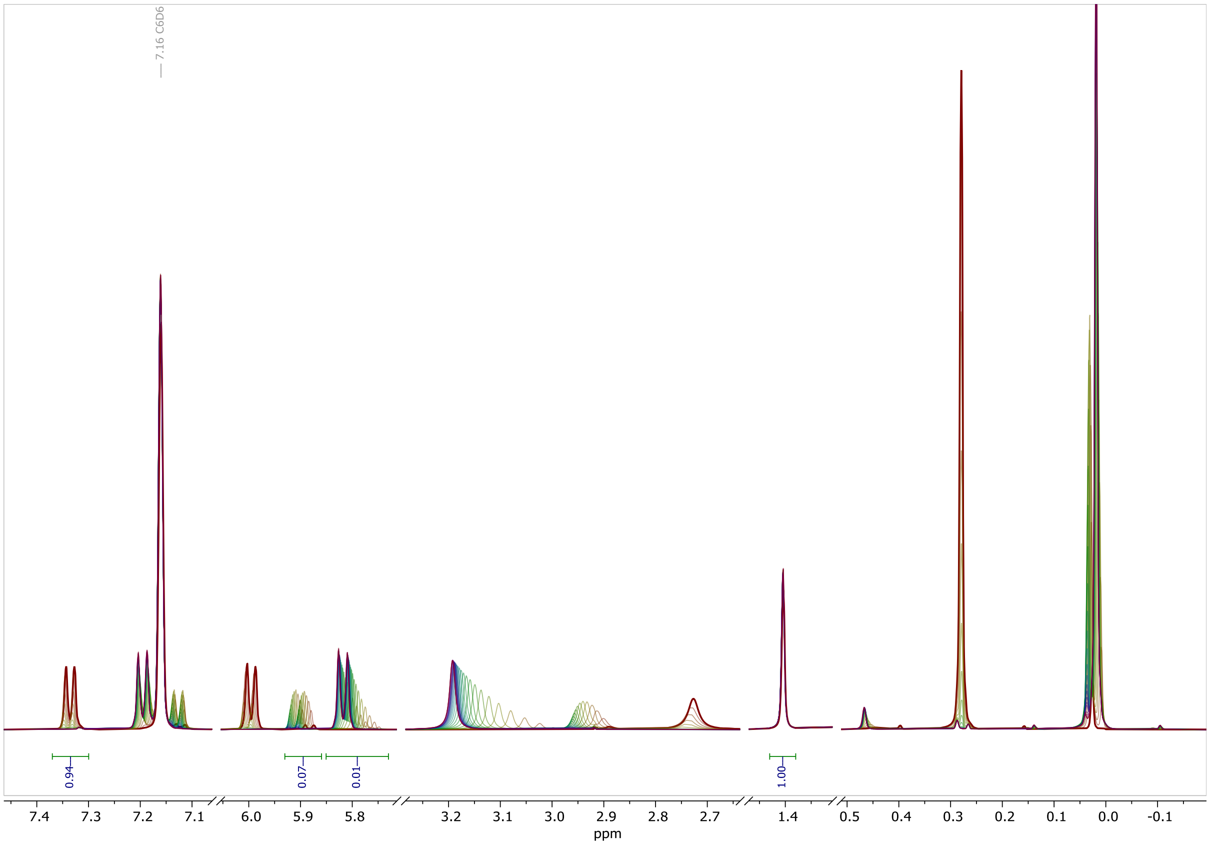

Figure S9.11.1. ^1H-NMR spectra (500 MHz, C_6D_6; selected regions shown) corresponding to entry SI-25. Superimposed spectra of all ^1H NMR spectra recorded during the experiment. The first spectrum recorded is colored red and the last spectrum recorded is colored purple. The spectra are recorded precisely every 12 minutes for the first 5 hours and 30 minutes and every 40 min for 2 hours and then every 70 minutes for the remaining time of the experiment. Only the regions with signals are shown; intermediate regions were removed as indicated by the slashes. The signal at 1.4 ppm corresponds to cyclohexane which was used as an internal standard.

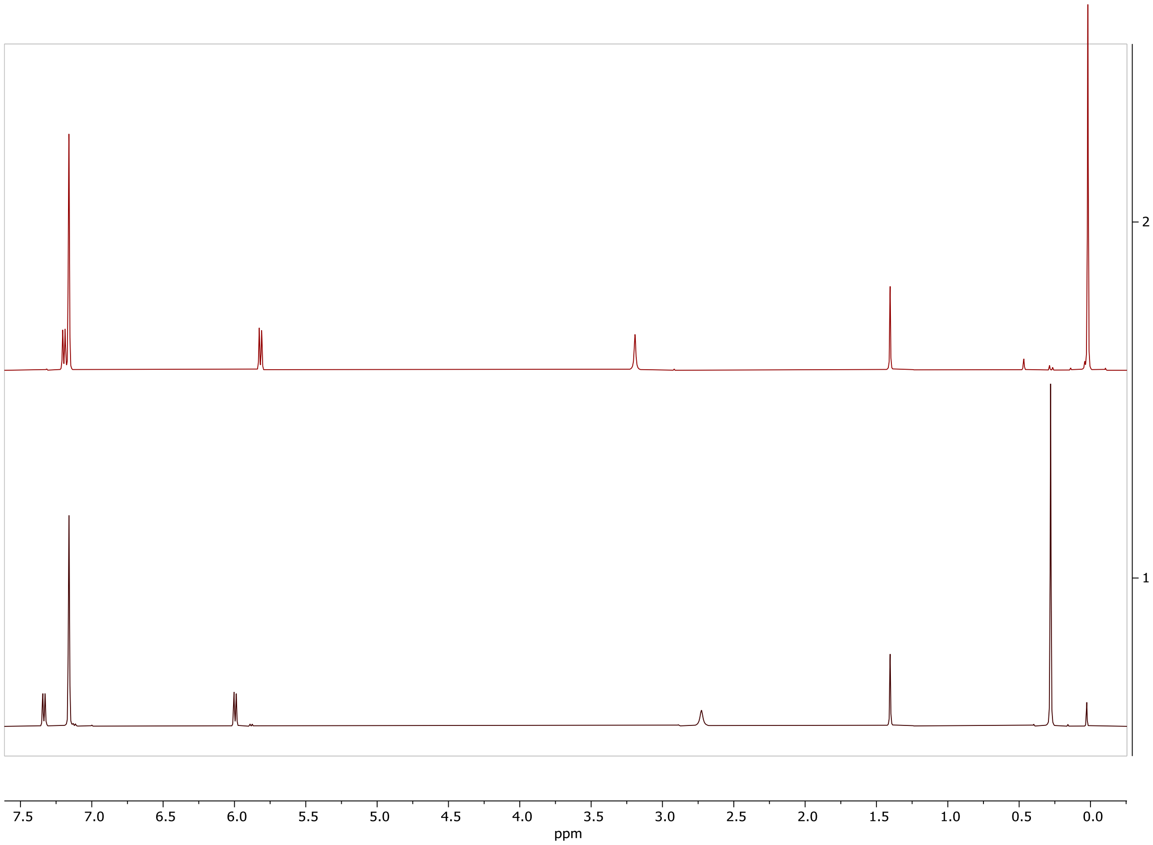

Figure S9.11.2. ^{1}H-NMR spectra (500 MHz, C_6D_6) corresponding to entry SI-25. Bottom spectrum was recorded at start of the reaction, and top spectrum was recorded after 11 hours and 30 minutes of reaction time. The signal at 1.4 ppm corresponds to cyclohexane which was used as an internal standard.

Section 9.12 – Entry SI-26

Reaction between **A** (R = *p*-NH_2) and **B** in C_6D_6. The ratio of **A:B** is 1:5. Concentration of **A** = 0.021 M. This experiment measured the ^{29}Si-nucleus.

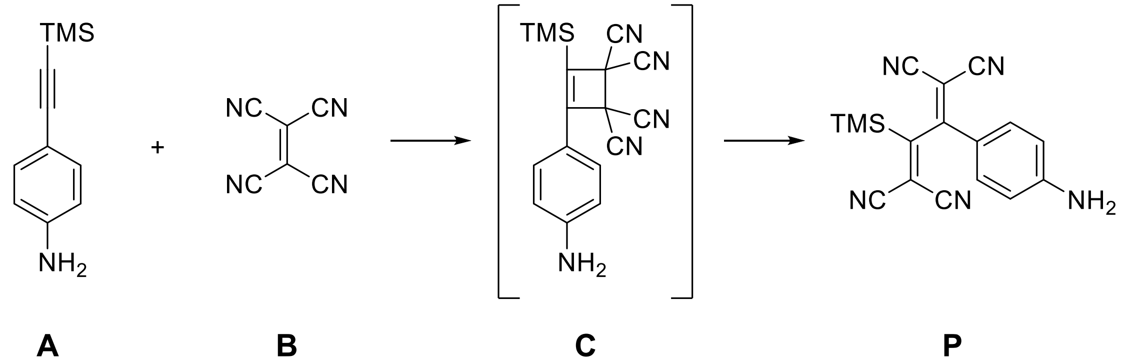

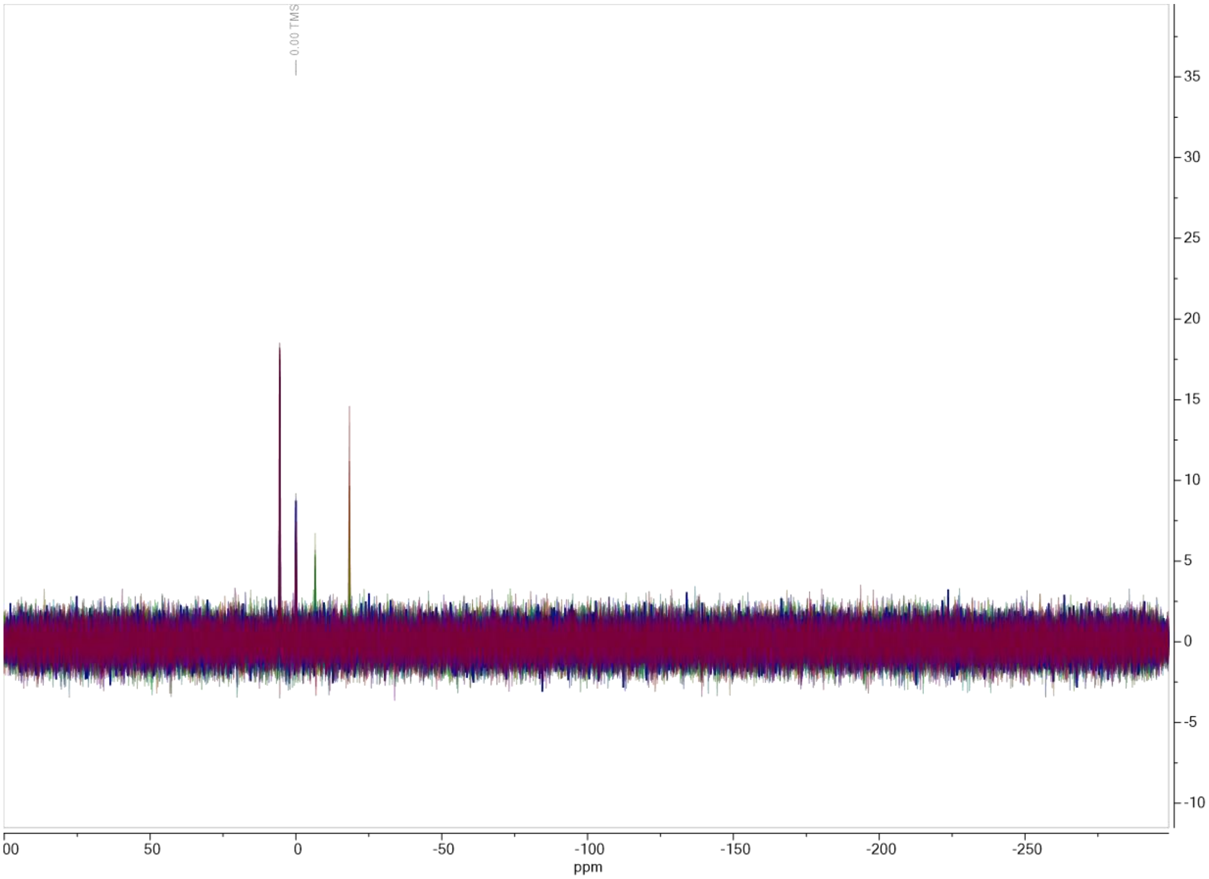

Figure S9.12.1. ^{29}Si-NMR spectra (500 MHz, C_6D_6) corresponding to entry SI-26. Superimposed spectra of all ^{29}Si NMR spectra recorded during the experiment. The spectra are recorded precisely every 15 minutes for the first 3 hours and 15 minutes and every 30 min for 6 hours and 30 min. The signal at 0.0 ppm corresponds to tetramethylsilane which was used as an internal standard.

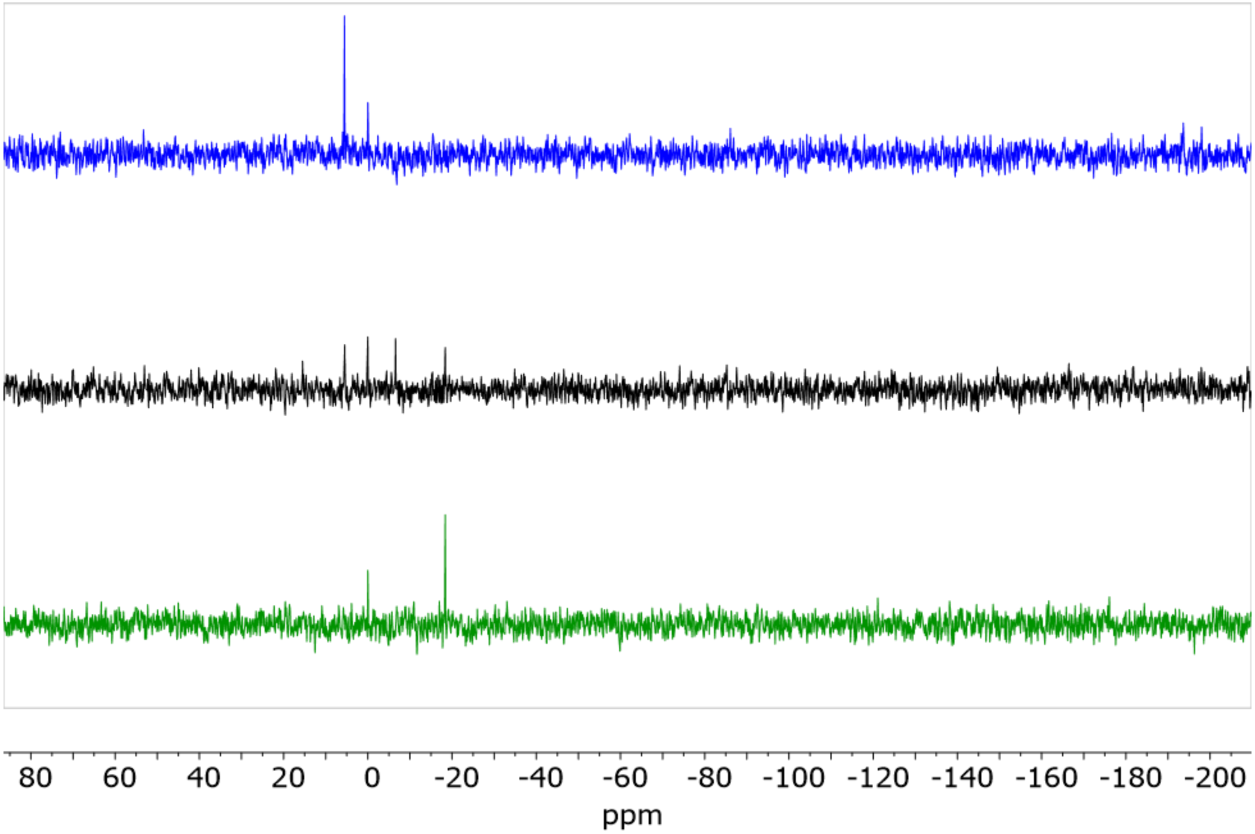

Figure S9.12.2. ^{29}Si-NMR spectra (500 MHz, C_6D_6) corresponding to entry SI-26. Bottom spectrum was recorded at start of the reaction, middle spectrum after 1 hour, and top spectrum was recorded after 9 hours and 30 minutes of reaction time. The signal at 0.0 ppm corresponds to tetramethylsilane which was used as an internal standard.

Section 9.13 – Entry SI-27

Reaction between **A** (R = *p*-NH_2) and **B** in C_6D_6. The ratio of **A:B** is 1:1. Concentration of **A** and **B** = 0.052 M. This experiment was conducted to investigate the influence of water present by saturating the solvent with water before it was used.

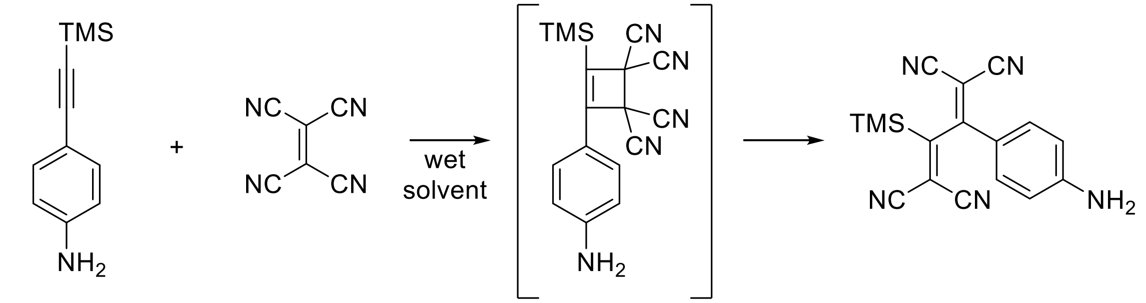

A **B** **C** **P**

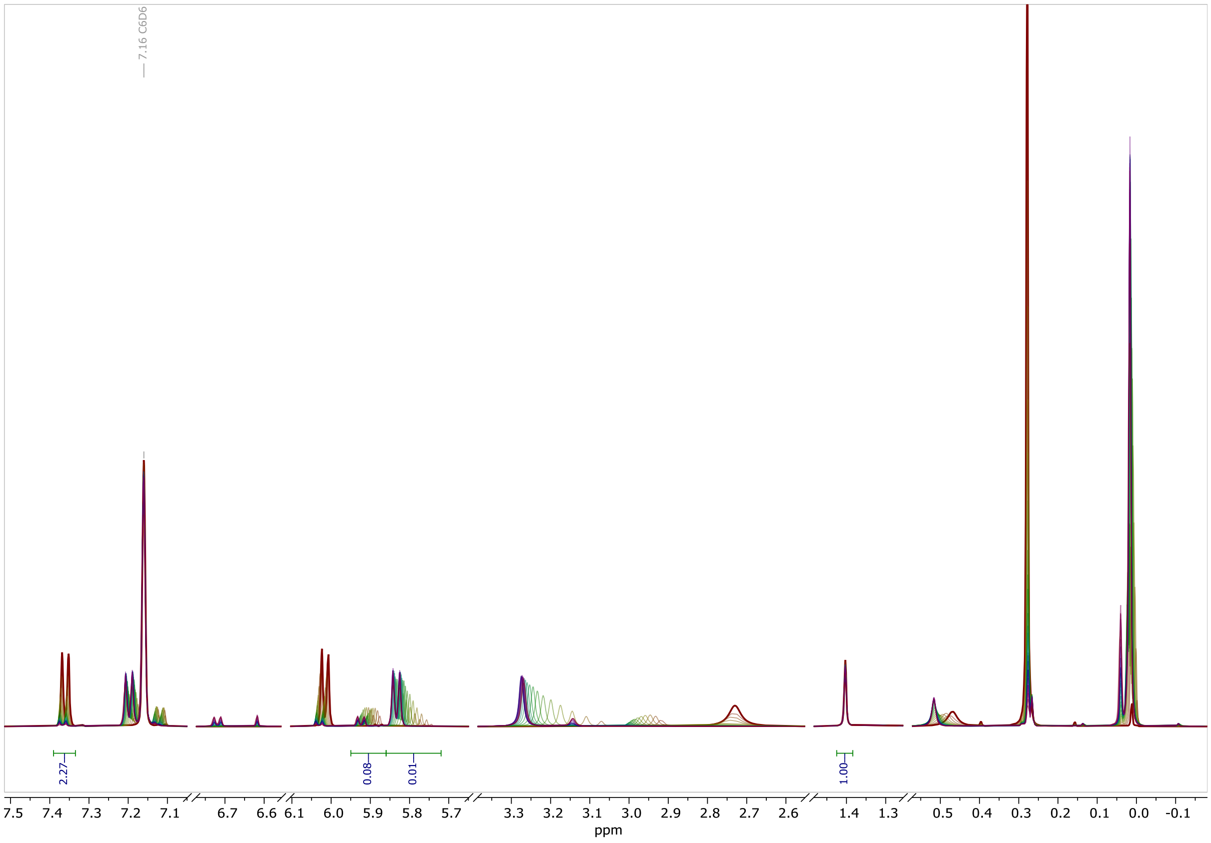

Figure S9.13.1. ^{1}H-NMR spectra (500 MHz, C_6D_6; selected regions shown) corresponding to entry SI-27. Superimposed spectra of all ^{1}H NMR spectra recorded during the experiment. The first spectrum recorded is colored red and the last spectrum recorded is colored purple. The spectra are recorded precisely every 25 minutes for the first 7 hours and 30 minutes and every 60 min for the remaining time of the experiment. Only the regions with signals are shown; intermediate regions were removed as indicated by the slashes. The signal at 1.4 ppm corresponds to cyclohexane which was used as an internal standard.

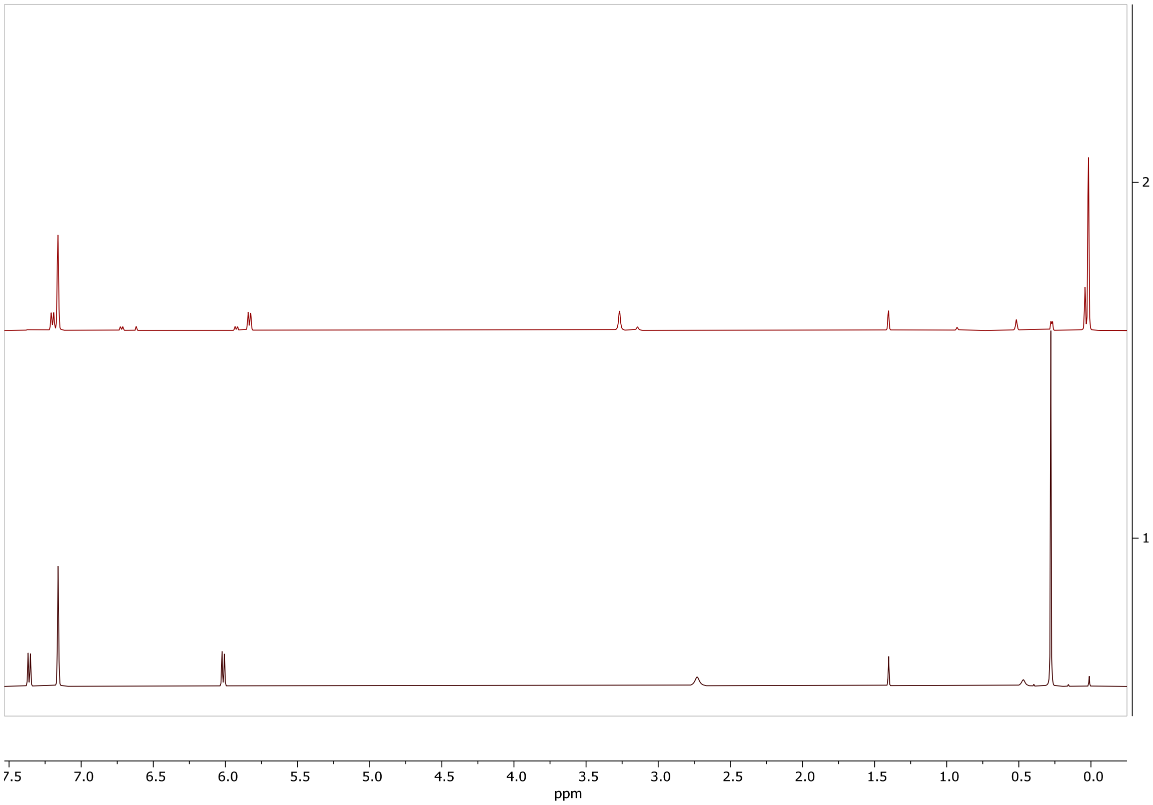

Figure S9.13.2. ^{1}H-NMR spectra (500 MHz, C_6D_6) corresponding to entry SI-27. Bottom spectrum was recorded at start of the reaction, and top spectrum was recorded after 14 hours and 40 minutes of reaction time. The signal at 1.4 ppm corresponds to cyclohexane which was used as an internal standard.

Section 9.14 – Entry SI-28

Reaction between **A** (R = *p*-NH_2) and **B** in C_6D_6. The ratio of **A:B** is 1:2. Concentration of **A** = 0.034 M. This experiment is identical to that of Entry SI-27 but with a different ratio of **A** and **B**. It was conducted to investigate the influence of water present by saturating the solvent with water before it was used.

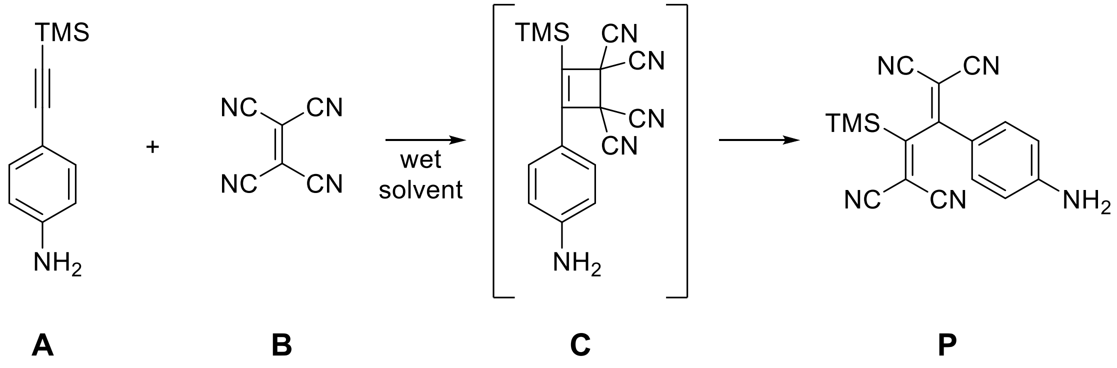

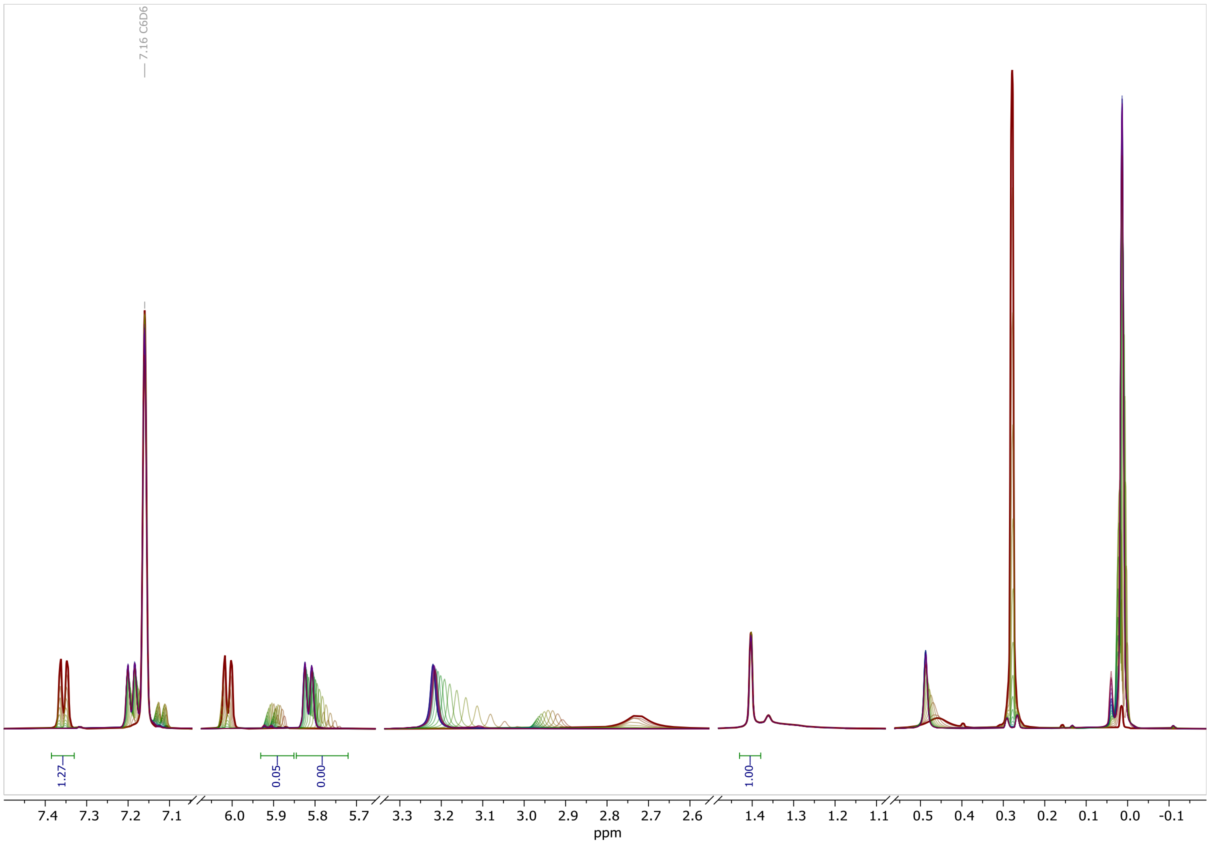

Figure S9.14.1. ^{1}H-NMR spectra (500 MHz, C_6D_6; selected regions shown) corresponding to entry SI-28. Superimposed spectra of all ^{1}H NMR spectra recorded during the experiment. The first spectrum recorded is colored red and the last spectrum recorded is colored purple. The spectra are recorded precisely every 25 minutes for the first 7 hours and 30 minutes and every 60 min for the remaining time of the experiment. Only the regions with signals are shown; intermediate regions were removed as indicated by the slashes. The signal at 1.4 ppm corresponds to cyclohexane which was used as an internal standard.

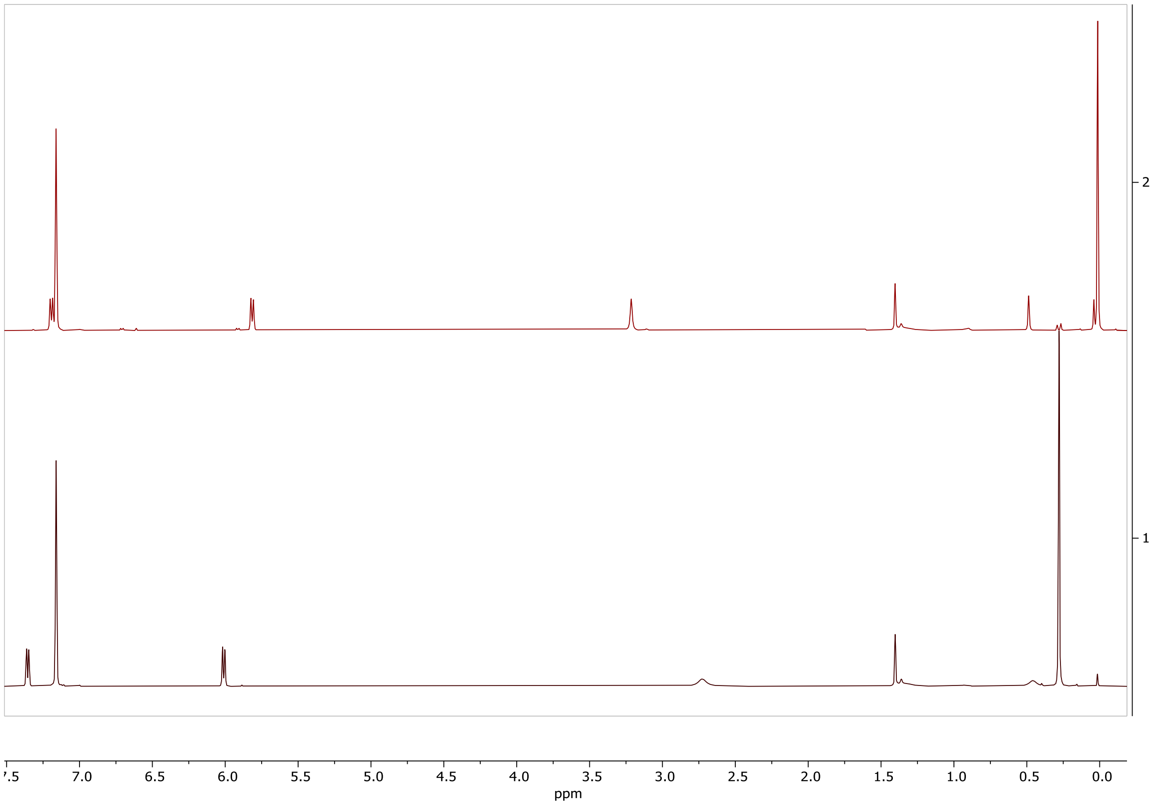

Figure S9.14.2. ^{1}H-NMR spectra (500 MHz, C_6D_6) corresponding to entry SI-28. Bottom spectrum was recorded at start of the reaction, and top spectrum was recorded after 14 hours and 40 minutes of reaction time. The signal at 1.4 ppm corresponds to cyclohexane which was used as an internal standard.

Section 9.15 – Entry SI-29

Reaction between **A** (R = *p*-NH_2) and **B** in C_6D_6. The ratio of **A:B** is 2:1. Concentration of **A** = 0.069 M. This experiment is identical to that of Entry SI-27 and Entry SI-28 but with a different ratio of **A** and **B**. It was conducted to investigate the influence of water present by saturating the solvent with water before it was used.

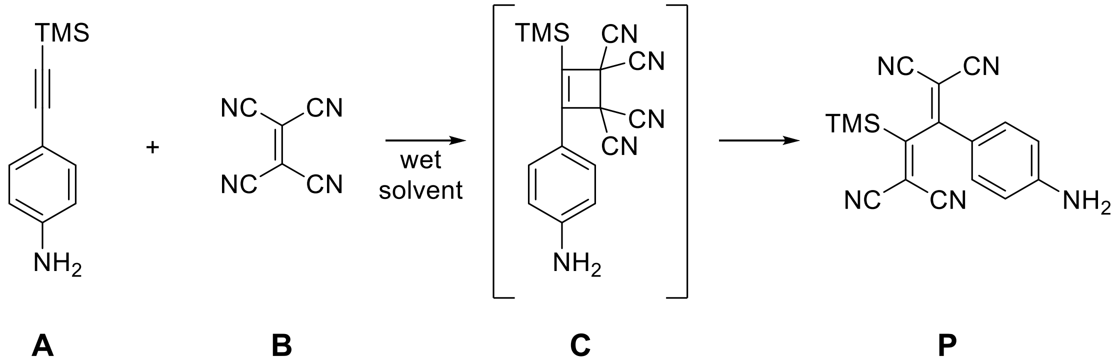

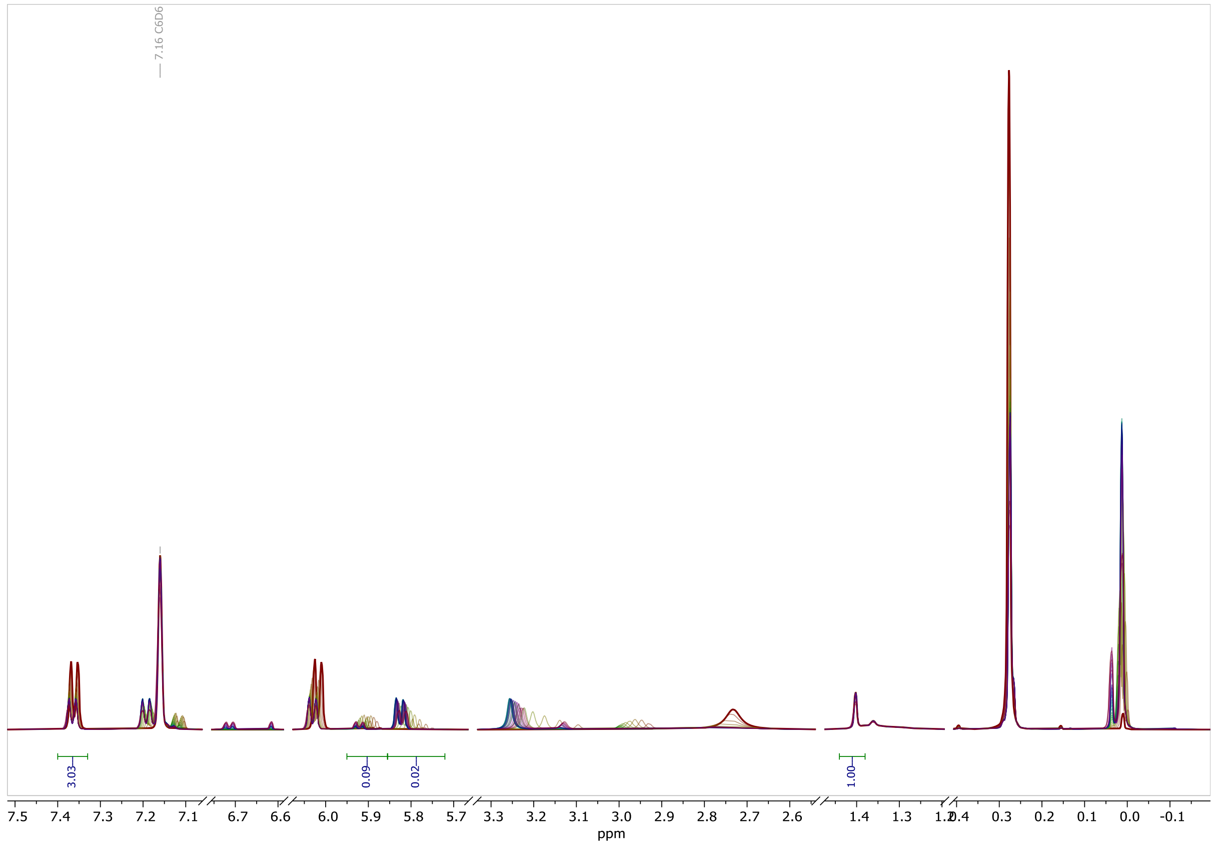

Figure S9.15.1. ^{1}H-NMR spectra (500 MHz, C_6D_6; selected regions shown) corresponding to entry SI-29. Superimposed spectra of all ^{1}H NMR spectra recorded during the experiment. The first spectrum recorded is colored red and the last spectrum recorded is colored purple. The spectra are recorded precisely every 25 minutes for the first 7 hours and 30 minutes and every 60 min for the remaining time of the experiment. Only the regions with signals are shown; intermediate regions were removed as indicated by the slashes. The signal at 1.4 ppm corresponds to cyclohexane which was used as an internal standard.

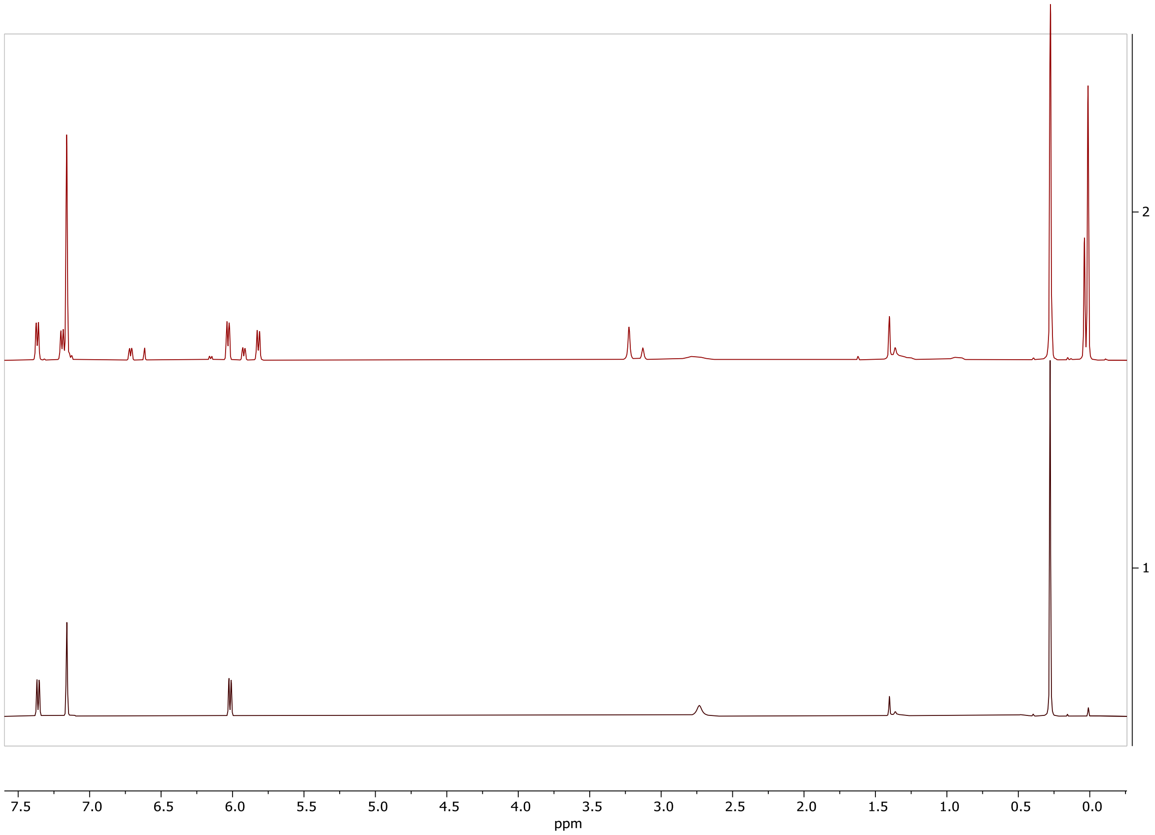

Figure S9.15.2. ^{1}H-NMR spectra (500 MHz, C_6D_6) corresponding to entry SI-29. Bottom spectrum was recorded at start of the reaction, and top spectrum was recorded after 14 hours and 40 minutes of reaction time. The signal at 1.4 ppm corresponds to cyclohexane which was used as an internal standard.

Section 9.16 – Entry SI-30

Reaction between **A** (R = *p*-NH_2) and **B** in C_6D_6. The ratio of **A:B** is 1:1. Concentration of **A** and **B** = 0.052 M. It was conducted to investigate the influence of water present by extra carefully drying the solvent before it was used.

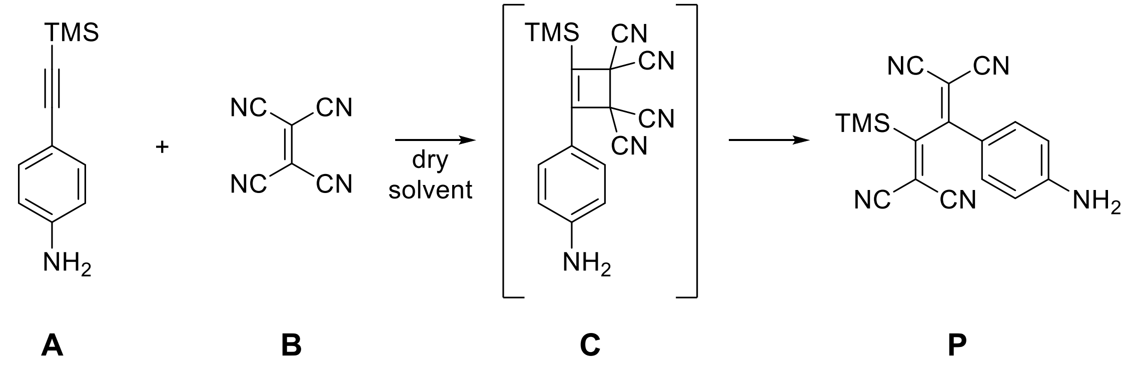

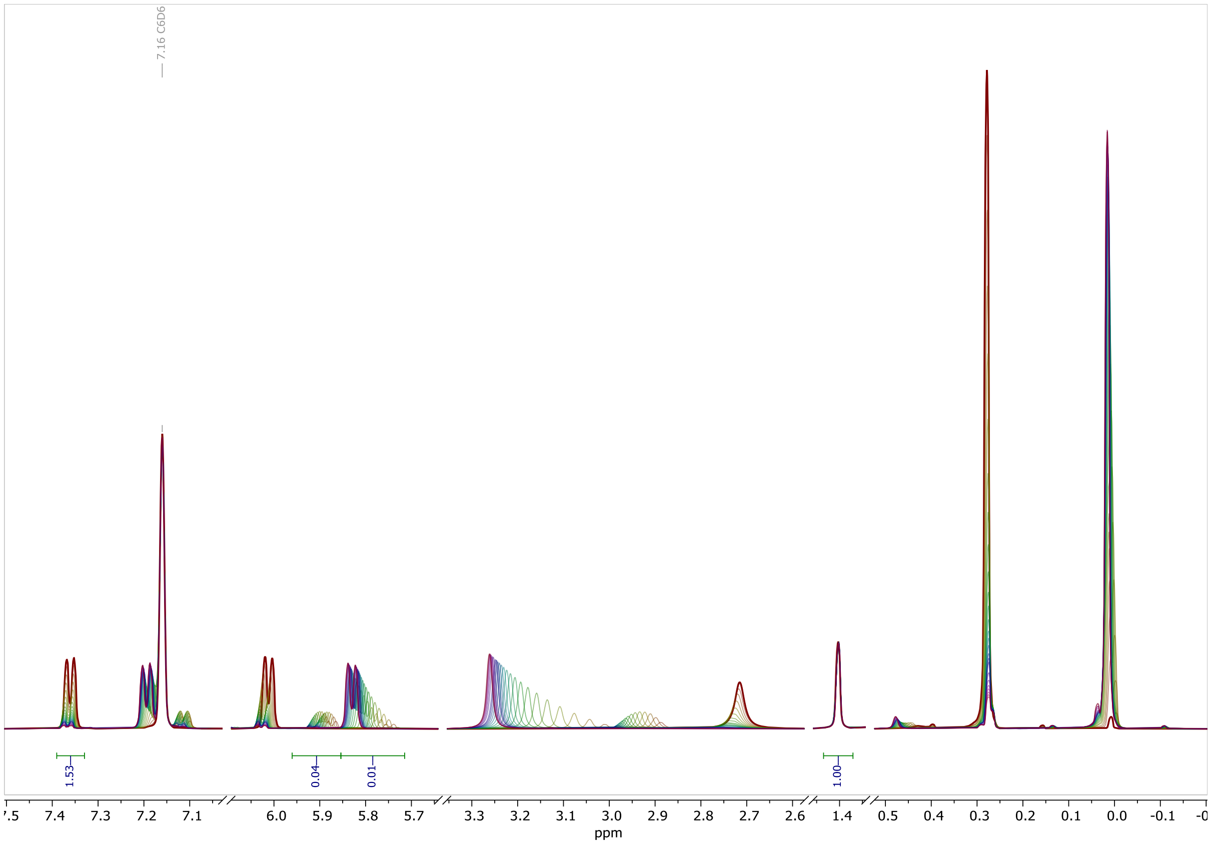

Figure S9.16.1. ^{1}H-NMR spectra (500 MHz, C_6D_6; selected regions shown) corresponding to entry SI-30. Superimposed spectra of all ^{1}H NMR spectra recorded during the experiment. The first spectrum recorded is colored red and the last spectrum recorded is colored purple. The spectra are recorded precisely every 25 minutes for the first 7 hours and 30 minutes and every 60 min for the remaining time of the experiment. Only the regions with signals are shown; intermediate regions were removed as indicated by the slashes. The signal at 1.4 ppm corresponds to cyclohexane which was used as an internal standard.

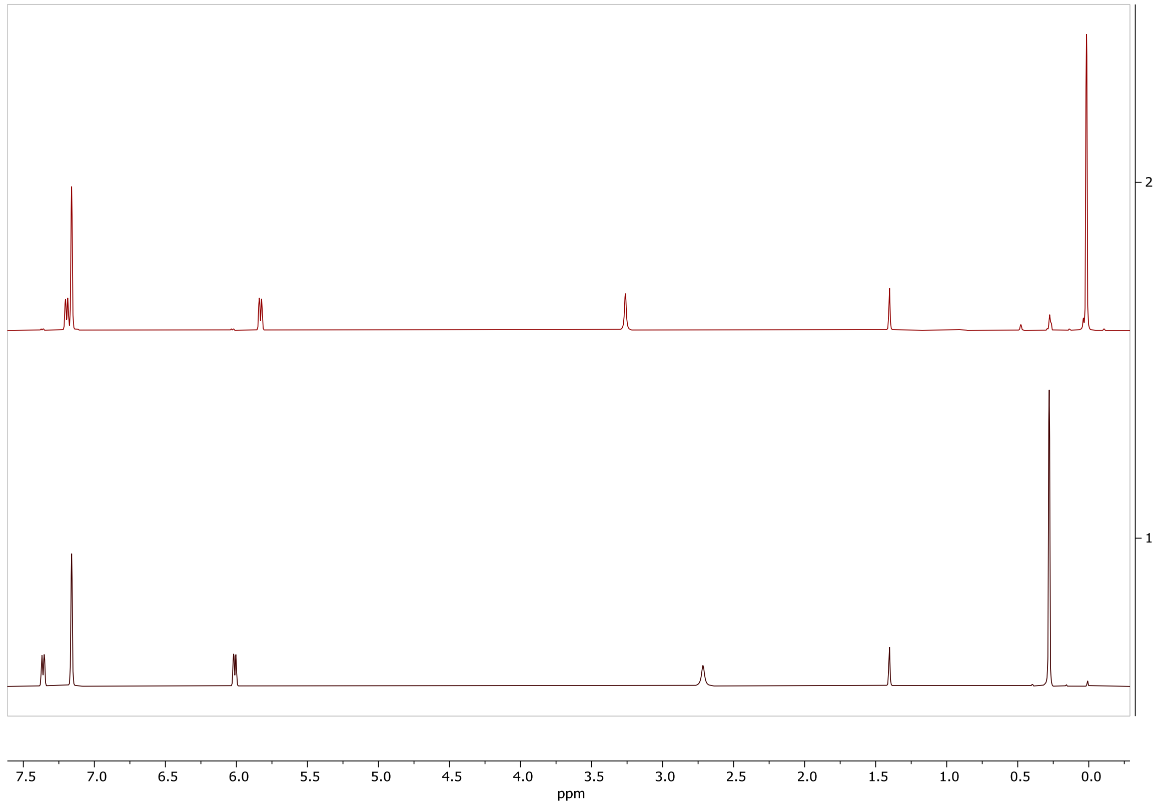

Figure S9.16.2. ^{1}H-NMR spectra (500 MHz, C_6D_6) corresponding to entry SI-30. Bottom spectrum was recorded at start of the reaction, and top spectrum was recorded after 14 hours and 40 minutes of reaction time. The signal at 1.4 ppm corresponds to cyclohexane which was used as an internal standard.

Section 9.17 – Entry SI-31

Reaction between **A** (R = *p*-NH_2) and **B** in C_6D_6. The ratio of **A:B** is 1:2. Concentration of **A** = 0.034 M. It was conducted to investigate the influence of water present by extra carefully drying the solvent before it was used.

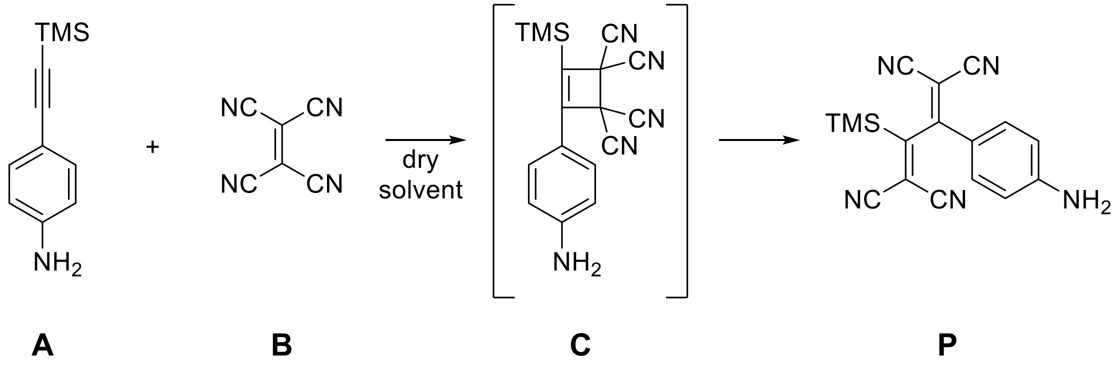

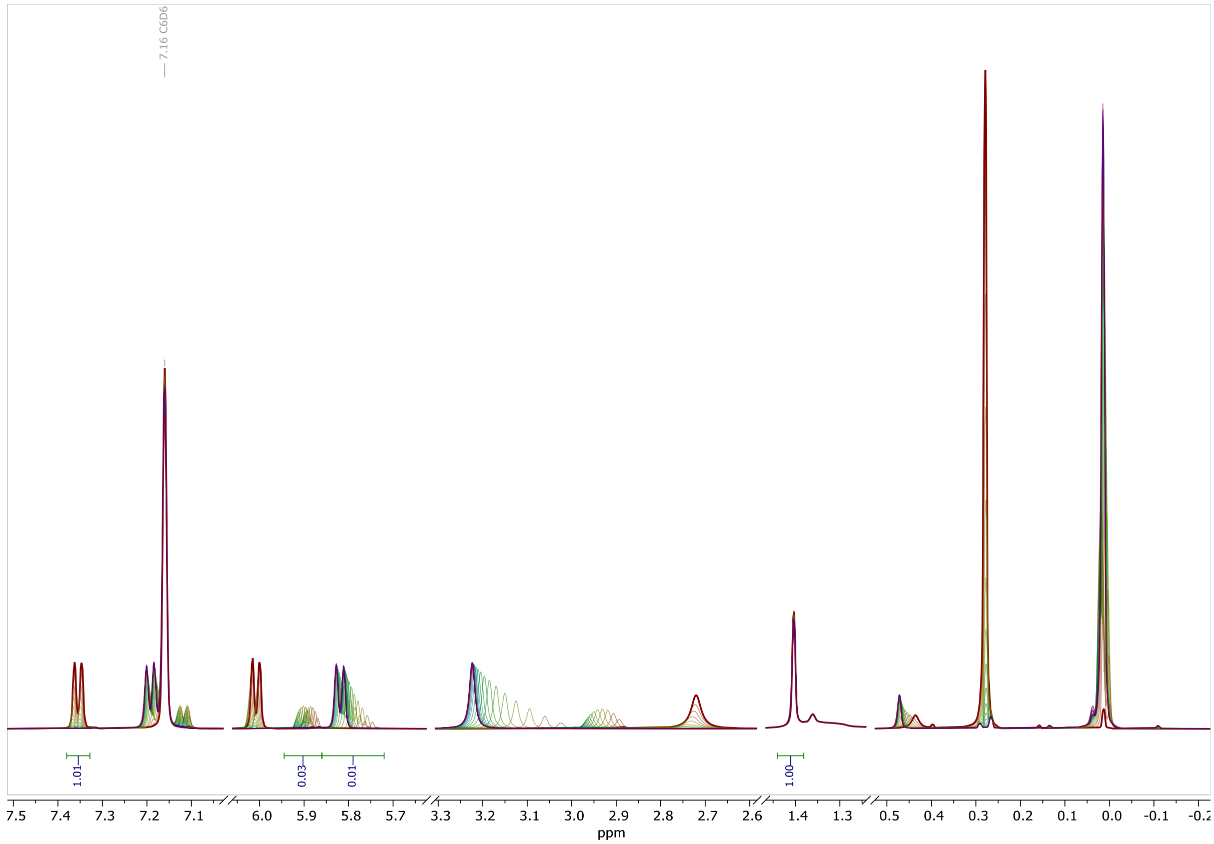

Figure S9.17.1. ^{1}H-NMR spectra (500 MHz, C_6D_6; selected regions shown) corresponding to entry SI-31. Superimposed spectra of all ^{1}H NMR spectra recorded during the experiment. The first spectrum recorded is colored red and the last spectrum recorded is colored purple. The spectra are recorded precisely every 25 minutes for the first 7 hours and 30 minutes and every 60 min for the remaining time of the experiment. Only the regions with signals are shown; intermediate regions were removed as indicated by the slashes. The signal at 1.4 ppm corresponds to cyclohexane which was used as an internal standard.

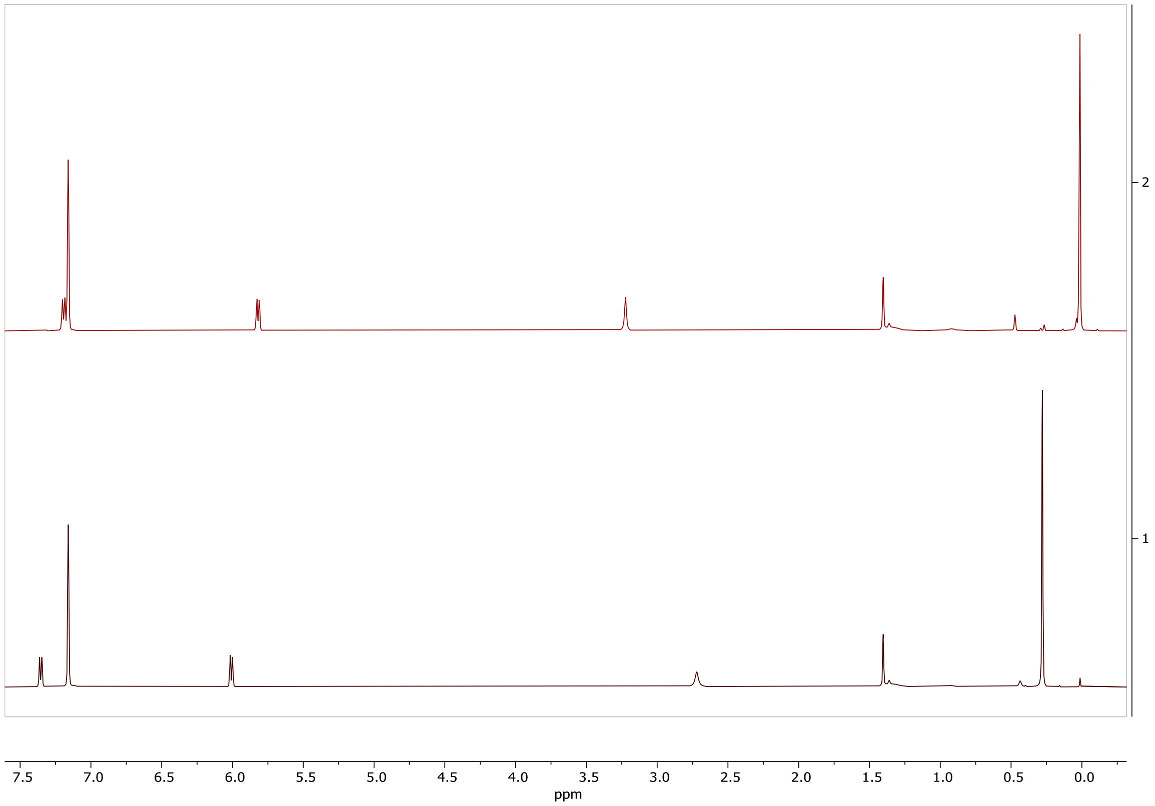

Figure S9.17.2. ^{1}H-NMR spectra (500 MHz, C_6D_6) corresponding to entry SI-31. Bottom spectrum was recorded at start of the reaction, and top spectrum was recorded after 14 hours and 40 minutes of reaction time. The signal at 1.4 ppm corresponds to cyclohexane which was used as an internal standard.

Section 9.18 – Entry SI-32

Reaction between **A** (R = *p*-NH_2) and **B** in C_6D_6. The ratio of **A:B** is 2:1. Concentration of **A** = 0.069 M. It was conducted to investigate the influence of water present by extra carefully drying the solvent before it was used.

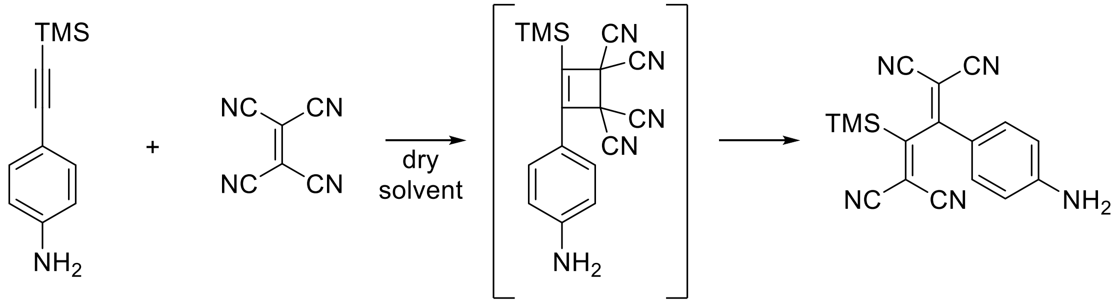

A **B** **C** **P**

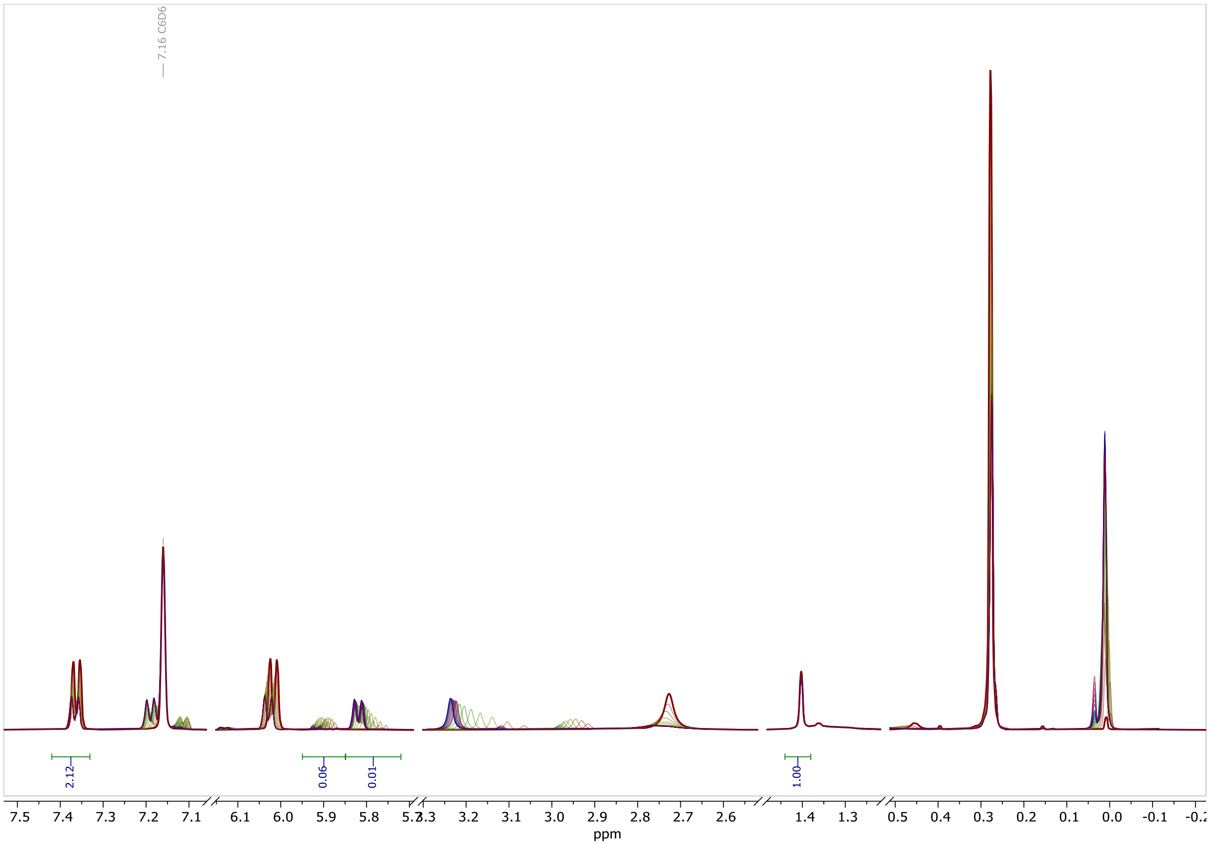

Figure S9.18.1. ^{1}H-NMR spectra (500 MHz, C_6D_6; selected regions shown) corresponding to entry SI-32. Superimposed spectra of all ^{1}H NMR spectra recorded during the experiment. The first spectrum recorded is colored red and the last spectrum recorded is colored purple. The spectra are recorded precisely every 25 minutes for the first 7 hours and 30 minutes and every 60 min for the remaining time of the experiment. Only the regions with signals are shown; intermediate regions were removed as indicated by the slashes. The signal at 1.4 ppm corresponds to cyclohexane which was used as an internal standard.

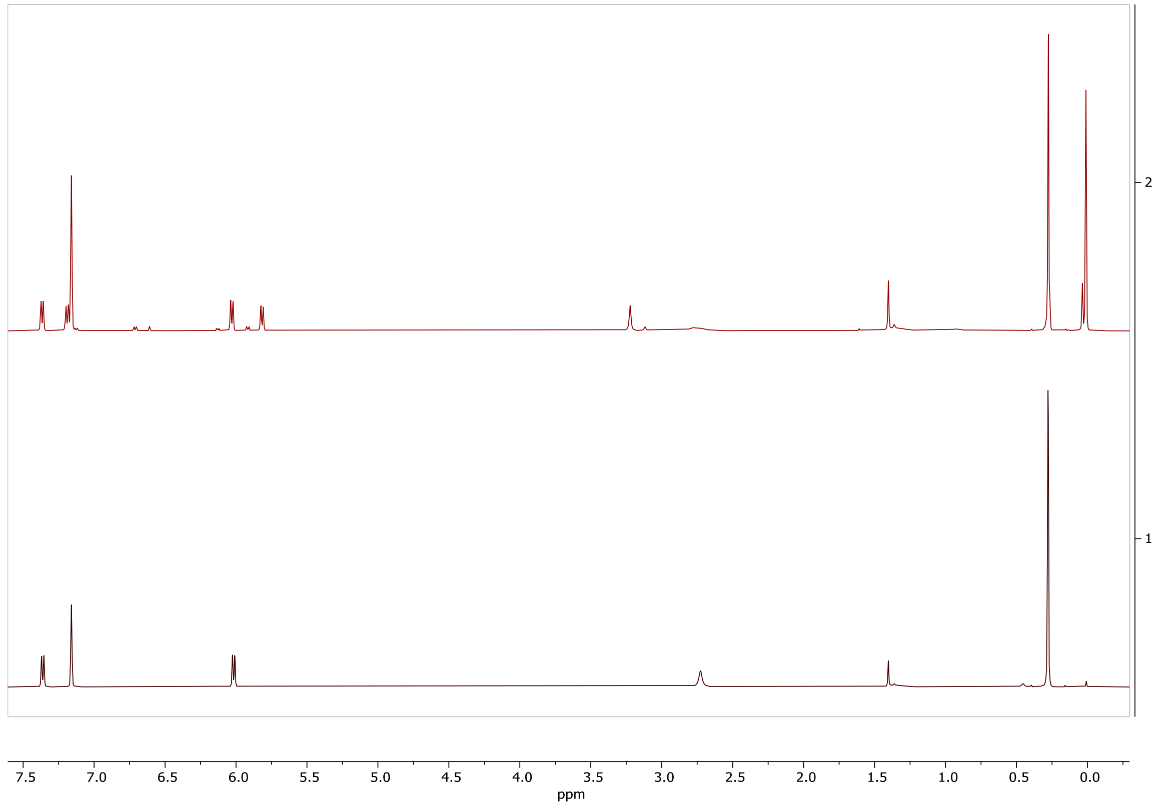

Figure S9.18.2. ^{1}H-NMR spectra (500 MHz, C_6D_6) corresponding to entry SI-32. Bottom spectrum was recorded at start of the reaction, and top spectrum was recorded after 13 hours and 40 minutes of reaction time. The signal at 1.4 ppm corresponds to cyclohexane which was used as an internal standard.

Section 9.18A – Comparison of experiments testing the influence of water

Below is 3 graphs who compare the experiments with ratios of 1:1, 1:2, and 2:1 experiments.

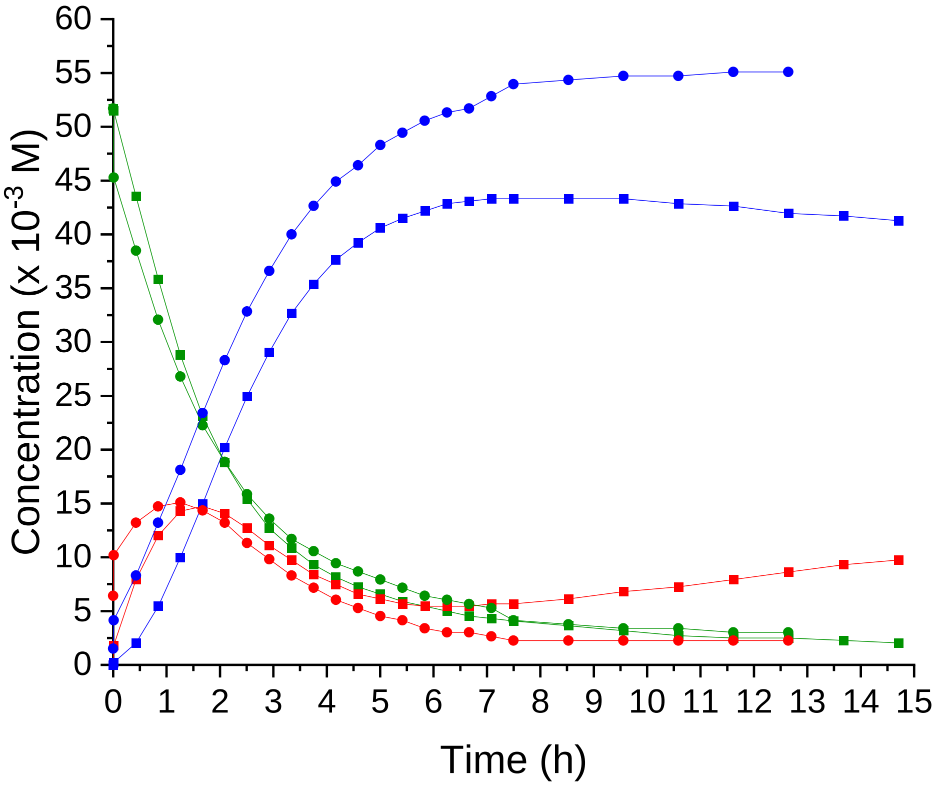

Figure S9.18A.1. Concentrations of species from experiment corresponding to entries SI-27 and SI-30 against time. Entry SI-27 data points are squares and Entry SI-30 data points are circles. In water saturated solvents protodesilylation was observed and ^{1}H-NMR of the deprotected product overlaps with the ^{1}H-NMR of intermediate **C** resulting in calculated concentration of **C** increases even if the actual concentration does not.

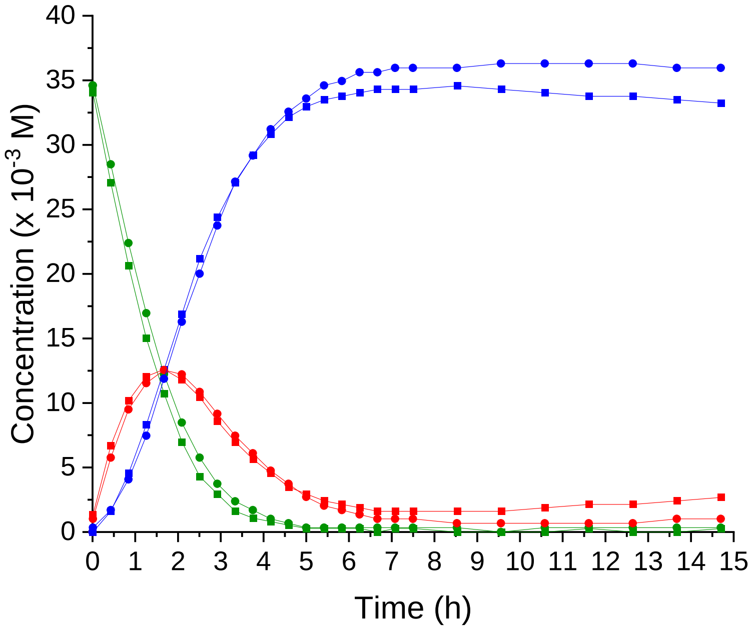

Figure S9.18A.2. Concentrations of species from experiment corresponding to entries SI-28 and SI-31 against time. Entry SI-28 data points are squares and Entry SI-31 data points are circles. In water saturated solvents protodesilylation was observed and ^{1}H-NMR of the deprotected product overlaps with the ^{1}H-NMR of intermediate **C** resulting in calculated concentration of **C** increases even if the actual concentration does not.

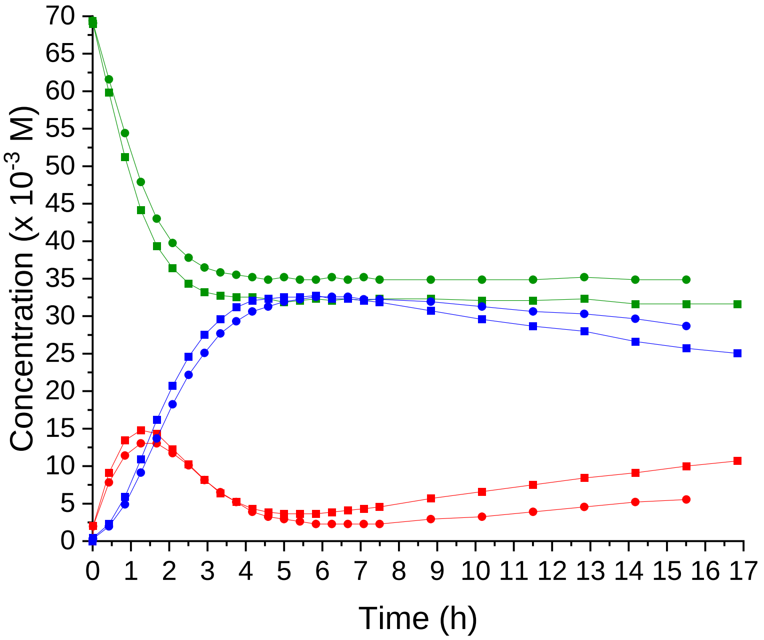

Figure S9.18A.3. Concentrations of species from experiment corresponding to entries SI-29 and SI-32 against time. Entry SI-29 data points are squares and Entry SI-32 data points are circles. In both experiments protodesilylation was observed and ^{1}H-NMR of the deprotected product overlaps with the ^{1}H-NMR of intermediate **C** resulting in calculated concentration of **C** increases even if the actual concentration does not.

Section 9.19 – Entry SI-33

Reaction between **A** (R = *p*-NH_2) and **B** in C_6D_6. The ratio of **A:B:TFA** is 1:1:1. Concentration of **A** and **B** and **TFA** = 0.027 M. This experiment tested the effect of the addition of strong acids like triflouroacetic acid (TFA). One equivalent of TFA was added at the start of the reaction.

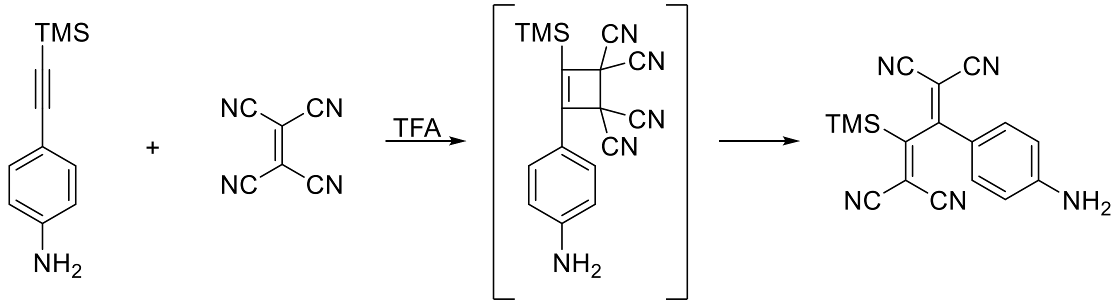

A **B** **C** **P**

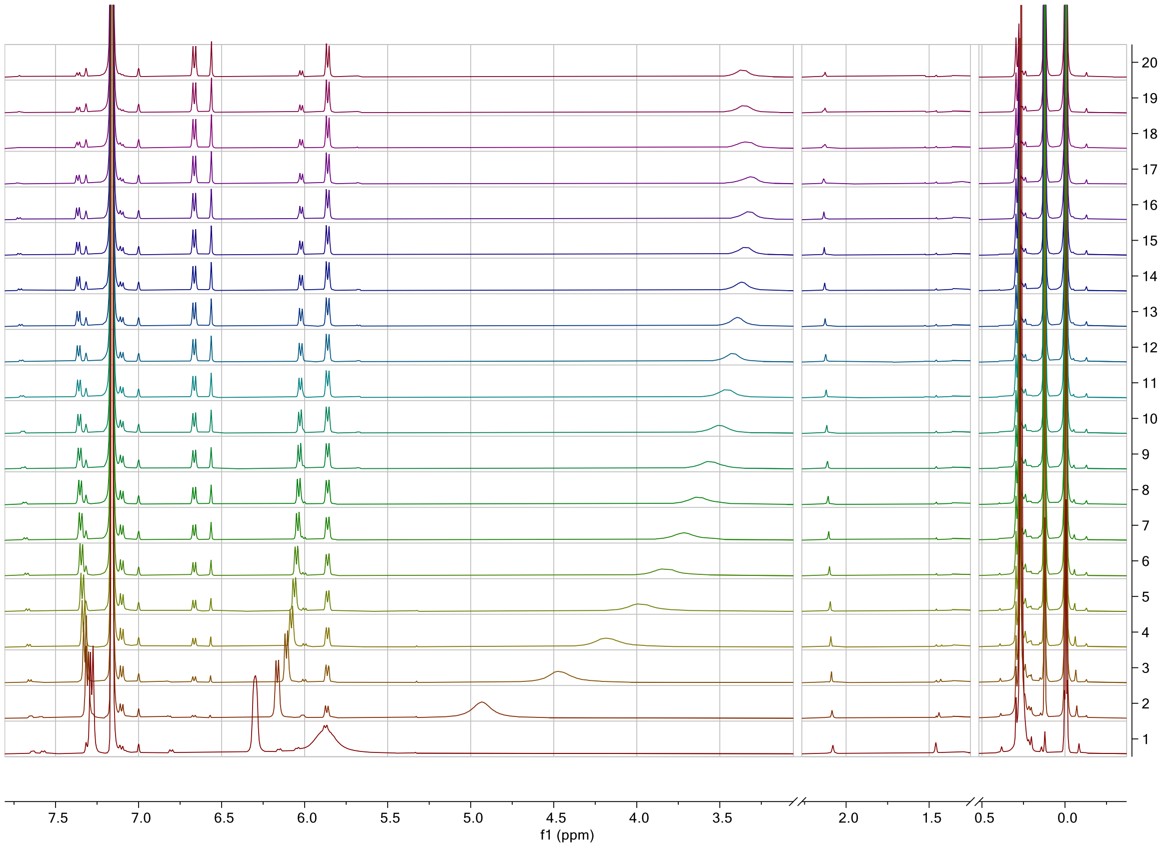

Figure S9.19.1. ^{1}H-NMR spectra (500 MHz, C_6D_6; selected regions shown) corresponding to entry SI-33. Stacked spectra of every 2nd ^{1}H NMR spectrum recorded during the experiment. The first spectrum recorded is at the bottom and the last recorded at the top. The spectra were recorded precisely every 24 minutes during the whole experiment duration. Only the regions with signals are shown; intermediate regions were removed as indicated by the slashes. The signal at 6.58 ppm corresponds to the proton, which originates from protodesilylation of **P**.

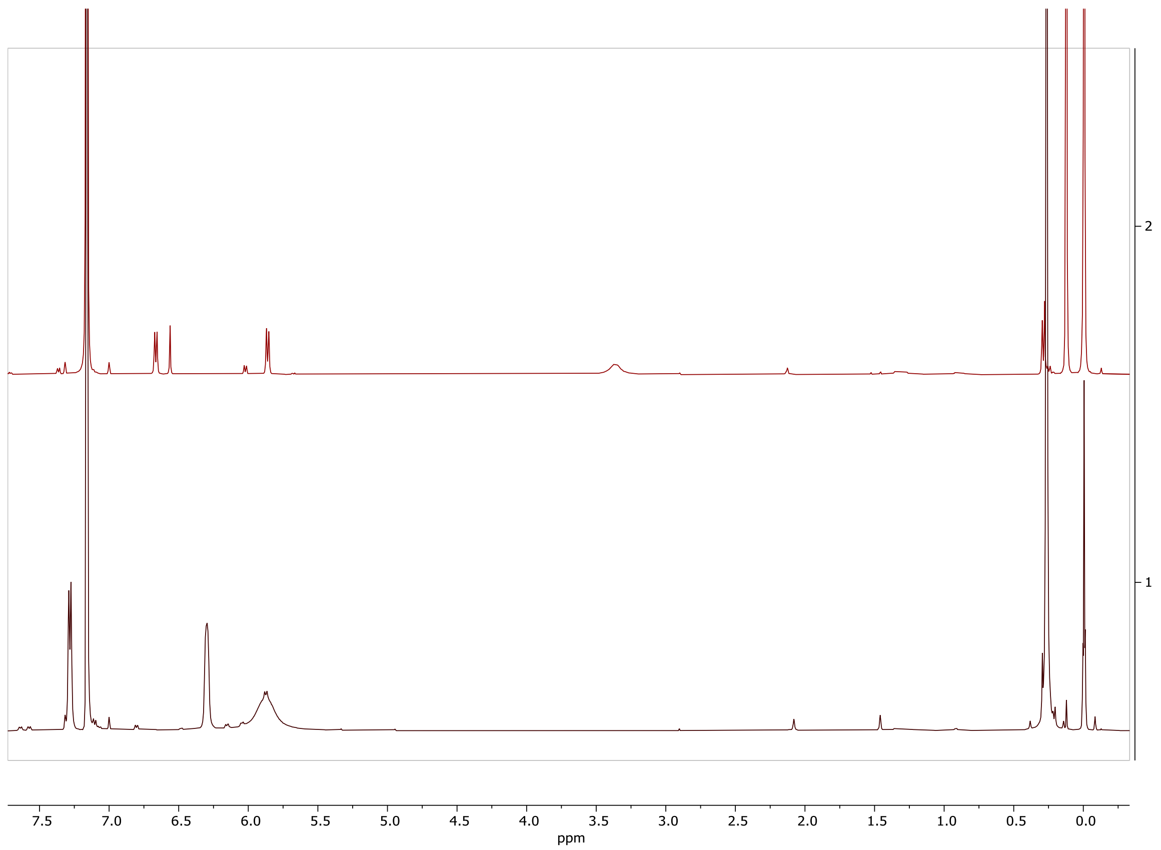

Figure S9.19.2. ^{1}H-NMR spectra (500 MHz, C_6D_6) corresponding to entry SI-33. Bottom spectrum was recorded at start of the reaction, and top spectrum was recorded after 7 hours and 20 minutes of reaction time. The signal at 6.58 ppm corresponds to the proton, which originates from protodesilylation of **P**.

Section 9.20 – Entry SI-34

Reaction between **A** (R = *p*-NH_2) and **B** in C_6D_6. The ratio of **A:B:TFA** is 1:1:0.5. Concentration of **A** and **B** = 0.027 M. This experiment tested the effect of the addition of strong acids like triflouroacetic acid (TFA). ½ equivalent of TFA was added at the start of the reaction.

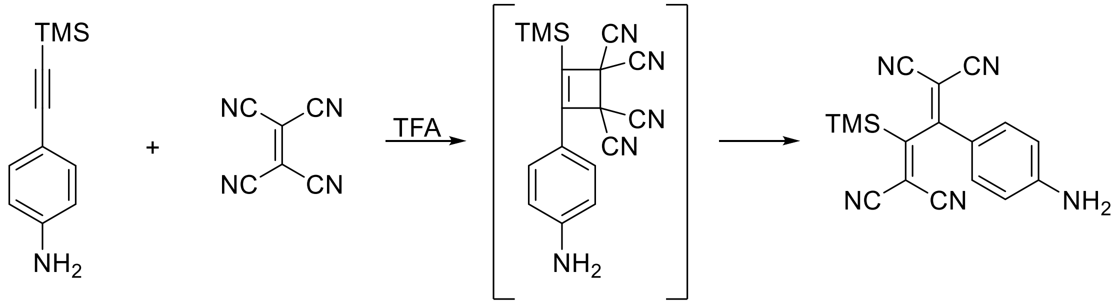

A **B** **C** **P**

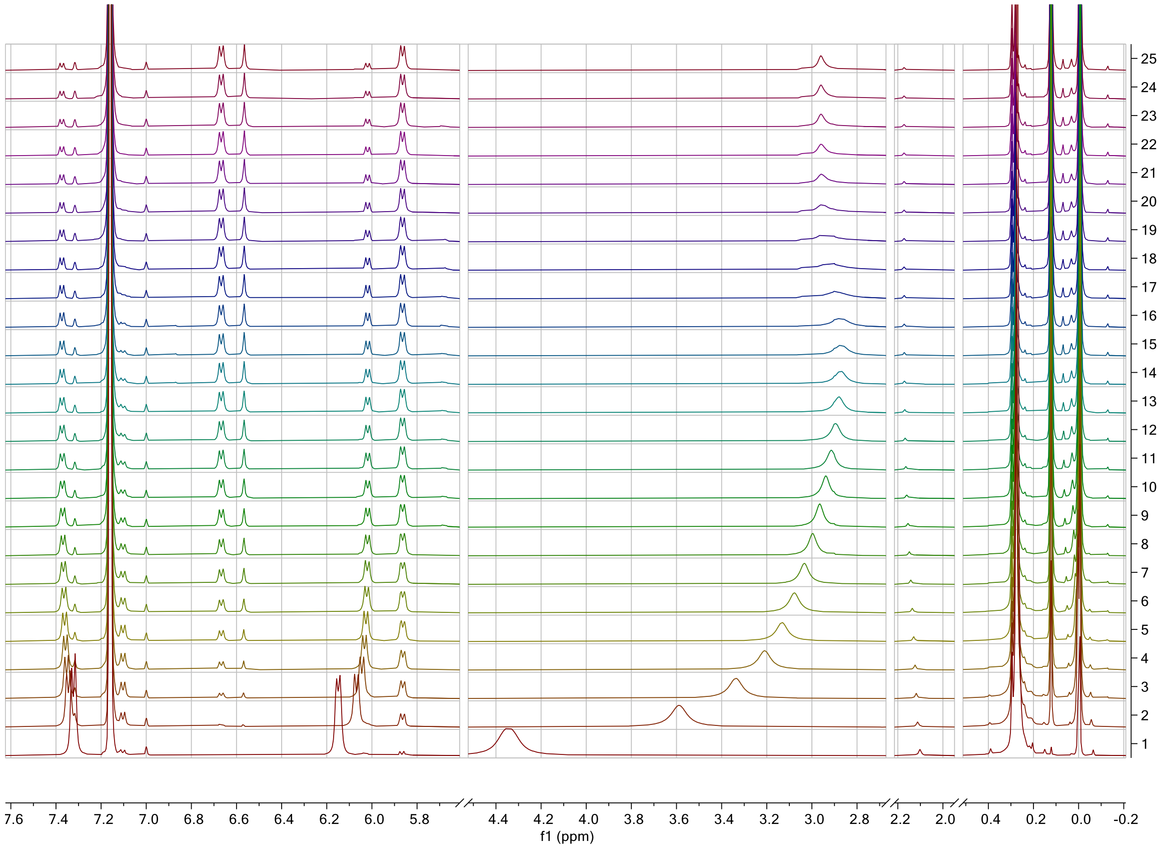

Figure S9.20.1. ^{1}H-NMR spectra (500 MHz, C_6D_6; selected regions shown) corresponding to entry SI-34. Stacked spectra of every 4th ^{1}H NMR recorded during the experiment. The first spectrum recorded is at the bottom and the last recorded at the top. The spectra were recorded precisely every 20 minutes during the whole experiment duration. Only the regions with signals are shown; intermediate regions were removed as indicated by the slashes. The signal at 6.58 ppm corresponds to the proton, which originates from protodesilylation of **P**.

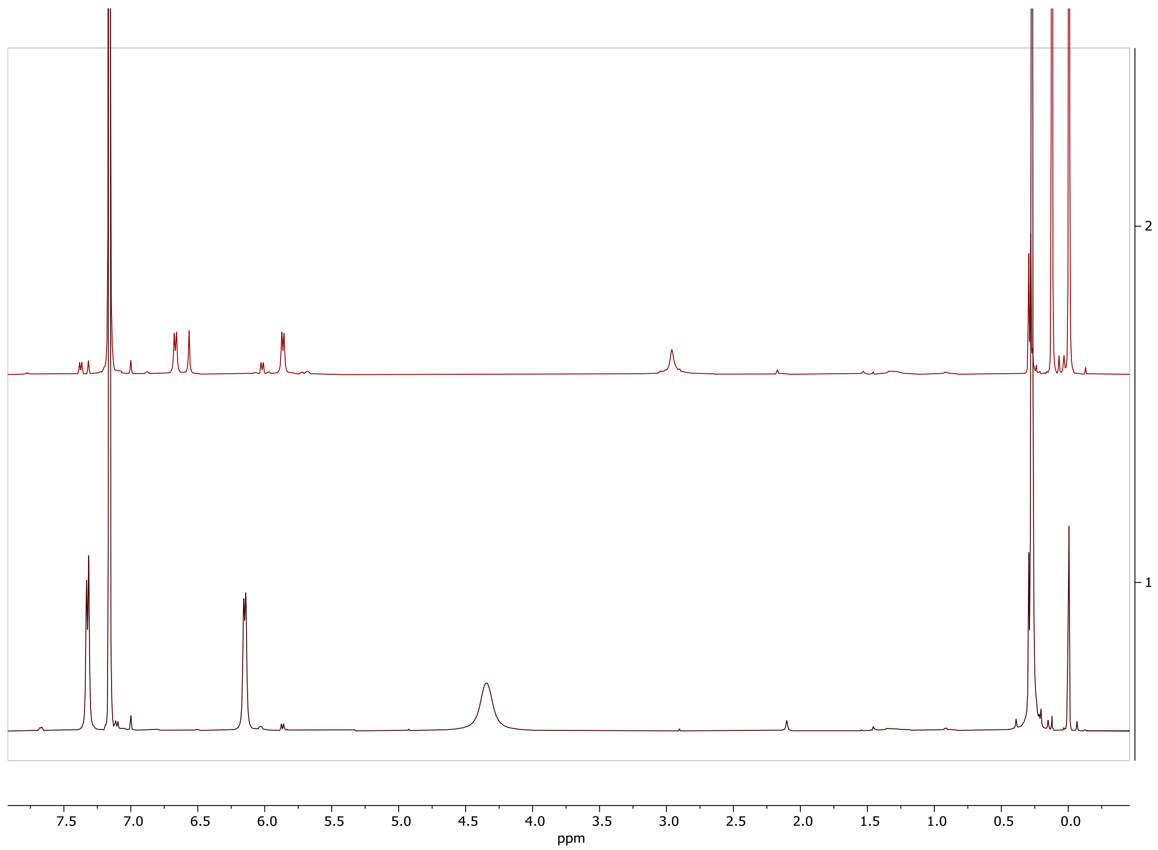

Figure S9.20.2. ^{1}H-NMR spectra (500 MHz, C_6D_6) corresponding to entry SI-34. Bottom spectrum was recorded at start of the reaction, and top spectrum was recorded after 12 hours and 20 minutes of reaction time. The signal at 6.58 ppm corresponds to the proton, which originates from protodesilylation of **P**.

Section 9.21 – Entry SI-35

Reaction between **A** (R = *p*-NH_2) and **B** in C_6D_6. The ratio of **A:B** is 1:1. This experiment tested the effect of the addition of strong acids like triflouroacetic acid (TFA). Large excess amount of TFA was added at the start of the reaction.

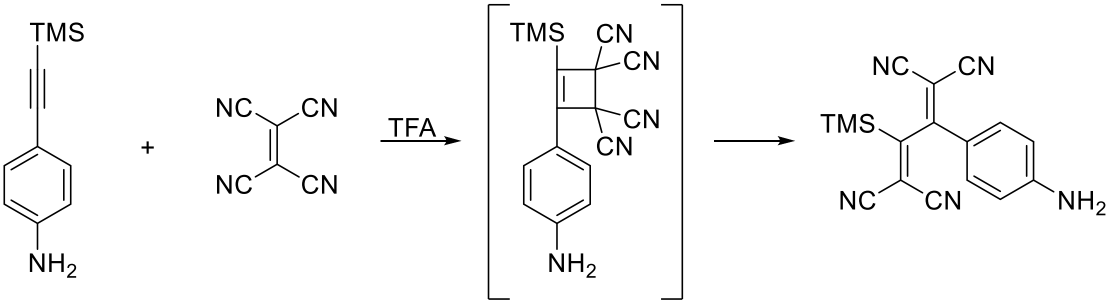

A **B** **C** **P**

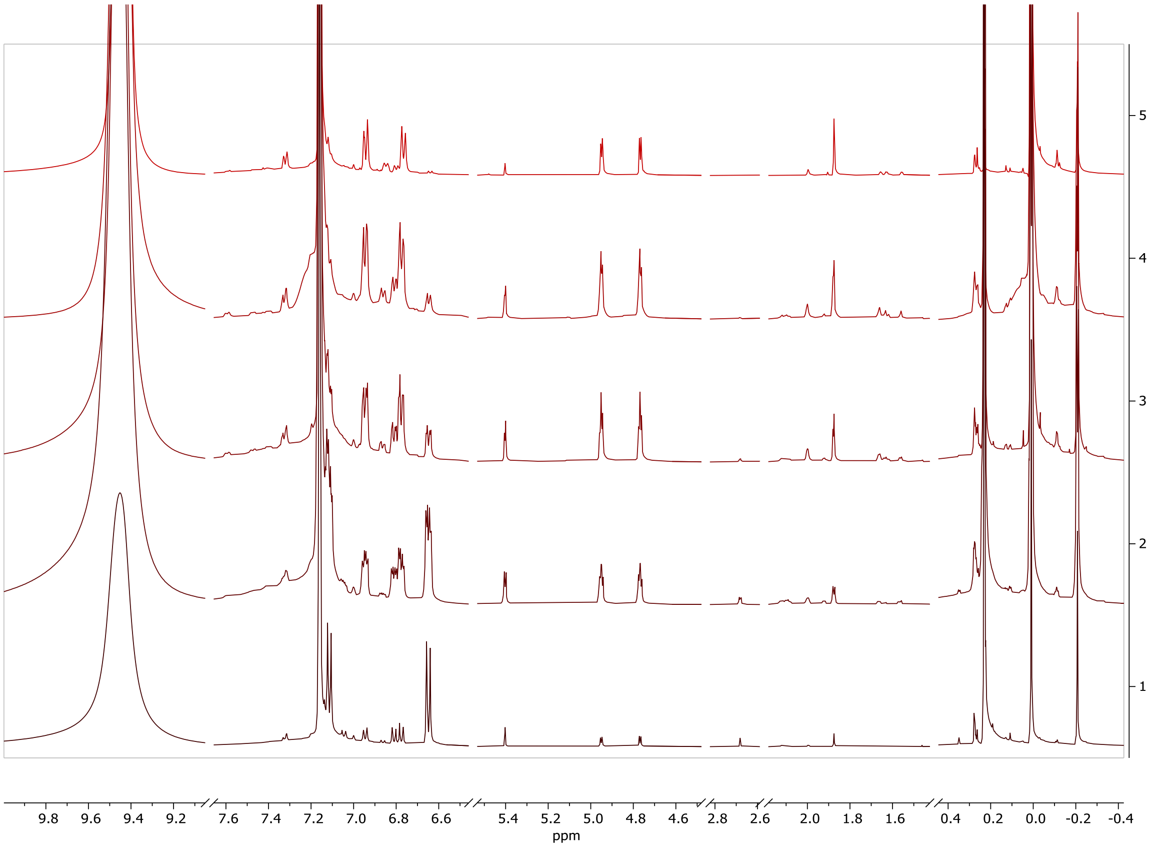

Figure S9.21.1. ^{1}H-NMR spectra (500 MHz, C_6D_6; selected regions shown) corresponding to entry SI-35. Stacked spectra of all ^{1}H NMR recorded during the experiment. The first spectrum recorded is at the bottom and the last recorded at the top. The spectra were recorded precisely after 12, 22, 70, 100 and 220 minutes, respectively Only the regions with signals are shown; intermediate regions were removed as indicated by the slashes.

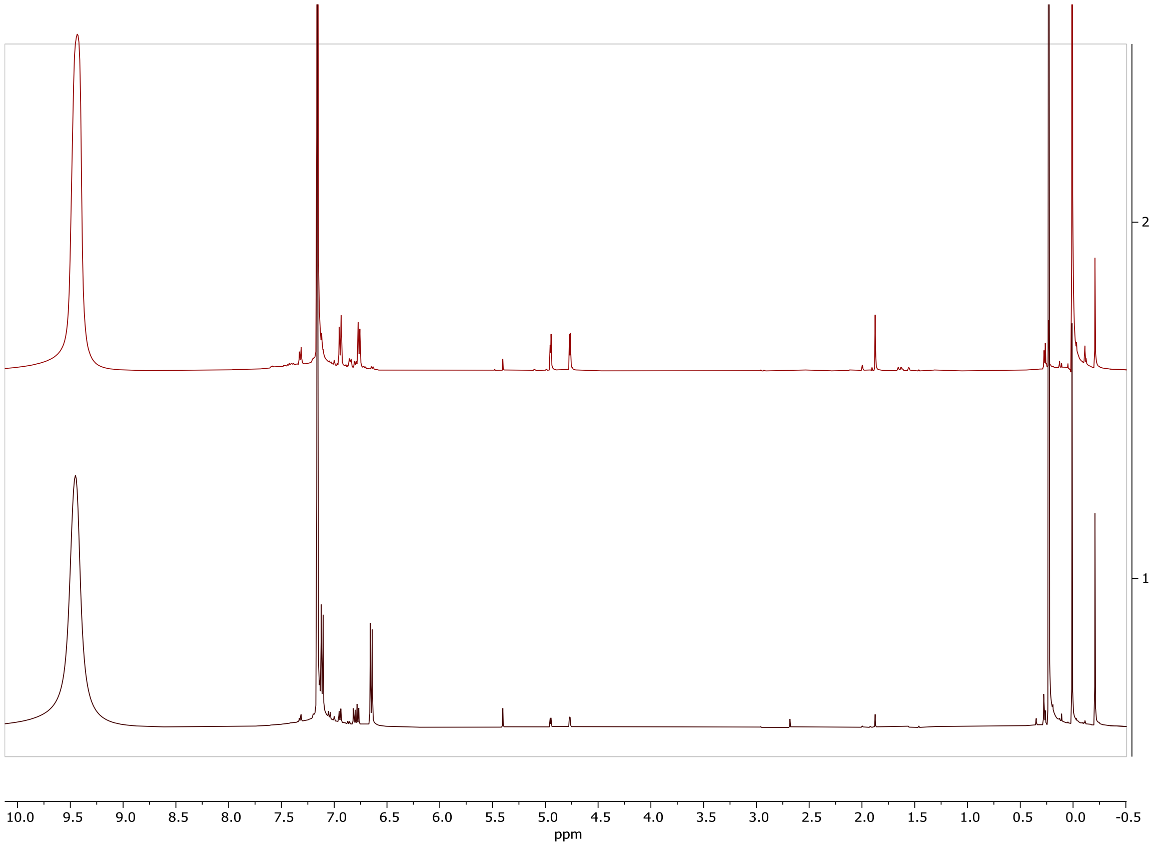

Figure S9.21.2. ^{1}H-NMR spectra (500 MHz, C_6D_6) corresponding to entry SI-35. Bottom spectrum was recorded after 12 minutes of reaction time, and top spectrum was recorded after 3 hours and 40 minutes of reaction time.

Section 9.22 – Entry SI-36

Reaction between **A** (R = *p*-NH_2) and **B** in C_6D_6. The ratio of **A:B:AcOH** is 1:1:0.25. Concentration of **A** and **B** = 0.036 M. This experiment tested the effect of the addition of weaker acids like acetic acid. 0.25 equivalent of AcOH was added at the start of the reaction.

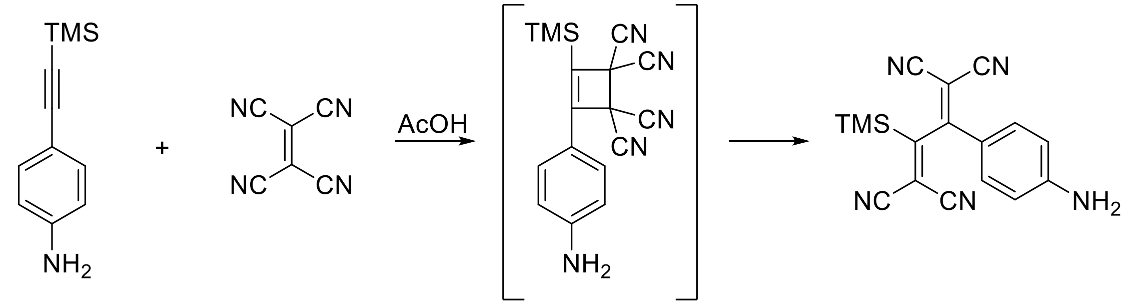

A **B** **C** **P**

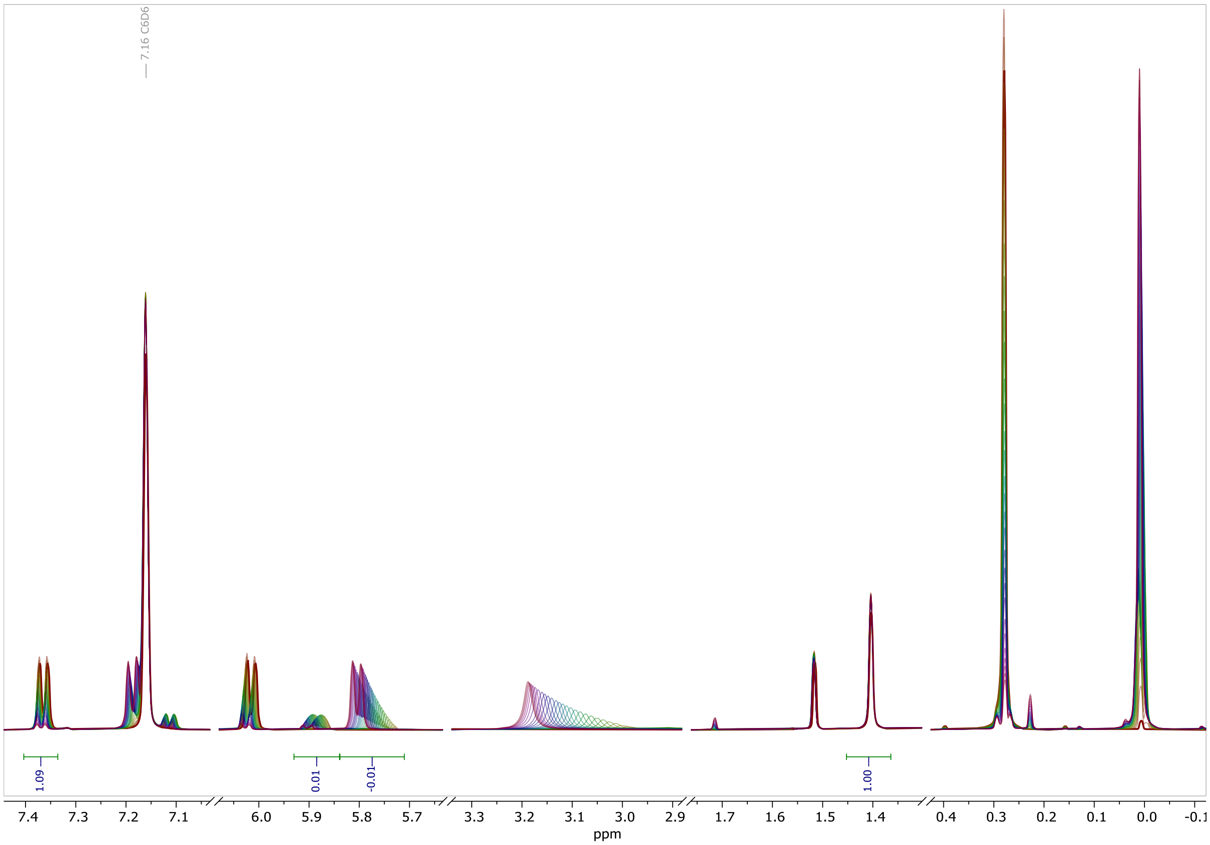

Figure S9.22.1. ^{1}H-NMR spectra (500 MHz, C_6D_6; selected regions shown) corresponding to entry SI-36. Superimposed spectra of all ^{1}H NMR spectra recorded during the experiment. The first spectrum recorded is colored red, and the last spectrum recorded is colored purple. The spectra were recorded precisely every 15 minutes during the full experiment duration. Only the regions with signals are shown; intermediate regions were removed as indicated by the slashes. The signal at 1.40 ppm corresponds to cyclohexane which was used as an internal standard.

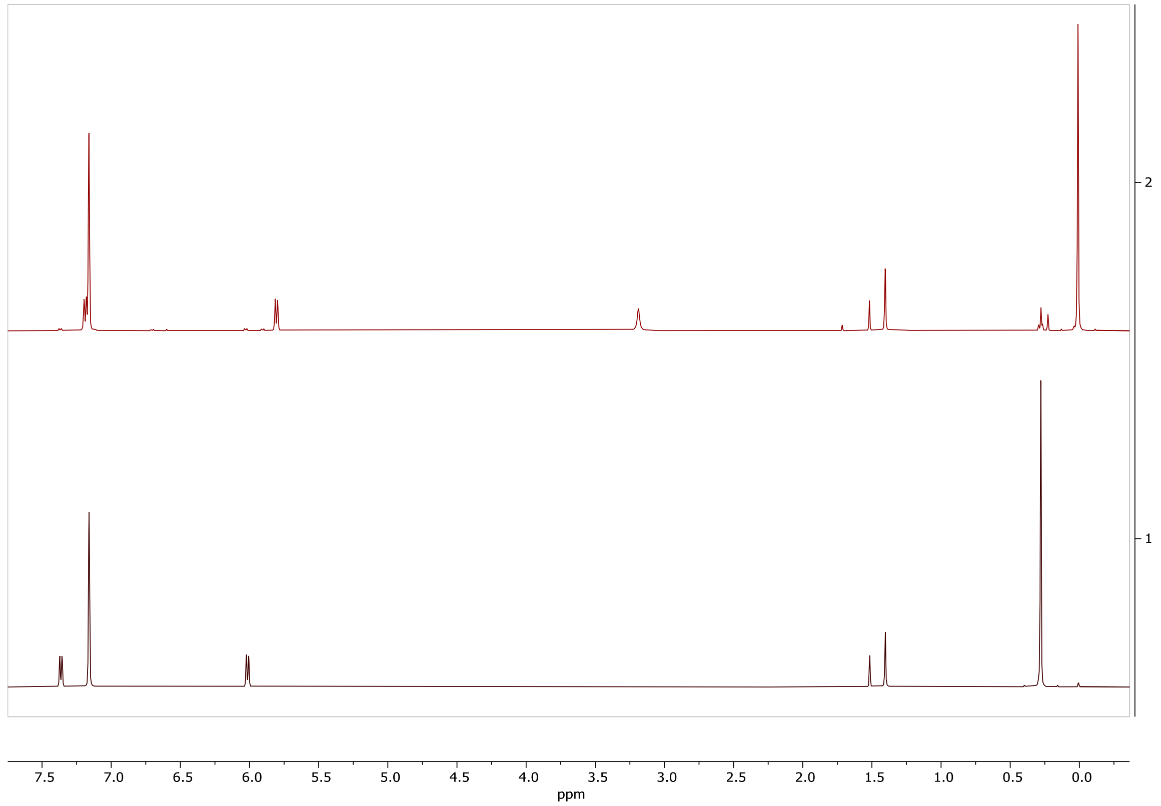

Figure S9.22.2. ^{1}H-NMR spectra (500 MHz, C_6D_6) corresponding to entry SI-36. Bottom spectrum was recorded at start of the reaction, and top spectrum was recorded after 15 hours and 15 minutes of reaction time. The signal at 1.40 ppm corresponds to cyclohexane, which was used as an internal standard.

Section 9.23 – Entry SI-37

Reaction between **A** (R = *p*-NH_2) and **B** in C_6D_6. The ratio of **A:B:AcOH** is 1:1:0.5. Concentration of **A** and **B** = 0.037 M. This experiment tested the effect of the addition of weaker acids like acetic acid. ½ equivalent of AcOH was added at the start of the reaction.

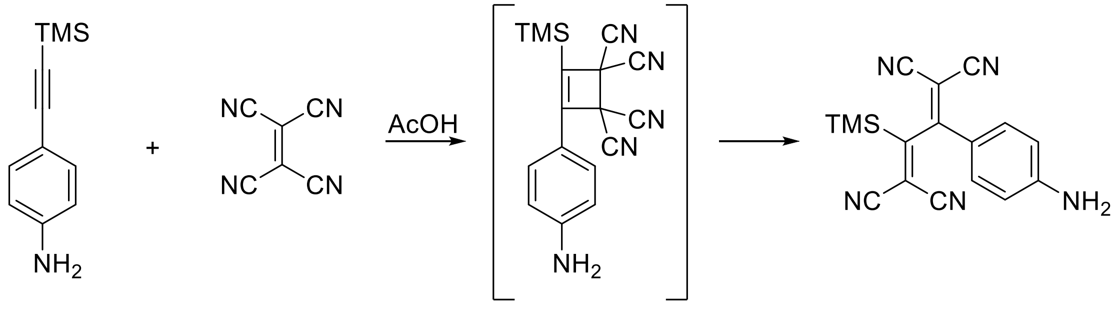

A **B** **C** **P**

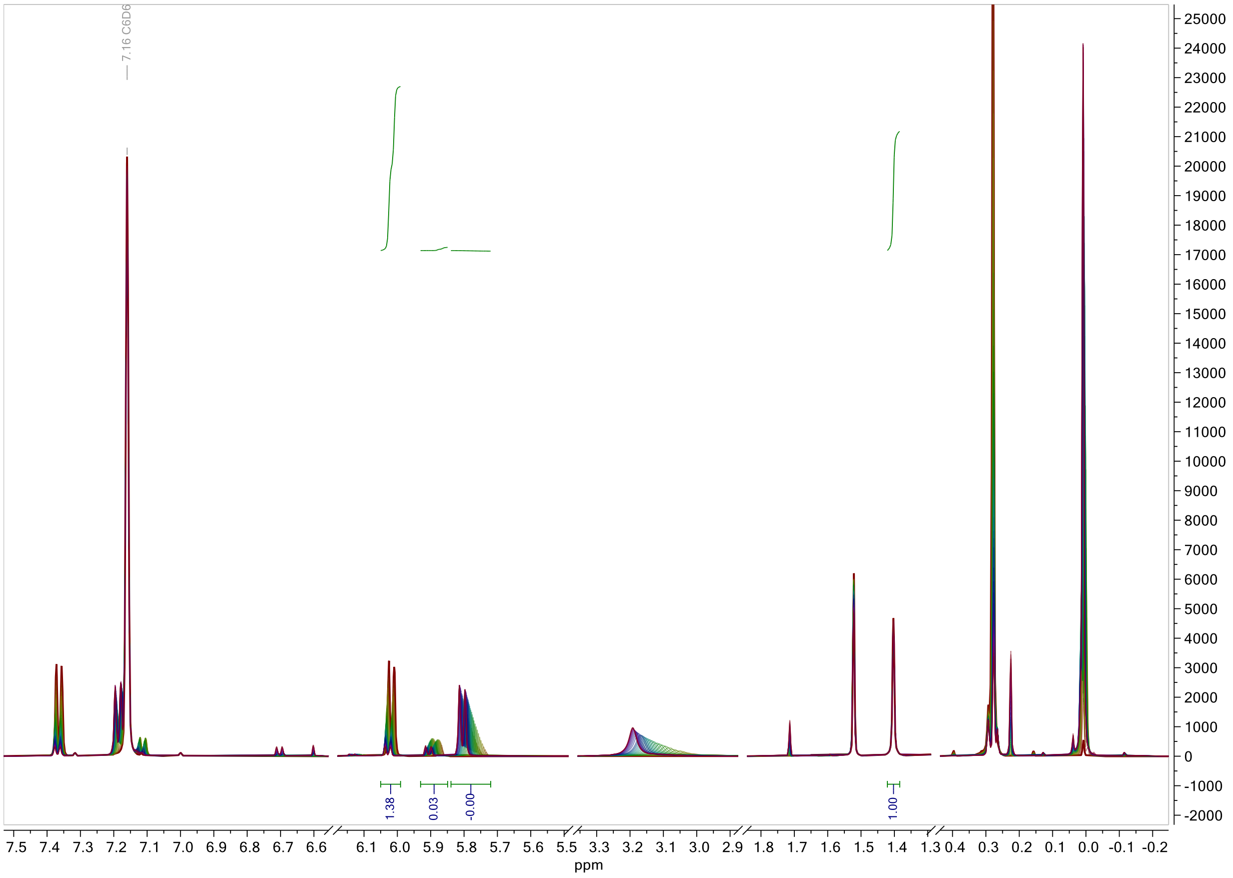

Figure S9.23.1. ^{1}H-NMR spectra (500 MHz, C_6D_6; selected regions shown) corresponding to entry SI-37. Superimposed spectra of all ^{1}H NMR spectra recorded during the experiment. The first spectrum recorded is colored red, and the last spectrum recorded is colored purple. The spectra were recorded precisely every 15 minutes for 6 hours and then every 40 minutes for the remaining time of the experiment duration. Only the regions with signals are shown; intermediate regions were removed as indicated by the slashes. The signal at 1.40 ppm corresponds to cyclohexane, which was used as an internal standard.

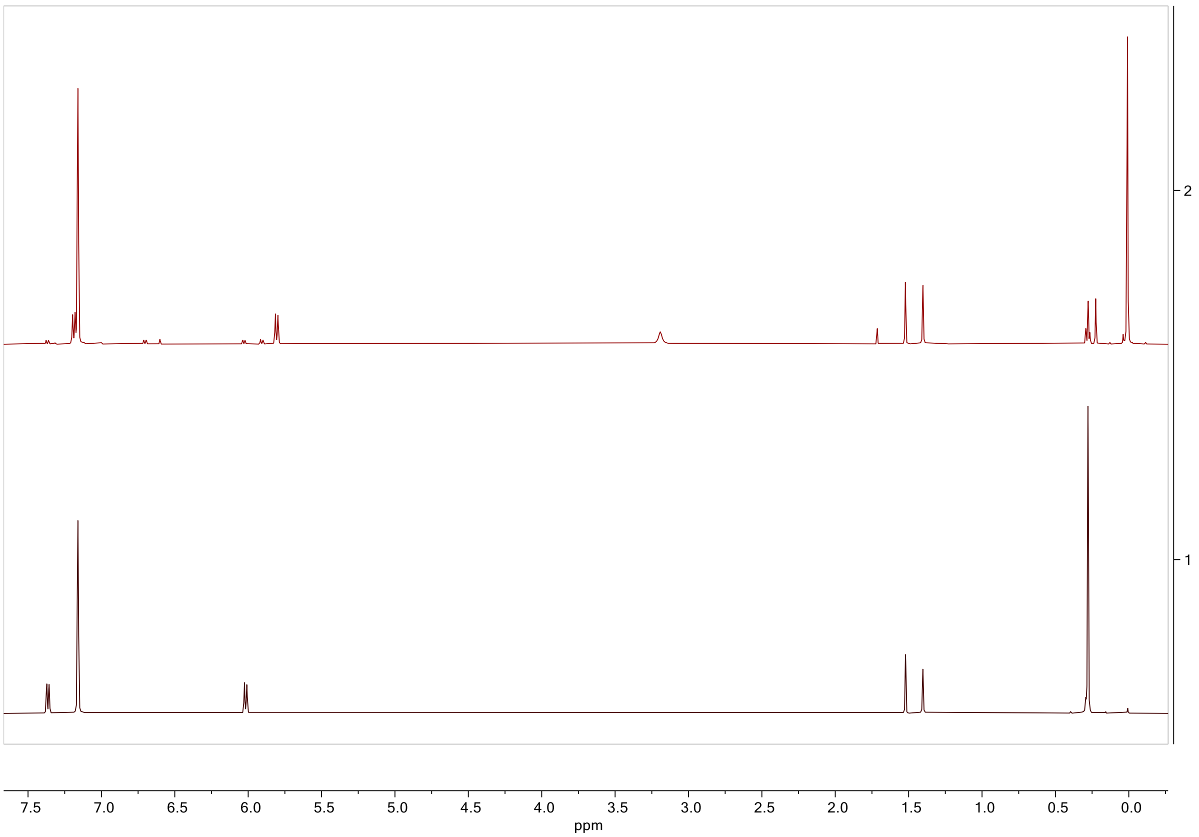

Figure S9.23.2. ^{1}H-NMR spectra (500 MHz, C_6D_6) corresponding to entry SI-37. Bottom spectrum was recorded at start of the reaction, and top spectrum was recorded after 14 hours and 40 minutes of reaction time. The signal at 1.40 ppm corresponds to cyclohexane, which was used as an internal standard.

Section 9.24 – Entry SI-38

Reaction between **A** (R = *p*-NH_2) and **B** in C_6D_6. The ratio of **A:B:AcOH** is 1:1:1. Concentration of **A** and **B** and **AcOH** = 0.037 M. This experiment tested the effect of the addition of weaker acids like acetic acid. 1 equivalent of AcOH was added at the start of the reaction.

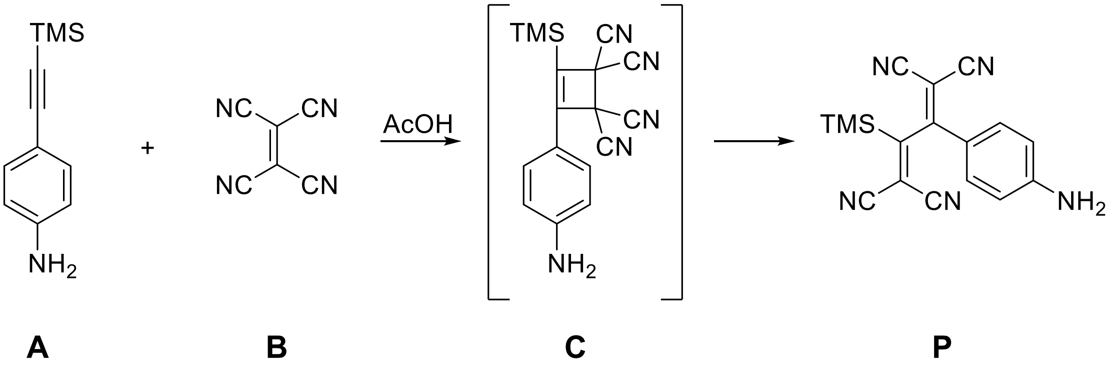

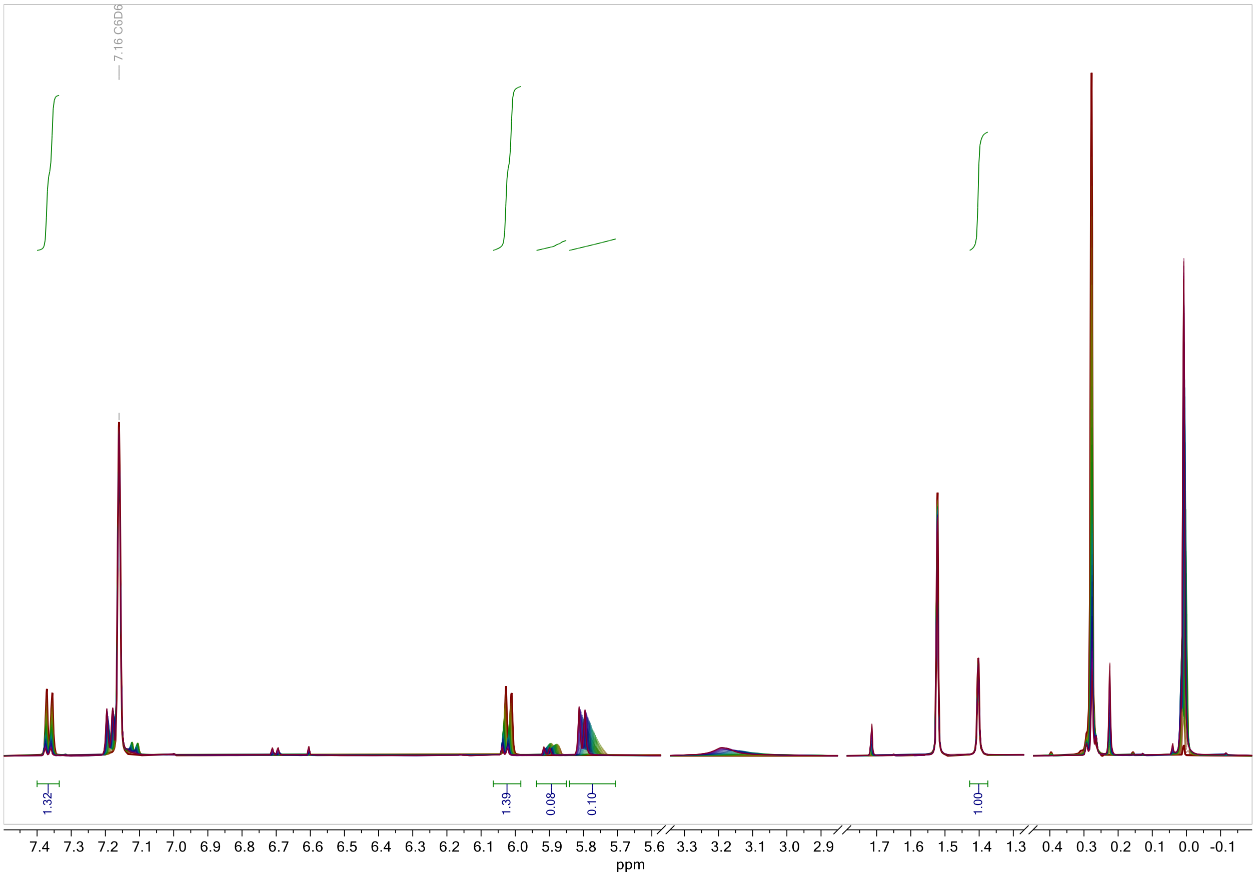

Figure S9.24.1. ^{1}H-NMR spectra (500 MHz, C_6D_6; selected regions shown) corresponding to entry SI-38. Superimposed spectra of all ^{1}H NMR spectra recorded during the experiment. The first spectrum recorded is colored red, and the last spectrum recorded is colored purple. The spectra were recorded precisely every 10 minutes for 5 hours and 30 minutes and then every 40 minutes for the remaining time of the experiment duration. Only the regions with signals are shown; intermediate regions were removed as indicated by the slashes. The signal at 1.40 ppm corresponds to cyclohexane, which was used as an internal standard.

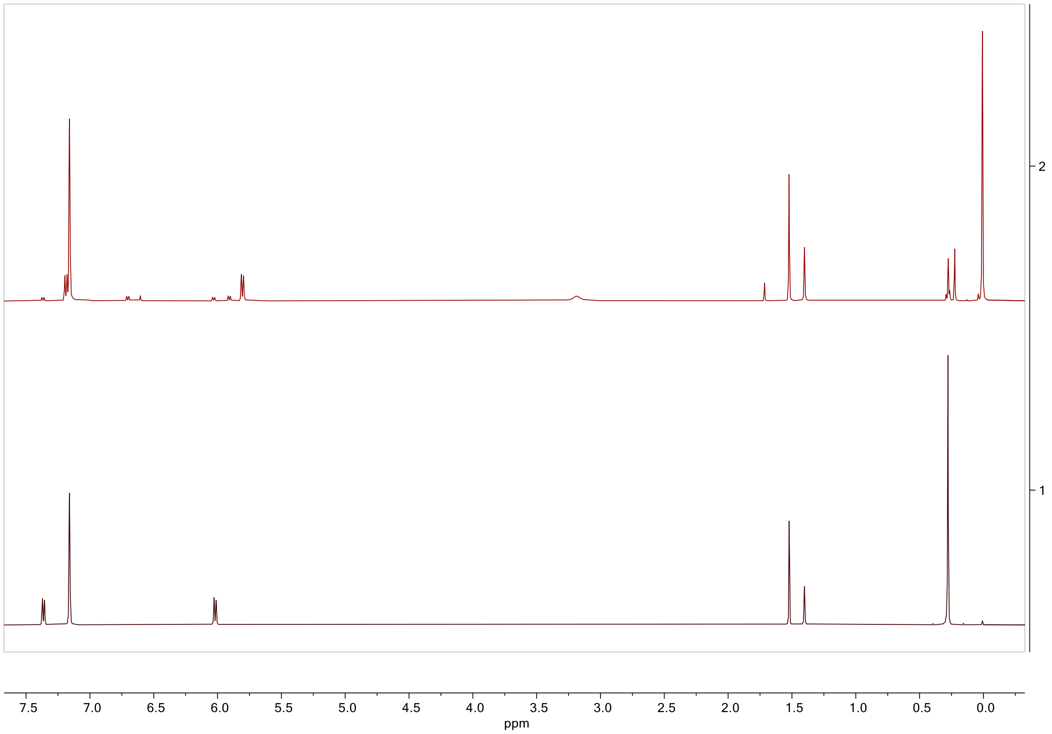

Figure S9.24.2. ^{1}H-NMR spectra (500 MHz, C_6D_6) corresponding to entry SI-38. Bottom spectrum was recorded at start of the reaction, and top spectrum was recorded after 13 hours and 20 minutes of reaction time. The signal at 1.40 ppm corresponds to cyclohexane, which was used as an internal standard.

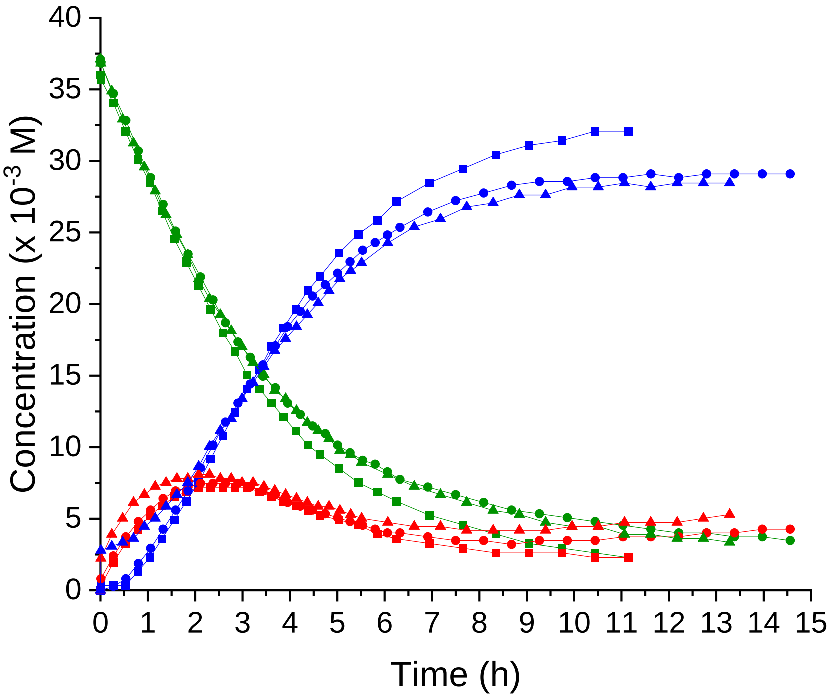

Figure S9.24.3. A comparison of entries SI-36 to SI-38 by showing concentration of species over time. Entry SI-36 has square data point, Entry SI-37 has circle data points, and Entry SI-38 has triangle data points.

Section 9.25 – Entry SI-39

Reaction between **A** (R = *p*-NH_2) and **B** in CD_2Cl_2. The ratio of **A:B:AcOH** is 1:1:1. Concentration of **A** and **B** and **AcOH** = 0.034 M. This experiment tested the effect of the addition of weaker acids like acetic acid but in another solvent than previously used. 1 equivalent of AcOH was added at the start of the reaction.

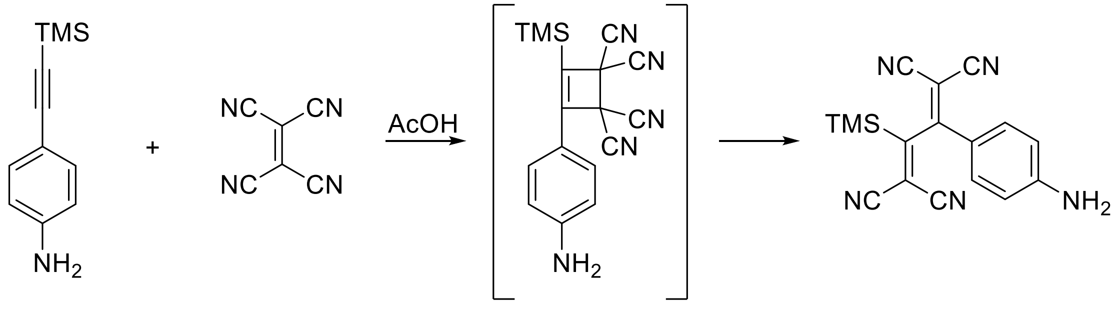

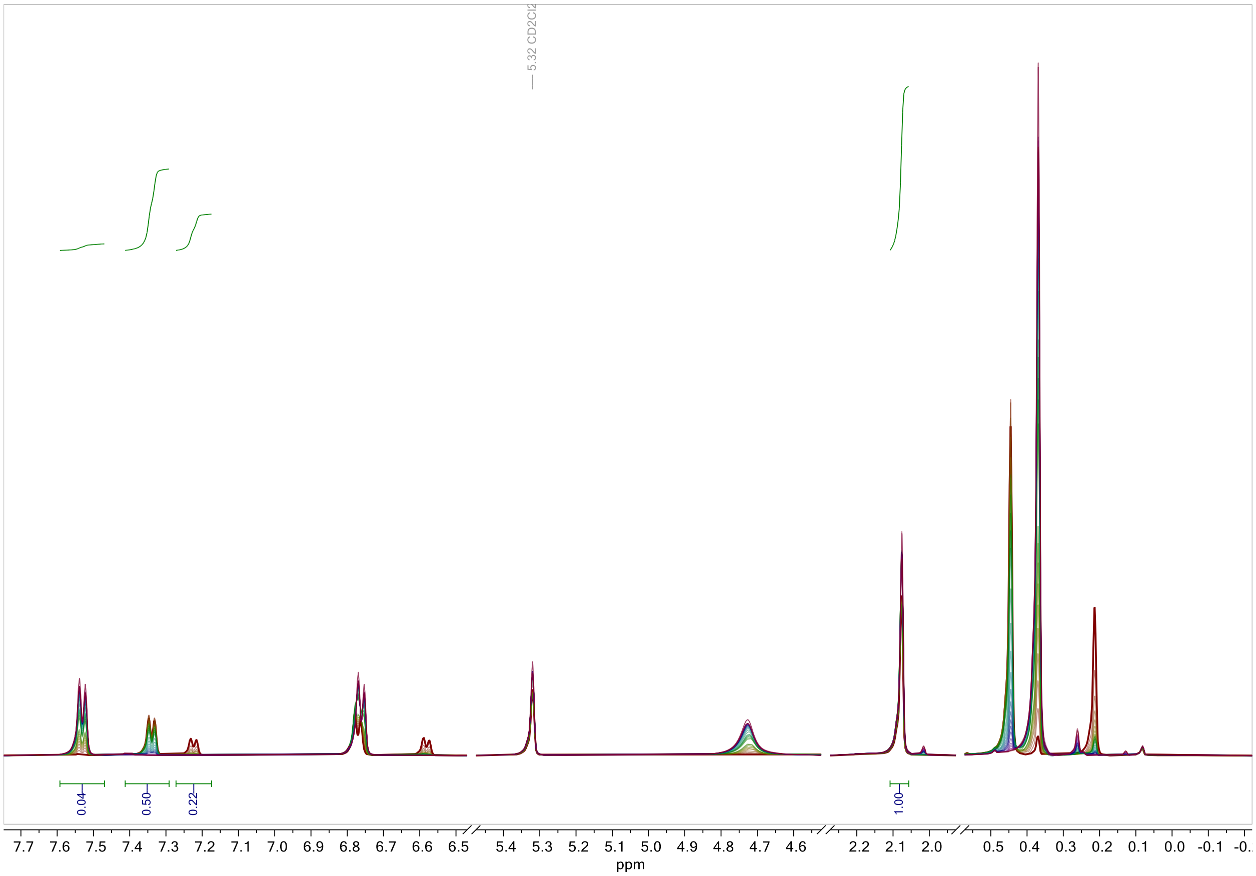

Figure S9.25.1. ^{1}H-NMR spectra (500 MHz, CD_2Cl_2; selected regions shown) corresponding to entry SI-39. Superimposed spectra of all ^{1}H NMR spectra recorded during the experiment. The first spectrum recorded is colored red, and the last spectrum recorded is colored purple. The spectra were recorded precisely every 4 minutes for 52 minutes and then every 10 minutes for the remaining time of the experiment duration. Only the regions with signals are shown; intermediate regions were removed as indicated by the slashes.

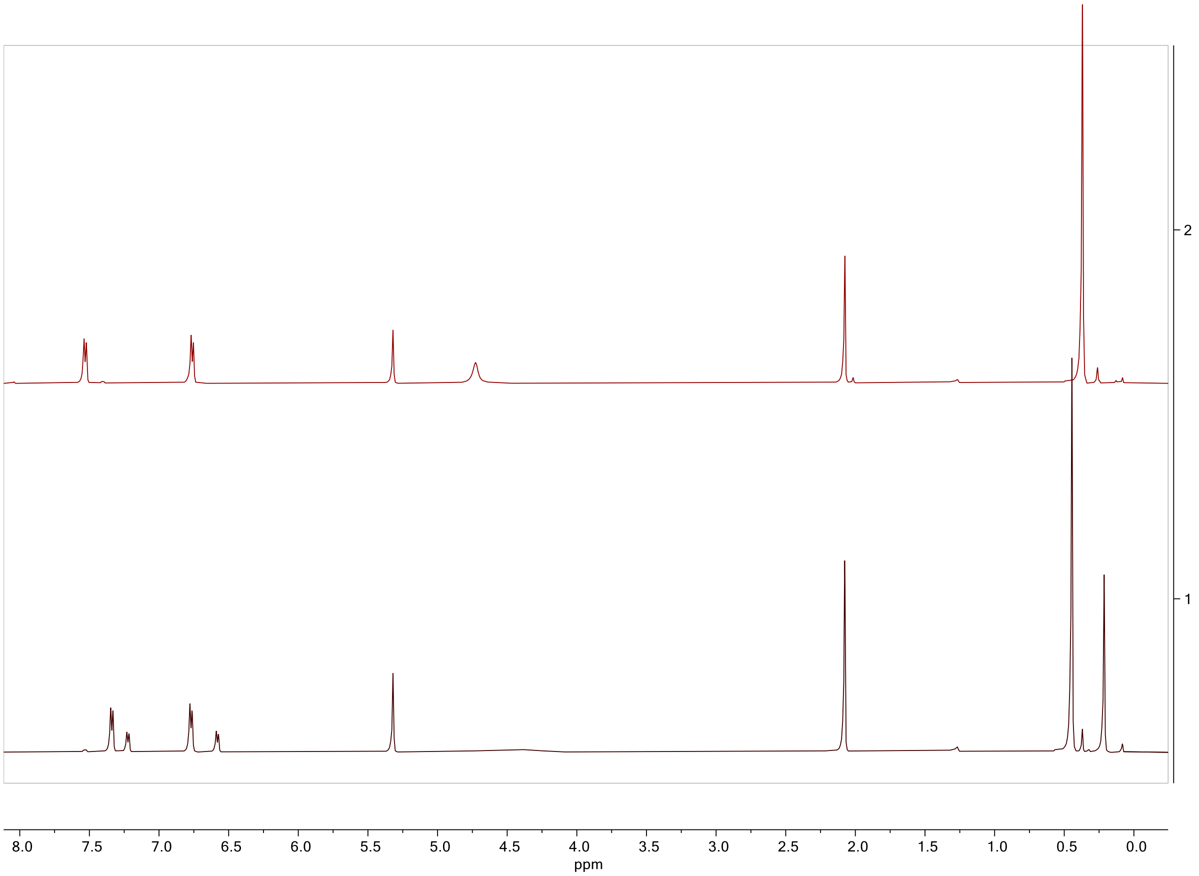

Figure S9.25.2. ^{1}H-NMR spectra (500 MHz, CD_2Cl_2) corresponding to entry SI-39. Bottom spectrum was recorded at start of the reaction, and top spectrum was recorded after 5 hours and 50 minutes of reaction time. In the bottom spectrum (first spectrum recorded), the intermediate had formed almost immediately explaining the two sets of aromatic protons.

Section 9.26 – Entry SI-40

Reaction between **A** (R = *p*-NH_2) and **B** in CD_3COOD. The ratio of **A:B** is 1:1. Concentration of **A** and **B** = 0.038 M and 0.039 M respectively. This experiment tested the effect of using a weak acid as solvent.

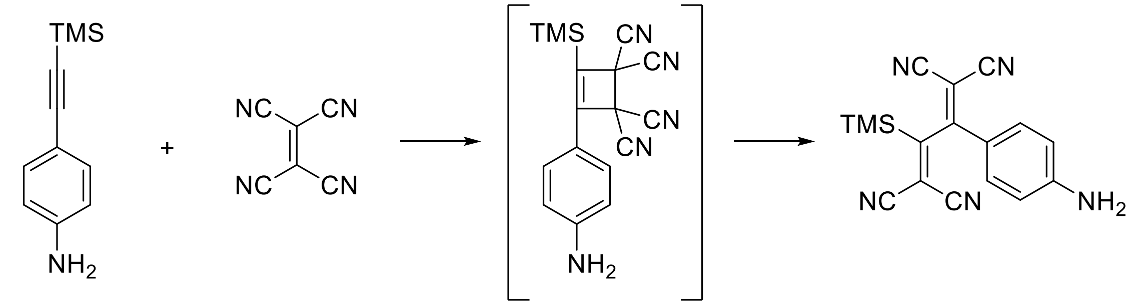

A **B** **C** **P**

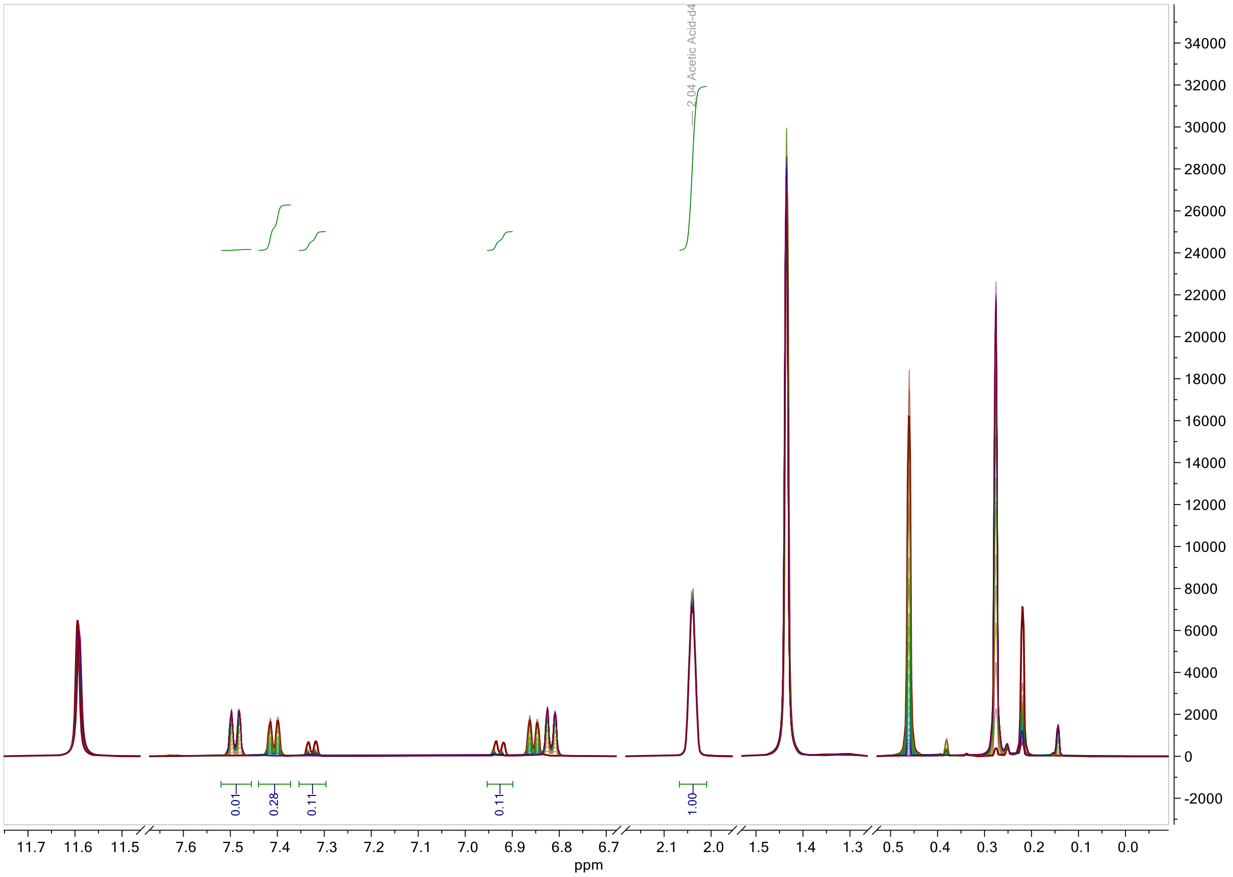

Figure S9.26.1. ^{1}H-NMR spectra (500 MHz, CD_3COOD; selected regions shown) corresponding to entry SI-40. Superimposed spectra of all ^{1}H NMR spectra recorded during the experiment. The first spectrum recorded is colored red, and the last spectrum recorded is colored purple. The spectra were recorded precisely every 15 minutes for 6 hours and then every 45 minutes for the remaining time of the experiment duration. Only the regions with signals are shown; intermediate regions were removed as indicated by the slashes. The signal at 1.40 ppm corresponds to cyclohexane, which was used as an internal standard.

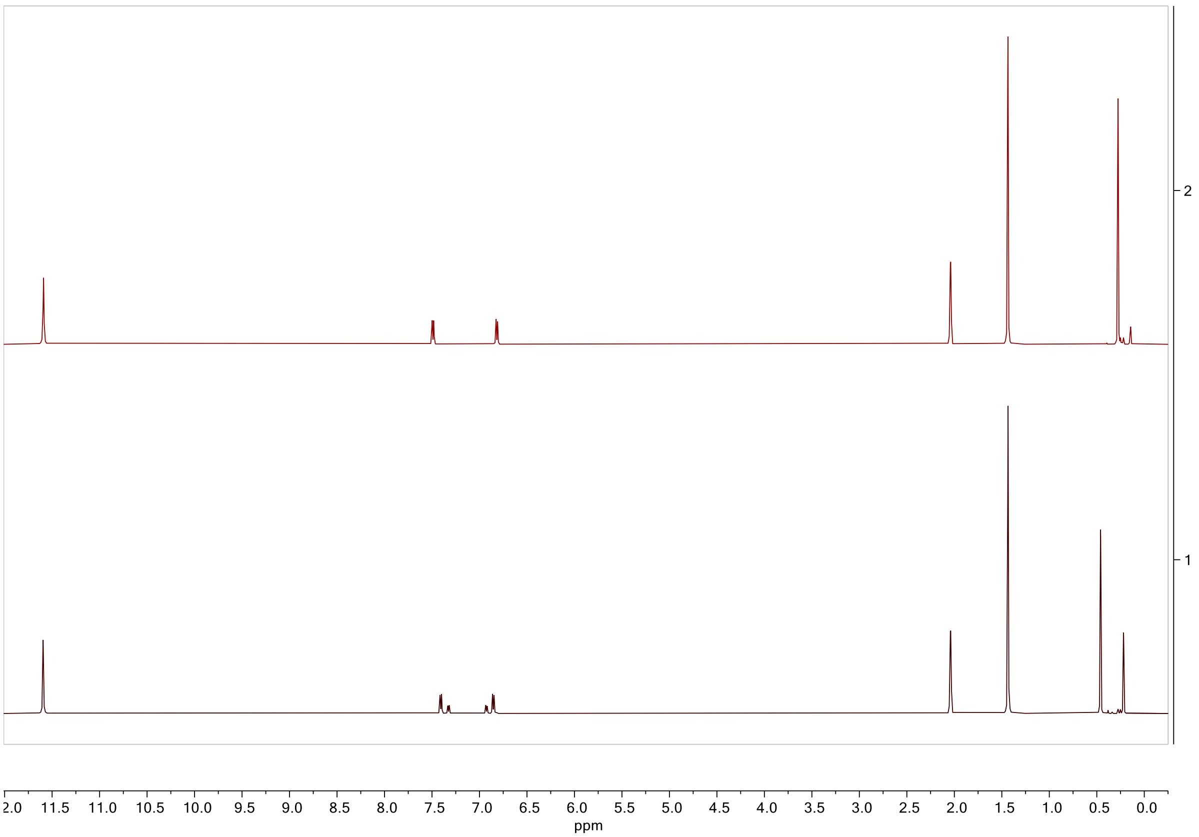

Figure S9.26.2. ^{1}H-NMR spectra (500 MHz, CD_3COOD) corresponding to entry SI-40. Bottom spectrum was recorded at start of the reaction, and top spectrum was recorded after 13 hours and 30 minutes of reaction time. The signal at 1.40 ppm corresponds to cyclohexane, which was used as an internal standard.

Section 9.27 – Entry SI-41

Reaction between **A** (R = *p*-NH_2) and **B** in C_6D_6. The ratio of **A:B** is 1:1. NH_4OAc was added at the start of this reaction.

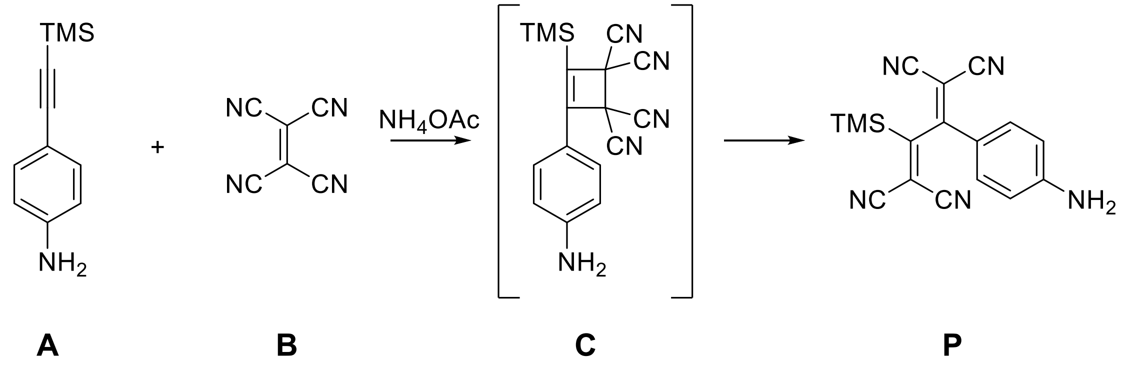

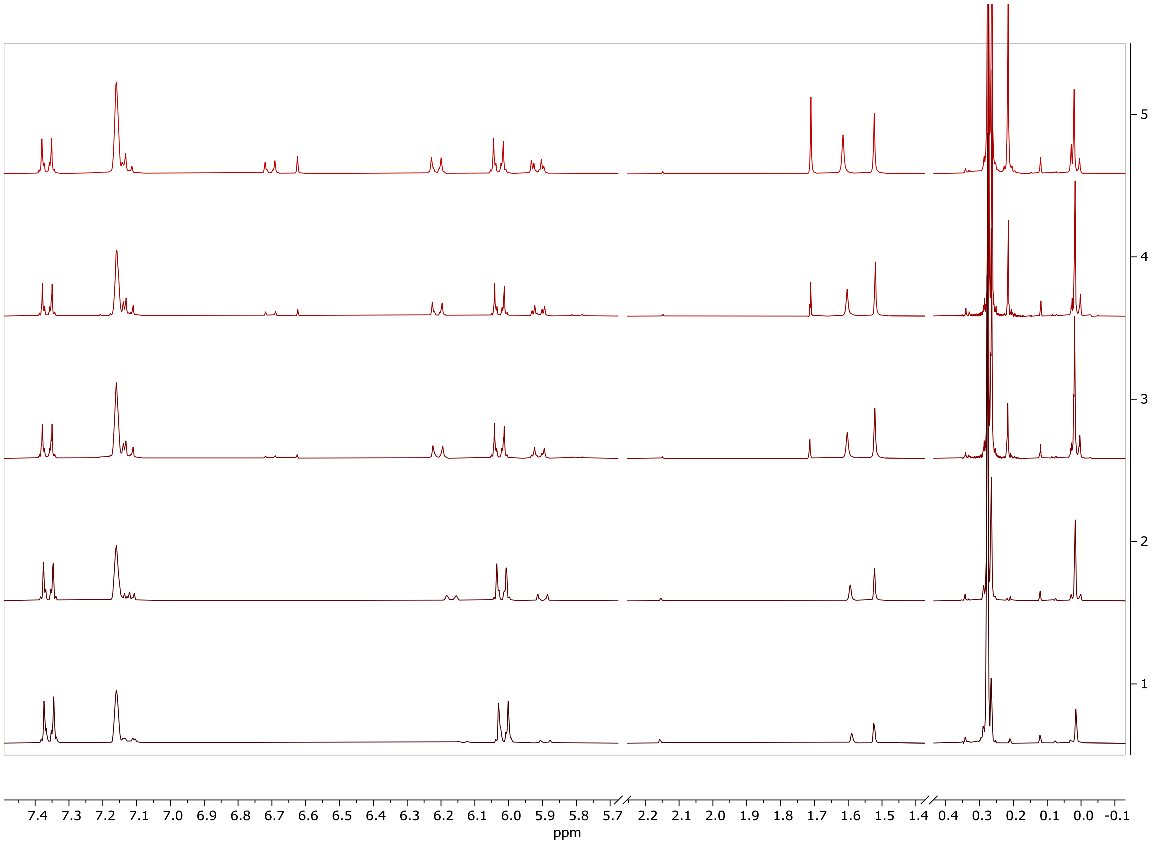

Figure S9.27.1. ^{1}H-NMR spectra (300 MHz, C_6D_6; selected regions shown) corresponding to entry SI-41. Stacked spectra of all ^{1}H NMR spectra recorded during the experiment. The spectra were recorded at 3 minutes, 15 minutes, 43 minutes, 75 minutes, and 145 minutes, respectively, starting from the bottom. Only the regions with signals are shown; intermediate regions were removed as indicated by the slashes.

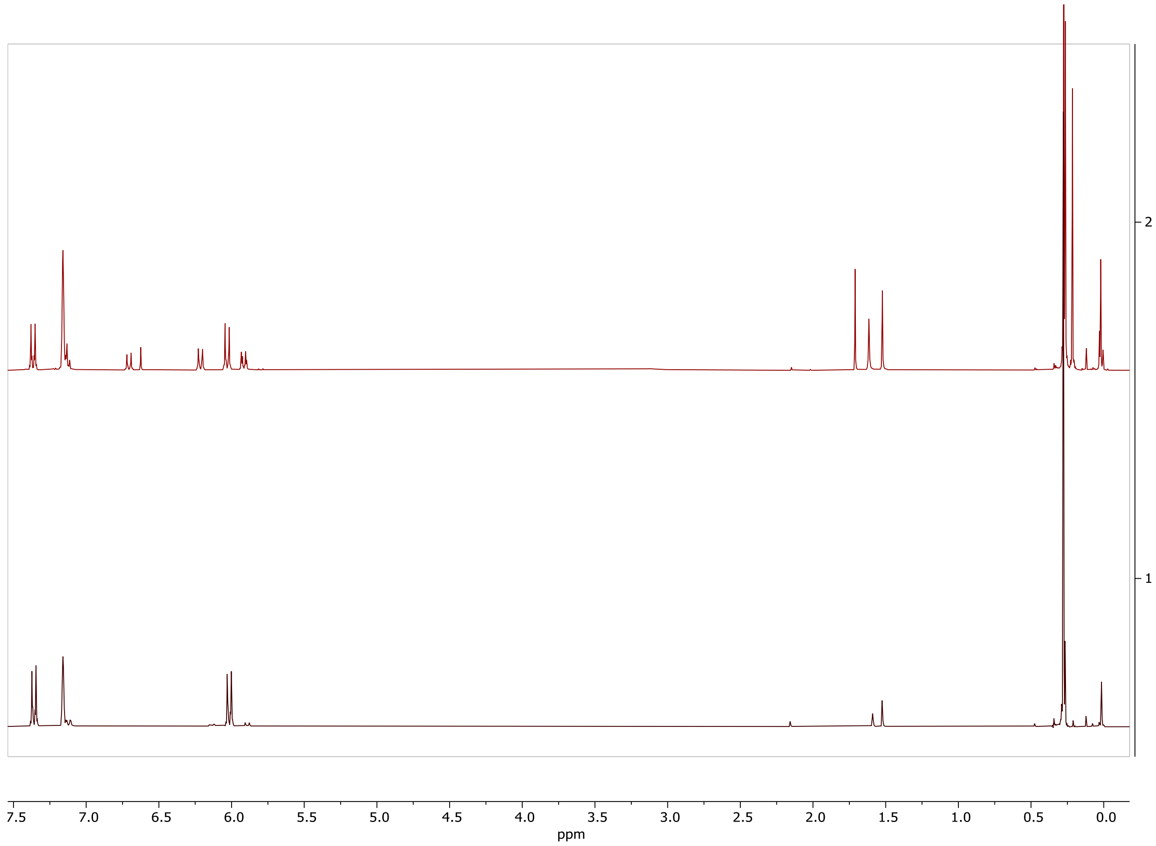

Figure S9.27.2. ^{1}H-NMR spectra (300 MHz, C_6D_6) corresponding to entry SI-41. Bottom spectrum was recorded at start of the reaction, and top spectrum was recorded after 145 minutes of reaction time.

Section 9.28 – Entry SI-42

Reaction between **A** (R = *p*-NH_2) and **B** in C_6D_6. The ratio of **A:B** is 1:1. Na_2CO_3 was added at the start of this reaction.

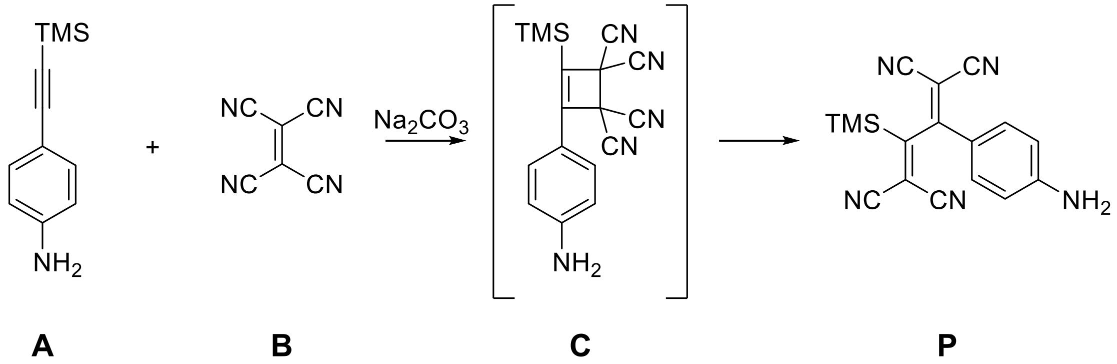

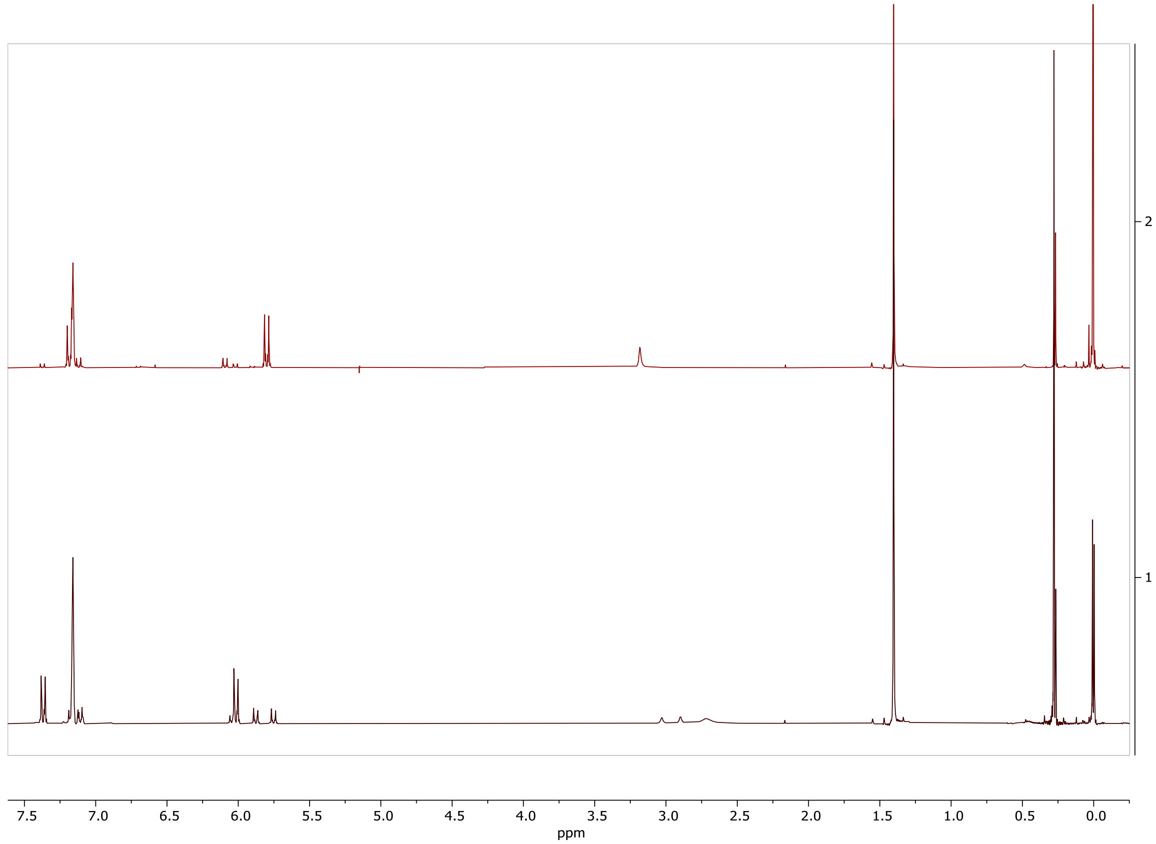

Figure S9.28.1. ¹H-NMR spectra (300 MHz, C_6D_6) corresponding to entry SI-42. Stacked spectra of the two ¹H-NMR spectra recorded during this experiment. Bottom spectrum was recorded after 2 hours of reaction time and top spectrum after 26 hours of reaction time. The signal at 1.4 ppm corresponds to cyclohexane, which was added as an internal standard.

Section 9.29 – Entry SI-43

Reaction between **A1** (R = *p*-NH_2) and **B** in the presence of **A2** (R = *p*-CN; not reacting) in C_6D_6. The ratio of **A1:A2:B** is 1:1:1.

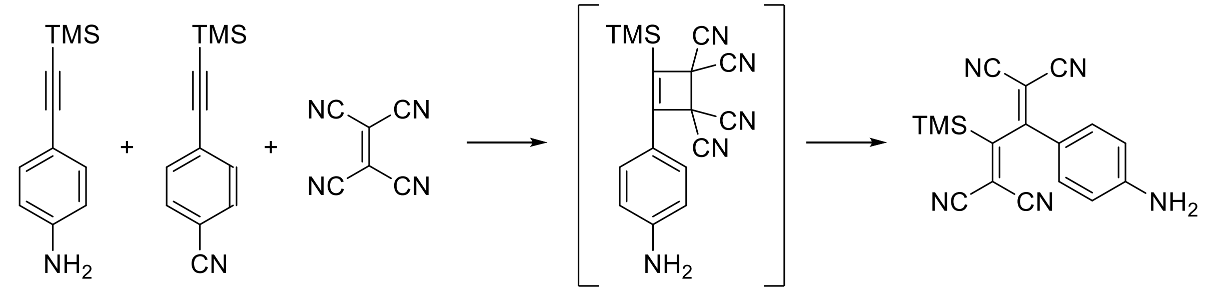

A1 **A2** **B** **C** **P**

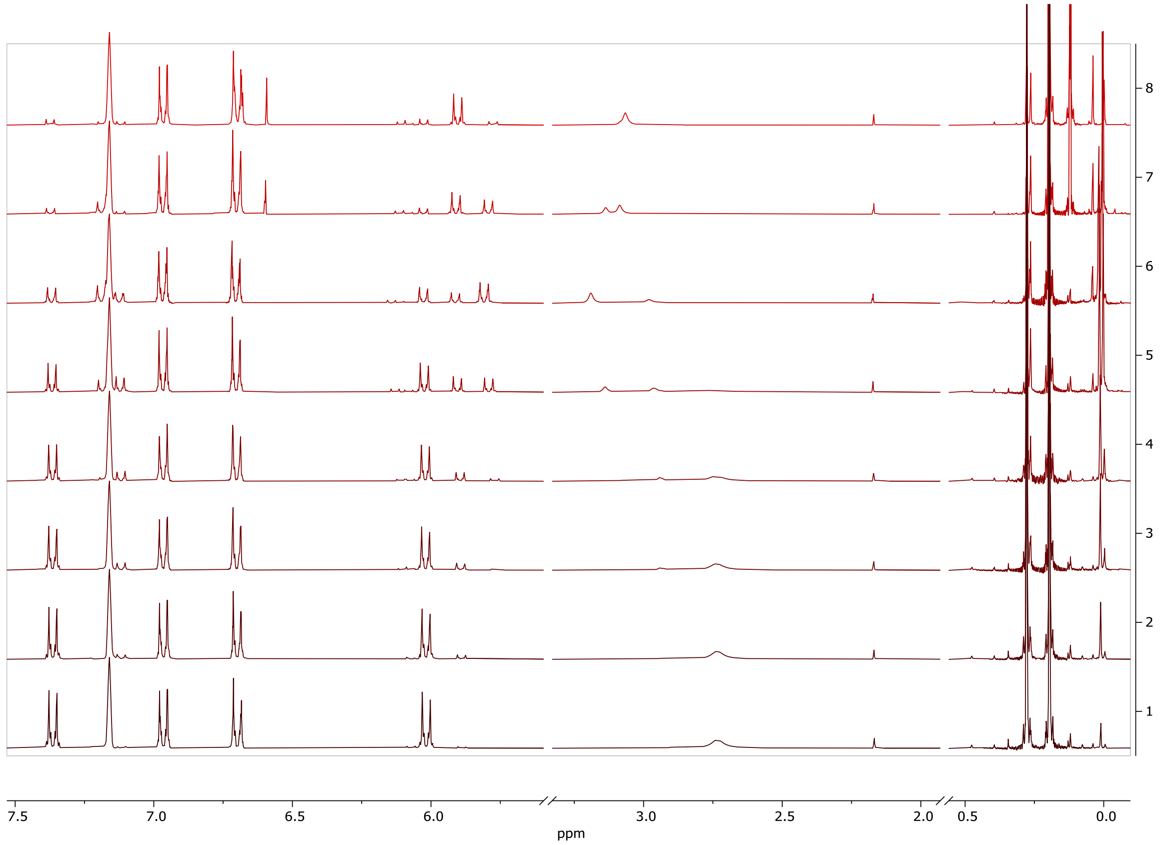

Figure S9.29.1. ^{1}H-NMR spectra (300 MHz, C_6D_6; selected regions shown) corresponding to entry SI-43. Stacked spectra of all ^{1}H NMR spectra recorded during the experiment. The spectra were recorded at 3 minutes, 13 minutes, 35 minutes, 50 minutes, and 130 minutes, 210 minutes, 27 hours and 73 hours, respectively, starting from the bottom. Only the regions with signals are shown; intermediate regions were removed as indicated by the slashes. **A1** reacted normally followed by a protodesilylation, but **A2** did not react at all.

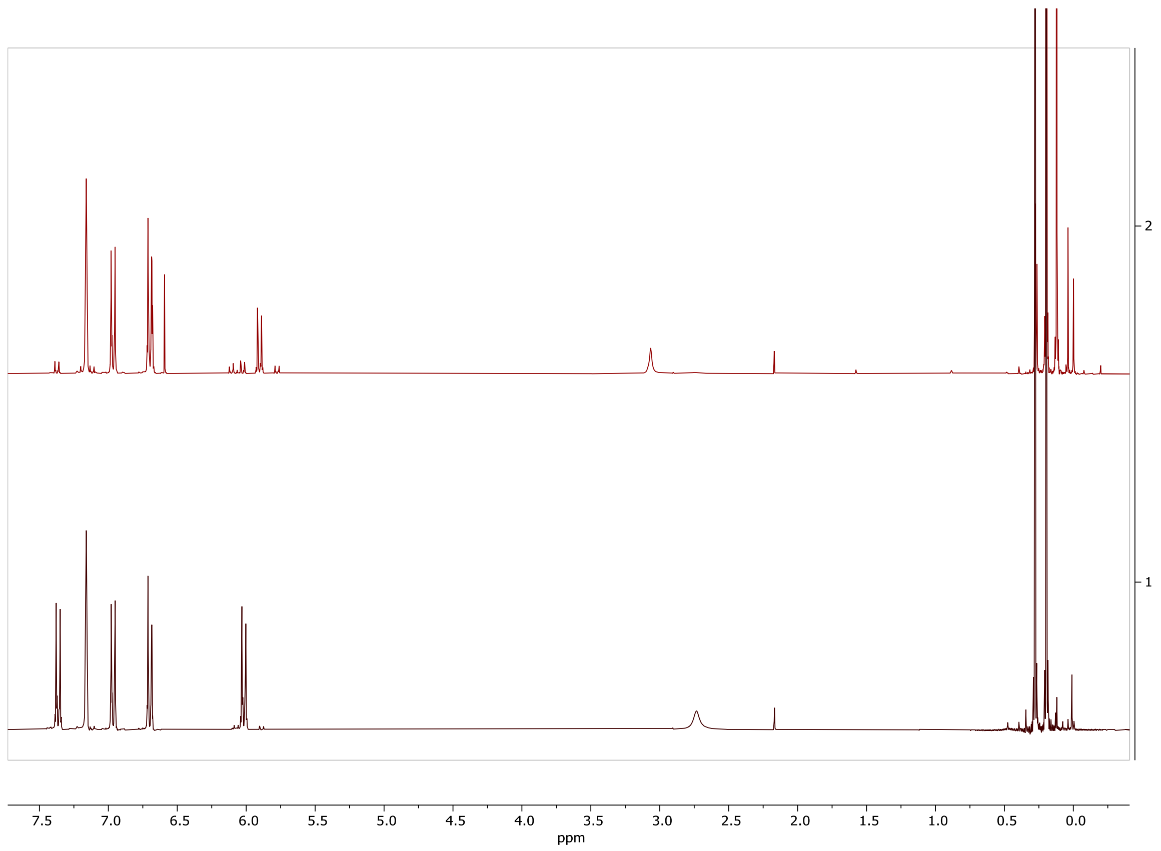

Figure S9.29.2. ^{1}H-NMR spectra (300 MHz, C_6D_6) corresponding to entry SI-43. Bottom spectrum was recorded at start of the reaction, and top spectrum was recorded after 73 hours of reaction time. **A1** reacted normally followed by a protodesilylation, but **A2** did not react at all.

Section 9.30 – Entry SI-44

Reaction between **A** (R = *p*-NH_2) and **B** in C_6D_6. The ratio of **A:Aniline:B** is 1:1:2. Concentration of **A** and **Aniline** = 0.022 M. Aniline was added at the start of this reaction.

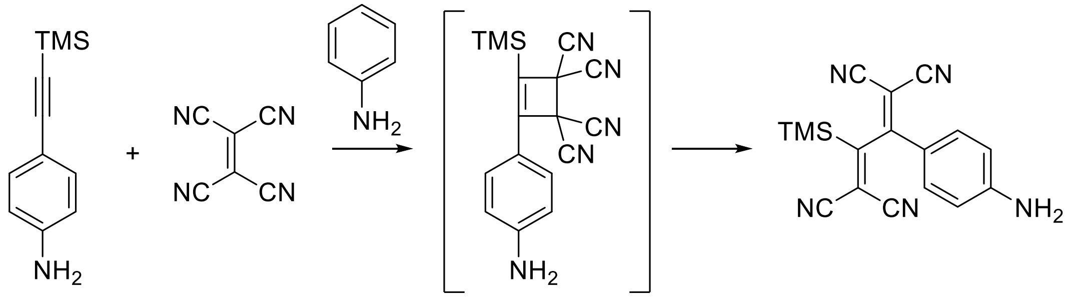

A **B** **C** **P**

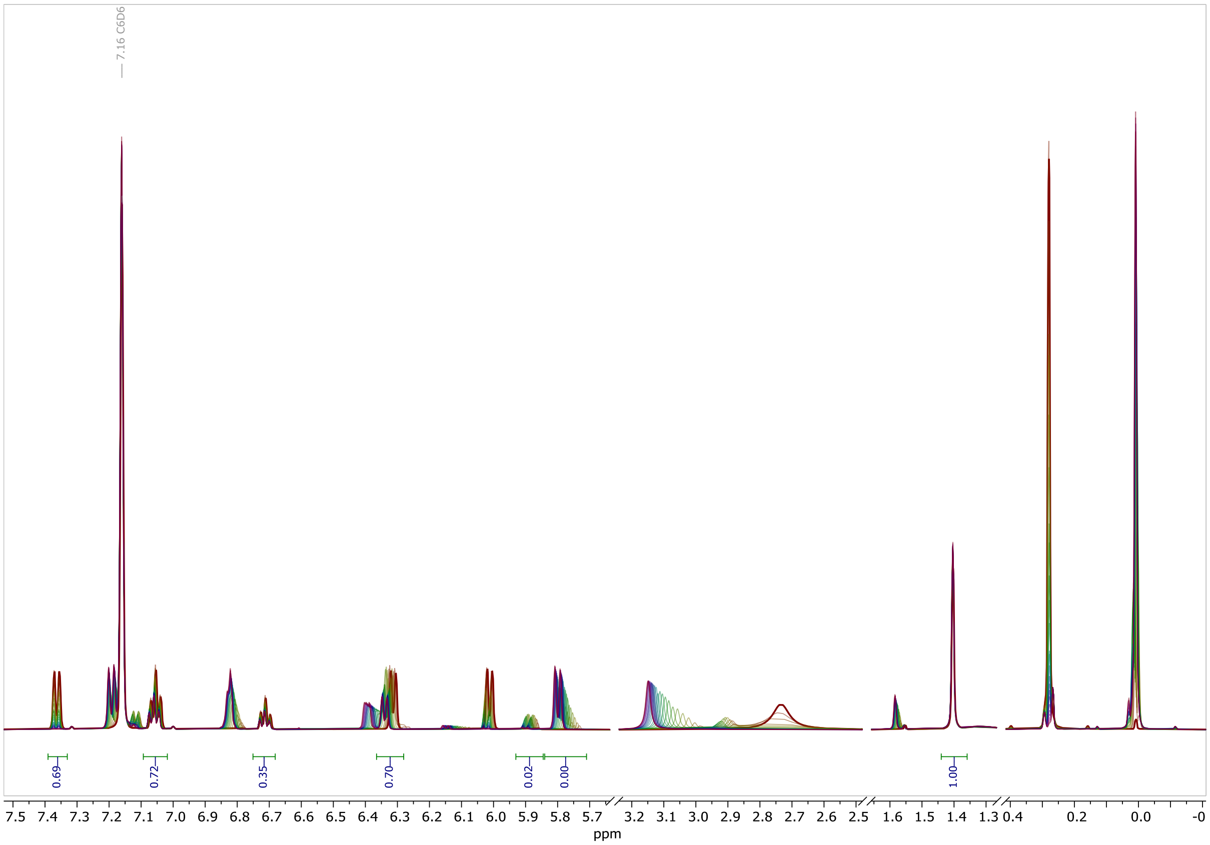

Figure S9.30.1. ^{1}H-NMR spectra (500 MHz, C_6D_6; selected regions shown) corresponding to entry SI-44. Superimposed spectra of all ^{1}H NMR spectra recorded during the experiment. The first spectrum recorded is colored red, and the last spectrum recorded is colored purple. The spectra were recorded every 25 minutes during the full duration of the experiment. Only the regions with signals are shown; intermediate regions were removed as indicated by the slashes. The signal at 1.4 ppm is cyclohexane, which was used as an internal standard.

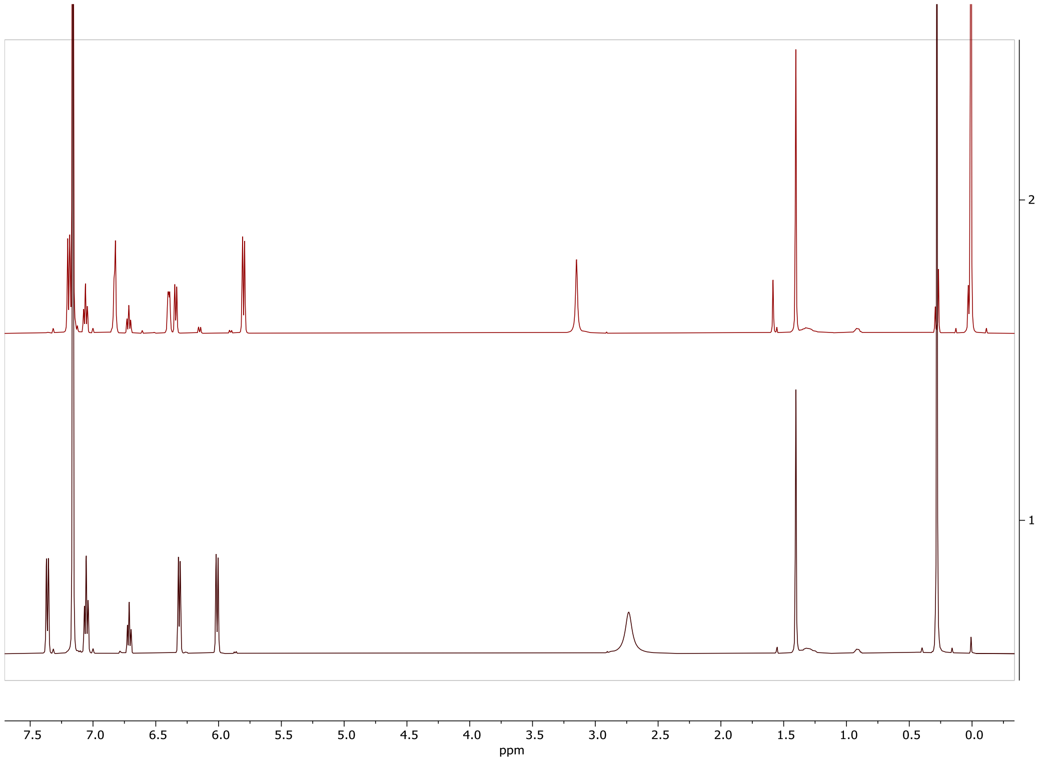

Figure S9.30.2. ^{1}H-NMR spectra (500 MHz, C_6D_6) corresponding to entry SI-44. Bottom spectrum was recorded at start of the reaction, and top spectrum was recorded after 12 hours and 10 minutes of reaction time. The signal at 1.4 ppm is cyclohexane, which was used as an internal standard.

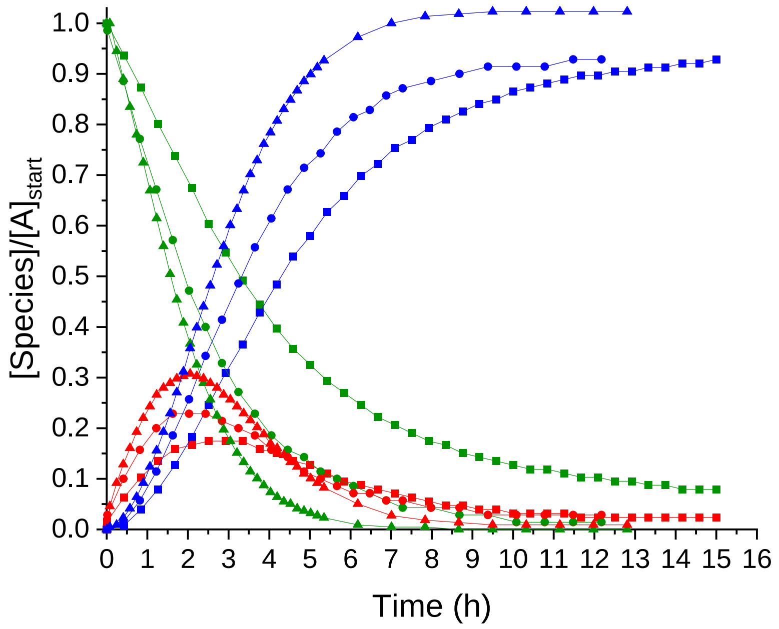

Figure S9.30.3. Comparison of Entry SI-23, Entry SI-24, and Entry SI-44. The y-axis is made by taking the concentration of a given species at any given time and dividing with the starting concentration of compound **A** to make the two experiments comparable. Entry SI-23 has square data points and Entry SI-44 has circle data points and Entry SI-24 has triangular data points.

Section 9.31 – Entry SI-45

Reaction between **A** (R = *p*-NH_2) and **B** in CD_2Cl_2. The ratio of **A:B** is 1:1. Concentration of **A** and **B** = 0.038 M.

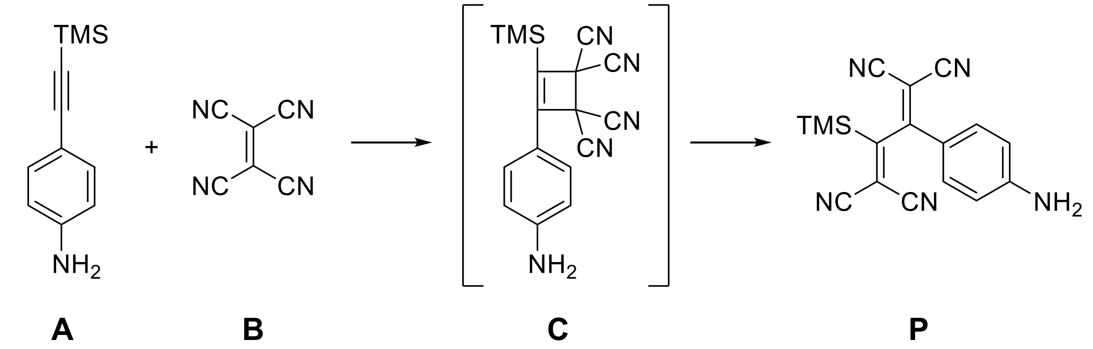

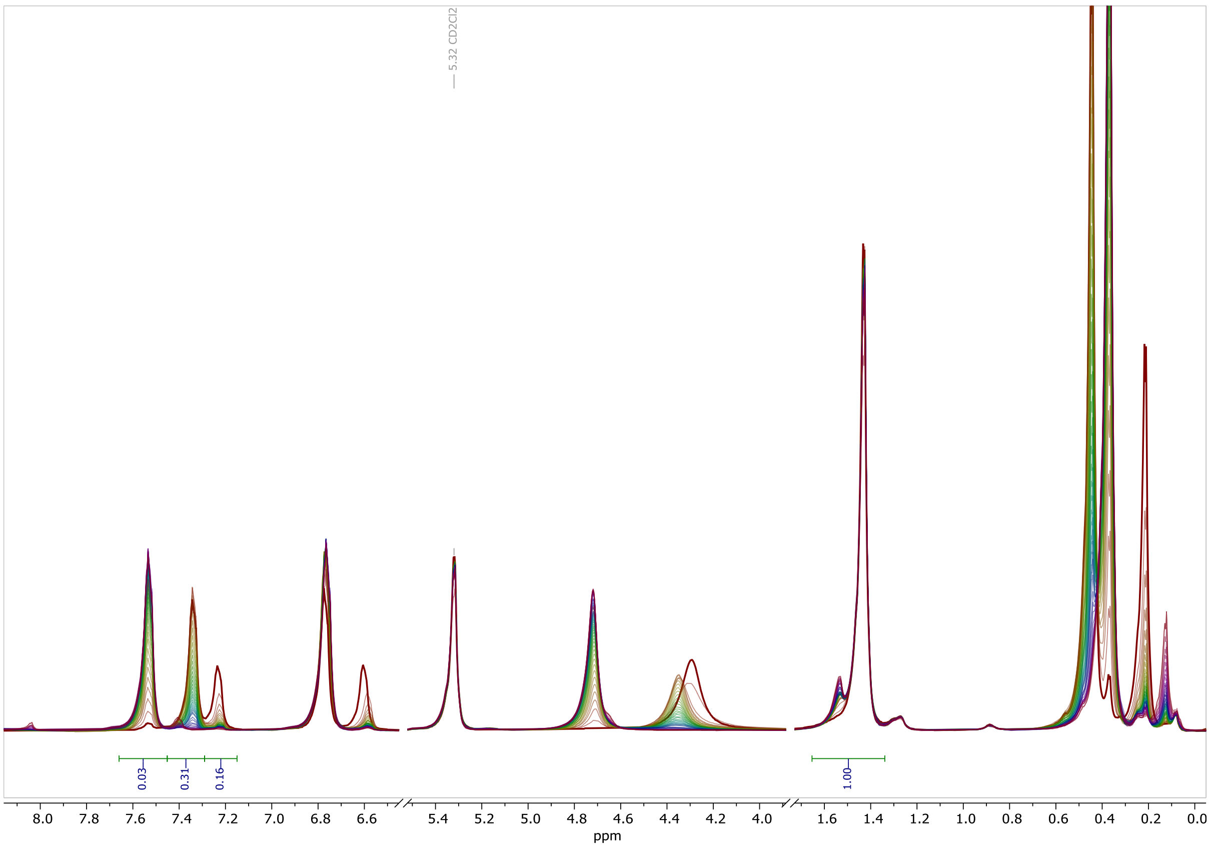

Figure S9.31.1. ^{1}H-NMR spectra (500 MHz, CD_2Cl_2; selected regions shown) corresponding to entry SI-45. Superimposed spectra of all ^{1}H NMR spectra recorded during the experiment. The first spectrum recorded is colored red, and the last spectrum recorded is colored purple. The spectra were recorded every 4 minutes for 3 hours, then every 15 minutes for 2 hours, then every 40 minutes for 2 hours, and lastly every 75 minutes for the remaining duration of the experiment. Only the regions with signals are shown; intermediate regions were removed as indicated by the slashes. The signal at 1.4 ppm is cyclohexane, which was used as an internal standard.

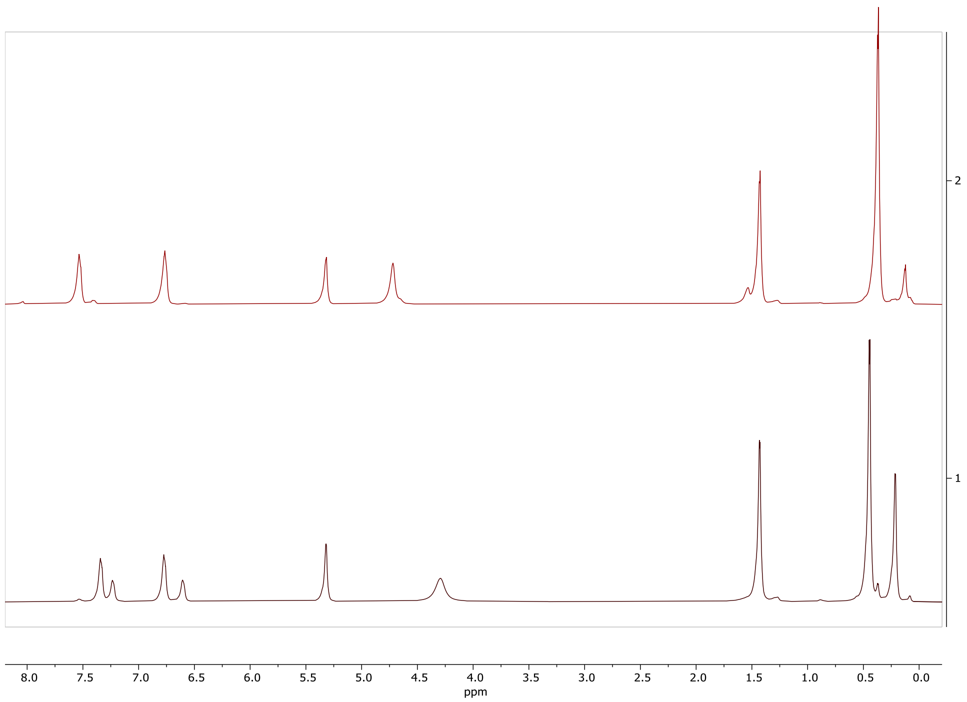

Figure S9.31.2. ^{1}H-NMR spectra (500 MHz, CD_2Cl_2) corresponding to entry SI-45. Bottom spectrum was recorded at start of the reaction, and top spectrum was recorded after 10 hours and 55 minutes of reaction time. In the bottom spectrum (first spectrum), the intermediate had formed almost immediately explaining the two sets of aromatic protons. The signal at 1.4 ppm is cyclohexane, which was used as an internal standard.

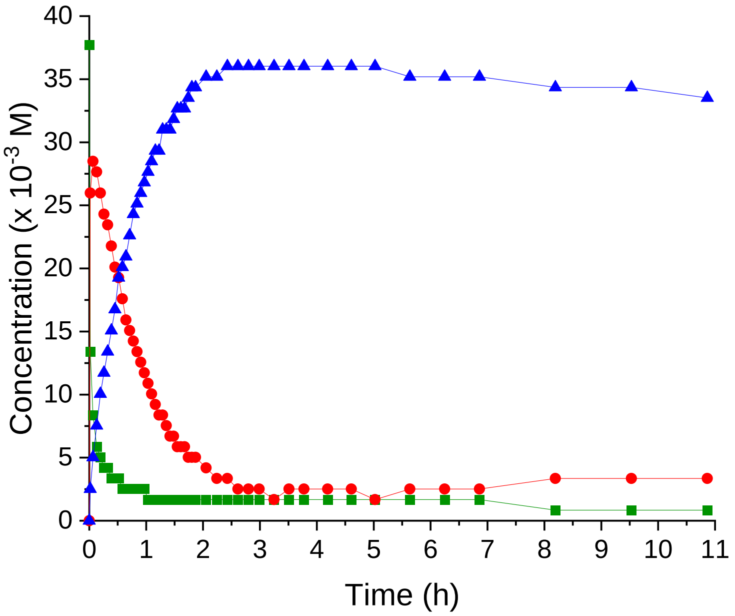

Figure S9.31.3. Concentrations over time of each species in the reaction. The concentrations are calculated from the ^{1}H-NMR by using cyclohexane as an internal standard. Intermediate **C** is formed almost instantaneously.

Section 9.32 – Entry SI-46

Reaction between **A** (R = *p*-NH_2) and **B** in CD_2Cl_2/C_6D_6. The ratio of **A:B** is 1:1. Concentration of **A** and **B** after C_6D_6 was added = 0.029 M. C_6D_6 was added 5 minutes after mixture of compounds **A** and **B**.

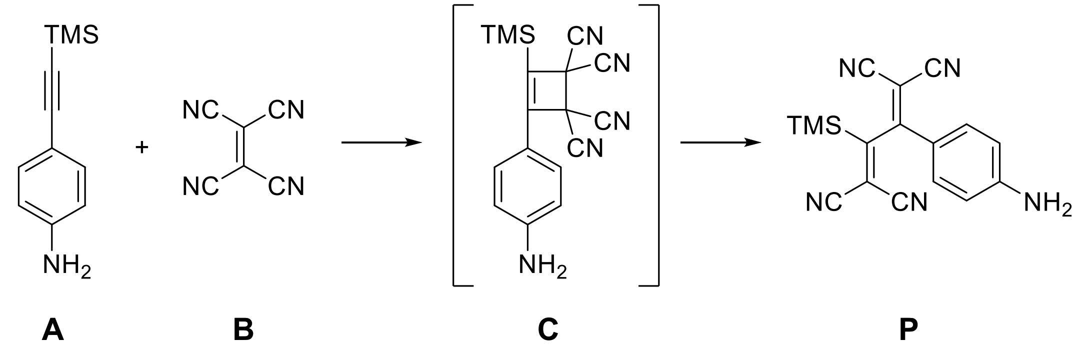

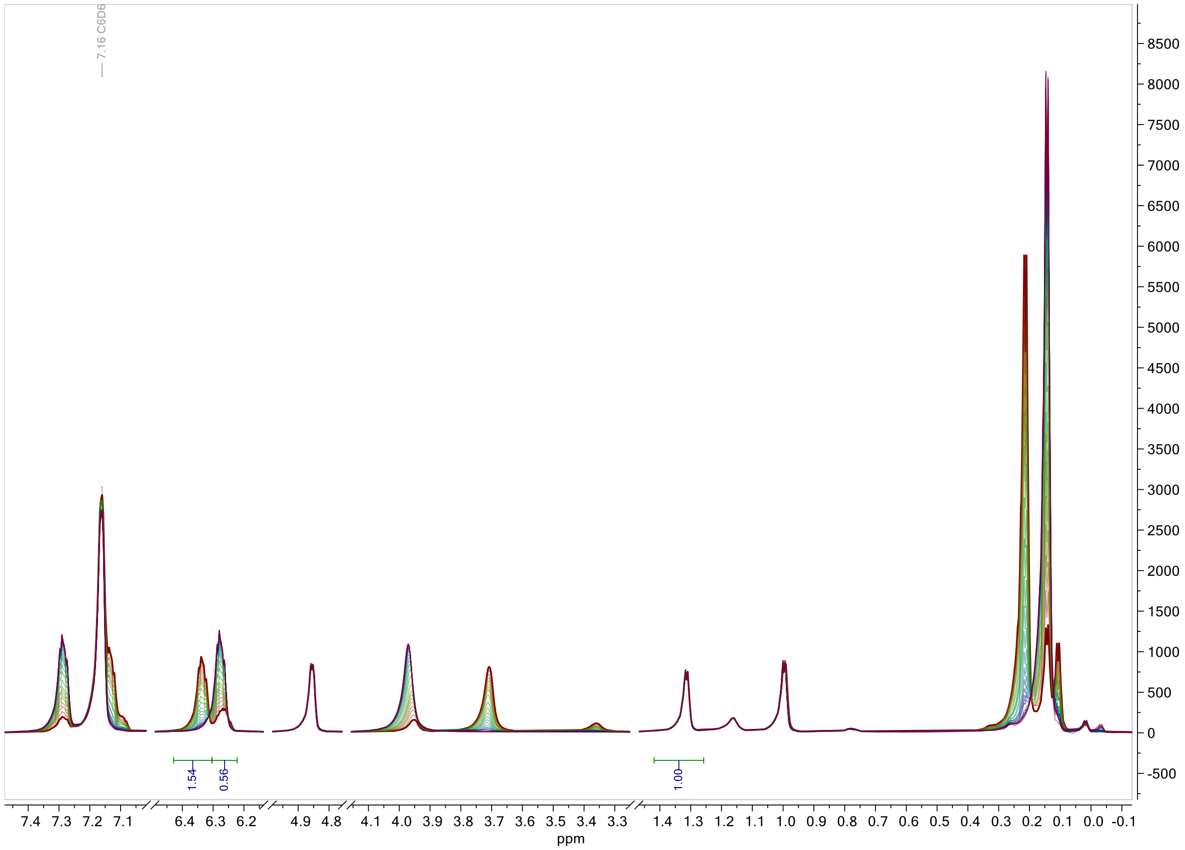

Figure S9.32.1. ^{1}H-NMR spectra (500 MHz, CD_2Cl_2/C_6D_6; selected regions shown) corresponding to entry SI-46. Superimposed spectra of all ^{1}H NMR spectra recorded during the experiment. The first spectrum recorded is colored red, and the last spectrum recorded is colored purple. The spectra were recorded every 4 minutes for 30 minutes, then every 15 minutes for 3 hours, and lastly every 35 minutes for 4.5 hours. Only the regions with signals are shown; intermediate regions were removed as indicated by the slashes. The signal at 1.32 ppm is cyclohexane, which was used as an internal standard. Normally the peak for cyclohexane is at 1.4 ppm in pure C_6D_6 but due to the solvent being a mixture it is slightly shifted.

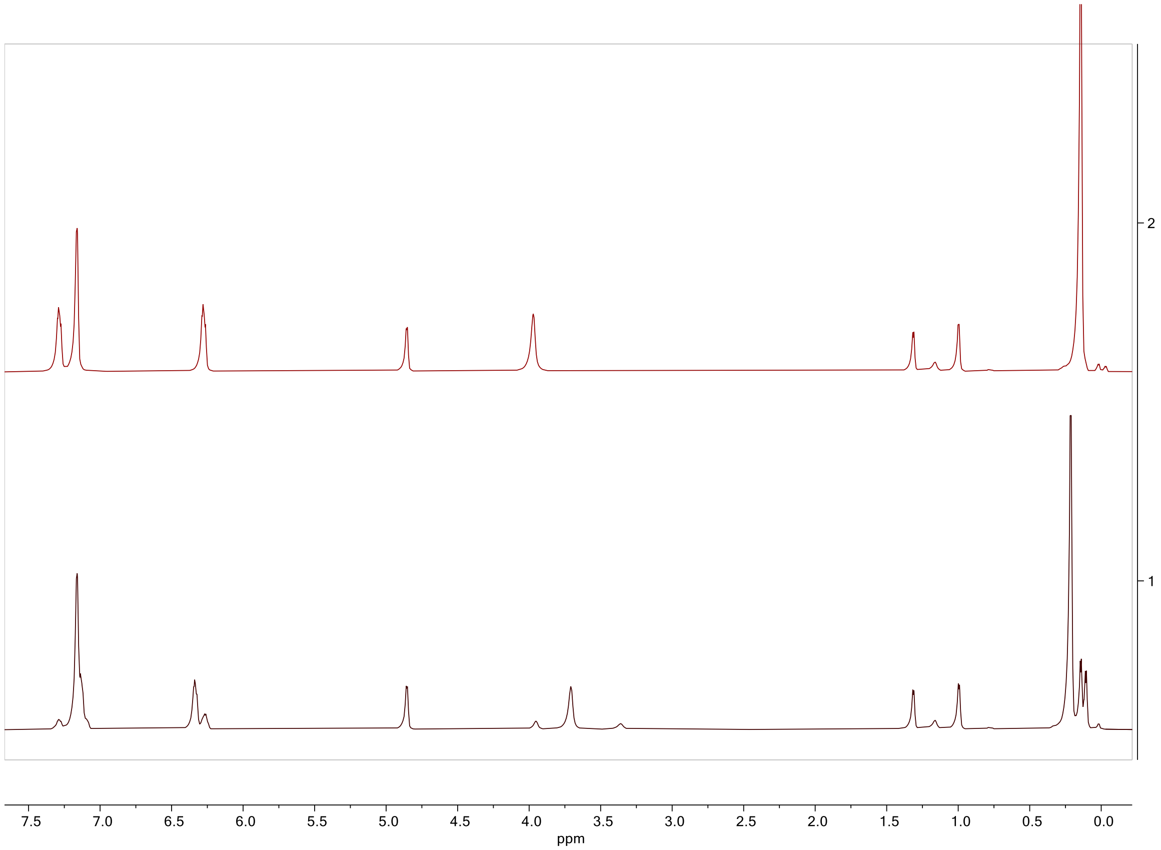

Figure S9.32.2. ^{1}H-NMR spectra (500 MHz, CD_2Cl_2/C_6D_6) corresponding to entry SI-46. Bottom spectrum was recorded directly after the addition of C_6D_6, which corresponds to 5 minutes of reaction time, and the top spectrum was recorded after 8 hours and 10 minutes of reaction time. In the bottom spectrum, the starting material **A** had already transformed into intermediate **C** and to a small amount of product **P**. The signal at 1.32 ppm is cyclohexane. Normally the peak for cyclohexane is at 1.4 ppm in pure C_6D_6 but due to the solvent being a mixture it is slightly shifted.

Section 9.33 – Entry SI-47

Reaction between **A** (R = *p*-NH_2) and **B** in C_6D_6. The ratio of **A:B** is 1:1. Concentration of **A** and **B** = 0.033 M. An additional amount of C_6D_6 was added 24 hours after mixing compounds **A** and **B** to see the effect of dilution.

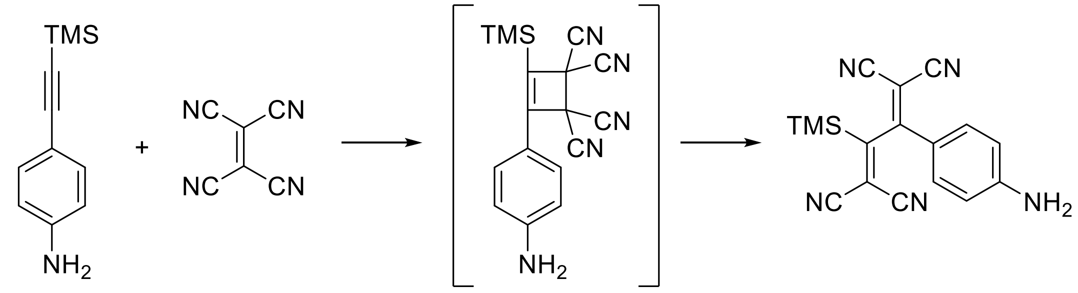

A **B** **C** **P**

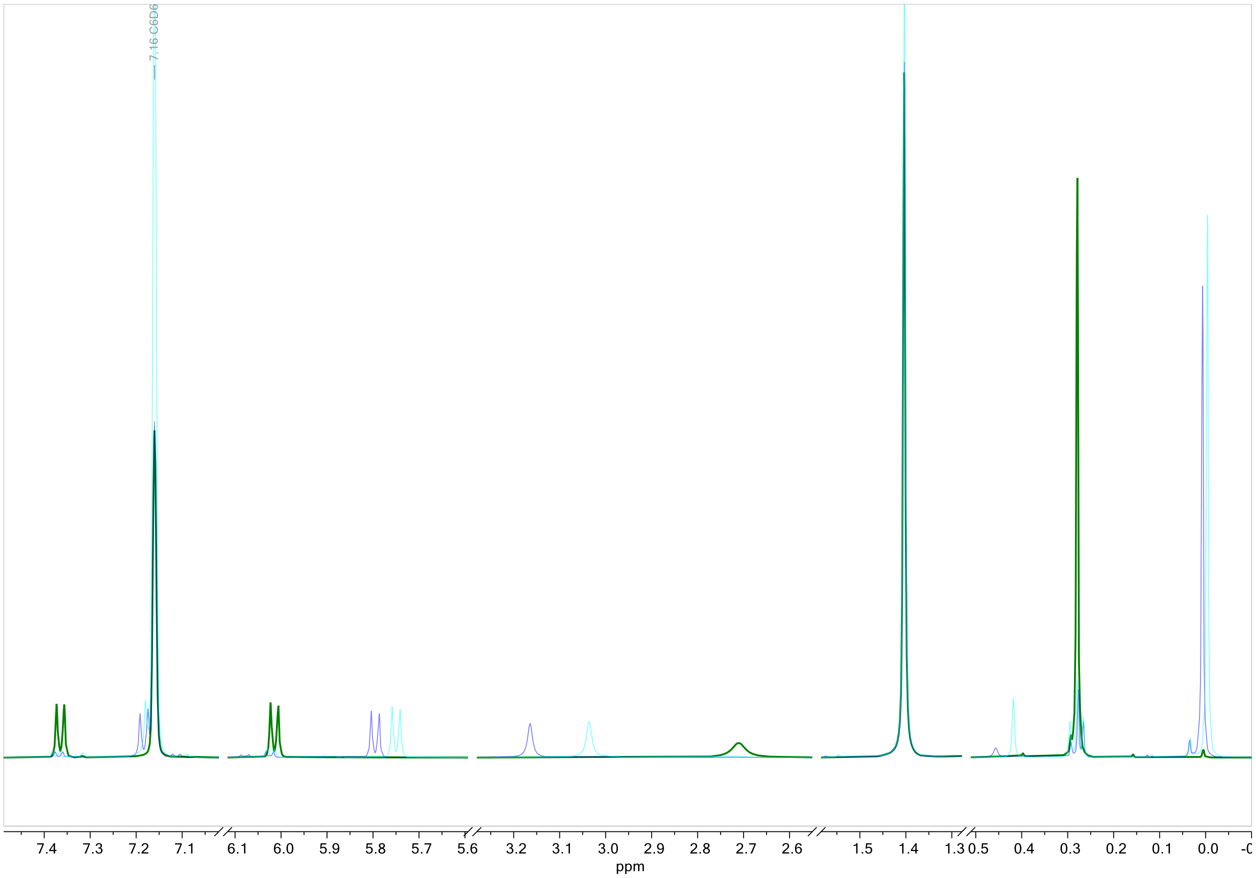

Figure S9.33.1. ^{1}H-NMR spectra (500 MHz, C_6D_6; selected regions shown) corresponding to entry SI-47. ^{1}H NMR spectrum recorded at the start of the experiment is shown in green.; a spectrum recorded after 24 hours in blue and a spectrum after dilution of the sample in cyan. Only the regions with signals are shown; intermediate regions were removed as indicated by the slashes. The signal at 1.4 ppm is cyclohexane, which was used as an internal standard.

Section 9.34 – Entry SI-48

Reaction between **A** (R = *p*-NH_2) and **B** in C_6D_6. The ratio of **A:B** is 1:5.

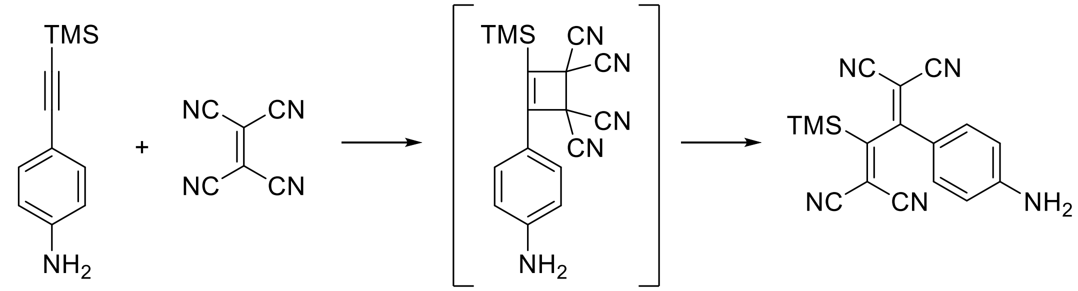

A **B** **C** **P**

COSY spectroscopic data for this experiment can be found in Section 7.3, starting from page S13.

Section 9.35 – Entry SI-49

Reaction between **A** (R = *p*-NH_2) and **B** in C_6D_6. The ratio of **A:B** is 1:5.

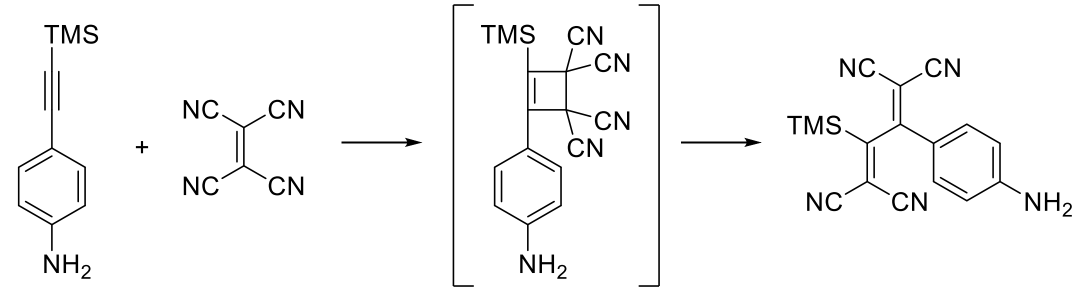

A **B** **C** **P**

HMBC spectroscopic data for this experiment can be found in Section 7.3, starting from page S13.

Section 9.36 – Entry SI-50

Reaction between **A** (R = *p*-NH_2) and **B** in C_6D_6. The ratio of **A:B** is 1:1. Concentration of **A** and **B** = 0.033 M. Another equivalent of **B** was added 24 hours after mixing of compounds **A** and **B** to observe a potential complexation of **B** and **P** and its effect.

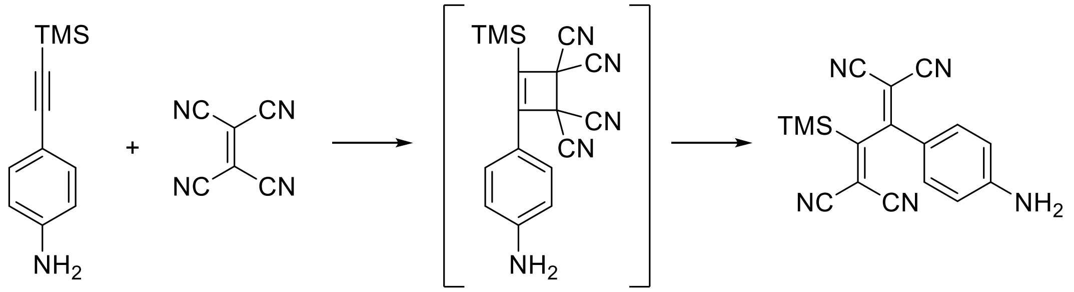

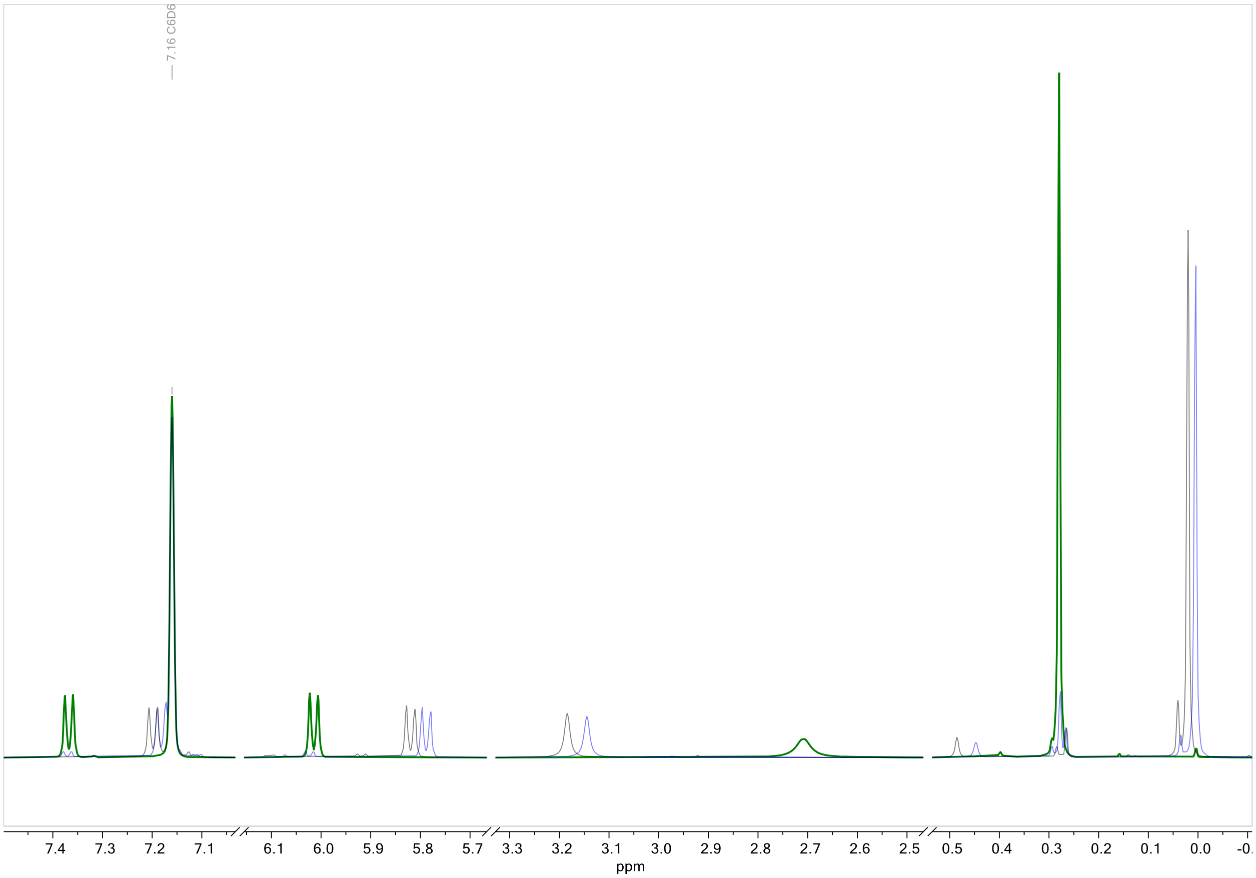

Figure S9.36.1. ^{1}H-NMR spectra (500 MHz, C_6D_6; selected regions shown) corresponding to entry SI-50. ^{1}H NMR spectrum recorded at the start of the experiment is shown in green.; a spectrum recorded after 24 hours in blue and a spectrum after addition of more **B** in black. Only the regions with signals are shown; intermediate regions were removed as indicated by the slashes.

Section 9.37 – Entry SI-51

Reaction between **A** (R = *p*-NH_2) and **B** in C_6D_6 containing product **P**. The ratio of **A:B**:**P** is 1:1:1. A sample of **A** and **B** was prepared in a 1:1 ratio of the two, which corresponds to the experiment shown in section 9.7 – Entry 23, page S48. After 24 hours another equivalent of both **A** and **B** was added to the reaction. The spectroscopic data below shows the result of the experiment after the additional equivalent of starting materials was added. The sample was then diluted to lower the concentration of the formed product to that of before the extra equivalent of **A** and **B** was added to observe the change in chemical shift.

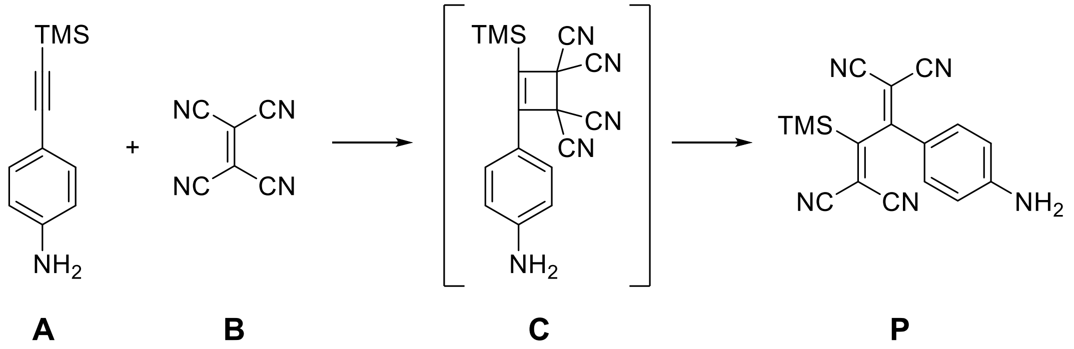

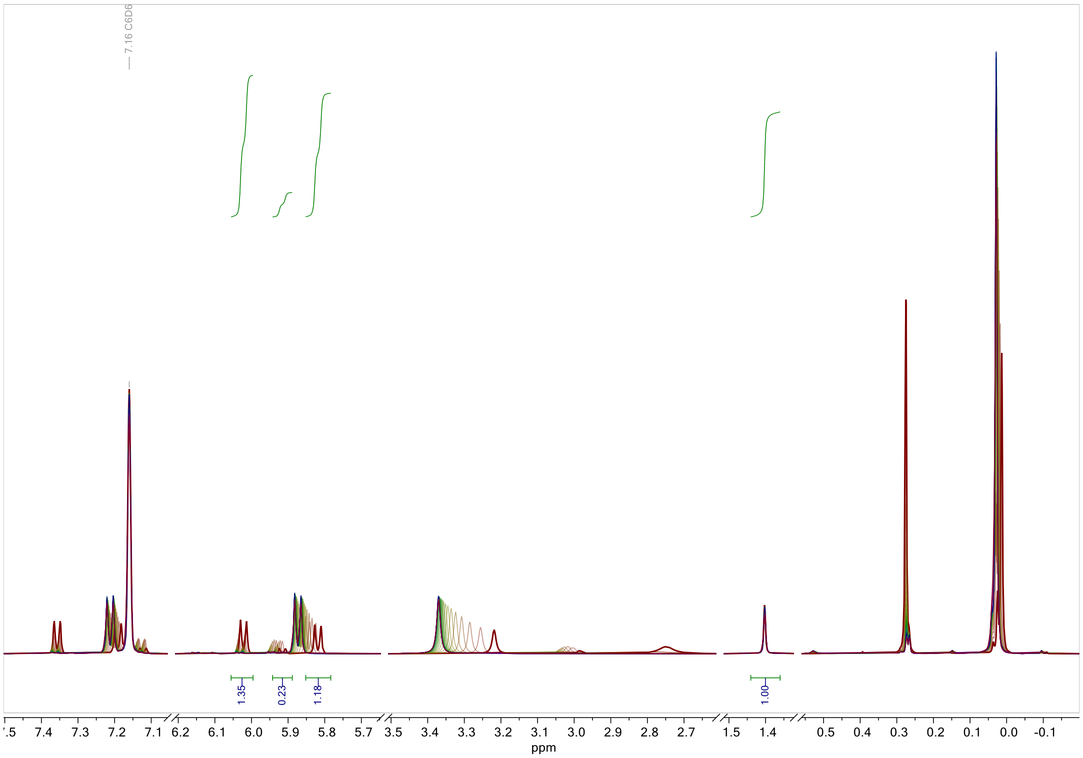

Figure S9.37.1. ^{1}H-NMR spectra (500 MHz, C_6D_6; selected regions shown) corresponding to entry SI-51. Superimposed spectra of all ^{1}H NMR spectra recorded during the experiment. The first spectrum recorded is colored red, and the last spectrum recorded is colored purple. The spectra were recorded precisely every 25 minutes for 15 hours. Only the regions with signals are shown; intermediate regions were removed as indicated by the slashes. The signal at 1.4 ppm corresponds to cyclohexane, which was used as an internal standard.

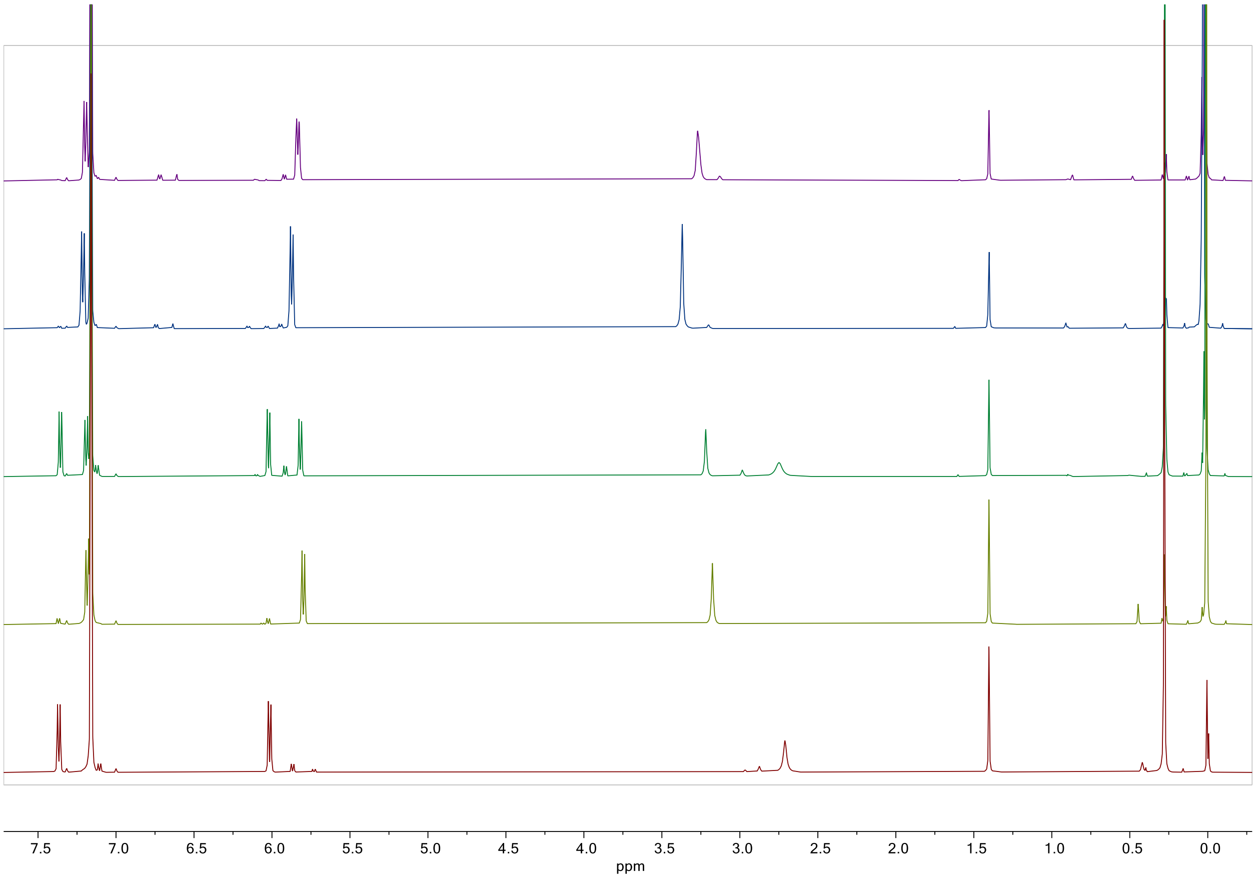

Figure S9.37.2. ^{1}H-NMR spectra (500 MHz, C_6D_6) corresponding to entry SI-23 and entry SI-51. Stacked spectra of all relevant spectra for this experiment. Bottom spectrum corresponds to the first recorded spectrum of Entry SI-23 colored red. Yellow spectrum corresponds to the last spectrum recorded of Entry SI-23. Green spectrum corresponds to the first spectrum of Entry SI-51 after an additional equivalent of **A** and **B** was added. Blue spectrum corresponds to the last spectrum after an additional equivalent of **A** and **B** was added. The purple spectrum was recorded after dilution with more C_6D_6. The aryl proton around 6 ppm changes resonance frequency during the experiment, and a graph showing this can be seen in the article. The signal at 1.4 ppm corresponds to cyclohexane, which was used as an internal standard.

Section 9.38 – Entry SI-52

Reaction between **A** (R = *p*-NH_2) and **B** in C_6D_6 containing product **P**. The ratio of **A**:**B**:**P** is 1:1:0.2. Concentration of **A** and **B** = 0.0272 M.

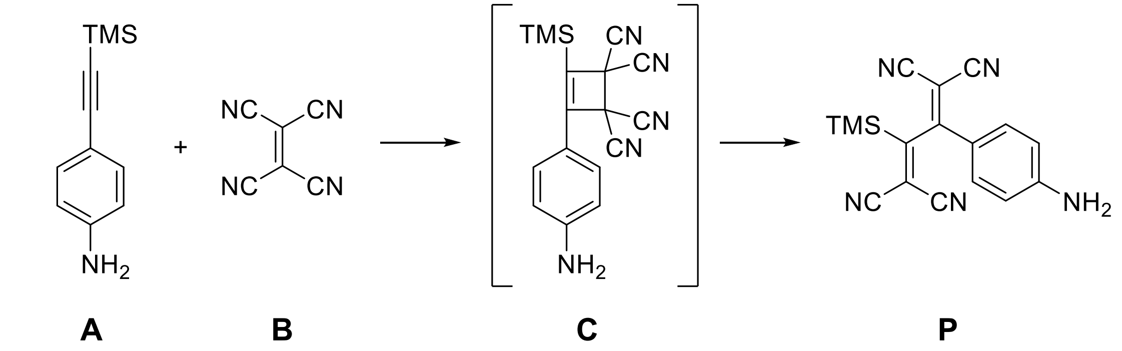

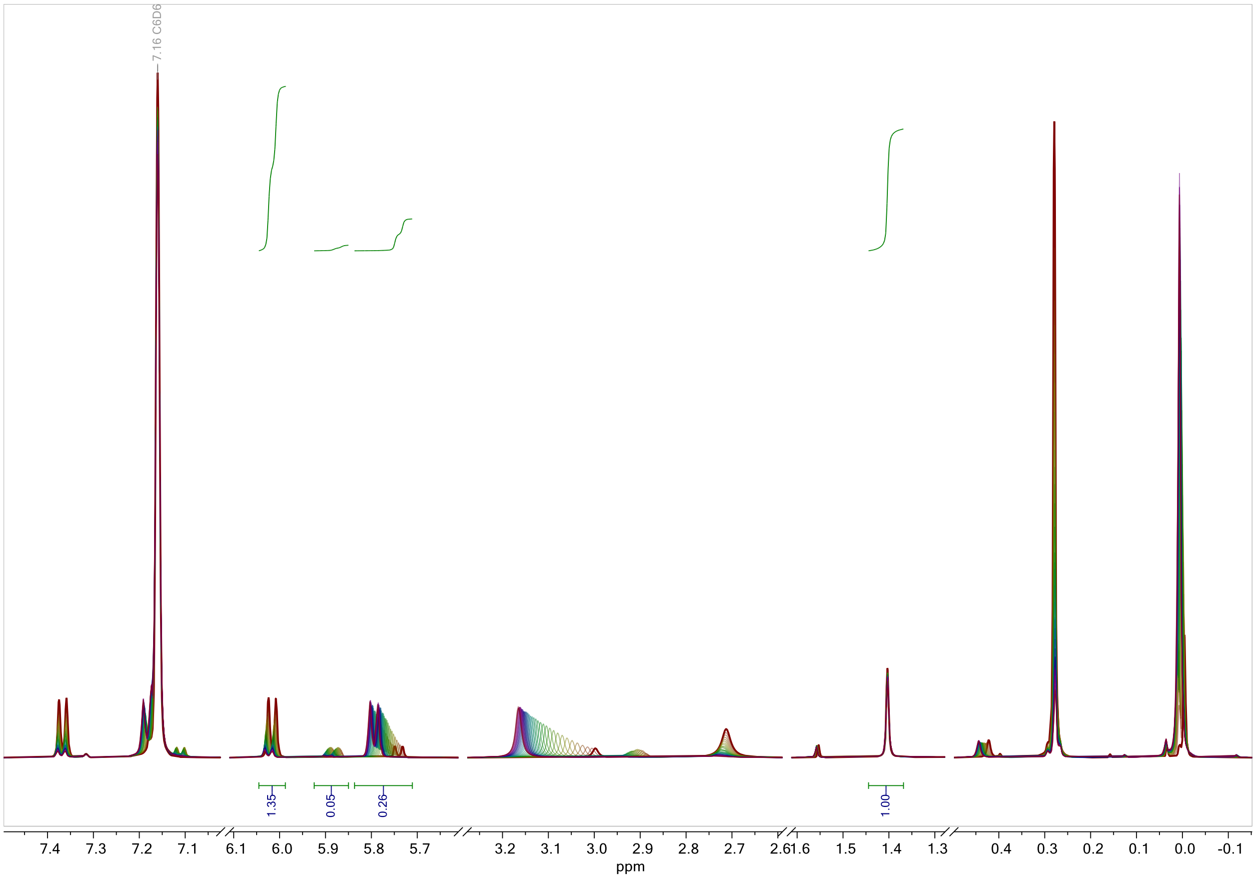

Figure S9.38.1. ^{1}H-NMR spectra (500 MHz, C_6D_6; selected regions shown) corresponding to entry SI-52. Superimposed spectrum of all ^{1}H NMR spectra recorded during the experiment. The first spectrum recorded is colored red, and the last spectrum recorded is colored purple. The spectra were recorded precisely every 20 minutes for 12.5 hours. Only the regions with signals are shown; intermediate regions were removed as indicated by the slashes. The signal at 1.4 ppm corresponds to cyclohexane, which was used as an internal standard.

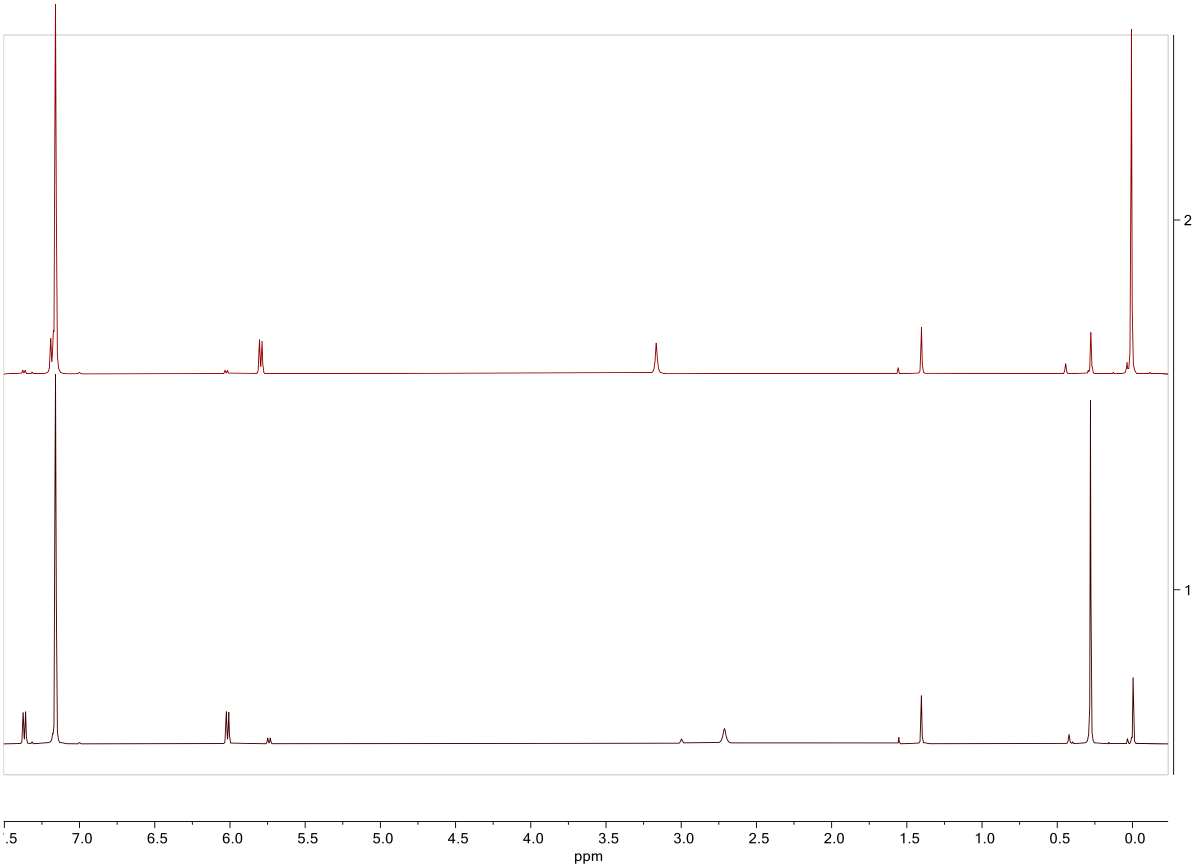

Figure S9.38.2. ^{1}H-NMR spectra (500 MHz, C_6D_6) corresponding to entry SI-52. Bottom spectrum was recorded at start of the reaction, and top spectrum was recorded after 12.5 hours of reaction time. The signal at 1.4 ppm corresponds to cyclohexane, which was used as an internal standard.

Section 9.39 – Entry SI-53

Reaction between **A** (R = *p*-NH_2) and **B** in C_6D_6 containing product **P**. The ratio of **A**:**B**:**P** is 1:1:0.4. Concentration of **A** and **B** = 0.0272 M

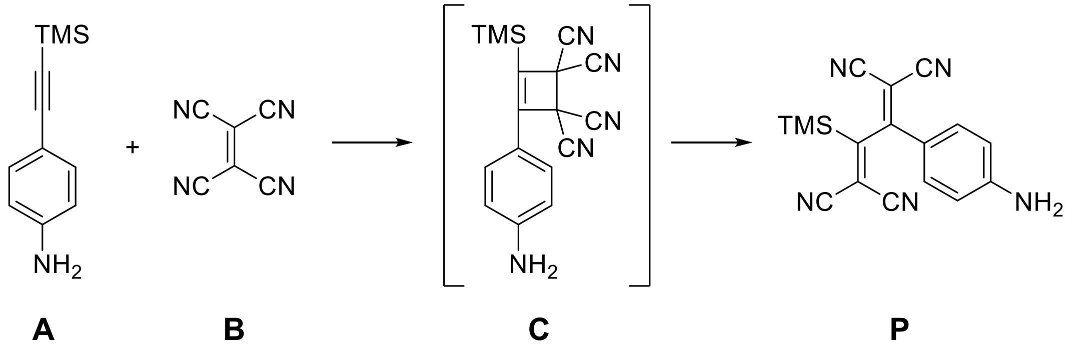

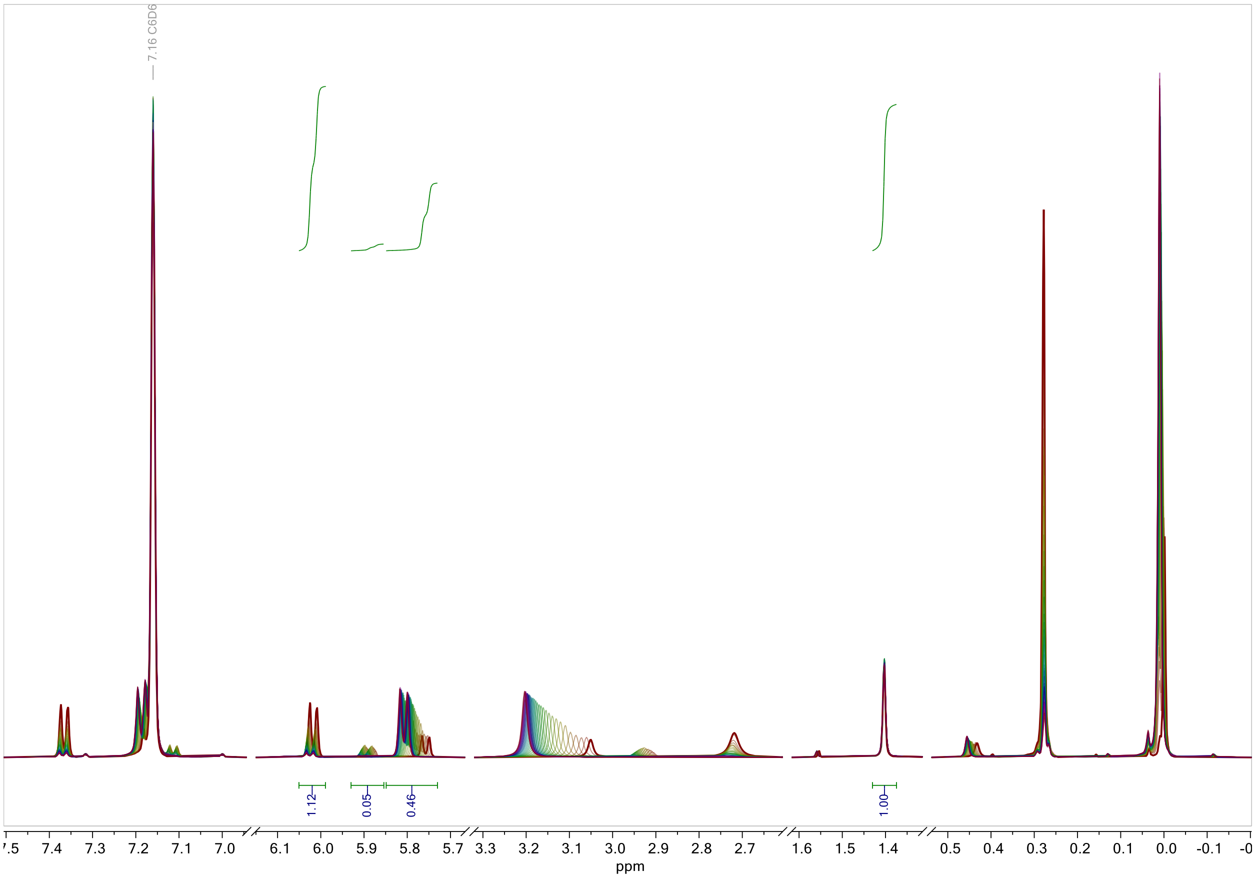

Figure S9.39.1. ^{1}H-NMR spectra (500 MHz, C_6D_6; selected regions shown) corresponding to entry SI-53. Superimposed spectra of all ^{1}H NMR spectra recorded during the experiment. The first spectrum recorded is colored red, and the last spectrum recorded is colored purple. The spectra were recorded precisely every 20 minutes for 12.5 hours. Only the regions with signals are shown; intermediate regions were removed as indicated by the slashes. The signal at 1.4 ppm corresponds to cyclohexane, which was used as an internal standard.

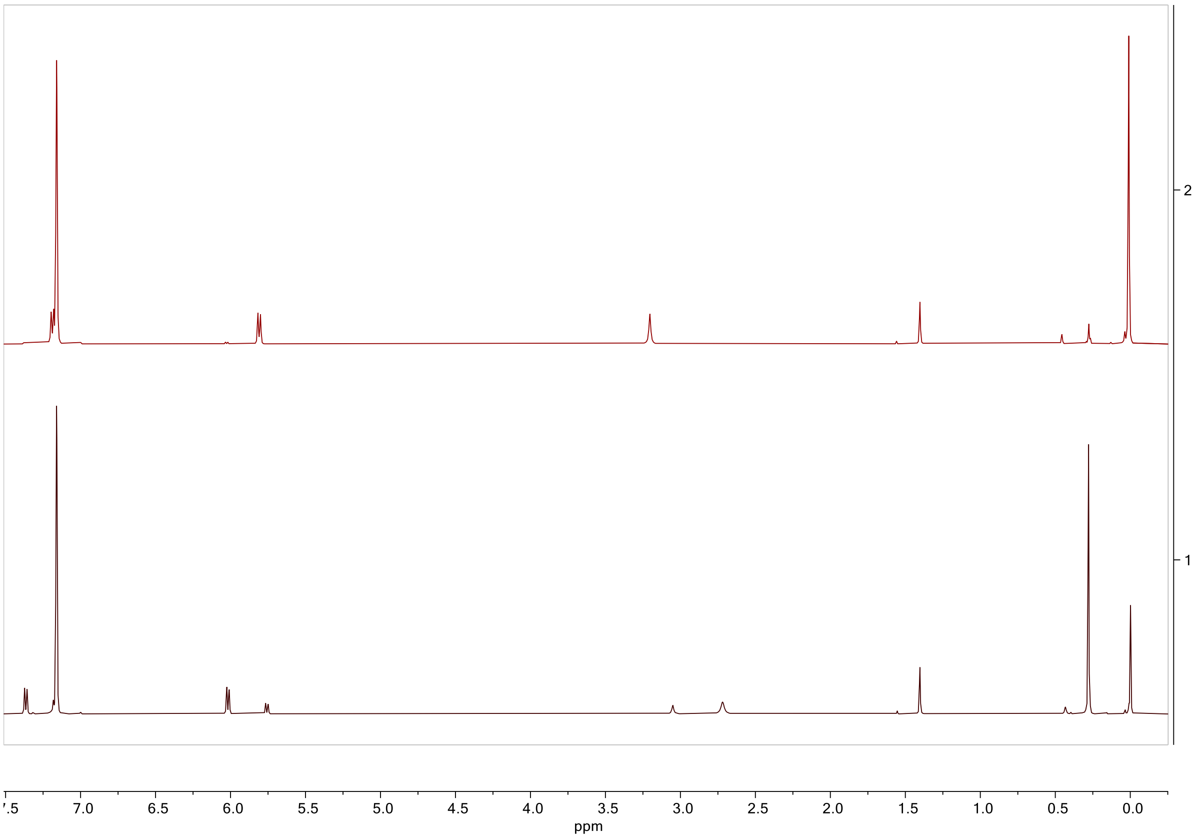

Figure S9.39.2. ^{1}H-NMR spectra (500 MHz, C_6D_6) corresponding to entry SI-53. Bottom spectrum was recorded at start of the reaction, and top spectrum was recorded after 12.5 hours of reaction time. The signal at 1.4 ppm corresponds to cyclohexane, which was used as an internal standard.

Section 9.40 – Entry SI-54

Reaction between **A** (R = *p*-NH_2) and **B** in C_6D_6 containing product **P**. The ratio of **A**:**B**:**P** is 1:1:1. Concentration of **A** and **B** = 0.0272 M. This experiment is identical to Section 9.37 - Entry 51, page S102 but with the concentrations of **A**, **B** and **P** needing to be precisely 0.0272 M to be comparable to a few other selected experiments.

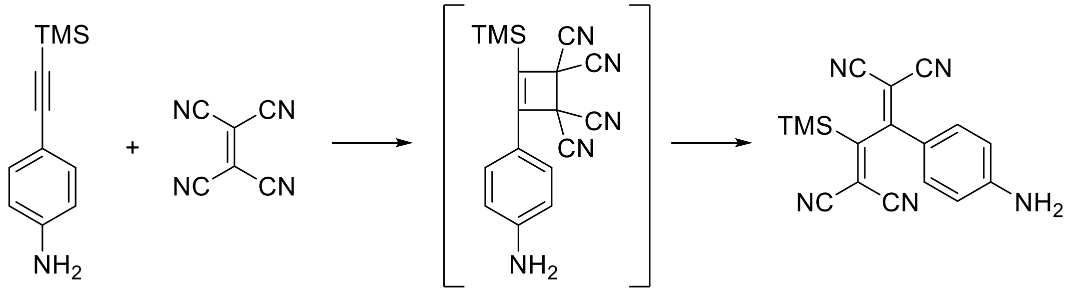

A **B** **C** **P**

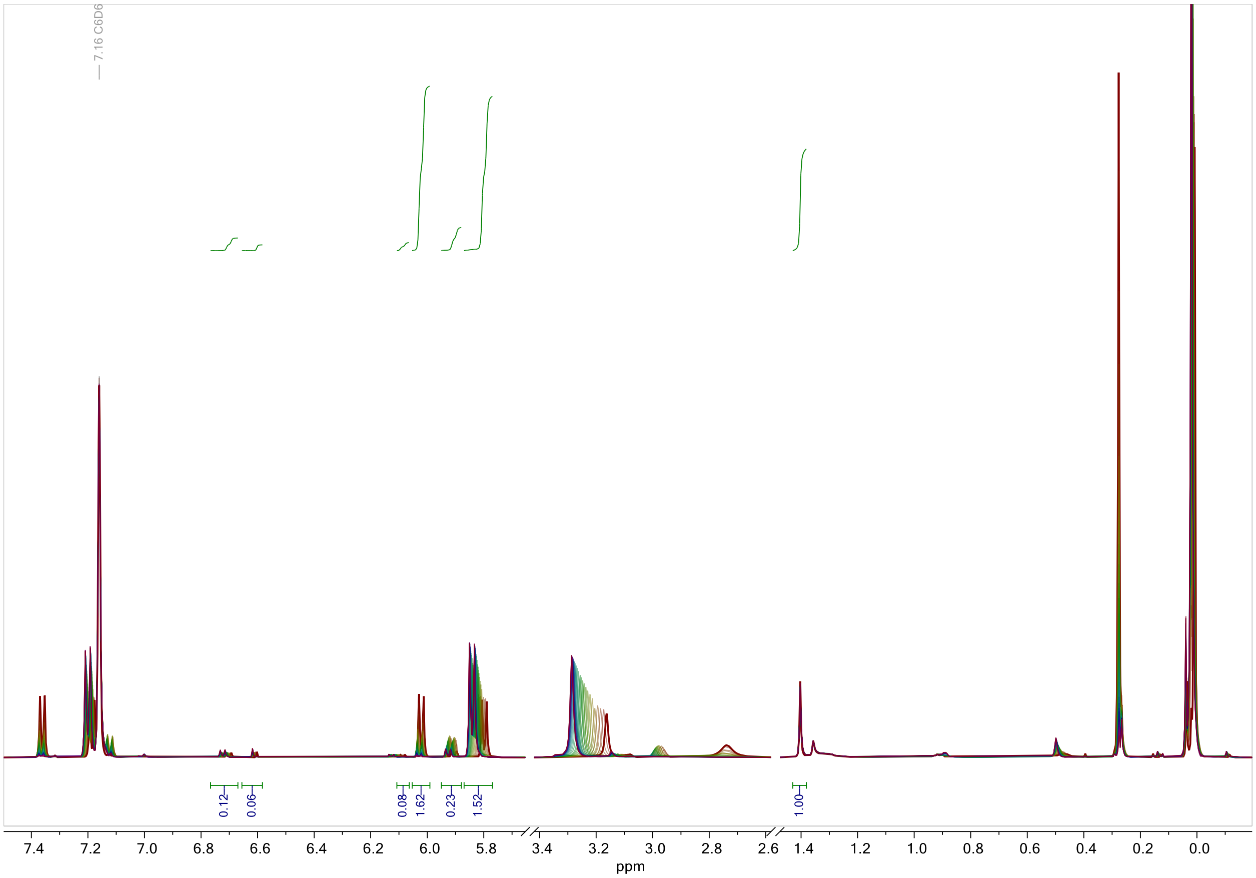

Figure S9.40.1. ^{1}H-NMR spectra (500 MHz, C_6D_6; selected regions shown) corresponding to entry SI-54. Superimposed spectra of all ^{1}H NMR spectra recorded during the experiment. The first spectrum recorded is colored red, and the last spectrum recorded is colored purple. The spectra were recorded precisely every 23 minutes for 10 hours and 24 minutes. Only the regions with signals are shown; intermediate regions were removed as indicated by the slashes. The signal at 1.4 ppm corresponds to cyclohexane, which was used as an internal standard.

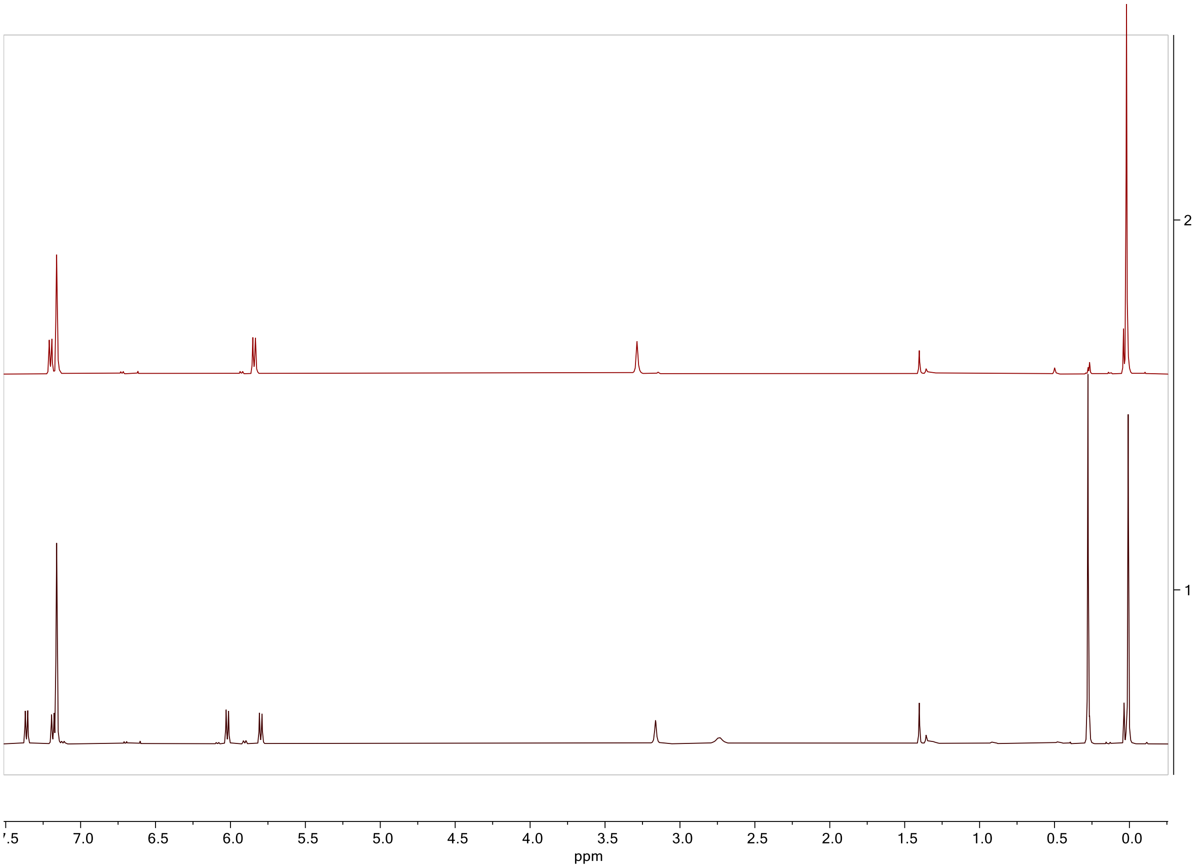

Figure S9.40.2. ^{1}H-NMR spectra (500 MHz, C_6D_6) corresponding to entry SI-54. Bottom spectrum was recorded at start of the reaction, and top spectrum was recorded after 10 hours and 24 minutes of reaction time. The signal at 1.4 ppm corresponds to cyclohexane, which was used as an internal standard.

Section 10 – Spectroscopic data for UV-Vis experiments

Section 10.1 – Entry SI-UV

A stock solution of TCNE (91.2 mg, 7.12 mmol) and aniline (65 µL, 7.12 mmol) in benzene (50 mL) was made (concentration c = 0.0142 M). Several diluted samples were prepared. The UV-Vis spectra were recorded for these samples and for another sample (TCNE: 13.2 mg, 0.103 mmol; aniline: 9.4 µL, 0.103 mmol; benzene: 10 mL; concentration c = 0.0103). The spectra can be seen in Figure S10.1.1. A charge-transfer absorption band was observed at a maximum of 584 nm with absorbance A. By plotting c/A against $1/A^{1/2}$, a linear fit was obtained with slope α = 0.01395 M and intersection of the y-axis at y_0 = 4.08372 x 10^{-4} M (Figure S10.1.2). The association constant $K_{as} = y_0 / \alpha^2 = 2\ M^{-1}$.

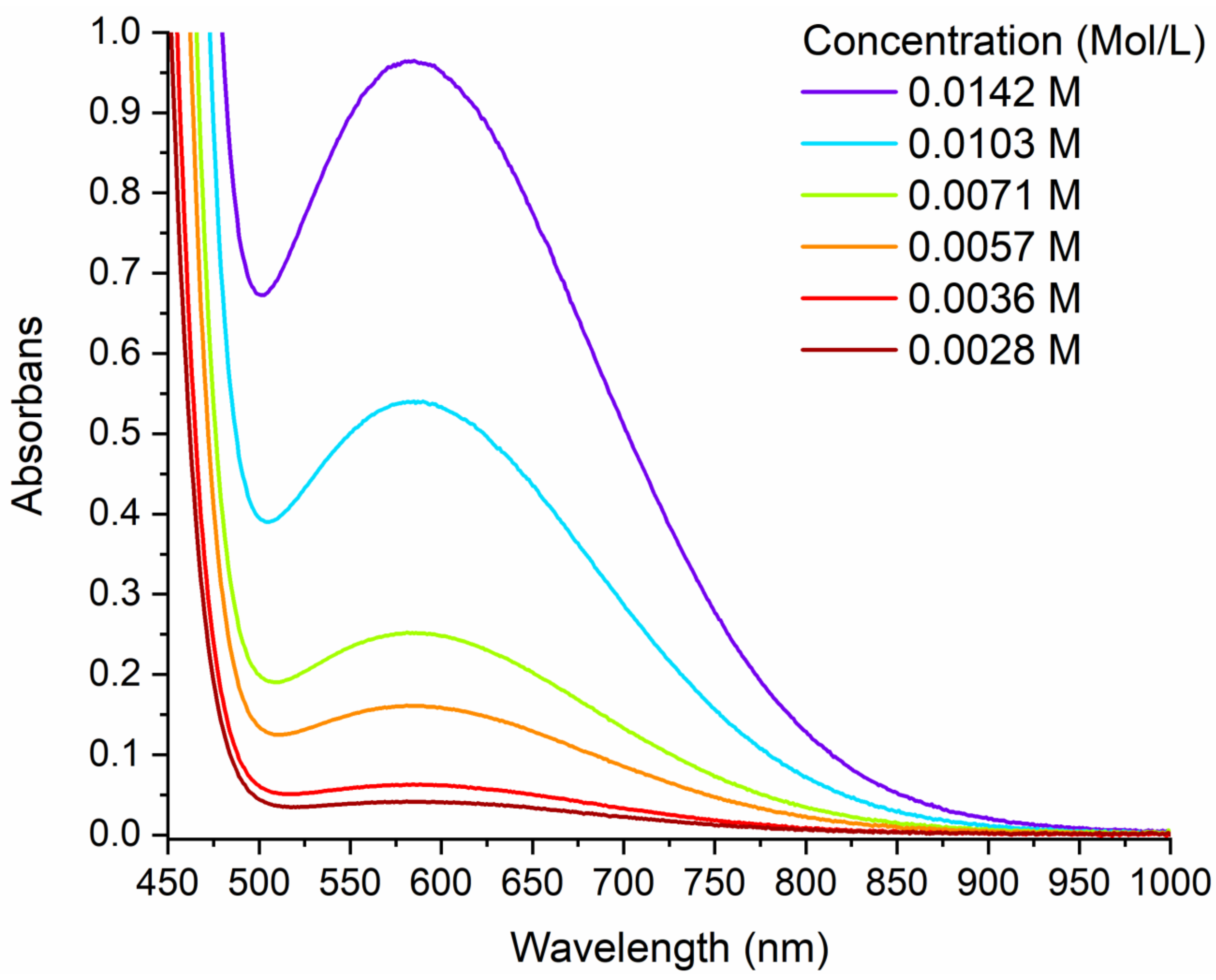

Figure S10.1.1. UV-Vis absorption spectra at different 1:1 concentrations of aniline and TCNE, corresponding to Entry SI-UV.

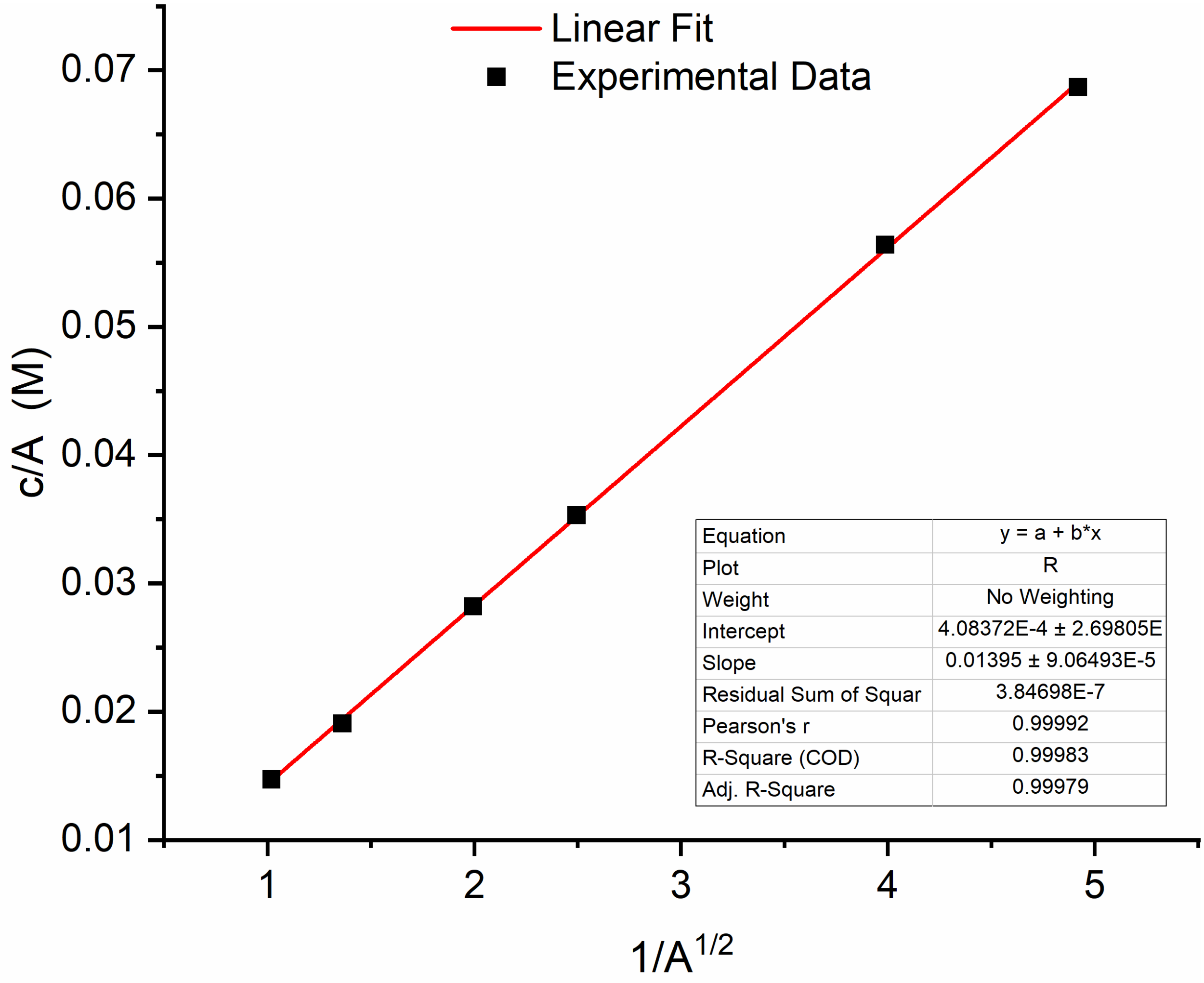

Figure S10.1.2. Plot of c/A against $1/A^{1/2}$ and the linear fit with corresponding data.

Section 11 – Spectroscopic data for the IR experiment

Section 11.1 – Entry SI-IR

IR spectra were measured *in situ* of a reaction mixture of TCNE (126.2 mg, 0.99 mmol) and 4-[(trimethylsilyl)ethynyl]aniline (32.8 mg, 0.173 mmol) by inserting the IR-probe directly into the reaction. A 3D surface graph of the experiment can be seen in Figure 11.1.1 and a top view of the same graph can be seen in Figure 11.1.2. The measured values were compared to calculated values (Figure 11.1.3).

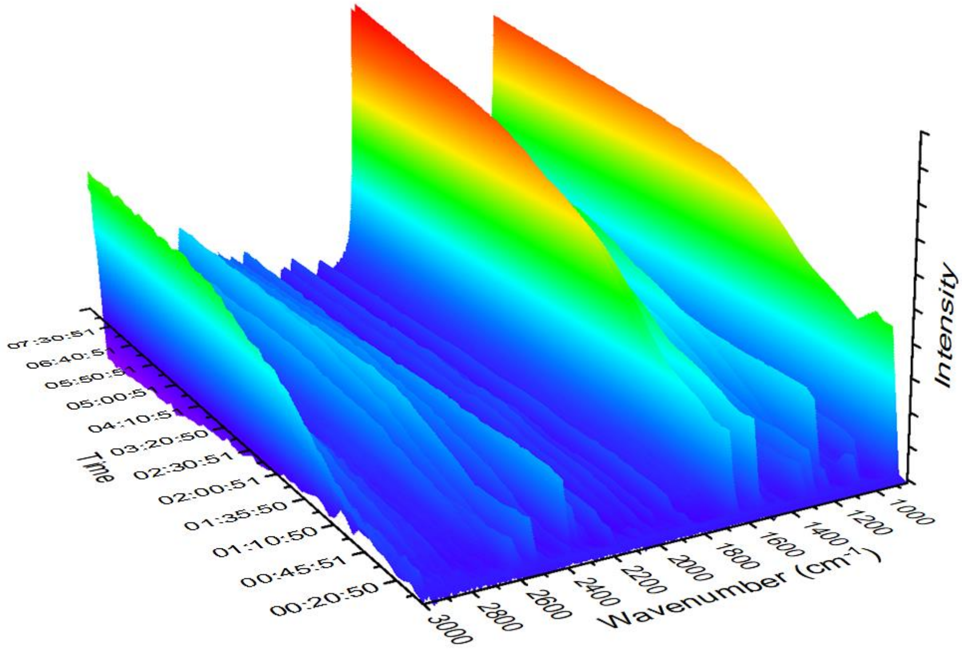

Figure 11.1.1. Measured IR spectra combined in a 3D surface graph in the range 850 cm^{-1} to 3000 cm^{-1}. The spectra were recorded every 5 minutes for 2 hours and 20 minutes and then every 10 min for 5 hours and 50 minutes for a total of 8 hours and 10 minutes reaction time.

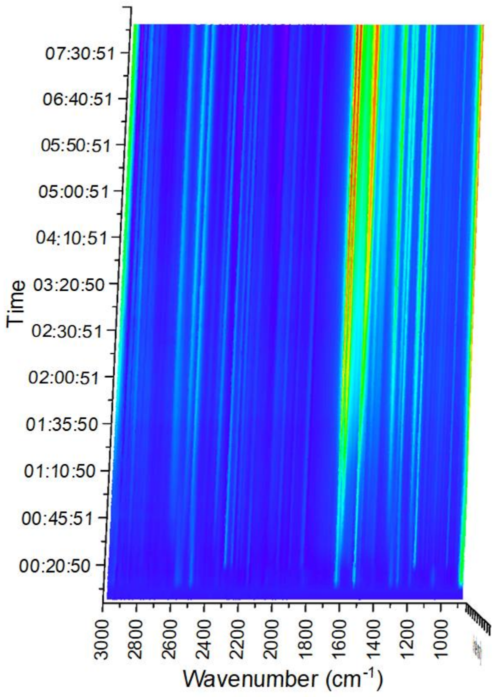

Figure 11.1.2. A bird view of the graph in Figure 11.1.1.

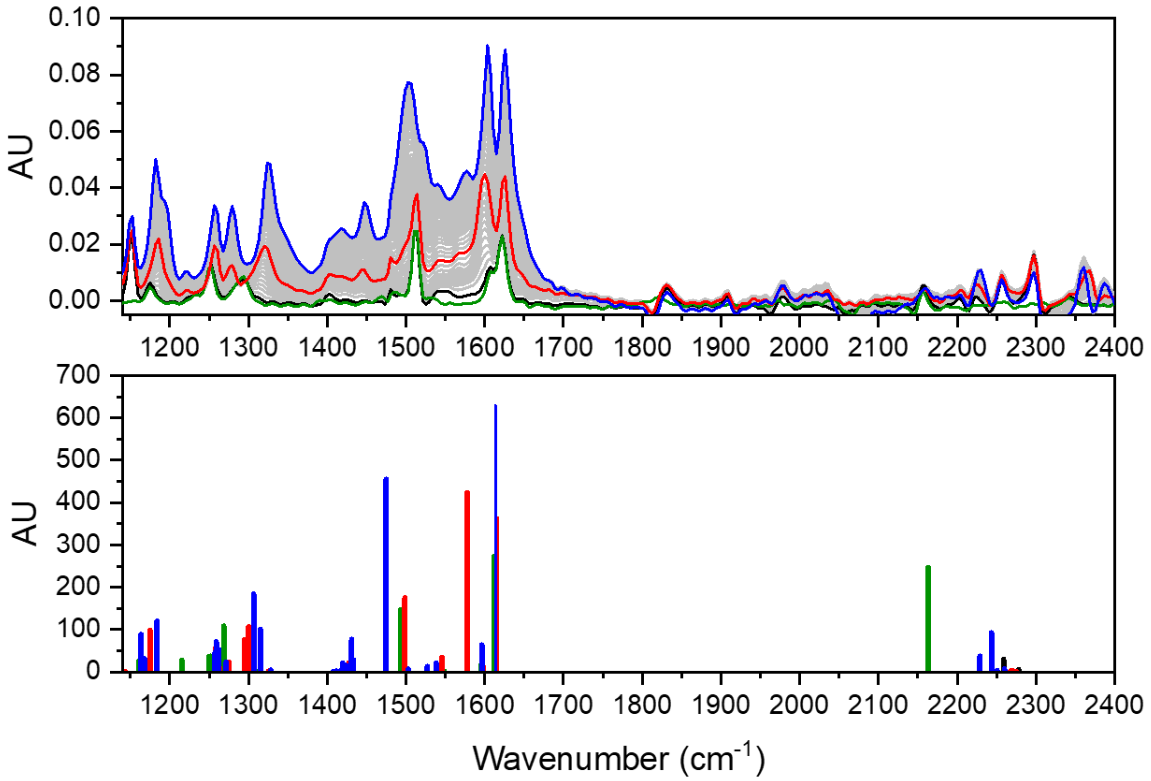

Figure 11.1.3. Top; a 2D overview of a selected area of the measured IR spectra with color coding matching the time at which color coded species had its highest concentration. Bottom; calculated IR values of each species (B3LYP/6-31+G(d,p); all wavenumbers were scaled by 0.9648).

Section 12 – Description of video recordings of the *CA-RE* reaction

Section 12.1 – Entry SI-VR1

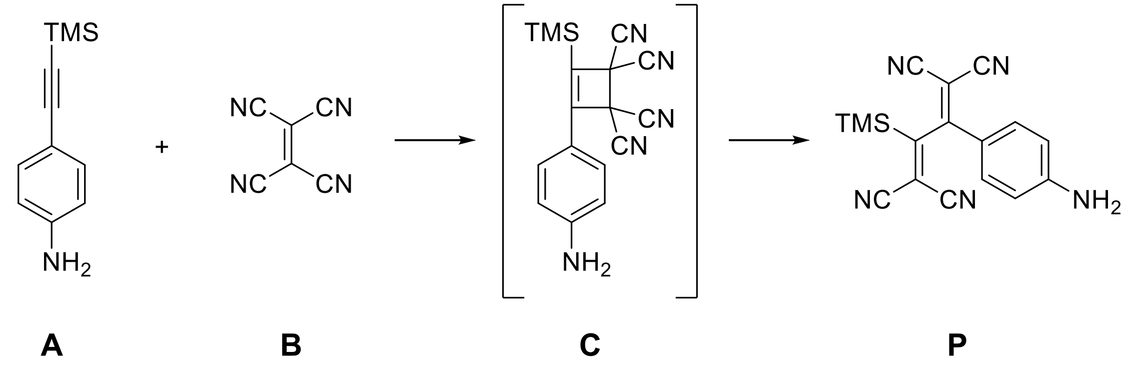

The recorded reaction is made from mixing two stock solutions made of TCNE (9.2 mg, 0.072 mmol) and **A** (14.9 mg, 0.079 mmol) so they are in a 1:1 ratio. The video starts with a solution of **A** where a solution of **B** is added after a few seconds. After mixing, the concentration of both species is 0.037 M. The color goes from yellow, which corresponds to TCNE complexing with benzene, to green when **A** is added; the mixture then turns gradually darker and yellow again until it ends in a brown/yellow color.

Section 12.2 – Entry SI-VR2

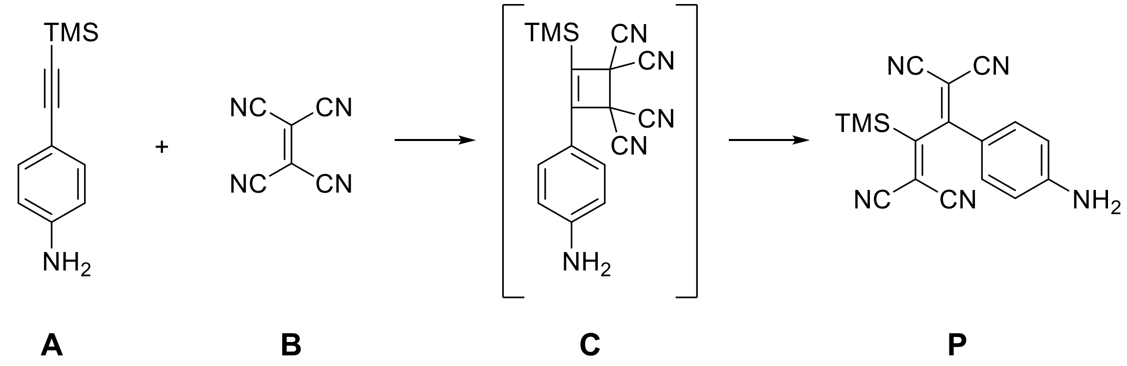

The recorded reaction is made from mixing two stock solutions made of TCNE (8.9 mg, 0.069 mmol) and **A** (14.3 mg, 0.076 mmol) so they are in a 1:1 ratio. The video starts with a yellow solution of **B** whereafter a solution of **A** is added after a few seconds. After mixing, the concentration of both species is 0.036 M. The color goes from transparent to blue, then within 2 minutes to green and then it switches to yellow over 5 minutes whereafter the solution darkens within an hour.

Section 13 – Summary of simulations of experimental data

Section 13.1 – Kinetic fits using model 1

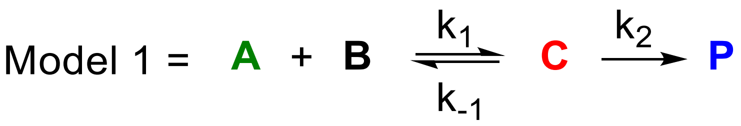

Figure S13.1.1. Schematic of model 1.

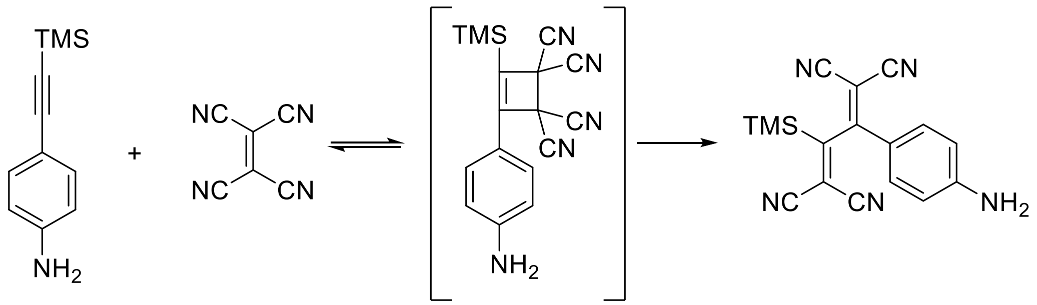

A B C P

Figure S13.1.2. Model 1 with structures shown.

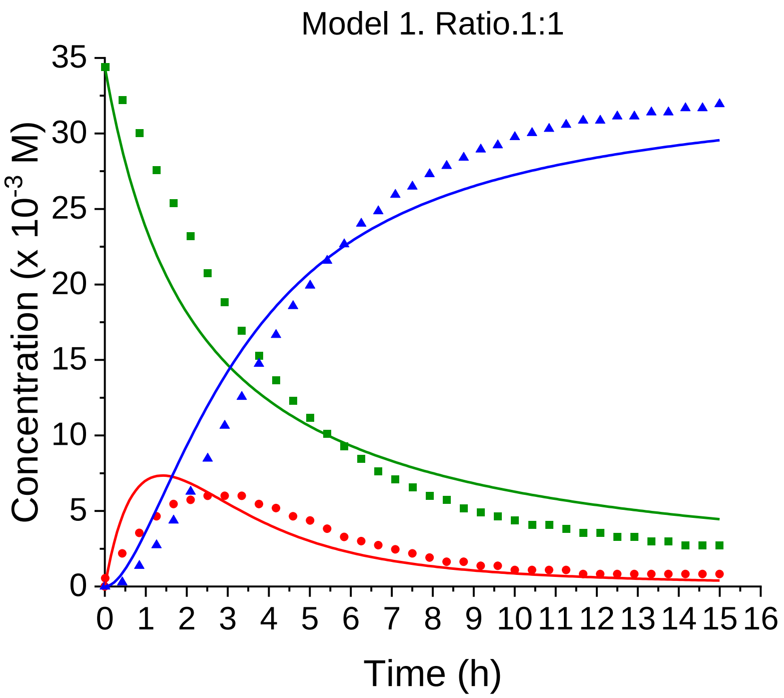

Figure S13.1.3. Concentrations of each component in the *CA-RE* reaction in a 1:1 ratio of **A** and **B** based on ^{1}H-NMR spectroscopic data as a function of time (colored symbols). Calculated emulations of the reaction based on model 1 are shown as solid curves (taking a total of 4 experiments into account).

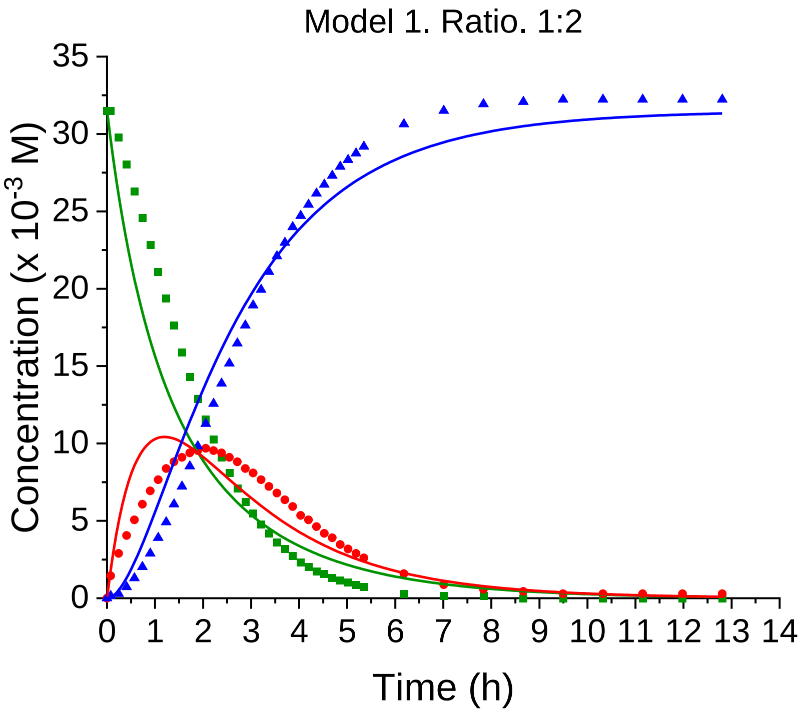

Figure S13.1.4. Concentrations of each component in the *CA-RE* reaction in a 1:2 ratio of **A** and **B** based on ^{1}H-NMR spectroscopic data as a function of time (colored dots). Calculated emulations of the reaction based on model 1 are shown as solid curves (taking a total of 4 experiments into account).

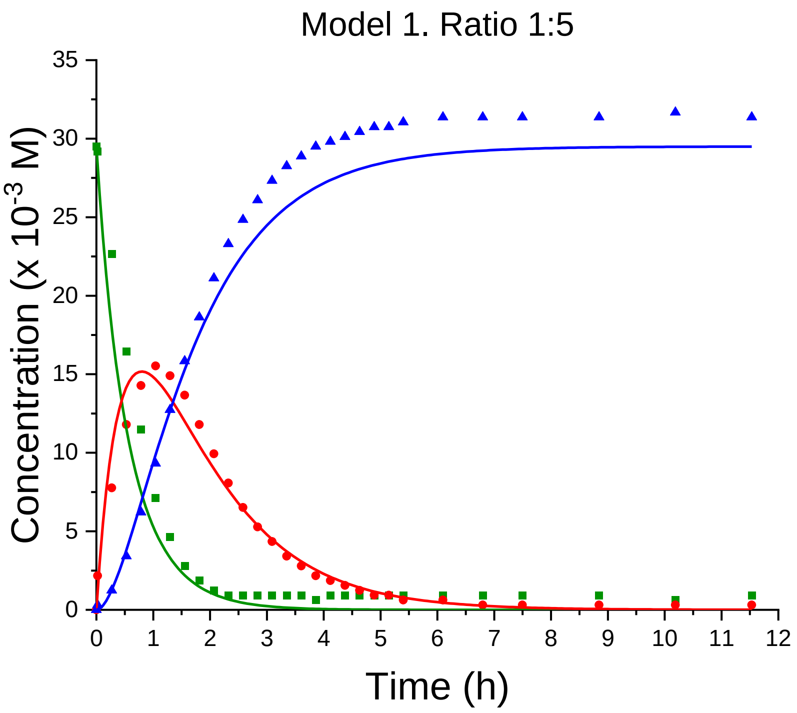

Figure S13.1.5. Concentrations of each component in the *CA-RE* reaction in a 1:5 ratio of **A** and **B** based on ^{1}H-NMR spectroscopic data as a function of time (colored symbols). Calculated emulations of the reaction based on model 1 are shown as solid curves (taking a total of 4 experiments into account).

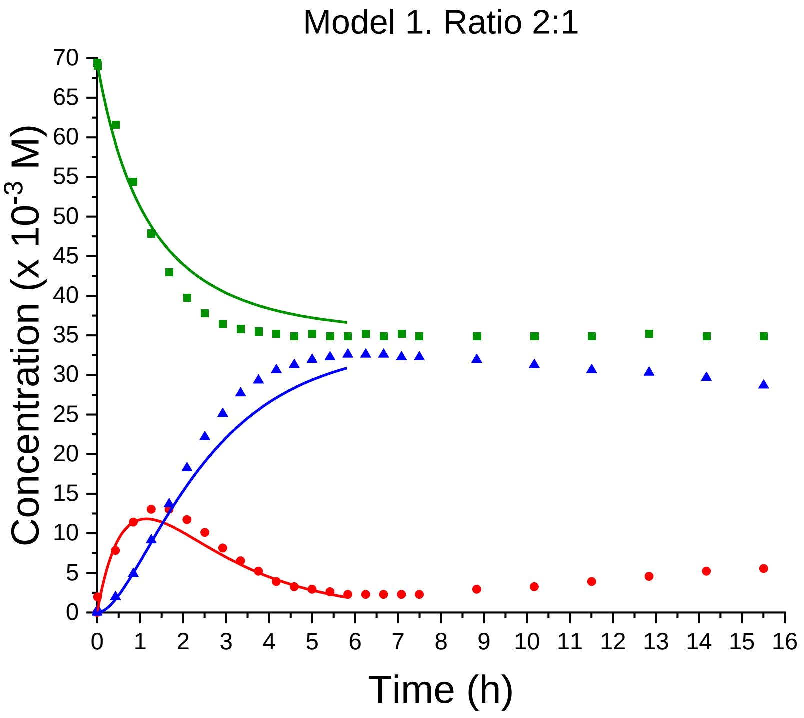

Figure S13.1.6. Concentrations of each component in the *CA-RE* reaction in a 2:1 ratio of **A** and **B** based on ^{1}H-NMR spectroscopic data as a function of time (colored symbols). Calculated emulations of the reaction based on model 1 are shown as solid curves (taking a total of 4 experiments into account).

Section 13.2 – Kinetic fits using model 2

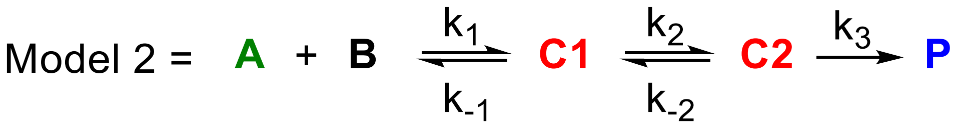

Figure S13.2.1. Schematic of model 2.

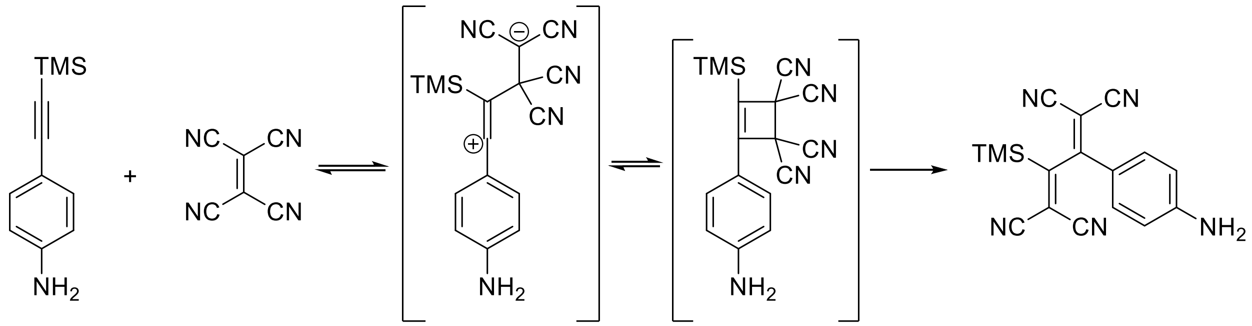

A B C1 C2 P

Figure S13.2.2. Model 2 with structures shown.

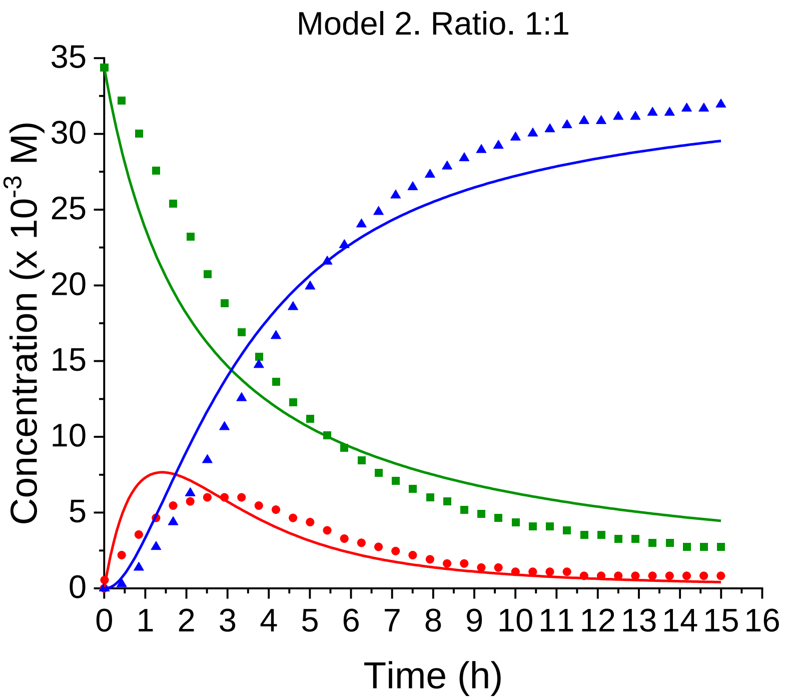

Figure S13.2.3. Concentrations of each component in the *CA-RE* reaction in a 1:1 ratio of **A** and **B** based on ^{1}H-NMR spectroscopic data as a function of time (colored symbols). Calculated emulations of the reaction based on model 2 are shown as solid curves (taking a total of 4 experiments into account).

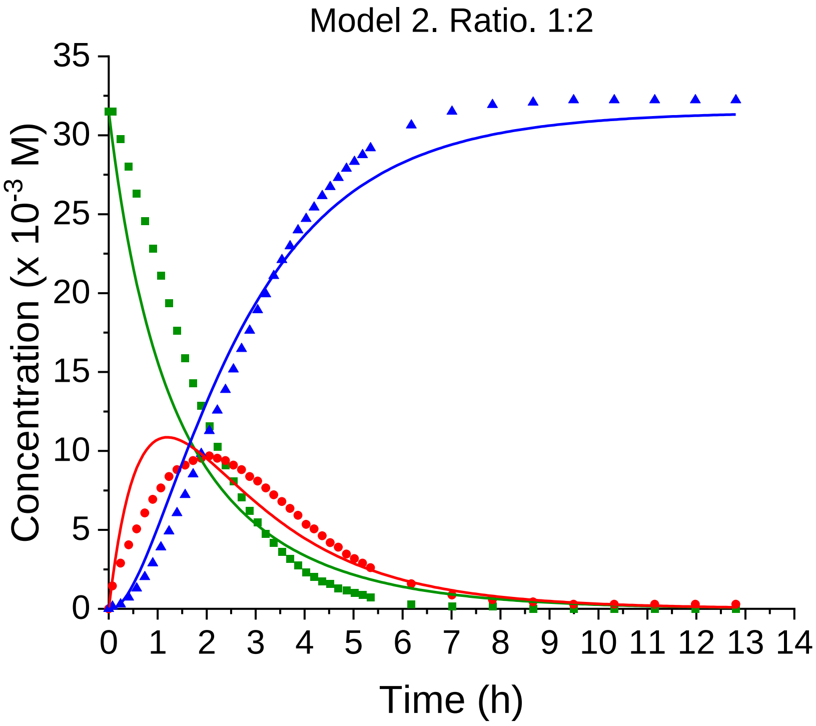

Figure S13.2.4. Concentrations of each component in the *CA-RE* reaction in a 1:2 ratio of **A** and **B** based on ^{1}H-NMR spectroscopic data as a function of time (colored symbols). Calculated emulations of the reaction based on model 2 are shown as solid curves (taking a total of 4 experiments into account).

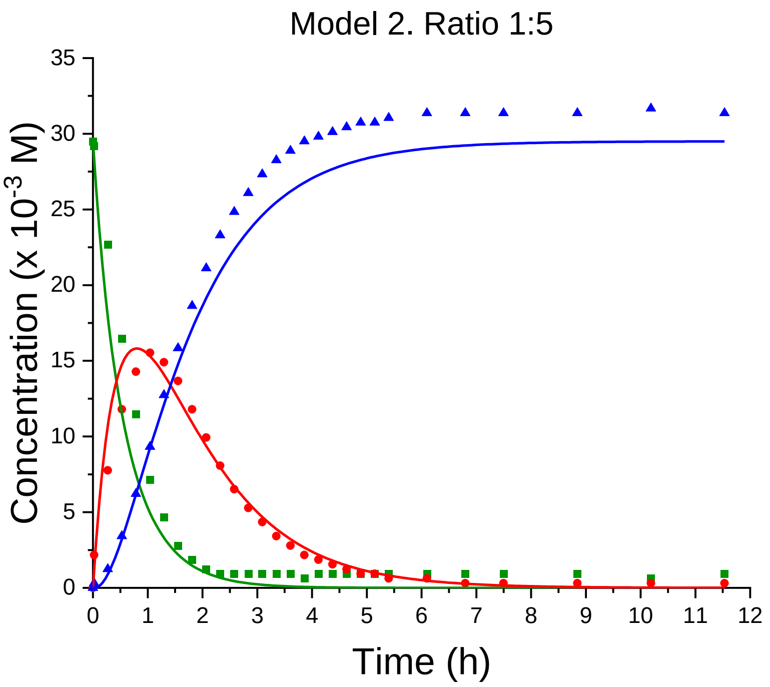

Figure S13.2.5. Concentrations of each component in the *CA-RE* reaction in a 1:5 ratio of **A** and **B** based on ^{1}H-NMR spectroscopic data as a function of time (colored symbols). Calculated emulations of the reaction based on model 2 are shown as solid curves (taking a total of 4 experiments into account).

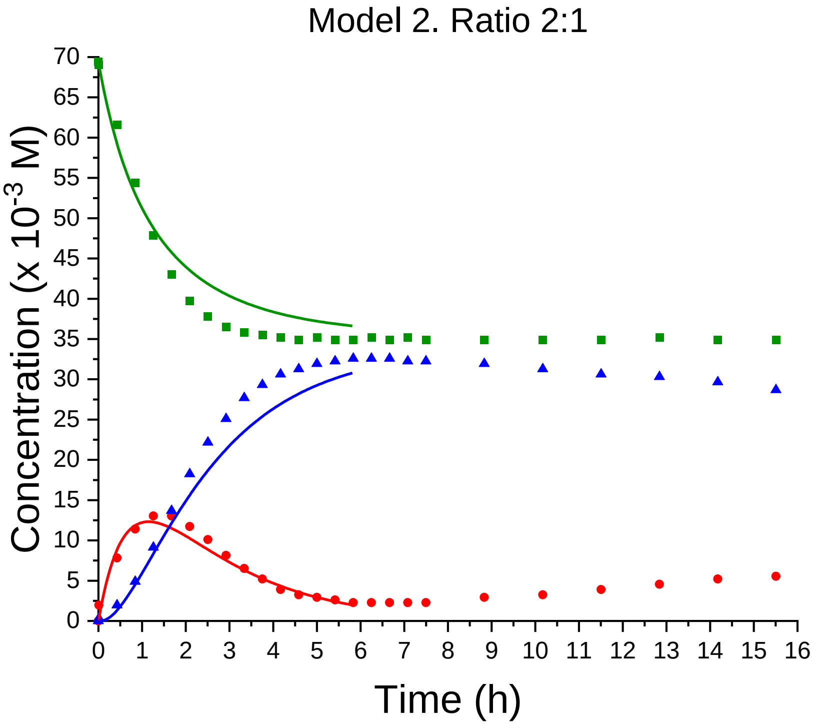

Figure S13.2.5. Concentrations of each component in the *CA-RE* reaction in a 2:1 ratio of **A** and **B** based on ^{1}H-NMR spectroscopic data as a function of time (colored symbols). Calculated emulations of the reaction based on model 2 are shown as solid curves (taking a total of 4 experiments into account).

Section 13.3 – Kinetic fits using model 3

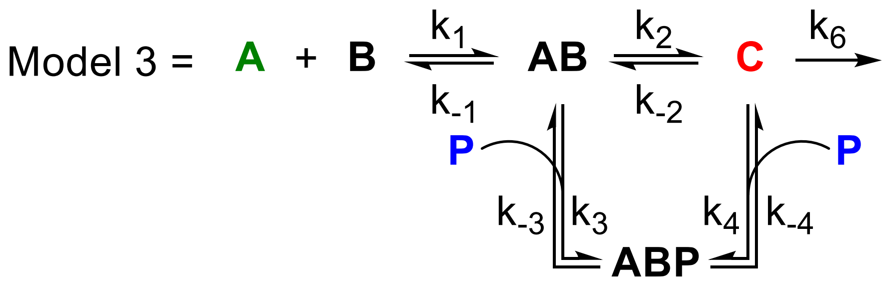

Figure S13.3.1. Schematic of model 3.

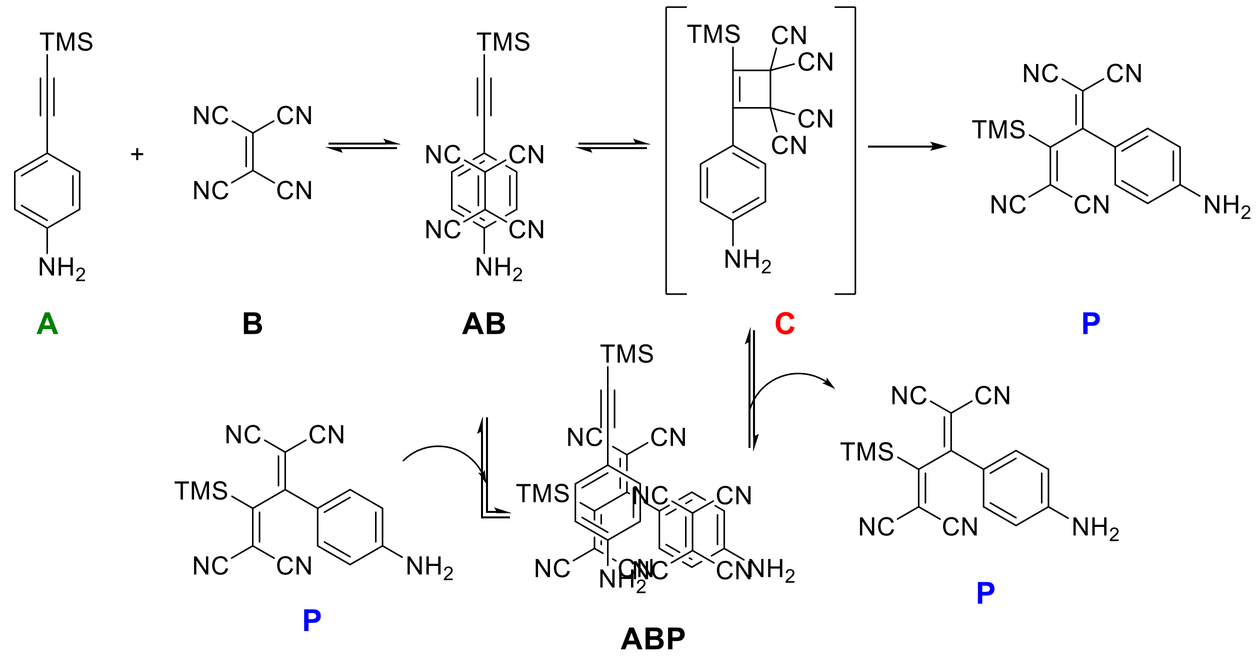

Figure S13.3.2. Model 3 with structures shown.

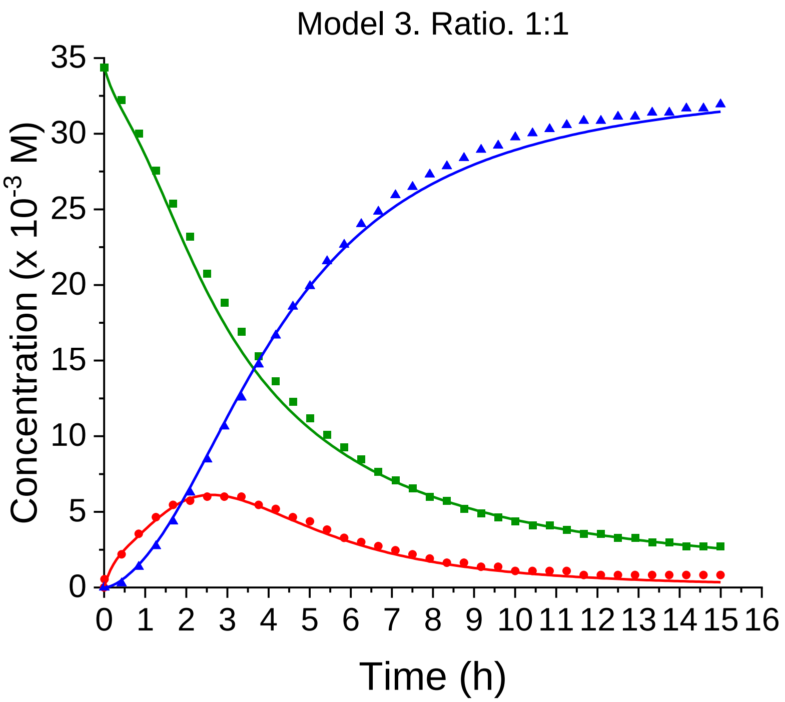

Figure S13.3.3. Concentrations of each component in the *CA-RE* reaction in a 1:1 ratio of **A** and **B** based on ^{1}H-NMR spectroscopic data as a function of time (colored symbols). Calculated emulations of the reaction based on model 3 are shown as solid curves (taking a total of 6 experiments into account).

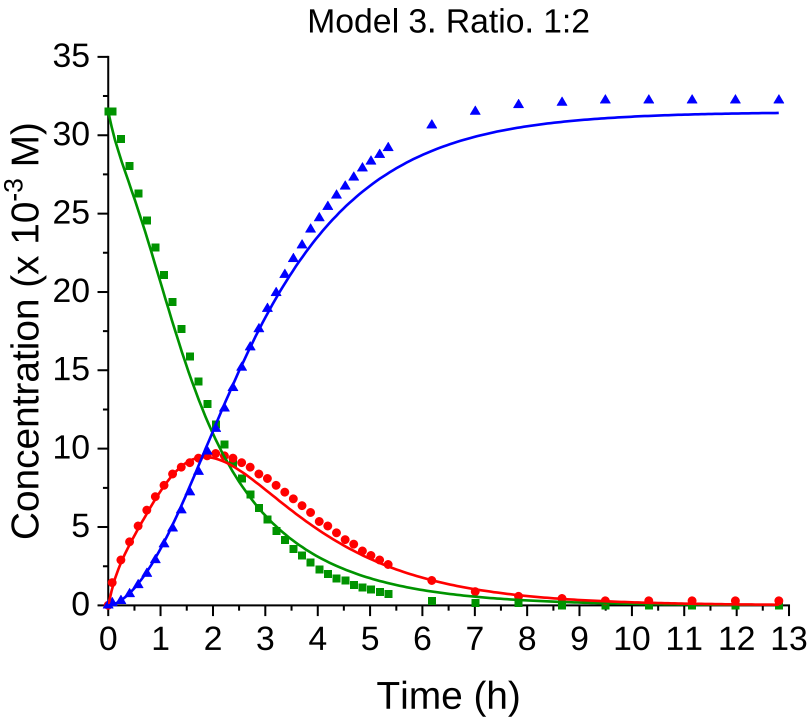

Figure S13.3.4. Concentrations of each component in the *CA-RE* reaction in a 1:2 ratio of **A** and **B** based on ^{1}H-NMR spectroscopic data as a function of time (colored symbols). Calculated emulations of the reaction based on model 3 are shown as solid curves (taking a total of 6 experiments into account).

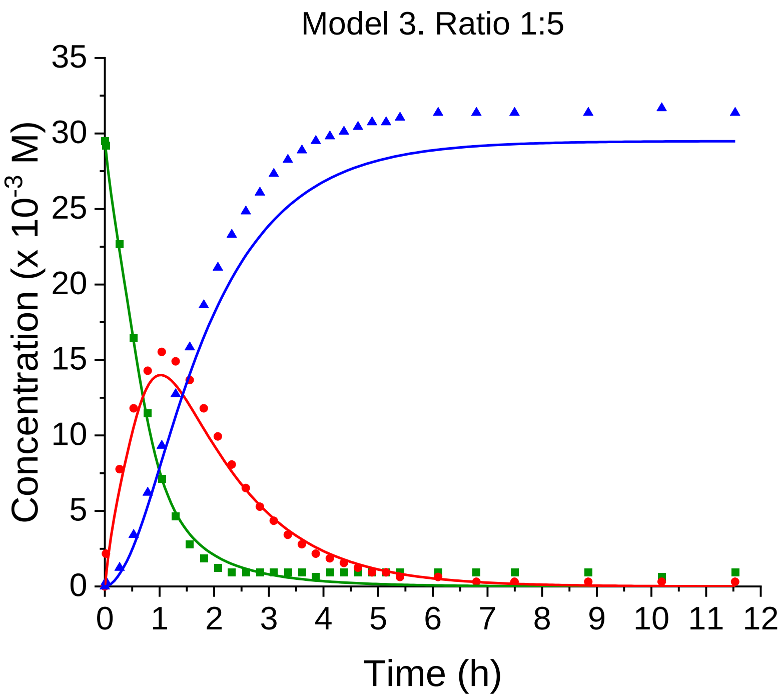

Figure S13.3.5. Concentrations of each component in the *CA-RE* reaction in a 1:5 ratio of **A** and **B** based on ^{1}H-NMR spectroscopic data as a function of time (colored symbols). Calculated emulations of the reaction based on model 3 are shown as solid curves (taking a total of 6 experiments into account).

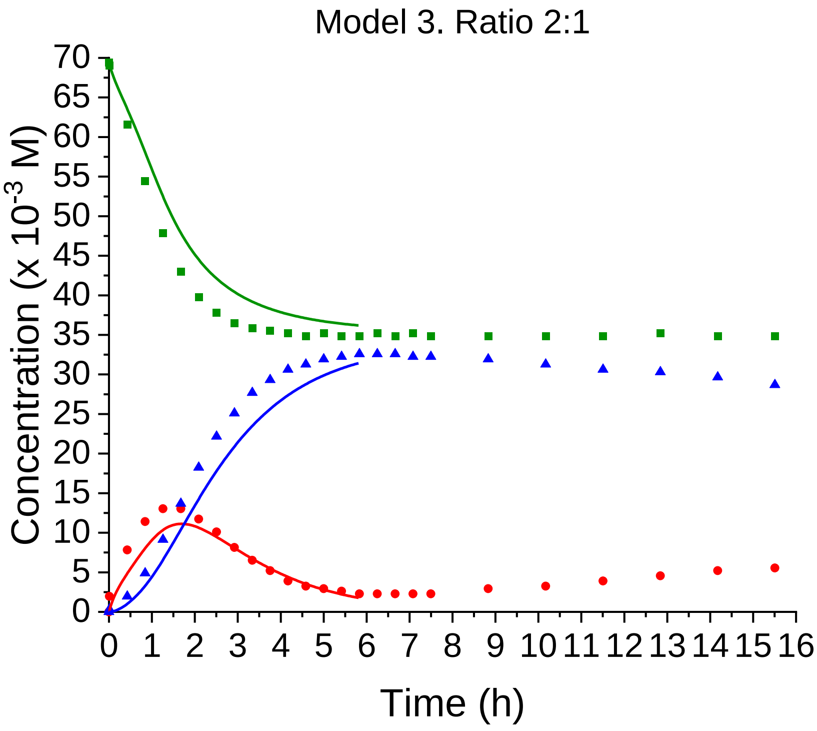

Figure S13.3.6. Concentrations of each component in the *CA-RE* reaction in a 2:1 ratio of **A** and **B** based on ^{1}H-NMR spectroscopic data as a function of time (colored symbols). Calculated emulations of the reaction based on model 3 are shown as solid curves (taking a total of 6 experiments into account).

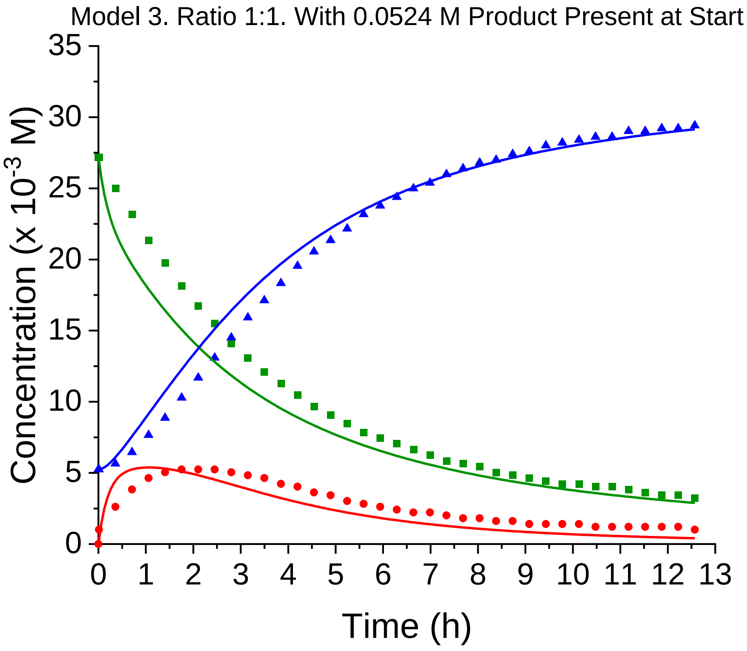

Figure S13.3.7. Concentrations of each component in the *CA-RE* reaction in a 1:1 ratio of **A** and **B** with 0.0524 M **P** present at start based on ^{1}H-NMR spectroscopic data as a function of time (colored symbols). Calculated emulations of the reaction based on model 3 are shown as solid curves (taking a total of 6 experiments into account).

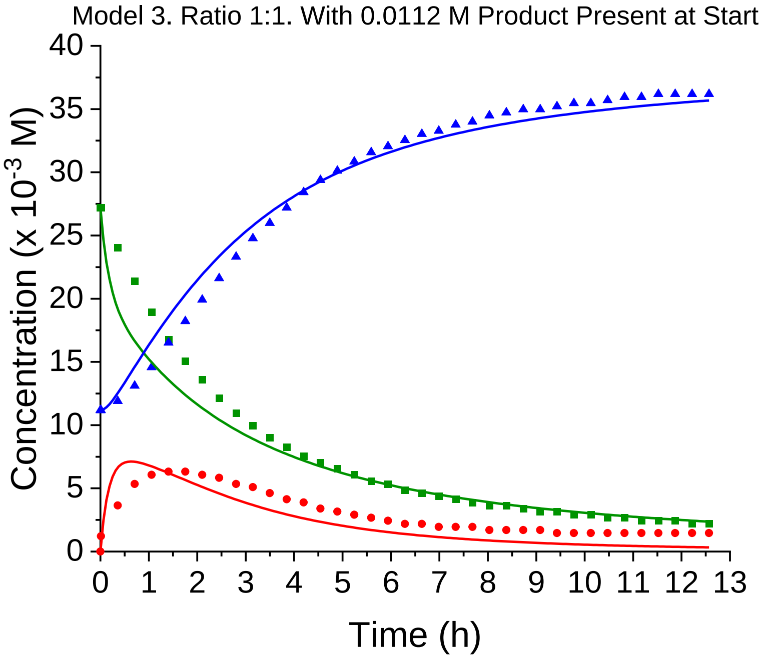

Figure S13.3.8. Concentrations of each component in the *CA-RE* reaction in a 1:1 ratio of **A** and **B** with 0.0112 M **P** present at start based on ^{1}H-NMR spectroscopic data as a function of time (colored symbols). Calculated emulations of the reaction based on model 3 are shown as solid curves (taking a total of 6 experiments into account).

Section 13.4 – Kinetic fits using model 4

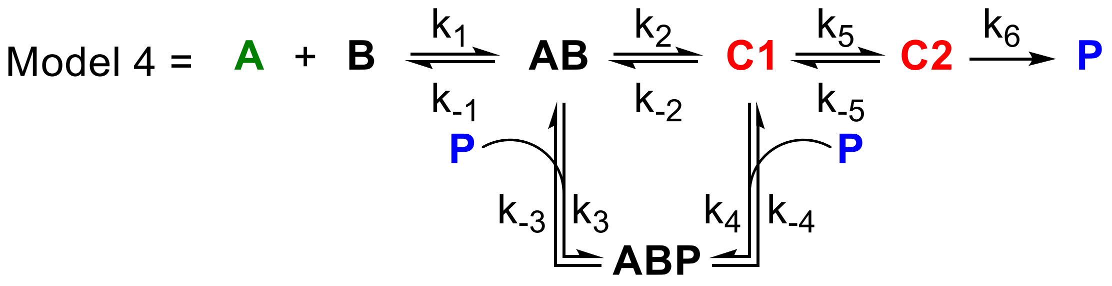

Figure S13.4.1. Schematic of model 4.

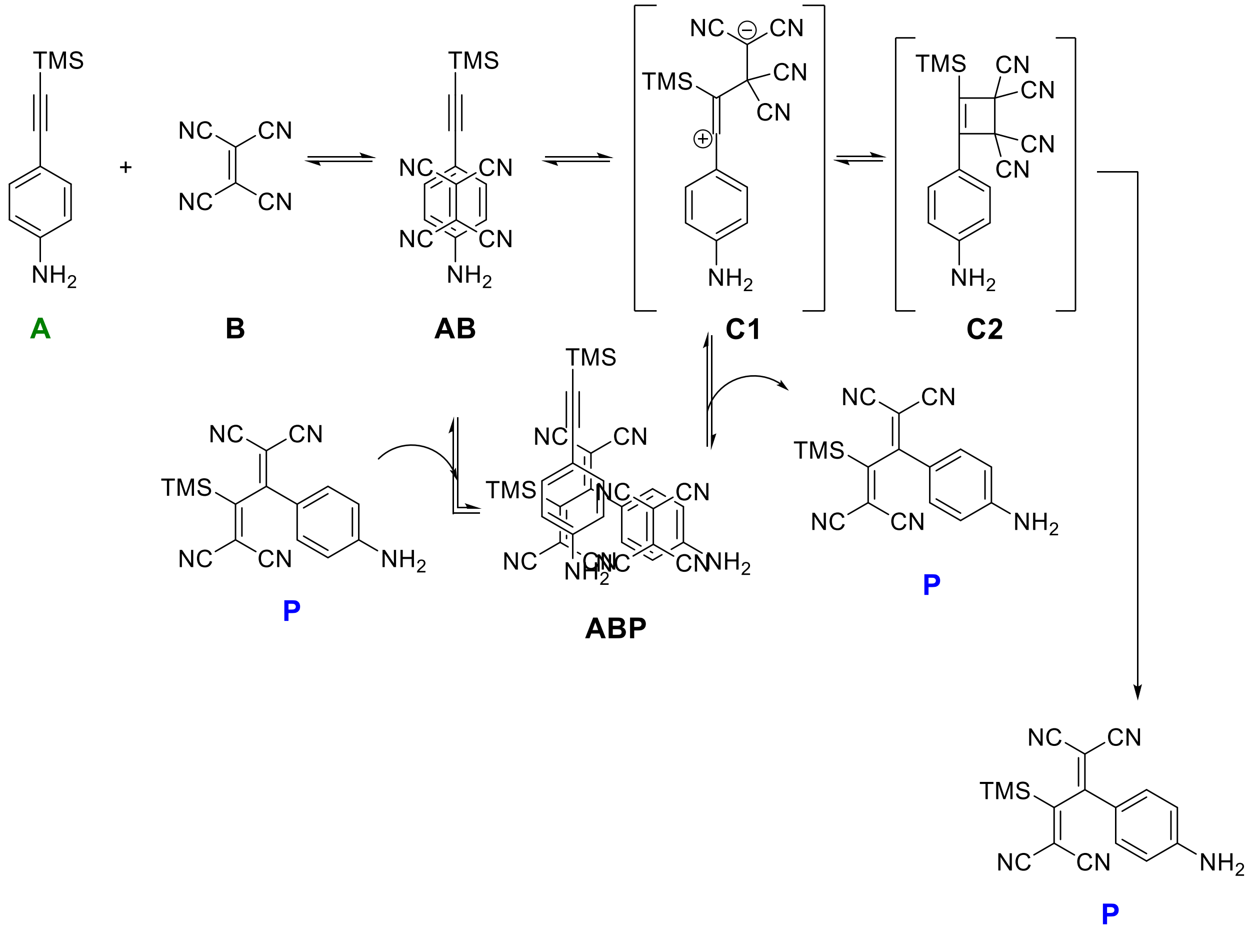

Figure S13.4.2. Model 4 with structures shown.

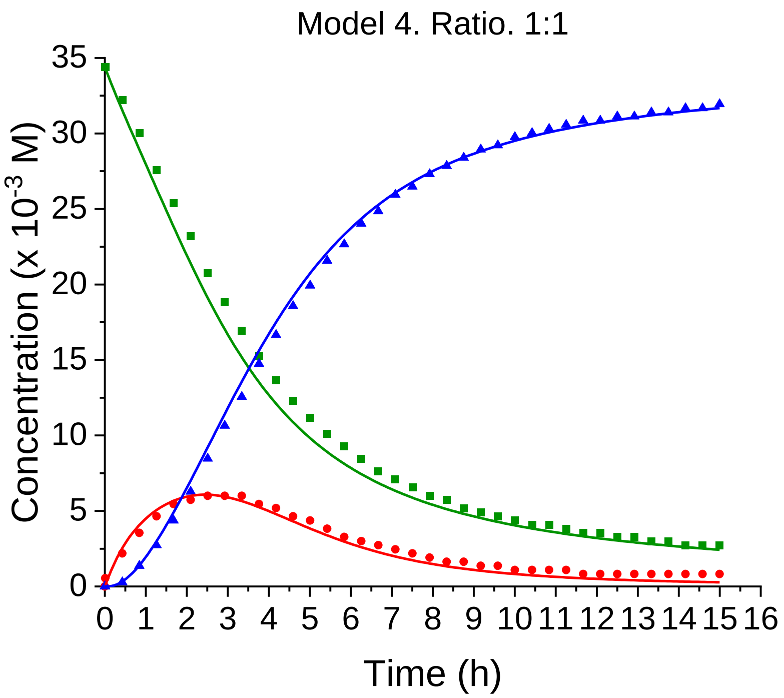

Figure S13.4.3. Concentrations of each component in the *CA-RE* reaction in a 1:1 ratio of **A** and **B** based on ^{1}H-NMR spectroscopic data as a function of time (colored symbols). Calculated emulations of the reaction based on model 4 are shown as solid curves (taking a total of 6 experiments into account).

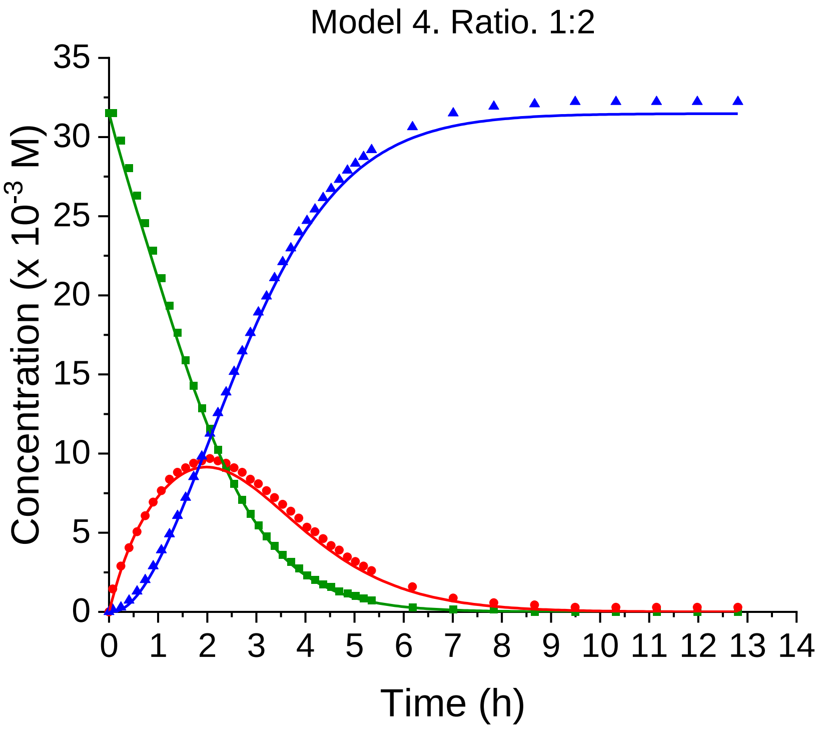

Figure S13.4.4. Concentrations of each component in the *CA-RE* reaction in a 1:2 ratio of **A** and **B** based on ^{1}H-NMR spectroscopic data as a function of time (colored symbols). Calculated emulations of the reaction based on model 4 are shown as solid curves (taking a total of 6 experiments into account).

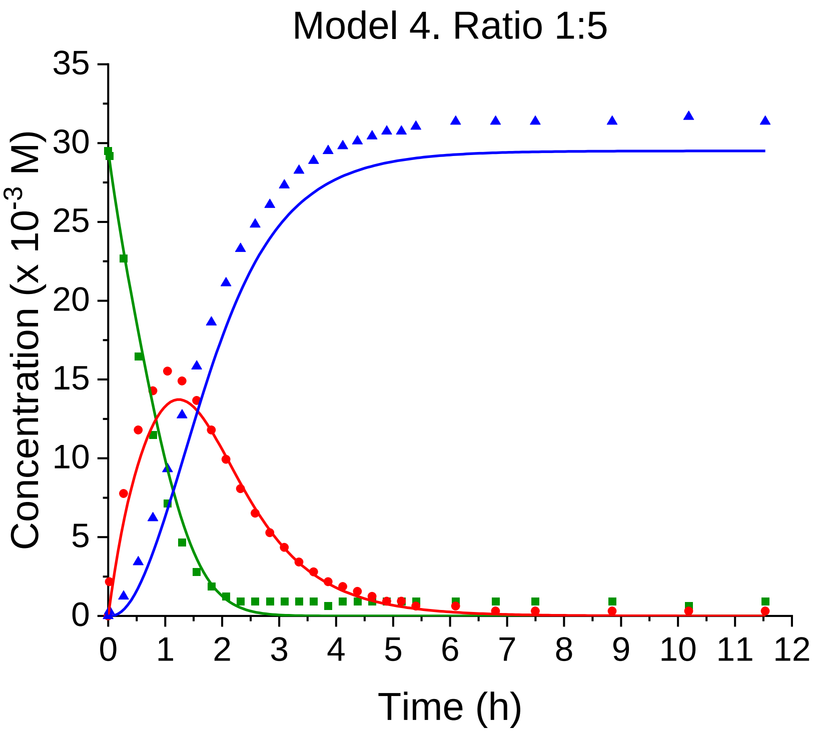

Figure S13.4.5. Concentrations of each component in the *CA-RE* reaction in a 1:5 ratio of **A** and **B** based on ^{1}H-NMR spectroscopic data as a function of time (colored symbols). Calculated emulations of the reaction based on model 4 are shown as solid curves (taking a total of 6 experiments into account).

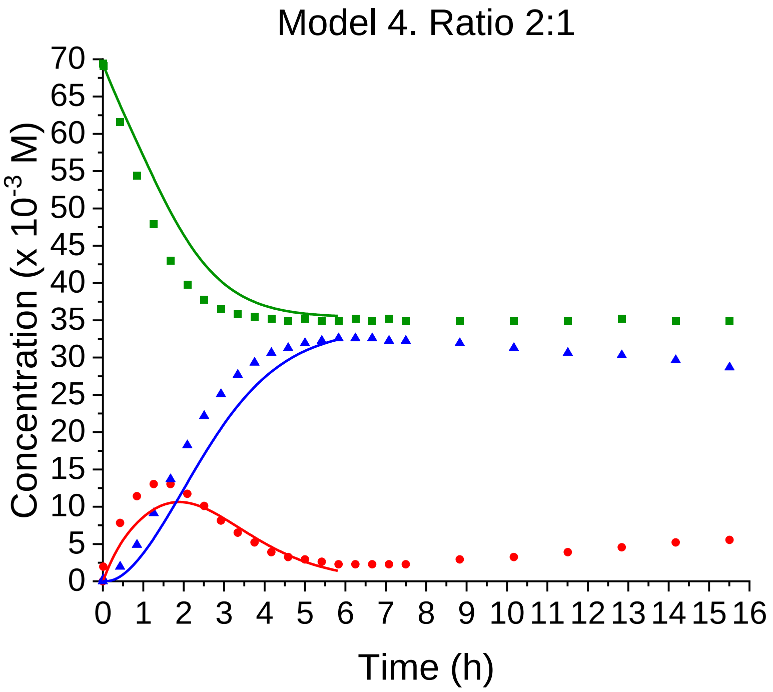

Figure S13.4.6. Concentrations of each component in the *CA-RE* reaction in a 1:5 ratio of **A** and **B** based on ^{1}H-NMR spectroscopic data as a function of time (colored symbols). Calculated emulations of the reaction based on model 4 are shown as solid curves (taking a total of 6 experiments into account).

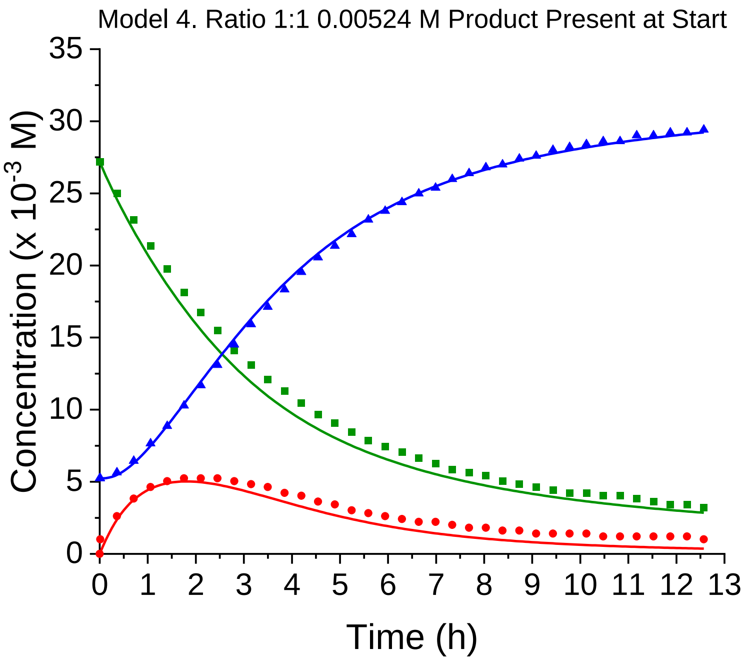

Figure S13.3.8. Concentrations of each component in the *CA-RE* reaction in a 1:1 ratio of **A** and **B** with 0.00524 M **P** present at start based on ^{1}H-NMR spectroscopic data as a function of time (colored symbols). Calculated emulations of the reaction based on model 4 are shown as solid curves (taking a total of 6 experiments into account).

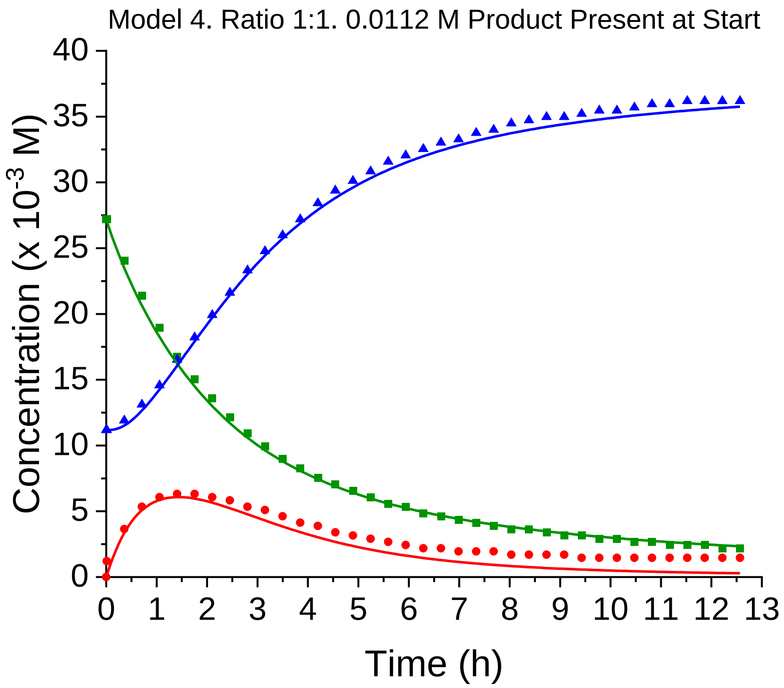

Figure S13.3.8. Concentrations of each component in the *CA-RE* reaction in a 1:1 ratio of **A** and **B** with 0.0112 M **P** present at start based on ^{1}H-NMR spectroscopic data as a function of time (colored symbols). Calculated emulations of the reaction based on model 4 are shown as solid curves (taking a total of 6 experiments into account).

Section 14 – Example of Kinetic Studies on an unnumbered model

Section 14.1 – Kinetic fits using an unnumbered model

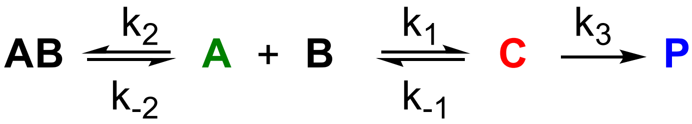

Figure S14.1.1. Schematic of an unnumbered model.

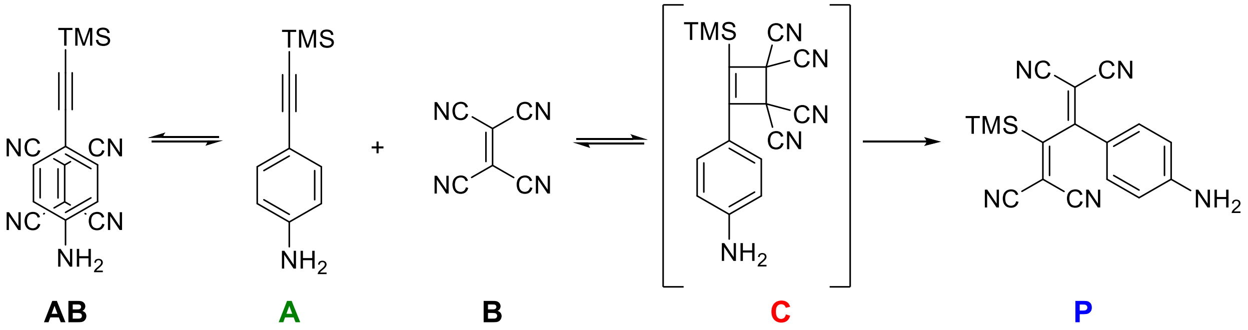

Figure S14.1.2. Unnumbered model with structures shown.

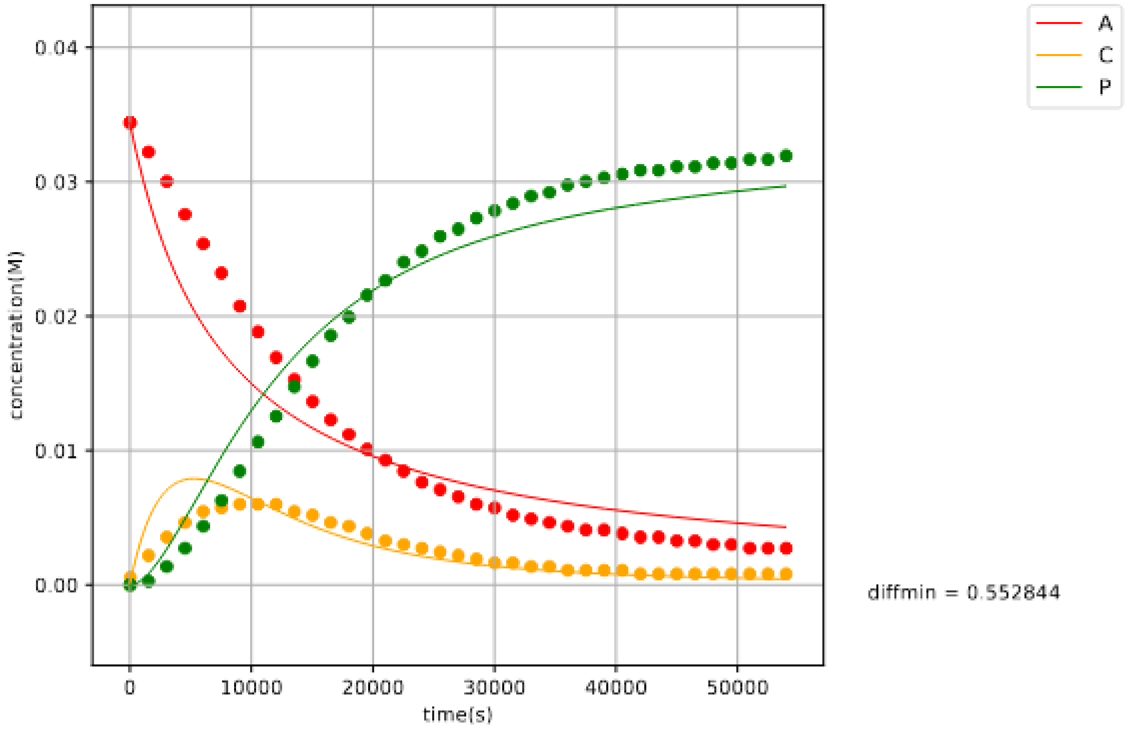


```
# A: alkyne, B: TCNE, C: intermediate, P: product

@sc =0.4;
@gr = 0.25;
@k1 = 0.00377084761;
@km1 = 0.00000000062;
@k2 = 0.000032928;
@km2 = 0.01487556;
@k3 = 0.00019921215;

stime = 0; dtime = 1000; etime = 56000;

1:    A + B  <=>   C          ; k> = k1;  k< = km1;
2:    A + B  <=>   AB         ; k> = k2;  k< = km2;
3:        C   ->   P          ; k> = k3;
```

Figure S14.1.3. Experimental data in dot of the *CA-RE* reaction in a 1:1 ratio (**A**/**B**) with a simulated fit as a line based of an unnumbered model described at the bottom. The calculated rate constants are seen in the middle. Figures taken directly from the exported data the custom-made program computes. The program has taken data from 4 experiments at the same time when calculating the rate constants.

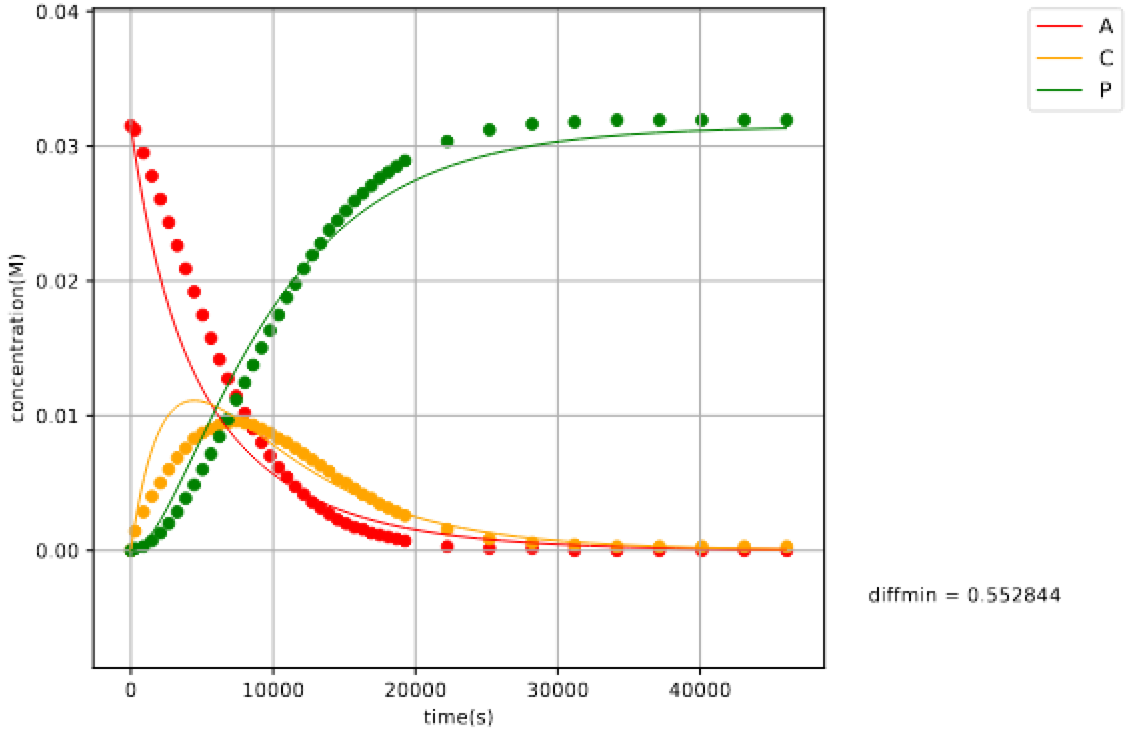


```
# A: alkyne, B: TCNE, C: intermediate, P: product

@sc =0.4;
@gr = 0.25;
@k1 = 0.00377084761;
@km1 = 0.00000000062;
@k2 = 0.000032928;
@km2 = 0.01487556;
@k3 = 0.00019921215;

stime = 0; dtime = 1000; etime = 56000;

1:    A + B  <=>   C          ; k> = k1;  k< = km1;
2:    A + B  <=>   AB         ; k> = k2;  k< = km2;
3:        C   ->   P          ; k> = k3;
```

Figure S14.1.4. Experimental data in dot of the *CA-RE* reaction in a 1:2 ratio (**A**/**B**) with a simulated fit as a line based of an unnumbered model described at the bottom. The calculated rate constants are seen in the middle. Figures taken directly from the exported data the custom-made program computes. The program has taken data from 4 experiments at the same time when calculating the rate constants.

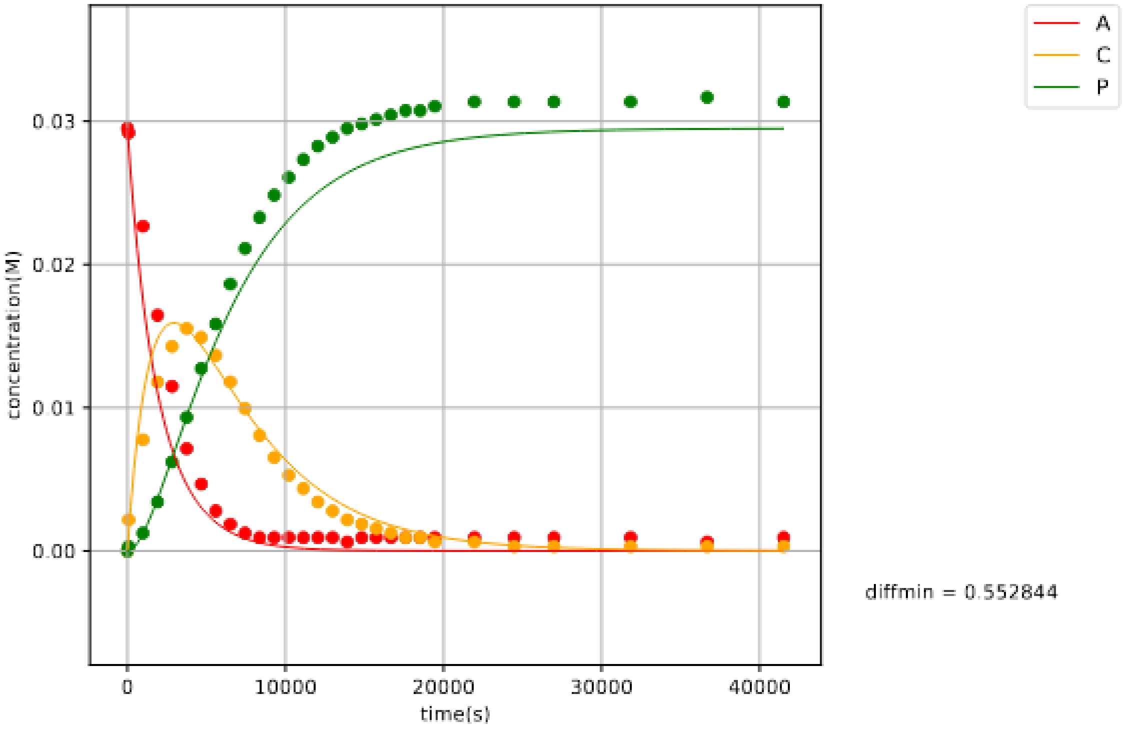


```
# A: alkyne, B: TCNE, C: intermediate, P: product

@sc =0.4;
@gr = 0.25;
@k1 = 0.00377084761;
@km1 = 0.00000000062;
@k2 = 0.000032928;
@km2 = 0.01487556;
@k3 = 0.00019921215;

stime = 0; dtime = 1000; etime = 56000;

1:    A + B  <=>   C           ; k> = k1;  k< = km1;
2:    A + B  <=>   AB          ; k> = k2;  k< = km2;
3:        C   ->   P           ; k> = k3;
```

Figure S14.1.5. Experimental data in dot of the *CA-RE* reaction in a 1:5 ratio (**A**/**B**) with a simulated fit as a line based of an unnumbered model described at the bottom. The calculated rate constants are seen in the middle. Figures taken directly from the exported data the custom-made program computes. The program has taken data from 4 experiments at the same time when calculating the rate constants.

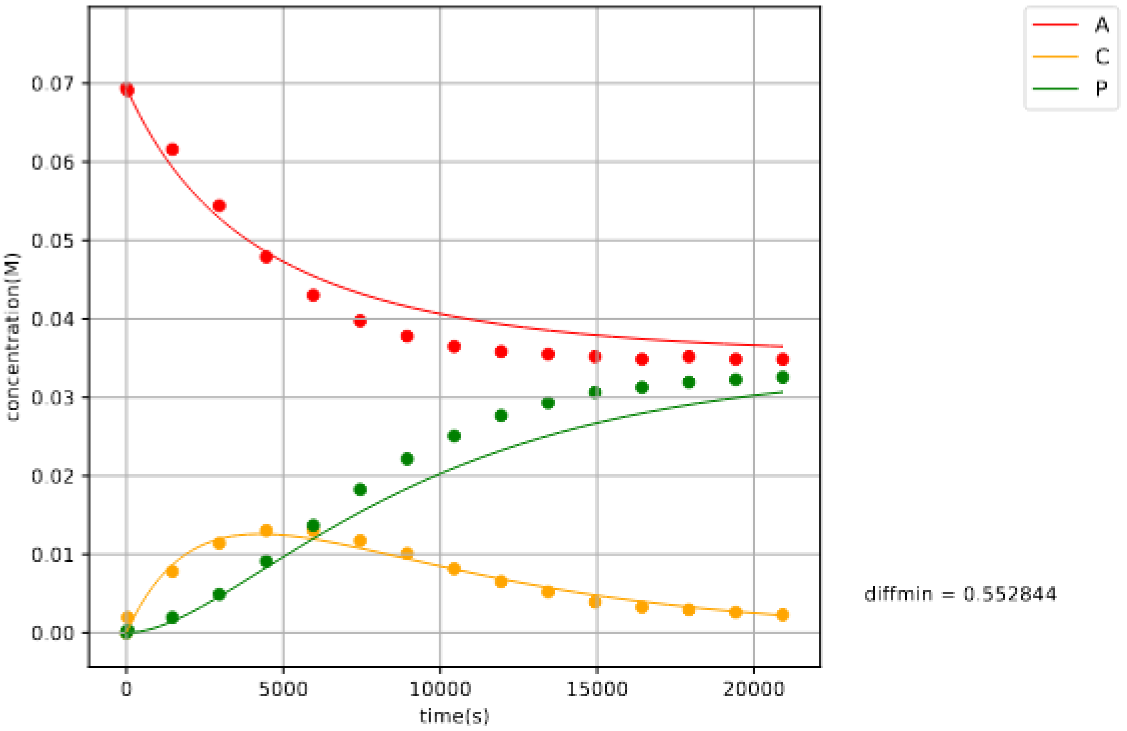


```
# A: alkyne, B: TCNE, C: intermediate, P: product

@sc =0.4;
@gr = 0.25;
@k1 = 0.00377084761;
@km1 = 0.00000000062;
@k2 = 0.000032928;
@km2 = 0.01487556;
@k3 = 0.00019921215;

stime = 0; dtime = 1000; etime = 56000;

1:   A + B  <=>   C          ; k> = k1;  k< = km1;
2:   A + B  <=>   AB         ; k> = k2;  k< = km2;
3:       C   ->   P          ; k> = k3;
```

Figure S14.1.3. Experimental data in dot of the *CA-RE* reaction in a 2:1 ratio (**A**/**B**) with a simulated fit as a line based of an unnumbered model described at the bottom. The calculated rate constants are seen in the middle. Figures taken directly from the exported data the custom-made program computes. The program has taken data from 4 experiments at the same time when calculating the rate constants.
